# Supplementary material for: Directed evolution of broadly crossreactive chemokine-blocking antibodies efficacious in arthritis
Source: Nat Commun. 2018 Apr 13;9:1461. doi: 10.1038/s41467-018-03687-x (PMC5899157; doi:10.1038/s41467-018-03687-x)
Supplement: Supplementary file 1 — Supplementary Information [file 41467_2018_3687_MOESM1_ESM.pdf]

# Supplementary Information

**Angelini *et al.*, Directed evolution of broadly crossreactive chemokine-blocking antibodies efficacious in arthritis**

# Supplementary Discussion

## Potential mechanisms of SA138 and SA157* broad crossreactivity

Conformational changes and structural plasticity might enable different optimal geometries and the exploration of alternative energetically favorable interactions that adapt to the topology of diverse targets needed for broad crossreactivity[1-4]. These rearrangements could involve local side chains and loop reorientations as well as the global rearrangement of the entire backbone and scaffold (e.g. movements of $V_L$ and $V_H$ domains). Notably, the addition of stabilizing intermolecular disulfide bridges (ds) to the broadly crossreactive SA138 and SA157* molecules had adverse consequences on both expression and binding to $ELR^+$ CXC chemokines (**Supplementary Fig. 8**). We speculate that the presence of disulfide bond pairs in highly promiscuous antibodies could have imposed rigidity by restricting the geometry of the $V_L$-$V_H$ dimer to non-optimal binding conformations. Indeed, single $V_L$ and $V_H$ domains of SA157* are stable only when produced separately and are able to bind the target when mixed equimolarly. Therefore, SA157* in particular may very well represent an extreme case of crossreactivity achieved though conformational flexibility. In contrast to the broadly crossreactive clones SA138 and SA157*, no decrease in binding affinity was observed when intermolecular disulfide bridges were added to two different locations of the more specific SA129. This is in line with previous studies reporting the acquisition of mutations that confer structural rigidity during the evolution of highly specific antibodies[2, 4, 5].

Binding promiscuity could also have been achieved through entropy-driven interactions[6]. Intriguingly, enrichment in proline residues has been observed for both highly crossreactive CK138 and CK157 molecules. Prolines are known to impose conformational constraints and

rigidity on protein loops leading to smaller losses of conformational entropy on binding and ultimately increased affinity[7]. Similar roles have been attributed to proline residues present on bNAbs and other naturally evolved crossreactive proteins[8]. We speculate that the presence of proline in the binding loops of our crossreactive antibodies might induce favorable conformations, thus facilitating binding of multiple structurally similar $ELR^+$ CXC chemokines through molecular mimicry mechanisms, a phenomena often refereed as "rigid adaptation"[9]. Co-evolution of both crossreactivity and binding affinity may require the combination of conformational flexibility and rigidity, with mutations inducing flexibility and promiscuity coexisting with and compensated by mutations that confer rigidity and increase binding affinity[10, 11].

Finally, we cannot exclude the possibility that broad crossreactivity may have been achieved through differential ligand positioning, a mechanism in which a single antibody recognizes different antigens by exploiting spatially distinct regions of its binding site[12]. However, while site-directed mutagenesis approaches are useful to map the epitopes that influence binding and specificity, more accurate measurements of energetic contributions to binding, including three-dimensional structures of crossreactive antibodies in complex with different $ELR^+$ CXC chemokines, will be required to fully understand the underlying molecular basis of binding promiscuity.

# Supplementary Figures

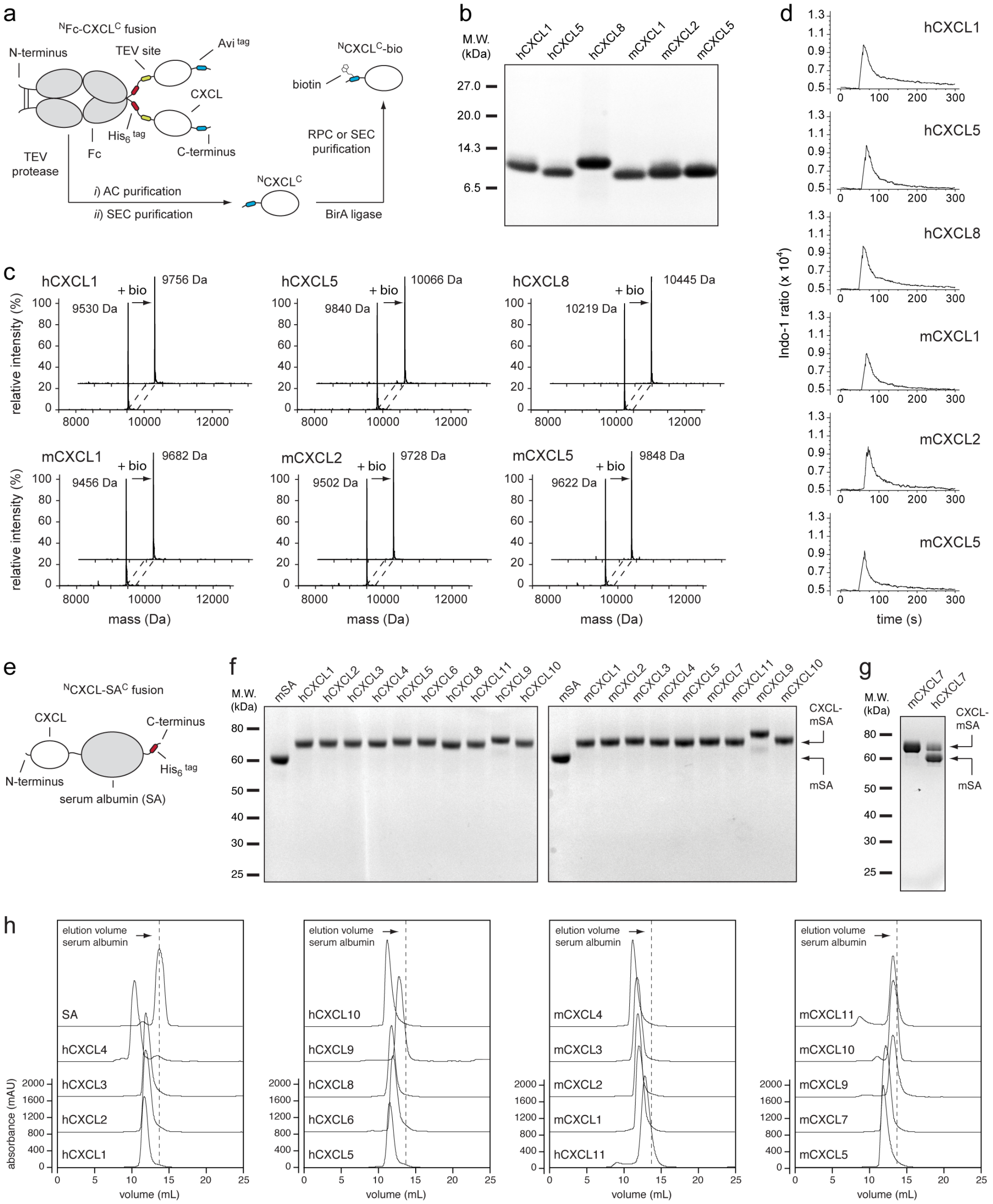


**Supplementary Figure 1. Production and characterization of CXC chemokines. a**) Schematic representation of the (*i*) Fc-ELR$^+$ CXC chemokine fusion protein constructs (Fc-CXCL) and (*ii*) purification scheme applied to obtain pure, active and biotinylated ELR$^+$ CXC

chemokines (CXCL-bio). The Fc domain derived from a murine IgG2 heavy-chain constant regions $C_H2$ and $C_H3$ is colored in grey, the hexa-histidine tag ($His_6$) in red, the Tobacco Etch Virus proteolytic cleavage site (TEV) in yellow, the $ELR^+$ CXC chemokine in white and the 15 amino acid peptide target sequence AviTag for biotin ligase BirA in blue. Superscript N and C letters indicate the N- and C-terminus respectively; **b**) Comassie blue stained SDS-PAGE of purified and biotinylated $ELR^+$ CXC chemokines. M.W., molecular-weight size markers; **c**) Mass spectra of human (hCXCL1, hCXCL5 and hCXCL8) and murine (mCXCL1, mCXCL2 and mCXCL5) $ELR^+$ CXC chemokines before and after enzymatic biotinylation. All the masses are shifted by 226 Da, which corresponds to the mass change upon reaction with one biotin molecule; **d**) Tracing representing the ratiometric measurement of calcium using fluorescent indicator Indo-1 AM. Results are expressed as Indo-1 ratio against time (seconds). Purified human and murine derived neutrophils loaded with Indo-1 were stimulated with 10 nM of biotinylated $ELR^+$ CXC chemokines. Baseline fluorescence was recorded for 60 seconds before the addition of biotinylated $ELR^+$ CXC chemokines and ratio fluorescence was measured for an additional 240 seconds; **e**) Schematic representation of the CXC chemokine expressed as N-terminal fusion to mouse serum albumin (CXCL-SA). Mouse serum albumin (mSA) is colored in grey, the $ELR^+$ CXC chemokine in white and the hexa-histidine tag ($His_6$) in red; **f**) Comassie blue stained SDS-PAGE of purified human and murine CXCL-SA fusion proteins; **g**) Comassie blue stained SDS-PAGE of purified mouse serum albumin fusion murine mCXCL7 and human hCXCL7. While murine mCXCL7 fused to SA remained stable, the orthologous human hCXCL7 underwent degradation thus explaining its ability to bind antibodies when displayed on the surface on yeast but not when expressed as soluble SA fusion; **h**) Analytical size-exclusion chromatography of the purified CXCL-SA fusion proteins. All the proteins showed a high degree of purity and most of them were eluted at an elution volume ($V_e$) of about 12-13 mL that corresponds to proteins with an apparent molecular weight of 150 kDa, which agree with the dimeric state of CXCL-SA fusions. Highly oligomeric proteins hCXCL4 and hCXCL10 fusions are eluted at a $V_e$ of about 10-11 mL that corresponds to a protein with a apparent molecular weight of 300 kDa, which agree with the tetrameric state of the fusions. mSA alone is eluted at a $V_e$ of about 13.5 mL, which corresponds to a protein with an apparent molecular weight of 66 kDa.

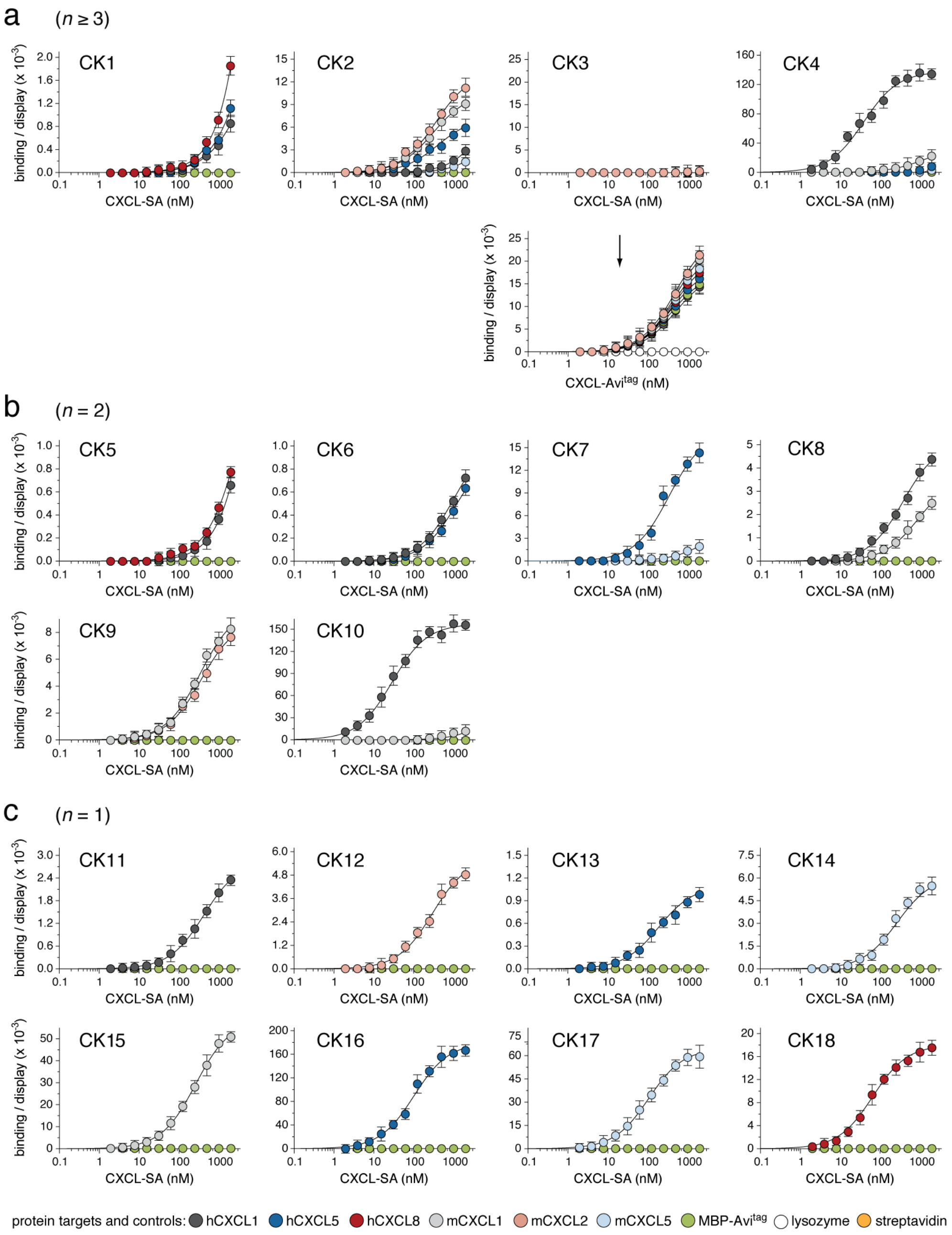


**Supplementary Figure 2**. **Binding titrations of selected antibody clones. a**) Binding titration of yeast-displayed antibody clones CK1, CK2, CK3 and CK4 that crossreact with at least three ELR$^+$ CXC chemokines ($n \geq 3$). Clone CK3 does not bind the ELR$^+$ CXC chemokine-SA fusion proteins (CXCL-SA) lacking the C-terminus biotinylated AviTag sequence; **b**) Binding titrations of yeast displayed antibody clones CK5, CK6, CK7, CK8, CK9 and CK10 that recognize two ELR$^+$ CXC chemokines ($n = 2$); **c**) Binding titrations of yeast displayed antibody clones CK11,

CK12, CK13, CK14, CK15, CK16, CK17 and CK18 that bind only one $ELR^+$ CXC chemokine (*n* = 1). Binding of selected yeast displayed antibody clones against soluble $ELR^+$ CXC chemokines were assessed by flow cytometry-based assay. The obtained fluorescence binding median intensities were normalized to the display median fluorescence intensities. $ELR^+$ CXC chemokines and the corresponding binding/display values (*y*-axis) are indicated as differently colored filled circles and represent the means of at least three independent experiments plotted against varying concentrations of soluble CXCL-SA fusions (2 - 2000 nM; *x*-axis). Control proteins: biotinylated maltose binding protein bearing the AviTag (MBP-AviTag), biotinylated lysozyme and fluorescently labeled streptavidin are colored in green, white and orange, respectively.

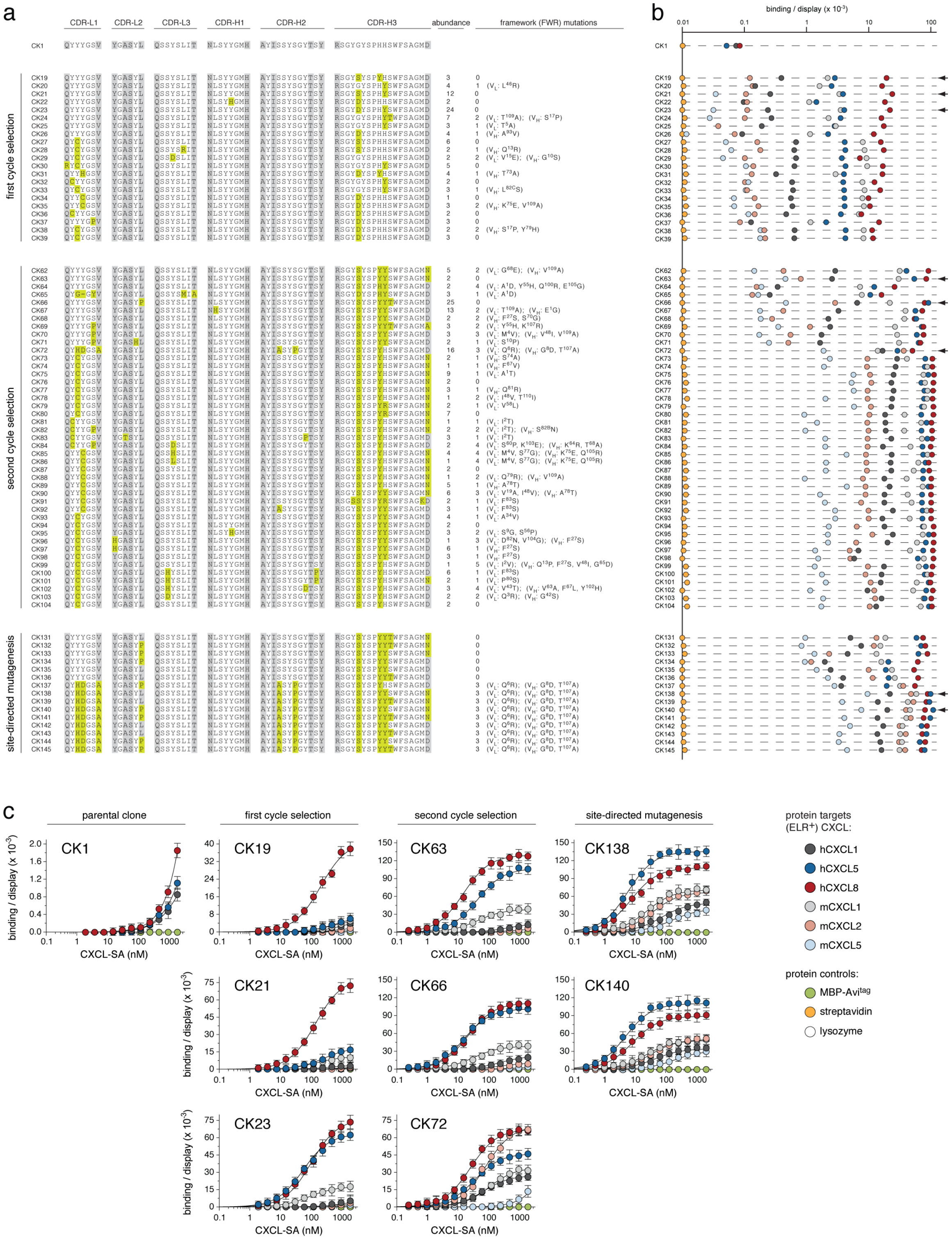

**Supplementary Figure 3. Molecular co-evolution of CK1 affinity and crossreactivity. a)** Amino acid sequence of clones isolated from two independent iterative processes of selection

(first and second cycle), each including the generation of random yeast-display antibody libraries and six cycles of flow cytometry sorting, followed by a third round of site-directed mutagenesis. The CDRs regions of light (VL: CDR-L1, CDR-L2 and CDR-L3) and heavy (VH: CDR-H1, CDR-H2 and CDR-H3) chains are shown. Mutated residues within the CDRs are shaded light green. Non-diversified positions present within the CDRs are shaded light gray. Abundance indicates the number of times an individual clone has been observed. The total number and the location of the mutations accumulated within the FWRs are indicated; **b**) Fluorescence binding signals of yeast displayed CK1 derived clones against soluble $ELR^+$ CXC chemokine were assessed by a flow cytometry-based assay. Binding intensities have been measured using single concentration of soluble biotinylated chemokine (50 nM) and obtained median values were normalized to the display median fluorescence intensities; **c**) Binding titration of the CK1 and its best derived clones isolated from each iterative process of selection. Binding of molecular co-evolved yeast displayed antibody clones against soluble $ELR^+$ CXC chemokines were assessed by flow cytometry-based assay. The obtained fluorescence binding median values were normalized to the display median fluorescence intensities. $ELR^+$ CXC chemokines and the corresponding binding/display values (*y*-axis) are indicated as differently colored filled circles and represent the means of at least three independent experiments plotted against varying concentrations of soluble CXCL-SA fusions (0.2 - 2000 nM; *x*-axis). Control proteins: biotinylated maltose binding protein bearing the AviTag (MBP-AviTag), biotinylated lysozyme and fluorescently labeled streptavidin are colored in green, white and orange, respectively.

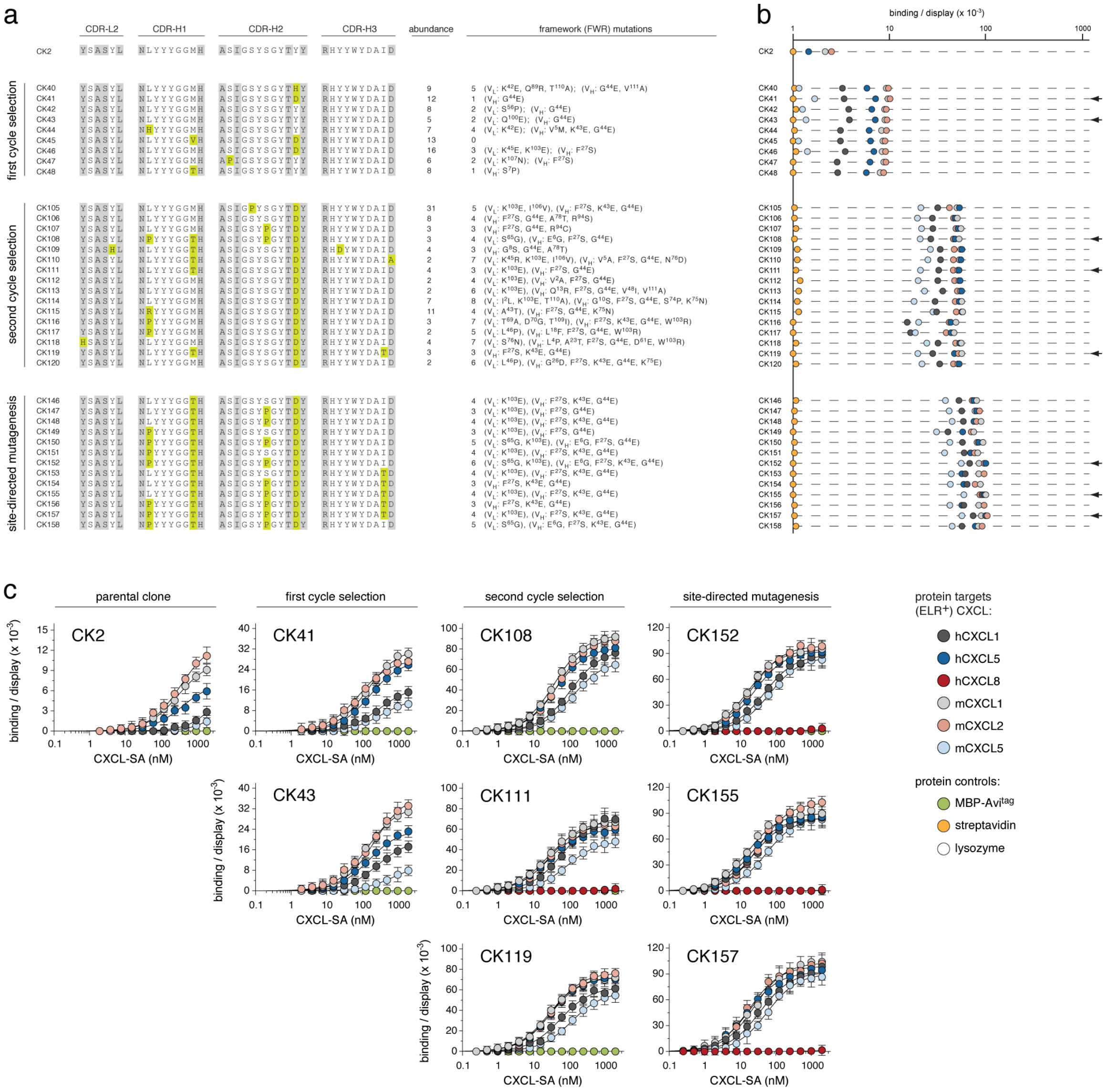

**Supplementary Figure 4. Molecular co-evolution of CK2 affinity and crossreactivity. a**) Amino acid sequence of clones isolated from two independent iterative processes of selection (first and second cycle), each including the generation of random yeast-display antibody libraries and five cycles of flow cytometry sorting, followed by a third round of site-directed mutagenesis. The CDRs regions of light (VL: CDR-L2) and heavy (VH: CDR-H1, CDR-H2 and CDR-H3) chains are shown. Mutated residues within the CDRs are shaded light green. Non-diversified positions present within the CDRs are shaded light gray. Abundance indicates the number of times an individual clone has been observed. The total number and the location of the mutations accumulated within the FWRs are indicated; **b**) Fluorescence binding signals of yeast displayed

CK2 derived clones against soluble ELR$^+$ CXC chemokine were assessed by a flow cytometry-based assay. Binding intensities have been measured using single concentration of soluble biotinylated chemokine (50 nM) and obtained median values were normalized to the display median fluorescence intensities; **c**) Binding titration of the CK2 and its best derived clones isolated from the each iterative process of selection. Binding of molecular co-evolved yeast displayed antibody clones against soluble ELR$^+$ CXC chemokines were assessed by flow cytometry-based assay. The obtained fluorescence binding median values were normalized to the display median fluorescence intensities. ELR$^+$ CXC chemokines and the corresponding binding/display values (*y*-axis) are indicated as differently colored filled circles and represent the means of at least three independent experiments plotted against varying concentrations of soluble CXCL-SA fusions (0.2 - 2000 nM; *x*-axis). Control proteins: biotinylated maltose binding protein bearing the AviTag (MBP-AviTag), biotinylated lysozyme and fluorescently labeled streptavidin are colored in green, white and orange, respectively.

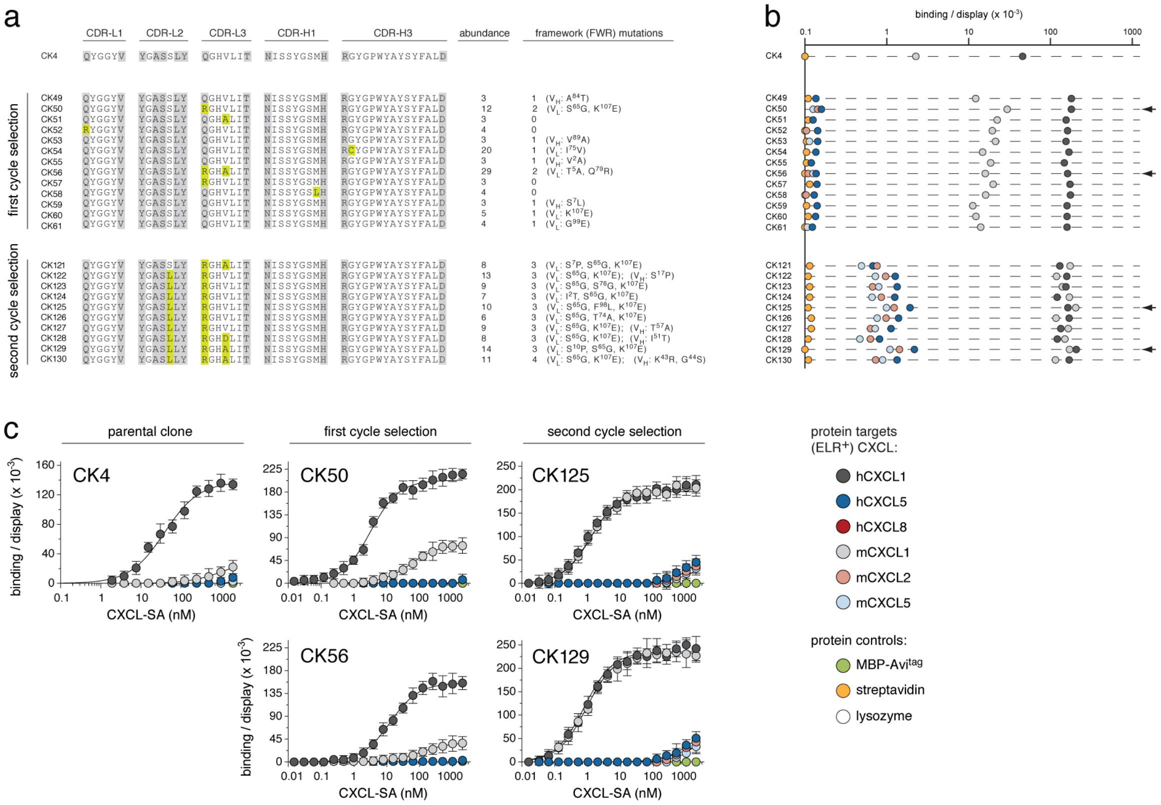


**Supplementary Figure 5. Molecular co-evolution of CK4 affinity and crossreactivity. a**) Amino acid sequence of clones isolated from two independent iterative processes of selection (first and second cycle), each including the generation of random yeast-display antibody libraries and five cycles of flow cytometry sorting. The CDRs regions of light (VL: CDR-L1, CDR-L2 and CDR-L3) and heavy (VH: CDR-H1 and CDR-H3) chains are shown. Mutated residues within the CDRs are shaded light green. Non-diversified positions present within the CDRs are shaded light gray. Abundance indicates the number of times an individual clone has been observed. The total number and the location of the mutations accumulated within the FWRs are indicated; **b**) Fluorescence binding signals of yeast displayed CK4 derived clones against soluble $ELR^+$ CXC chemokine were assessed by a flow cytometry-based assay. Binding intensities have been measured using single concentration of soluble biotinylated chemokine (50 nM) and obtained median values were normalized to the display median fluorescence intensities; **c**) Binding titration of the CK4 and its best derived clones isolated from the each iterative process of selection. Binding of molecular co-evolved yeast displayed antibody clones against soluble $ELR^+$ CXC chemokines were assessed by flow cytometry-based assay. The obtained

fluorescence binding median values were normalized to the display median fluorescence intensities. $ELR^+$ CXC chemokines and the corresponding binding/display values (*y*-axis) are indicated as differently colored filled circles and represent the means of at least three independent experiments plotted against varying concentrations of soluble CXCL-SA fusions (0.02 - 2000 nM; *x*-axis). Control proteins: biotinylated maltose binding protein bearing the AviTag (MBP-AviTag), biotinylated lysozyme and fluorescently labeled streptavidin are colored in green, white and orange, respectively.

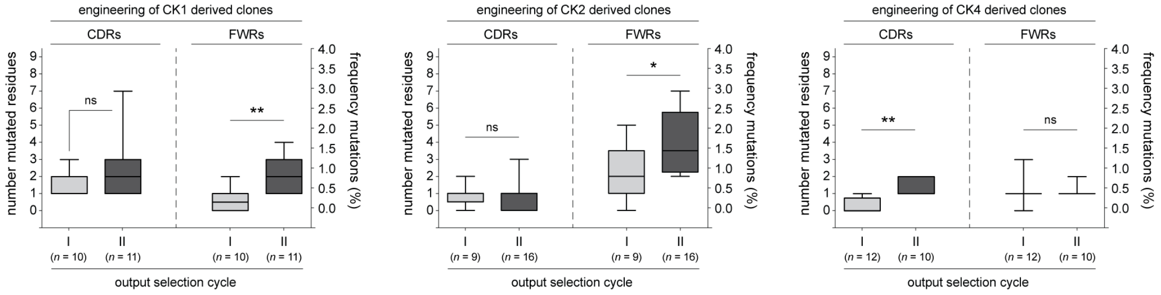


**Supplementary Figure 6. Frequency and distribution of mutations.** Box and whiskers graph comparing the value of total number (left *y*-axis) and frequencies (right *y*-axis) of accumulated mutations within CDRs (plot's left side) and FWRs (plot's right side) regions of both $V_L$ and $V_H$ domains of CK1 (left), CK2 (middle) and CK4 (right) derived clones selected after the first (I) and second (II) cycle of selection. "*n*" indicates samples size. The middle line within each box represents the median, and the lower and upper boundaries of the box indicate the 25th (Q1) and 75th (Q3) percentiles. Whiskers represent the 1.5 × interquartile range (IQR = Q3 – Q1) extending beyond box. Statistical comparisons were made between each group using one-way analysis of variance (ANOVA), followed by Tukey's test to calculate *P* values: **P* < 0.05, ** *P* < 0.01, *** *P* < 0.001; **** *P* < 0.0001. ns: non-significant.

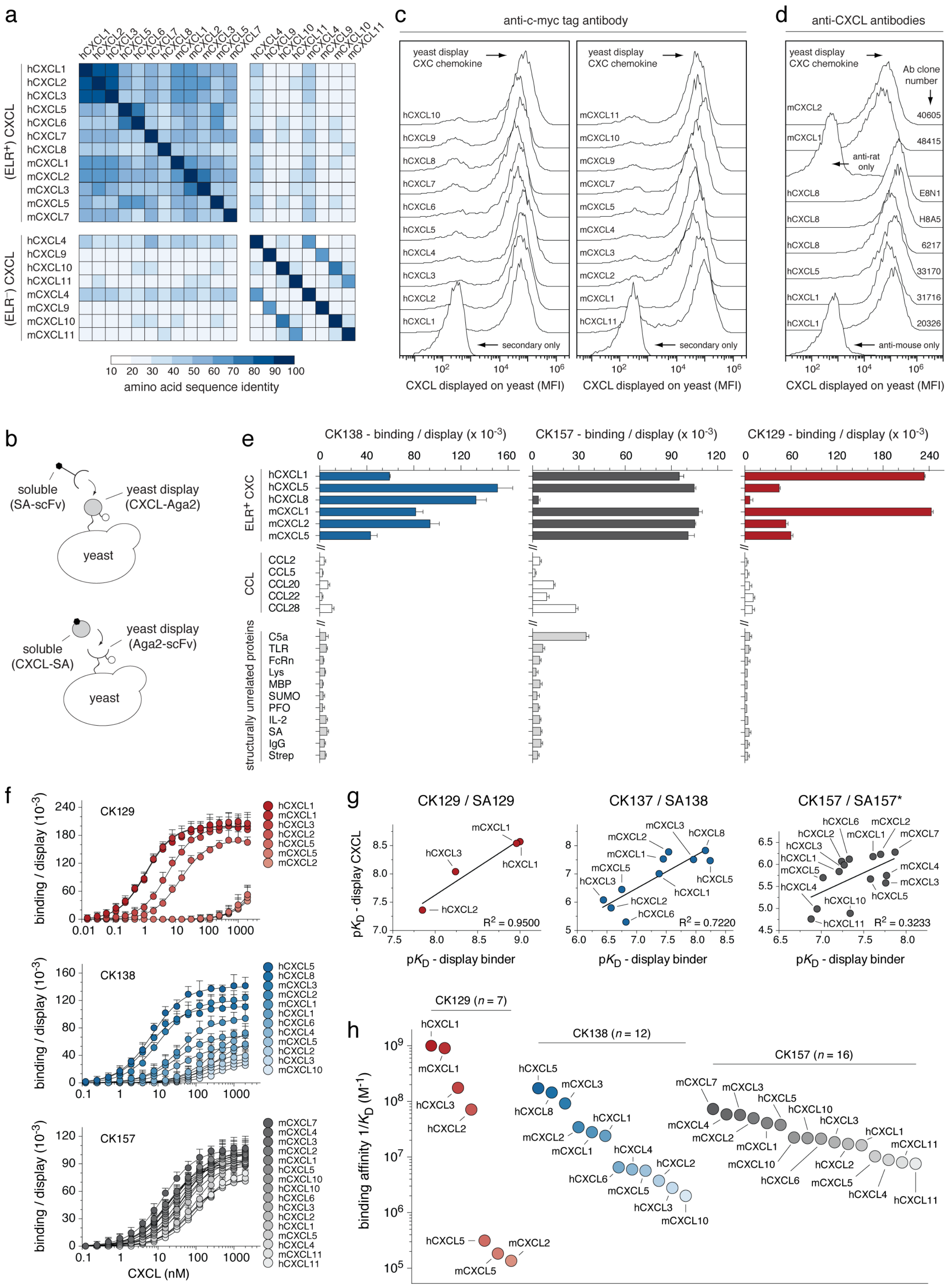
a
hCXCL1 hCXCL2 hCXCL3 hCXCL5 hCXCL6 hCXCL7 hCXCL8 mCXCL1 mCXCL2 mCXCL3 mCXCL5 mCXCL7 hCXCL4 hCXCL9 hCXCL10 hCXCL11 mCXCL4 mCXCL9 mCXCL10 mCXCL11
(ELR+) CXCL
(ELR−) CXCL
10 20 30 40 50 60 70 80 90 100
amino acid sequence identity
b
soluble (SA-scFv)
yeast display (CXCL-Aga2)
yeast
soluble (CXCL-SA)
yeast display (Aga2-scFv)
yeast
c
anti-c-myc tag antibody
yeast display CXC chemokine
secondary only
CXCL displayed on yeast (MFI)
d
anti-CXCL antibodies
Ab clone number
anti-rat only
anti-mouse only
40605 48415 E8N1 H8A5 6217 33170 31716 20326
CXCL displayed on yeast (MFI)
e
CK138 - binding / display (x 10-3)
CK157 - binding / display (x 10-3)
CK129 - binding / display (x 10-3)
ELR+ CXC
hCXCL1 hCXCL5 hCXCL8 mCXCL1 mCXCL2 mCXCL5
CCL
CCL2 CCL5 CCL20 CCL22 CCL28
structurally unrelated proteins
C5a TLR FcRn Lys MBP SUMO PFO IL-2 SA IgG Strep
f
CK129
CK138
CK157
binding / display (10-3)
CXCL (nM)
g
CK129 / SA129
CK137 / SA138
CK157 / SA157*
pKD - display CXCL
pKD - display binder
R2 = 0.9500
R2 = 0.7220
R2 = 0.3233
h
CK129 (n = 7)
CK138 (n = 12)
CK157 (n = 16)
binding affinity 1/KD (M-1)

**Supplementary Figure 7. Crossreactivity of engineered antibodies toward multiple CXC chemokines. a**) Heat map displaying the sequence identity among multiple human and murine CXC chemokines. The color of each element in the heat map indicates the percent of sequence identity, ranging from 10% (white) to 100% (dark blue); **b**) Symmetrical yeast display arrangements: *i*) soluble SA-scFv toward CXC chemokines displayed on the surface of yeast cells (top) and *ii*) soluble CXCL-SA fusions against yeast-displayed antibodies (gray; bottom); **c**) Flow cytometry analysis of the expression of human and murine CXC chemokines displayed on the surface of yeast cells as N-terminal fusion to Aga2p protein after staining with anti-c-myc antibody; **d**) Proper folding of three human and two murine $ELR^+$ CXC chemokines displayed on the surface of yeast cells was confirmed by staining with commercial anti-chemokine antibodies (Ab; **Supplementary Table 13**); **e**) Columns graph reporting the fluorescence binding intensities of yeast-displayed CK138 (blue), CK157 (dark gray) and CK129 (red) against six soluble CXCL-SA fusions, five structurally related CCL chemokines (white; **Supplementary Table 13**), and eleven structurally unrelated proteins (light gray; C5a: murine complement component C5a; TLR: toll-like receptor; FcRn: neonatal Fc receptor; Lys: lysozyme; MBP: maltose binding protein; SUMO: small ubiquitin-like modifier protein; PFO: perfringolysin O toxin; IL-2: interleukin-2; SA: serum albumin; IgG: immunoglobulin; Strep: streptavidin). Fluorescence binding signals were assessed by a flow cytometry-based assay using single concentrations (2 μM) of soluble proteins. Obtained median values were normalized to the display median fluorescence intensities of each single yeast surface displayed partner (binding/display). Normalized values (*y*-axis) represent the means ± s.e.m. (bars) of at least three independent experiments; **f**) Binding isotherms of yeast-displayed CK129 (red), CK138 (blue) and CK157 (gray) antibodies to soluble human and murine CXCL-SA fusions (0.01 - 2000 nM). Equilibrium binding affinities ($K_D$) were determined only for pairs exhibiting binding signals against high concentrations of soluble CXCL-SA fusions. Obtained binding median values were normalized to the display median fluorescence intensities of each single yeast surface displayed partner (binding/display). CXC chemokines and the corresponding binding/display values (*y*-axis) are indicated as differently colored filled circles and represent the means (dots) ± s.e.m. (bars) of at least three independent experiments plotted against varying concentrations of soluble serum albumin-antibody fusions (*x*-axis); **g**) Scatter plot showing the correlation between the experimental $pK_D$ values determined using the two complementary cell display arrangements:

soluble CXCL-SA fusions against yeast-displayed antibodies (*x*-axis, "p$K_D$ – display binder scFv") and soluble SA129, SA138 and SA157* fusions against CXC chemokines displayed on the surface of yeast cells (*y*-axis, "p$K_D$ – display CXCL"). $R^2$ indicates the coefficient of determination; **h**) Plot of the binding affinities of yeast-displayed antibodies CK129 (red), CK138 (blue) and CK157 (gray) to soluble human and murine CXCL-SA fusions. The measured $K_D$ values are indicated as differently colored filled circles and presented as inverted of equilibrium binding constants (1/$K_D$; $M^{-1}$). CXC chemokines are gradient colored ranging from dark (high affinity) to light (low affinity) red (SA129), blue (SA138) and gray (SA157) colors.

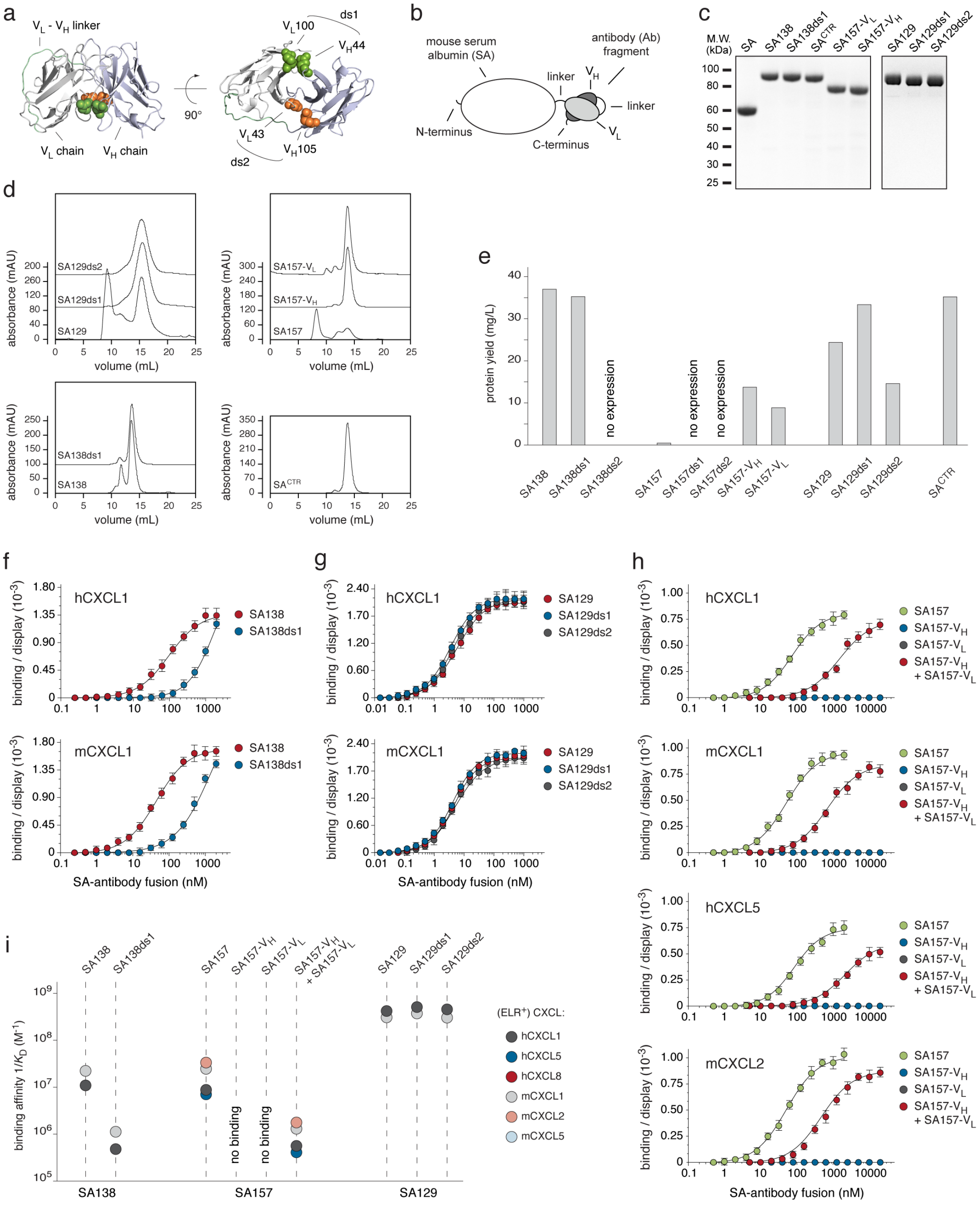

**Supplementary Figure 8. Production and characterization of serum albumin-antibody fusions. a**) Variable light $V_L$ (gray) and heavy $V_H$ (light blue) chains of a single-chain variable synthetic antibody fragment (PDB ID: 2KH2)[13], separated by a flexible linker are represented as ribbons. The structure is shown in two orientations (90° rotation) to allow the visualization of the

two most favorable locations selected for the introduction of pairs of cysteine residues into each single antibody fragment: ds1: $V_L$100 and $V_H$44 (green spheres); ds2: $V_L$43 and $V_H$105 (orange spheres; Kabat numbering system)[14-16]. ds, intermolecular disulfide bond connecting the $V_L$ and $V_H$ variable domains. Models were rendered using PyMOL (PyMOL Molecular Graphics System, Version 1.8 Schrödinger, LLC); **b**) Schematic representation of the antibody single-chain variable fragment fused the C-terminus of mouse serum albumin to generate SA129, SA138 and control $SA^{CTR}$ fusion proteins. $SA^{CTR}$ is used as control ("CTR") and it encodes for SA fused to sm3E, an antibody fragment targeting the carcinoembryonic antigen (CEA), a protein that does not exist in mice[17]; **c**) Comassie blue stained SDS-PAGE of purified serum albumin-antibody clones used for *in vitro* and *in vivo* studies. From the left to the right: serum albumin (SA), wild-type SA138, disulfide-stabilized SA138ds1, $SA^{CTR}$ fusion, light and heavy chain of SA157 fused to the C-terminus of mouse serum albumin (SA157-$V_L$ and SA157-$V_H$); wild-type SA129, disulfide-stabilized SA129ds1 and SA129ds2. M.W., molecular-weight size markers; **d**) Analytical size-exclusion chromatography of the purified serum albumin-antibody fusion proteins. Top left, SA129ds1 and SA129ds2 showed a high degree of purity and increased percent of monomeric forms compared to the wild-type SA129. Bottom left, SA138ds1 showed a high degree of purity and increased percent of monomeric forms compared to the wild-type SA138. Top right, single $V_L$ and $V_H$ chain of CK157 antibody clone separately fused to the C-terminus of mouse serum albumin (SA157-$V_L$ and SA157-$V_H$) showed a high degree of purity and increased percent of monomeric forms compared to the wild-type SA157. Bottom right, analytical size-exclusion chromatography of the purified $SA^{CTR}$ fusion; **e**) Columns graph reporting the final protein yield (mg $L^{-1}$) of the purified SA-antibody fusions from 1 L of HEK293 mammalian cell culture. The final yield of wild-type SA157 was extremely low (< 0.5 mg $L^{-1}$) while no expression was detected for disulfide-stabilized SA138ds2, SA157ds1 and SA157ds2; **f**) Binding titration of SA138 (red circle) and SA138ds1 (blue circle) against yeast-displayed hCXCL1 (top) and mCXCL1 (bottom) chemokines; **g**) Binding titration of SA129 (red circle), SA129ds1 (blue circle) and SA129ds2 (gray circle) against yeast-displayed hCXCL1 (top) and mCXCL1 (bottom) chemokines; **h**) Binding titration of SA157 (green circle), SA157-$V_H$ (blue circle), SA157-$V_L$ (gray circle) and equimolarly combined CK157-$V_H$ + CK157-$V_L$ fusions (SA157*, red circle) against yeast-displayed hCXCL1 (top), mCXCL1, hCXCL5 and mCXCL2 (bottom) chemokines. Binding of yeast displayed $ELR^+$ CXC chemokines against

soluble SA129, SA138 and SA157 were assessed by flow cytometry-based assay. The obtained fluorescence binding median values were normalized to the display median fluorescence intensities. $ELR^+$ CXC chemokines and the corresponding binding/display values (*y*-axis) are indicated as differently colored filled circles and represent the means of at least three independent experiments plotted against varying concentrations of soluble serum albumin-antibody fusions (0.03 - 30000 nM; *x*-axis); **i**) Plot of the binding affinities of purified SA-antibody fusion proteins as determined from titration curves in panel **f**, **g** and **h**. $ELR^+$ CXC chemokines and the corresponding binding affinity values are reported as differently colored filled circles and indicate the means of at least three independent experiments. Data are presented as inverted of equilibrium binding constants ($1/K_D$; $M^{-1}$).

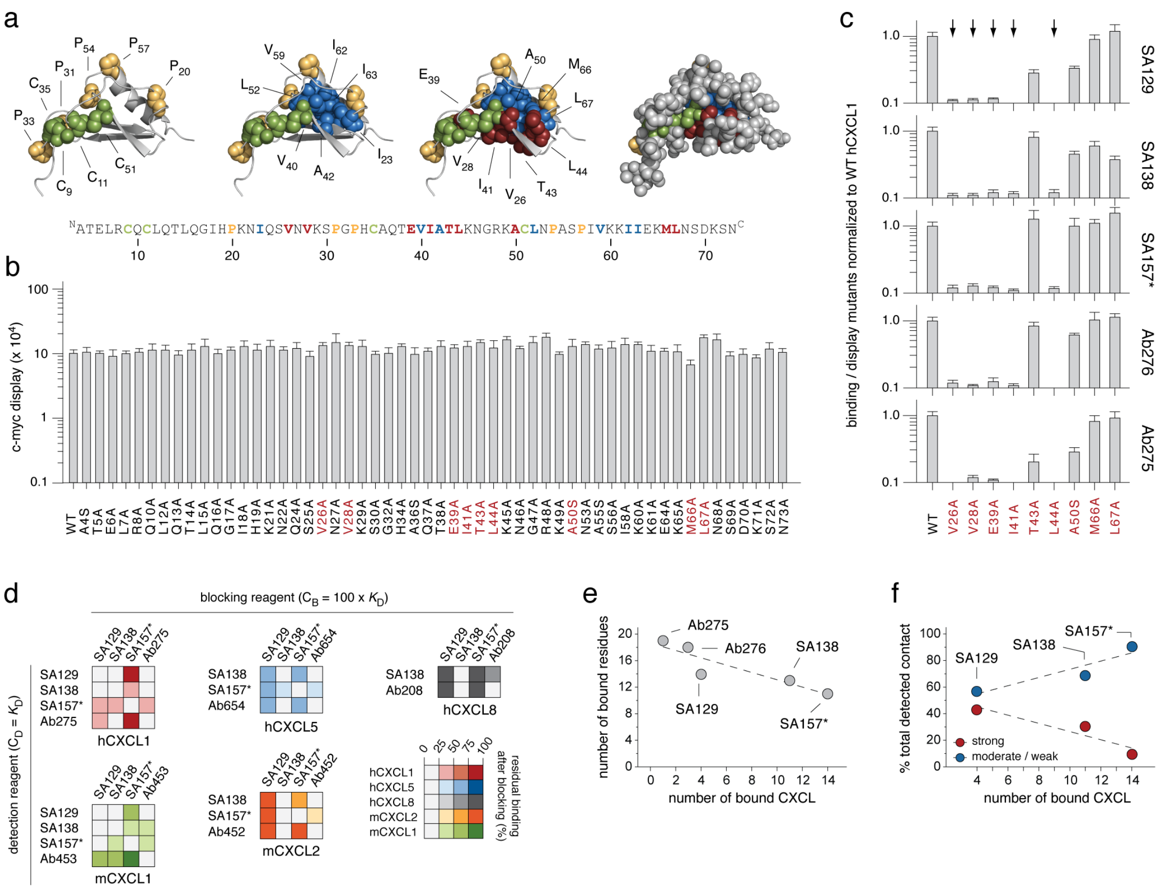


**Supplementary Figure 9. Epitope mapping of crossreactive antibodies. a**) The commonly bound human ELR$^{+}$ CXC chemokine hCXCL1 was selected for alanine scanning. The amino acid sequence and tridimensional structure of hCXCL1 (PDB ID: 1MGS[18]) represented as light gray ribbons are shown. Proline residues (P20, P31, P33, P54 and P57 shown as yellow spheres), cysteine residues (C9, C11, C35 and C52 shown as green spheres) and structurally buried hydrophobic amino acids (I23, V40, A42, L52, V59, I62 and I63 shown as blue spheres) that are crucial for overall folding and stability of the chemokine were left unaltered. Amino acids V26, V28, E39, I41, T43, L44, A50, M66 and L67 for which the relative solvent exposure and degree of burial in the structure is ambiguous are shown as dark red spheres and still mutated to alanine. The remaining forty-five solvent exposed residues that have been mutated to alanine are shown as light gray spheres. Solvent accessibility of amino acid residues of human hCXCL1 was determined by using both ASAView[19] and PyMOL (PyMOL Molecular Graphics System, Version 1.8 Schrödinger, LLC) tools; **b**) Columns graph reporting the level of expression of each distinct hCXCL1 alanine mutant (*x*-axis) displayed on the surface of yeast cell. Fluorescence

binding intensities have been determined by flow cytometry (anti-c-myc antibody). The determined display values represent the mean ± s.e.m. (bars) of three independent experiments; **c**) Columns graph reporting the fluorescence binding intensities of yeast-displayed V26, V28, E39, I41, T43, L44, A50, M66 and L67 alanine-mutants and wild-type hCXCL1 against engineered promiscuous serum albumin-antibody fusions SA129, SA138, SA157* and commercial neutralizing monoclonal antibodies Ab276 and Ab275. Fluorescence binding intensities were assessed by a flow cytometry-based assay using concentrations of soluble antibodies that are equals to their $K_D$ values. The obtained fluorescence binding median values were normalized to the display median fluorescence intensities of each single hCXCL1 alanine mutant (binding/display) and further normalized to the value measured for the wild-type hCXCL1. Normalized values (*y*-axis) represent the means ± s.e.m. (bars) of at least three independent experiments. Five hCXCL1 mutants V26, V28, E39, I41, and L44 that exhibited an intense loss of binding upon incubation with all antibodies have been removed while alanine-mutants T43, A50, M66 and L67 have been further characterized; **d**) Competitive binding assay of soluble SA129, SA138, SA157* fusions and neutralizing antibodies (Ab) for binding to hCXCL1 (red), hCXCL5 (blue), hCXCL8 (gray), mCXCL1 (green) and mCXCL2 (orange) chemokine displayed on the surface of yeast. Yeast cells displaying the chemokines were pre-incubated with concentration of soluble un-biotinylated antibodies ("blocking reagents"; $C_B$) that were equals to 100-times their $K_D$ values followed by addition of soluble biotinylated antibodies ("detection reagents"; $C_D$) at concentrations that were equals to their $K_D$ values. Binding was assessed by flow cytometry-based assay using fluorescently labeled streptavidin. The determined residual fluorescence binding levels after blocking are indicated in percentage (%) and represent the mean values of three independent experiments; **e**) Number of interacting residues plotted against the number of bound CXC chemokines ligands (CXCL); **f**) The percent of strong (red) and combined weak and moderate interactions (blue) of each selected protein binders (CK129 CK138 and CK157) are plotted against the number of bound CXC chemokines.

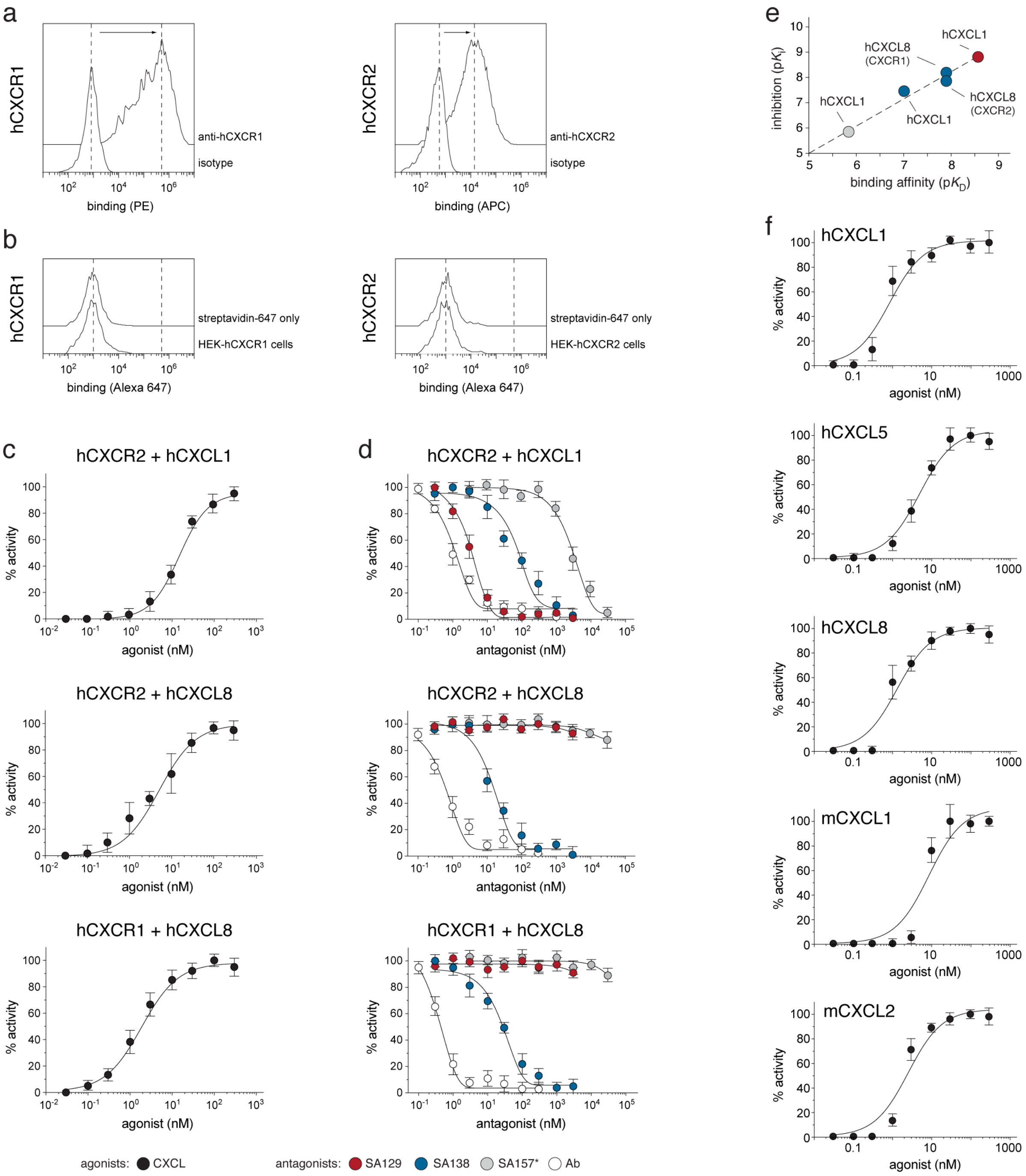


**Supplementary Figure 10. Inhibition of receptor binding and activation. a**) Histograms representing the flow cytometry analysis of surface expression of human CXCR1 (left) and CXCR2 (right) receptors in HEK cells after staining with PE anti-CXCR1 (clone 8F1) and APC anti-CXCR2 (clone 5E8) antibodies, respectively; **b**) Histograms representing the flow cytometry analysis of potential binding of secondary reagents only to HEK cells expressing

human CXCR1 and CXCR2 receptors in absence of biotinylated $ELR^+$ CXC chemokines; **c**) Dose-response titration curves dependent $ELR^+$ CXC chemokine binding using flow cytometry-based assay. Titration curves were obtained using increasing concentrations of biotinylated human hCXCL1 and hCXCL8 (0.03 – 300 nM) to human CXCR1 and CXCR2 and the $EC_{50}$ determined; **d**) Competitive binding of biotinylated human hCXCL1 and hCXCL8 to human CXCR1 and CXCR2 in the presence of varying concentrations of SA129 (0.3 – 3000 nM, red), SA138 (0.3 – 3000 nM, blue), SA157* (0.003 – 30 μM, dark gray) fusions and commercial neutralizing anti-hCXCL1 and anti-hCXCL8 (0.1 – 300 nM) antibodies (Ab, light gray). Flow cytometry-based assay was performed using concentrations of agonist biotinylated human hCXCL1 and hCXCL8 that are equals to their $EC_{50}$ values. Measured median fluorescence intensities were normalized to the maximal response obtained in the absence of antagonist and expressed as % of activity. Data represent the mean (dots) ± s.e.m. (bars) of three independent experiments; **e**) Plot displaying $pK_i$ versus $pK_D$ of SA129 (red), SA138 (blue), and SA157* (gray) fusions; **f**) Dose-response titration curves dependent $ELR^+$ CXC chemokine binding using ratiometric measurement of calcium in the presence of fluorescent indicator Indo-1 AM. Titration curves were obtained using purified human and murine derived neutrophils loaded with Indo-1 AM that have been stimulated with increasing concentrations of human hCXCL1, hCXCL5, hCXCL8 and murine mCXCL1 and mCXCL2 agonists (0.03 – 300 nM). The obtained values were normalized to the maximal response acquired, expressed as % of activity and the $EC_{50}$ values determined. Data represent the mean (dots) ± s.e.m. (bars) of three independent experiments.

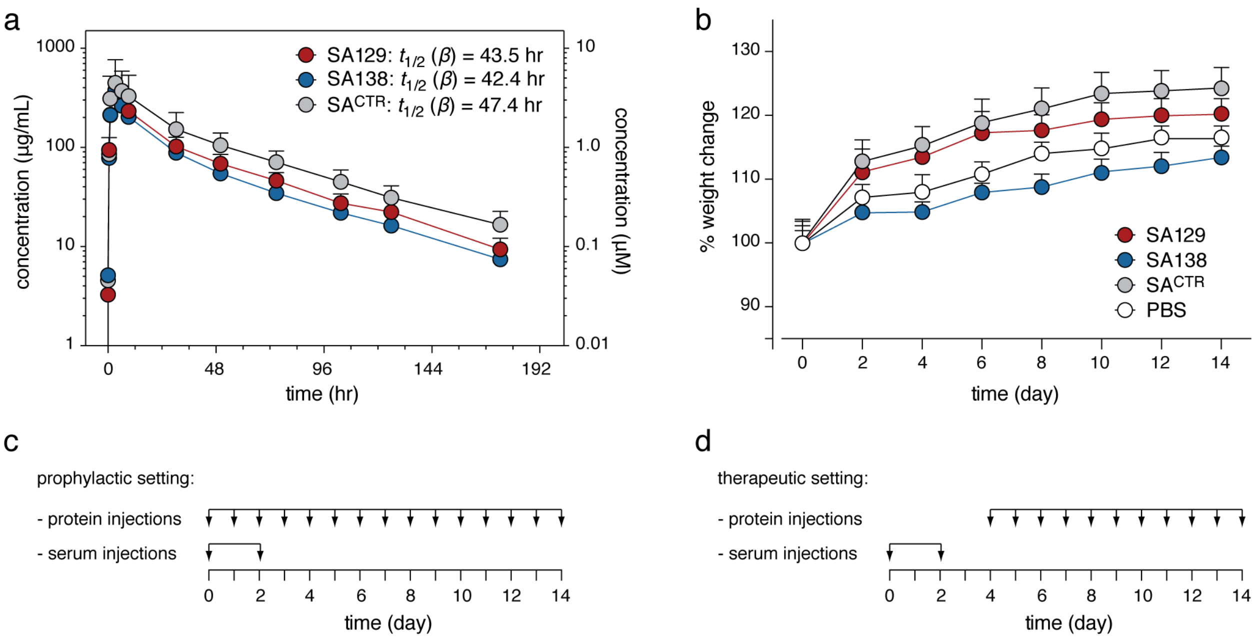


**Supplementary Figure 11. Pharmacokinetic of serum albumin-antibody fusions. a**) Pharmacokinetic profile of serum albumin-antibody fusions SA129 (red), SA138 (blue) and control $SA^{CTR}$ (light gray) in C57BL/6 mice. A single dose (1 mg per mouse) of Alexa Fluor 647-labeled serum albumin-antibody fusions (2 mg $mL^{-1}$) were injected intraperitoneally (i.p.). Blood samples were collected at various time points (immediately after injection and at 0.5, 1, 2, 3, 5, 8, 24, 48, 72, 96, 120, and 168 hours post injection) and the concentration of fusion proteins determined by measuring the fluorescent intensity on a Typhoon imager[20]. The indicated data represent the mean (dots) ± s.e.m. (bars) of three mice per group (*n* = 3). The calculated $t_{1/2}$ (*β*) half-lives are reported for each serum albumin-antibody fusion: 42.4 hrs for SA138, 43.5 hrs for SA129 and 47.4 hrs for $SA^{CTR}$. The determined protein fusion concentration is reported in µg $mL^{-1}$ (left *y*-axis) and µM (right *y*-axis) and plotted against time indicated in hours (*x*-axis); **b**) Body weight changes of K/BxN serum transferred mice treated i.p. every day for 14 days with high doses of serum albumin-antibody fusions (0.5 mL at 2 mg $mL^{-1}$, 1 mg per day per mice) normalized to starting weights. Serum albumin-antibody fusions SA129 (red), SA138 (blue) and controls $SA^{CTR}$ (light gray) and PBS (white) do not elicit systemic toxicity as measured by weight loss. Treated mice gained weight and exhibited good body conditions. Weight was measured every other day and the indicated data represent the mean (dots) ± s.e.m. (bars) of five mice per group (*n* = 5); **c**) Schematic representation of the *in vivo* experimental prophylactic

setting design. Arthritogenic serum was injected on C57BL/6J twice, on days 0 and 2. Serum transferred K/BxN mice were treated i.p. daily, starting on day 0, with SA129, SA138 and $SA^{CTR}$ fusions (0.5 mL at 2 mg $mL^{-1}$, 1 mg per day per mice); **d**) Schematic representation of the *in vivo* experimental therapeutic setting design. Arthritogenic serum was injected on C57BL/6J twice, on days 0 and 2. K/BxN serum transferred mice were treated i.p. daily, starting on day 4, with SA129, SA138 and $SA^{CTR}$ fusions (0.5 mL at 2 mg $mL^{-1}$, 1 mg per day per mice). Arrows indicate days of injections. Ankles thickness and forelimbs of mice were measured every two days for a total of 14 days.

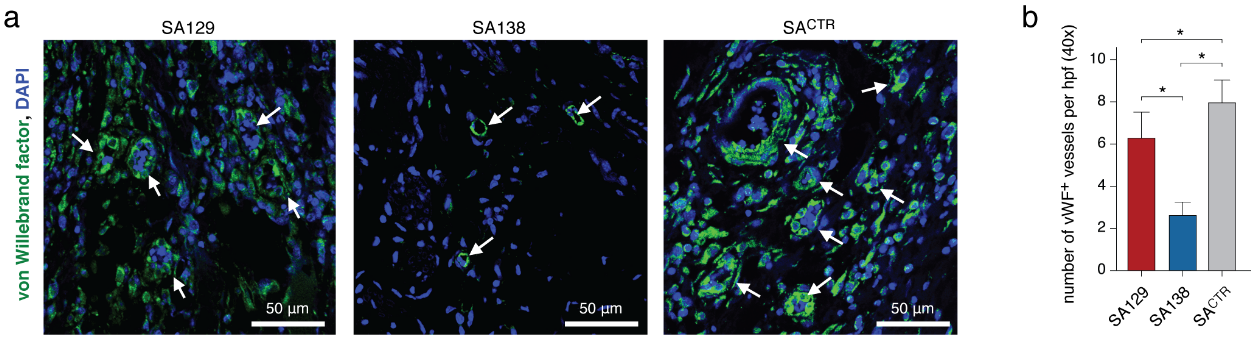


**Supplementary Figure 12. Crossreactive serum albumin-antibody fusion prevents angiogenesis.** Arthritogenic serum was injected into C57/BL6 on days 0 and 2. Mice were treated i.p. daily with SA129, SA138 and $SA^{CTR}$ fusions (0.5 mL at 2 mg $mL^{-1}$, 1 mg per day per mice) beginning on day 0. **a**) Representative immunofluorescent staining of ankle tissue sections harvested on day 8 from mice treated with SA129, SA138 and control $SA^{CTR}$. Scale bar represents 50 µm. White arrows indicate von Willebrand factor-positive ($vWF^{+}$) vessels; **b**) Quantitation of $vWF^{+}$ vessels per high-powered field (hpf) in the joint ($n$ = 3 mice per condition). Statistical comparisons were made between each group using one-way analysis of variance (ANOVA), followed by Tukey's test to calculate $P$ value: $*P < 0.05$.

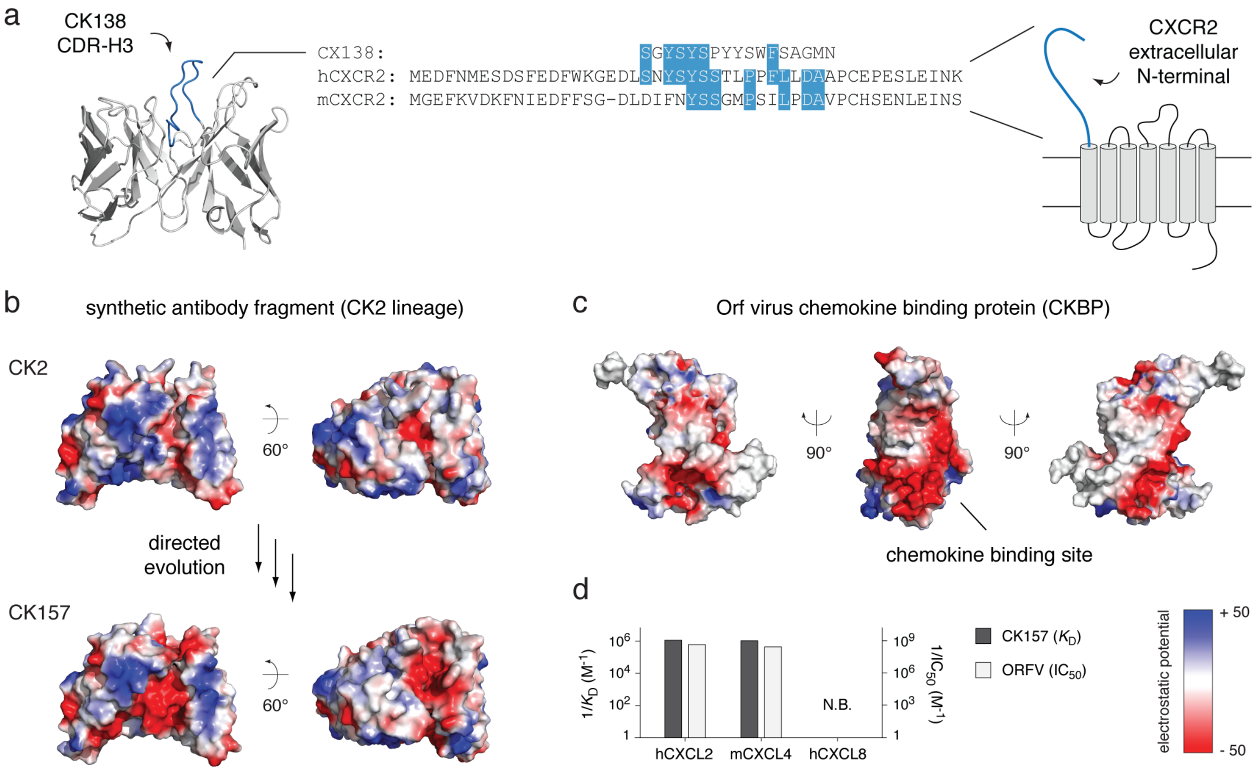


**Supplementary Figure 13. Similarities with naturally occurring chemokine-binding proteins. a**) The amino-acid composition and distribution of the CDR-H3 loop (blue) of the crossreactive CK138 antibody (left, represented as ribbons in light gray) resembles that of the N-terminus extracellular binding loop (blue) of CXCR2 receptor (right, schematically depicted)[21]. Sequence alignment of CDR-H3 loop and the N-terminus extracellular binding loop of both human ("h") and murine ("m") CXCR2 receptors are shown in the middles. Identical amino acid residues that are known to be important for the interaction with soluble ELR$^{+}$ CXC chemokine are shaded light blue; **b**) During the engineering process, the parental clone CK2 (top left) accumulated numerous surface exposed FWR mutations. These mutations caused the replacement of positive charged with negatively charged residues. The developed negative charged patches present on the surface of the evolved promiscuous antibody CK157 (bottom left) resembles that of the naturally evolved broad-spectrum viral chemokine binding proteins (vCKBP)[22-24]; **c**) Crystal structure of the promiscuous parapoxvirus orf virus (ORFV) CKBP protein (PDB ID: 4P5I)[22]. Similarly to the highly crossreactive CK157, the ORFV CKBP protein binds mCXCL2 ($IC_{50}$ = 1 nM) and mCXCL4 ($IC_{50}$ = 2.5 nM) but not hCXCL8. Functional studies have shown that ORFV CKBP exploits highly negatively charged groove and solvent

exposed hydrophobic amino acids to interact with both the CXCR2 and the GAG binding sites present on the surface of numerous $ELR^+$ CXC chemokines. Similarly to ORFV CKBP, CK157 antibody appears to recognize positively charged GAG binding residues present on the surface of $ELR^+$ CXC chemokines. Tridimensional structure models and electrostatic potential molecular surfaces of CK2, CK157 and ORFV CKBP proteins were generated and rendered using MODELLER and PyMOL, respectively. The structure of CK2 and CK157 are shown in two orientations (60° rotation) while the structure of ORFV CKBP is shown in three orientations (-90°, 0 and 90° rotation); **d**) Columns graph comparing the binding affinity ($K_D$) and the half maximal inhibitory concentration ($IC_{50}$) values measured for CK157 (dark gray) and ORFV CKBP (light gray) when tested against three CXC chemokines (mCXCL2, mCXCL4 and hCXCL8)[22], respectively. Data are presented as inverted of equilibrium binding constants ($1/K_D$; $M^{-1}$) and half maximal inhibitory concentrations ($1/IC_{50}$; $M^{-1}$).

## Supplementary Tables

| Chemokine nomenclature | |
|---|---|
| **Systematic name** | **Idiosyncratic name** |
| hCXCL1 | GROα |
| hCXCL2 | GROβ |
| hCXCL3 | GROγ |
| hCXCL5 | ENA-78 |
| hCXCL6 | GCP-2 |
| hCXCL7 | NAP-2 |
| hCXCL8 | IL-8 |
| mCXCL1 | KC |
| mCXCL2 | MIP-2 |
| mCXCL3 | DCIP-1 |
| mCXCL5 | LIX |
| mCXCL7 | NAP-2 |
| hCXCL4 | PF4 |
| hCXCL9 | MIG |
| hCXCL10 | IP-10 |
| hCXCL11 | I-TAC |
| mCXCL4 | PF4 |
| mCXCL9 | MIG |
| mCXCL10 | IP-10 |
| mCXCL11 | I-TAC |

**Supplementary Table 1.** The systematic names (left) of the chemokines are shown alongside their idiosyncratic nomenclature (right). "h" and "m" indicates human and murine, respectively.

| | Binding affinities – $K_D$ ± SE (nM) | | | | | | |
|---|---|---|---|---|---|---|---|
| | **hCXCL1** | **hCXCL5** | **hCXCL8** | **mCXCL1** | **mCXCL2** | **mCXCL5** | **MBP** |
| **CK1** | > 2000 | > 2000 | > 2000 | N.B. | N.B. | N.B. | N.B. |
| **CK2** | > 2000 | 605 ± 79 | N.B. | 481 ± 80 | 505 ± 86 | > 2000 | N.B. |
| **CK3** | N.B. | N.B. | N.B. | N.B. | N.B. | N.B. | N.B. |
| **CK3*** | 562 ± 88 | 448 ± 73 | 410 ± 61 | 589 ± 75 | 445 ± 69 | 594 ± 81 | 522 ± 79 |
| **CK4** | 39.4 ± 7.4 | > 2000 | N.B. | 744 ± 93 | N.B. | > 2000 | N.B. |
| **CK5** | > 2000 | N.B. | > 2000 | N.B. | N.B. | N.B. | N.B. |
| **CK6** | 1675 ± 191 | 1987 ± 228 | N.B. | N.B. | N.B. | N.B. | N.B. |
| **CK7** | N.B. | 344 ± 68 | N.B. | N.B. | N.B. | > 2000 | N.B. |
| **CK8** | 382 ± 73 | N.B. | N.B. | 825 ± 98 | N.B. | N.B. | N.B. |
| **CK9** | N.B. | N.B. | N.B. | 221 ± 49 | 278 ± 41 | N.B. | N.B. |
| **CK10** | 28.9 ± 4.5 | N.B. | N.B. | 801 ± 107 | N.B. | N.B. | N.B. |
| **CK11** | 425 ± 58 | N.B. | N.B. | N.B. | N.B. | N.B. | N.B. |
| **CK12** | N.B. | N.B. | N.B. | N.B. | 332 ± 57 | N.B. | N.B. |
| **CK13** | N.B. | 297 ± 98 | N.B. | N.B. | N.B. | N.B. | N.B. |
| **CK14** | N.B. | N.B. | N.B. | N.B. | N.B. | 269 ± 57 | N.B. |
| **CK15** | N.B. | N.B. | N.B. | 251 ± 25 | N.B. | N.B. | N.B. |
| **CK16** | N.B. | 102 ± 12 | N.B. | N.B. | N.B. | N.B. | N.B. |
| **CK17** | N.B. | N.B. | N.B. | N.B. | N.B. | 106 ± 11 | N.B. |
| **CK18** | N.B. | N.B. | 65 ± 6.1 | N.B. | N.B. | N.B. | N.B. |

**Supplementary Table 2.** Binding affinities of protein binders selected from a naïve library of synthetic single chain variable antibody fragments (scFv) displayed on the surface of yeast. Equilibrium binding constants ($K_D$) of each yeast-displayed antibody binder (CK) to three human (hCXCL1, hCXCL5 and hCXCL8) and three murine (mCXCL1, mCXCL2 and mCXCL5) ELR$^+$ CXC chemokine-SA fusion proteins ($^N$CXCL-SA$^C$) were determined by flow cytometry at 25 °C and physiological pH 7.4. Biotinylated maltose binding protein (MBP) bearing AviTag was used as control. The indicated values are means of at least three independent experiments. SE, standard error. N.B., not binding. Note: * stands for binding affinities measured using biotinylated ELR$^+$ CXC chemokines bearing AviTag at the C-terminus ($^N$CXCL-AviTag$^C$).

| Binding affinities – $K_D$ ± SE (nM) | | | | | | |
|---|---|---|---|---|---|---|
| | **hCXCL1** | **hCXCL5** | **hCXCL8** | **mCXCL1** | **mCXCL2** | **mCXCL5** |
| **CK1** | > 2000 | > 2000 | > 2000 | N.B. | N.B. | N.B. |
| **CK19** | 1262 ± 219 | 895 ± 72 | 212 ± 21 | 931 ± 81 | > 2000 | > 2000 |
| **CK21** | > 2000 | 273 ± 17 | 144 ± 8.4 | 280 ± 37 | > 2000 | > 2000 |
| **CK23** | > 2000 | 76.4 ± 5.8 | 104 ± 8.2 | 98.3 ± 33.4 | > 2000 | > 2000 |
| **CK63** | > 2000 | 42.9 ± 8.2 | 15.2 ± 3.3 | 53.5 ± 9.8 | > 2000 | > 2000 |
| **CK66** | 594 ± 39 | 19.1 ± 1.6 | 21.5 ± 3.6 | 52.5 ± 8.1 | > 2000 | > 2000 |
| **CK72** | 120 ± 10 | 35.1 ± 2.3 | 25.8 ± 3.9 | 107 ± 15 | 63.6 ± 9.8 | > 2000 |
| **CK138** | 61.9 ± 4.1 | 5.8 ± 0.9 | 7.4 ± 1.1 | 34.8 ± 3.2 | 36.2 ± 6.5 | 193 ± 22 |
| **CK140** | 64.6 ± 13 | 4.9 ± 0.6 | 8.2 ± 2.1 | 32.9 ± 2.8 | 33.2 ± 7.4 | 197 ± 10 |
| **CK2** | > 2000 | 605 ± 79 | N.B. | 481 ± 80 | 505 ± 86 | > 2000 |
| **CK41** | 304 ± 44 | 220 ± 65 | N.B. | 143 ± 14 | 75.4 ± 19 | 429 ± 24 |
| **CK43** | 368 ± 59 | 154 ± 31 | N.B. | 137 ± 11 | 213 ± 27 | 762 ± 98 |
| **CK108** | 110 ± 24 | 40.9 ± 6.4 | N.B. | 39.8 ± 7.5 | 40.6 ± 6.2 | 136 ± 19 |
| **CK111** | 62.9 ± 8.4 | 35.3 ± 2.1 | N.B. | 30.5 ± 2.8 | 23.8 ± 2.9 | 97.8 ± 11 |
| **CK119** | 56.7 ± 7.2 | 39.3 ± 6.4 | N.B. | 29.8 ± 2.1 | 27.5 ± 3.8 | 116 ± 20 |
| **CK152** | 48.4 ± 6.5 | 25.4 ± 2.8 | N.B. | 17.4 ± 2.8 | 21.6 ± 3.1 | 66.5 ± 10 |
| **CK155** | 24.1 ± 2.2 | 18.9 ± 2.5 | N.B. | 15.9 ± 2.4 | 33.5 ± 5.5 | 53.7 ± 8.9 |
| **CK157** | 36.2 ± 4.3 | 16.9 ± 1.7 | N.B. | 20.6 ± 4.1 | 18.2 ± 3.3 | 57.1 ± 3.9 |
| **CK4** | 39.4 ± 7.4 | > 2000 | N.B. | 744 ± 93 | N.B. | > 2000 |
| **CK50** | 3.1 ± 0.5 | > 2000 | N.B. | 53.8 ± 3.5 | > 2000 | > 2000 |
| **CK56** | 12.6 ± 2.5 | > 2000 | N.B. | 108 ± 4.5 | > 2000 | |
| **CK125** | 1.23 ± 0.2 | > 2000 | N.B. | 1.31 ± 0.1 | > 2000 | > 2000 |
| **CK129** | 0.79 ± 0.1 | > 2000 | N.B. | 0.93 ± 0.1 | > 2000 | > 2000 |

**Supplementary Table 3.** Binding affinities of protein binders selected from the mutagenized libraries. Equilibrium binding constants ($K_D$) of each yeast-displayed antibody binder to human (hCXCL1, hCXCL5 and hCXCL8) and murine (mCXCL1, mCXCL2 and mCXCL5) $ELR^+$ CXC chemokines-SA fusion proteins ($^N$CXCL-SA$^C$) were determined by flow cytometry at 25 °C and

physiological pH 7.4. The indicated values are means of at least three independent experiments. SE, standard error. N.B., not binding.

| Binding affinities – $K_D$ ± SE (nM) | | | | |
|---|---|---|---|---|
| | **hCXCL1** | **hCXCL5** | **mCXCL1** | **mCXCL2** |
| **SA129** | 2.4 ± 0.4 | N.B. | 3.1 ± 0.7 | N.B. |
| **SA129ds1** | 1.9 ± 0.4 | N.B. | 2.5 ± 0.8 | N.B. |
| **SA129ds2** | 2.1 ± 0.5 | N.B. | 3.3 ± 0.7 | N.B. |
| | | | | |
| **SA138** | 88 ± 11 | N.B. | 43 ± 3.8 | N.B. |
| **SA138ds1** | 2020 ± 102 | N.B. | 840 ± 52 | N.B. |
| | | | | |
| **SA157** | 96.7 ± 8.4 | 126 ± 25 | 35.4 ± 4.1 | 29.1 ± 3.2 |
| **SA157-$V_H$** | N.B. | N.B. | N.B. | N.B. |
| **SA157-$V_L$** | N.B. | N.B. | N.B. | N.B. |
| **SA157-$V_H$ + SA157-$V_L$** | 1540 ± 98 | 2214 ± 301 | 610 ± 74 | 553 ± 62 |

**Supplementary Table 4.** Binding affinities of variants of soluble crossreactive serum albumin-antibody fusions (SA129, SA138 and SA157) against human (hCXCL1 and hCXCL5) and murine (mCXCL1 and mCXCL2) $ELR^+$ CXC chemokine-SA fusion proteins ($^N$CXCL-$SA^C$). Equilibrium binding constants ($K_D$) were determined by flow cytometry at 25 °C and physiological pH 7.4. The indicated values are means of at least three independent experiments. SE, standard error. N.B., not binding.

| Binding affinities – $K_D$ ± SE (nM) | | | | | | |
|---|---|---|---|---|---|---|
| | CK129 / SA129 | | CK138 / SA138 | | CK157 / SA157* | |
| Display | CK129 | CXCL | CK138 | CXCL | CK157 | CXCL |
| Soluble | CXCL | SA129 | CXCL | SA138 | CXCL | SA157* |
| **hCXCL1** | 1.0 ± 0.1 | 2.7 ± 0.3 | 41.5 ± 4.5 | 96.7 ± 2.4 | 61.1 ± 5.6 | 1433 ± 108 |
| **hCXCL2** | 13.9 ± 1.1 | 43.6 ± 4.1 | 267 ± 38 | 1591 ± 315 | 57.9 ± 4.8 | 853 ± 67 |
| **hCXCL3** | 5.7 ± 0.5 | 9.2 ± 0.9 | 349 ± 41 | 836 ± 130 | 53.9 ± 2.1 | 1034 ± 87 |
| **hCXCL5** | > 2000 | N.B. | 5.8 ± 0.5 | 33.7 ± 2.2 | 26.3 ± 2.3 | 2125 ± 269 |
| **hCXCL6** | N.B. | N.B. | 153 ± 15 | > 2000 | 46.6 ± 3.1 | 751 ± 88 |
| **hCXCL7** | N.B. | N.B. | N.B. | 40.6 ± 1.6 | N.B. | N.B. |
| **hCXCL8** | N.B. | N.B. | 6.9 ± 0.5 | 12.7 ± 0.9 | N.B. | N.B. |
| **mCXCL1** | 1.1 ± 0.1 | 2.9 ± 0.3 | 35.7 ± 3.3 | 29.4 ± 2.8 | 24.3 ± 1.9 | 666 ± 47 |
| **mCXCL2** | > 2000 | N.B. | 29.1 ± 4.1 | 14.7 ± 0.5 | 19.9 ± 1.7 | 591 ± 62 |
| **mCXCL3** | N.B. | N.B. | 10.9 ± 1.1 | 31.4 ± 3.1 | 17.4 ± 1.4 | 2647 ± 264 |
| **mCXCL5** | > 2000 | N.B. | 176 ± 21 | 357 ± 33 | 96.9 ± 6.9 | 2018 ± 169 |
| **mCXCL7** | N.B. | N.B. | N.B. | N.B. | 13.6 ± 0.8 | 528 ± 53 |
| **hCXCL4** | N.B. | N.B. | 167 ± 28 | N.B. | 112 ± 5.1 | > 20000 |
| **hCXCL9** | N.B. | N.B. | N.B. | N.B. | N.B. | N.B. |
| **hCXCL10** | N.B. | N.B. | N.B. | N.B. | 45.5 ± 3.8 | > 20000 |
| **hCXCL11** | N.B. | N.B. | N.B. | N.B. | 131 ± 11 | > 20000 |
| **mCXCL4** | N.B. | N.B. | N.B. | N.B. | 17.1 ± 1.2 | 1770 ± 119 |
| **mCXCL9** | N.B. | N.B. | N.B. | N.B. | N.B. | N.B. |
| **mCXCL10** | N.B. | N.B. | 500 ± 55 | N.B. | 44.4 ± 3.7 | N.B. |
| **mCXCL11** | N.B. | N.B. | N.B. | N.B. | 124 ± 13 | N.B. |

**Supplementary Table 5.** Binding affinities of selected yeast display crossreactive protein binders against multiple human and murine CXC chemokines. Equilibrium binding constants ($K_D$) of both yeast-displayed crossreactive antibodies and CXC chemokine to twenty human and murine CXC chemokine-SA fusion proteins ($^{N}$CXCL-SA$^{C}$) and soluble SA129, SA138 and SA157*, respectively, were determined by flow cytometry at 25 °C and physiological pH 7.4. The indicated values are means of at least three independent experiments. SE, standard error. N.B., not binding. Note: * stands for SA157-$V_L$ and SA157-$V_H$ combined together equimolarly (SA157*).

| Epitope mapping interactions | | | | | |
|---|---|---|---|---|---|
| | **Ab275** | **Ab276** | **SA129** | **SA138** | **SA157** |
| **strong (0.0 – 0.25)** | G17 | L15 | Q13 | L12 | N46 |
| | T43 | G17 | L15 | N46 | |
| | N46 | I18 | I18 | G47 | |
| | G47 | H19 | N46 | R48 | |
| | R48 | N46 | G47 | | |
| | K49 | K61 | R48 | | |
| | | K65 | | | |
| **moderate (0.25 – 0.5)** | R8 | Q16 | T14 | R8 | I18 |
| | L12 | K21 | G17 | T14 | H19 |
| | T14 | K45 | G32 | L15 | K21 |
| | L15 | G47 | T43 | G32 | K29 |
| | Q16 | K71 | A50 | A50 | K45 |
| | I18 | | | L67 | G47 |
| | G32 | | | | I58 |
| | K45 | | | | K65 |
| | A50 | | | | K71 |
| **weak (0.5 – 0.75)** | Q10 | T14 | N22 | Q13 | N22 |
| | Q13 | N22 | K45 | I18 | |
| | N22 | R48 | E64 | M66 | |
| | N53 | A50 | | | |
| | | I58 | | | |
| | | K60 | | | |
| **Total residues** | 19 | 18 | 14 | 13 | 11 |

**Supplementary Table 6.** Name and number of amino-acid residues identified during the hCXCL1 epitope mapping experiment. Mutations are grouped based on their ability to affect the binding of serum albumin-antibody fusions (SA129, SA138 and SA157) and antibodies (Ab275, Ab276) to hCXCL1 strongly (0.0 - 0.25), moderately (0.25 - 0.50) and weakly (0.50 - 0.75). Mutations that did not significantly affect the binding (0.75 - 1.0) are not reported.

| Half maximal effective concentration – $EC_{50}$ ± SE (nM) | | |
|---|---|---|
| **hCXCL1 / hCXCR2** | **hCXCL8 / hCXCR2** | **hCXCL8 / hCXCR1** |
| 24.7 ± 4.5 | 4.1 ± 0.4 | 4.9 ± 0.8 |

**Supplementary Table 7.** Fluorescence-based binding assay of wild type human CXCR1 and CXCR2 receptors to human $ELR^+$ CXC chemokines. HEK293 cells stably transfected with plasmids encoding human CXCR1 and CXCR2[25] were analyzed for their ligand binding properties with biotinylated hCXCL1 and hCXCL8. At the top of each table column is the name of the corresponding CXCR receptor tested. The half maximal effective concentration ($EC_{50}$) was determined by measuring the fluorescence-binding signal of cells encoding CXCR1 and CXCR2 receptors incubated with increasing concentrations of biotinylated $ELR^+$ CXC chemokines, ranging from 0.03 to 300 nM. The binding was determined by flow cytometry at 25 °C and physiological pH 7.4. The indicated values are means of at least three independent experiments. SE, standard error. N.B., not binding.

| Inhibitory activities – $K_i$ ± SE (nM) | | | |
|---|---|---|---|
| | **hCXCL1 / hCXCR2** | **hCXCL8 / hCXCR2** | **hCXCL8 / hCXCR1** |
| **SA129** | 1.54 ± 0.3 | N.B. | N.B. |
| **SA138** | 33.1 ± 6.5 | 6.53 ± 0.9 | 13.7 ± 1.9 |
| **SA157*** | 1420 ± 162 | N.B. | N.B. |
| **Ab** | 0.32 ± 0.08 | 0.24 ± 0.05 | 0.17 ± 0.02 |

**Supplementary Table 8.** Inhibitory activities of serum albumin-antibody fusions and commercial neutralizing antibodies towards human and murine $ELR^+$ CXC chemokines. The ability of serum albumin-antibody fusions SA129, SA138 and SA157* to compete for binding of biotinylated $ELR^+$ CXC chemokines (hCXCL1 and hCXCL8) to their cognate receptors CXCR1 and CXCR2 was determined by flow cytometry at 25 °C and physiological pH 7.4. Commercial neutralizing antibodies (Ab208 and Ab275) were included in the assay as positive control. Concentrations of biotinylated $ELR^+$ CXC chemokines that were equivalent to their $EC_{50}$ values (hCXCL1 = 25 nM; hCXCL8 = 5 nM) were used for the experiments. The inhibition constants ($K_i$) were derived from the $IC_{50}$ values using the Cheng-Prusoff equation. The indicated values are means of at least three independent experiments. SE, standard error. N.B., not binding.

| Half maximal effective concentration – $EC_{50}$ ± SE (nM) | | | | |
|---|---|---|---|---|
| **hCXCL1** | **hCXCL5** | **hCXCL8** | **mCXCL1** | **mCXCL2** |
| 0.94 ± 0.2 | 4.8 ± 0.8 | 1.29 ± 0.4 | 0.81 ± 0.9 | 2.5 ± 0.7 |

**Supplementary Table 9.** Flow cytometry-based free calcium mobilization assay of neutrophils in response to human and murine $ELR^+$ CXC chemokines. The half maximal effective concentration ($EC_{50}$) was determined by measuring the intracellular calcium level of human and murine neutrophils pre-loaded with ratiometric calcium indicator Indo-1 AM upon stimulation with increasing concentrations of five diverse $ELR^+$ CXC chemokines (hCXCL1, hCXCL5, hCXCL8, mCXCL1 and mCXCL2), ranging from 0.03 to 300 nM. The intracellular calcium levels were measured at 405/30 nm (Indo-1 low) and 485/nm (Indo-1 high) emission fluorescence after excitation at 355 nm. The indicated values are means of at least three independent experiments. SE, standard error. N.B., not binding.

| Inhibitory activities – $K_i$ ± SE (nM) | | | | | |
|---|---|---|---|---|---|
| | **hCXCL1** | **hCXCL5** | **hCXCL8** | **mCXCL1** | **mCXCL2** |
| **SA129** | 0.12 ± 0.02 | > 2000 | N.B. | 0.33 ± 0.02 | > 2000 |
| **SA138** | 44.9 ± 5.2 | 2.51 ± 0.5 | 1.62 ± 0.2 | 5.87 ± 0.6 | 3.2 ± 0.4 |
| **SA157*** | 334 ± 53 | 429 ± 61 | N.B. | 197 ± 29 | 145 ± 21 |
| **Ab** | 0.05 ± 0.006 | 1.20 ± 0.2 | 0.23 ± 0.03 | 0.08 ± 0.009 | 0.16 ± 0.03 |

**Supplementary Table 10.** Inhibitory activities of serum albumin-antibody fusions and commercial neutralizing antibodies towards human and murine $ELR^+$ CXC chemokines. The ability of promiscuous serum albumin-antibody fusions SA129, SA138 and SA157* to compete for binding of five diverse $ELR^+$ CXC chemokines (hCXCL1, hCXCL5, hCXCL8, mCXCL1 and mCXCL2) to their cognate receptors CXCR1 and CXCR2 expressed on the surface of human and murine neutrophils was determined by flow cytometry-based intracellular calcium mobilization assay. Commercial neutralizing antibodies (Ab208, Ab275, Ab452, Ab453 and Ab654) were included in the assay as positive control. Concentrations of $ELR^+$ CXC chemokines that were equivalent to their $EC_{50}$ values (hCXCL1 = 1 nM; hCXCL5 = 5 nM; hCXCL8 = 1 nM; mCXCL1 = 7.5 nM and mCXCL2 = 2.5 nM) were used for the experiments. The inhibition constants ($K_i$) were derived from the $IC_{50}$ values using the Cheng-Prusoff equation. The indicated values are means of at least three independent experiments. SE, standard error. N.B., not binding.

| Pharmacokinetic parameters | | | |
|---|---|---|---|
| | **SA129** | **SA138** | **SA$^{CTR}$** |
| *Dose* (mg kg$^{-1}$) | 50 | 50 | 50 |
| $C_{max}$ (µg mL$^{-1}$) | 372.4 | 360.4 | 443.5 |
| $t_{max}$ (hr) | 3 | 3 | 3 |
| *A* | 0.61 | 0.67 | 0.56 |
| $\alpha$ (hr$^{-1}$) | 0.138 | 0.147 | 0.090 |
| *B* | 0.39 | 0.33 | 0.44 |
| $\beta$ (hr$^{-1}$) | 0.016 | 0.016 | 0.015 |
| $t_{1/2,\alpha}$ (hr) | 5.0 | 4.7 | 7.7 |
| $t_{1/2,\beta}$ (hr) | 43.5 | 42.4 | 47.4 |
| $AUC_{0\text{-tmax}}$ (hr mg mL$^{-1}$) | 0.626 | 0.859 | 0.869 |
| $AUC_{0\text{-}\infty}$ (µg day mL$^{-1}$) | 474.4 | 406.2 | 711.3 |
| *CL* (mL day kg$^{-1}$) | 105.4 | 123.1 | 70.3 |
| Number of mice | 3 | 3 | 3 |

**Supplementary Table 11.** Pharmacokinetic parameters of serum albumin-antibody fusions SA129, SA138 and control (SA$^{CTR}$) administered as a single intraperitoneal (i.p.) dose. One milligram of each Alexa Fluor 647-labeled albumin fusion protein was injected i.p. (50 mg kg$^{-1}$) on C57BL/6 mice ($n$ = 3 per construct) and their concentrations in blood plasma samples measured at various time points. *Dose*, amount of albumin fusion protein administered; $C_{max}$, maximal concentration (µg mL$^{-1}$) in plasma measured 3 hr after application; $t_{max}$, time (hr) to reach the $C_{max}$; $t_{1/2}$, elimination half-life, AUC, area under the curve; *CL*, clearance (mL day kg$^{-1}$); hr, hour. Serum levels were determined by a standard bi exponential decay equation[20].

| Flow cytometry reagents for yeast surface display selection | | | |
|---|---|---|---|
| **Primary** | **Secondary** | **Primary** | **Secondary** |
| $^{N}CXC^{C}$-bio | Streptavidin 488 | Chicken anti-c-myc | Goat anti-chicken 647 |
| $^{N}CXC^{C}$-bio | Streptavidin 647 | Chicken anti-c-myc | Goat anti-chicken 488 |
| $^{N}CXC^{C}$-bio | Neutravidin 488 | Chicken anti-c-myc | Goat anti-chicken 647 |
| $^{N}CXC^{C}$-bio | Neutravidin 650 | Chicken anti-c-myc | Goat anti-chicken 488 |
| $^{N}CXC^{C}$-bio | Mouse anti-biotin VioBlue | Chicken anti-c-myc | Goat anti-chicken 647 |
| $^{N}CXC^{C}$-bio | Mouse anti-biotin PE | Chicken anti-c-myc | Goat anti-chicken 647 |
| $^{N}CXC^{C}$-bio | Mouse anti-biotin APC | Chicken anti-c-myc | Goat anti-chicken 488 |
| $^{N}CXC^{C}$-bio | Streptavidin 488 | Mouse anti-c-myc | Goat anti-mouse 647 |
| $^{N}CXC^{C}$-bio | Streptavidin 647 | Mouse anti-c-myc | Goat anti-mouse 488 |
| $^{N}CXC^{C}$-bio | Neutravidin 488 | Mouse anti-c-myc | Goat anti-mouse 647 |
| $^{N}CXC^{C}$-bio | Neutravidin 650 | Mouse anti-c-myc | Goat anti-mouse 488 |

| Flow cytometry reagents for yeast surface display equilibrium binding titration | | | |
|---|---|---|---|
| **Primary** | **Secondary** | **Primary** | **Secondary** |
| $^{N}CXC^{C}$-bio | Streptavidin 647 | Chicken anti-c-myc | Goat anti-chicken 488 |
| $^{N}CXC^{C}$-bio | Streptavidin 647 | Mouse anti-c-myc | Goat anti-mouse 488 |
| $^{N}CXC$-$SA^{C}$ | Mouse anti-$His_6$ tag 647 | Chicken anti-c-myc | Goat anti-chicken 488 |

| Antibodies | Dilution | Company |
|---|---|---|
| Mouse anti-biotin IgG-VioBlue (Bio3-18E7; 0.1 mg $mL^{-1}$) | 1:10 | Miltenyi Biotec |
| Mouse anti-biotin IgG-APC (Bio3-18E7; 0.1 mg $mL^{-1}$) | 1:10 | Miltenyi Biotec |
| Mouse anti-biotin IgG-PE (BK-1/39; 0.2 mg $mL^{-1}$) | 1:25 | eBioscience |
| Neutravidin-DyLight 488 (1 mg $mL^{-1}$) | 1:100 | Thermo Fisher Scientific |
| Neutravidin-DyLight 650 (1 mg $mL^{-1}$) | 1:100 | Thermo Fisher Scientific |
| Streptavidin-Alexa Fluor 488 (2 mg $mL^{-1}$) | 1:250 | Thermo Fisher Scientific |
| Streptavidin-Alexa Fluor 647 (2 mg $mL^{-1}$) | 1:250 | Thermo Fisher Scientific |
| Chicken anti-c-myc IgY (1 mg $mL^{-1}$) | 1:500 | Gallus Immunotech |
| Mouse anti-c-myc IgG (9E10; 0.7 mg $mL^{-1}$) | 1:500 | Covance |
| Goat anti-chicken IgG-Alexa Fluor 647 (2 mg $mL^{-1}$) | 1:250 | Thermo Fisher Scientific |
| Goat anti-chicken IgG-Alexa Fluor 488 (2 mg $mL^{-1}$) | 1:250 | Thermo Fisher Scientific |
| Goat anti-mouse IgG-Alexa Fluor 488 (2 mg $mL^{-1}$) | 1:250 | Thermo Fisher Scientific |
| Goat anti-mouse IgG-Alexa Fluor 647 (2 mg $mL^{-1}$) | 1:250 | Thermo Fisher Scientific |
| Mouse anti-His tag IgG-Alexa Fluor 647 (0.2 mg $mL^{-1}$) | 1:50 | Qiagen |

**Supplementary Table 12.** Flow cytometry reagents used for yeast surface display selection and equilibrium binding titration. Combinations of detection reagents used for both primary and secondary staining are reported. Not listed biotinylated control proteins lysozyme (5 mg $mL^{-1}$) and MBP-AviTag fusion (1 mg $mL^{-1}$) were purchased from GeneTex and Avidity, respectively.

| Commercial recombinant proteins and antibodies for binding assays on yeast and mammalian cells | | |
|---|---|---|
| **Antibodies** | **Dilution** | **Company** |
| Mouse anti-human CD16 IgG-APC (3G8; 0.15 mg $mL^{-1}$) | 1:500 | BioLegend |
| Mouse anti-human CD66b IgM-FITC antibody (G10F5; 0.3 mg $mL^{-1}$) | 1:500 | BioLegend |
| Mouse anti-human CD45 IgG-PE (HI30; 0.01 mg $mL^{-1}$) | 1:50 | BioLegend |
| Rat anti-mouse CD11b IgG-APC (M1/70; 0.2 mg $mL^{-1}$) | 1:500 | BioLegend |
| Rat anti-mouse Ly-6G/Ly-6C (Gr-1) IgG-PE (RB6-8C5; 0.2 mg $mL^{-1}$) | 1:500 | BioLegend |
| Mouse anti-human CXCR1 IgG-PE (8F1; 0.025 mg $mL^{-1}$) | 1:20 | BioLegend |
| Mouse anti-human CXCR2 IgG-APC (5E8; 0.025 mg $mL^{-1}$) | 1:20 | BioLegend |

| **Antibodies** | **Concentration** | **Company** |
|---|---|---|
| Mouse anti-hCXCL1 $IgG_{2B}$ (clone 20326, MAB275) | 0.5 mg $mL^{-1}$ | R&D Systems |
| Mouse anti-hCXCL1 $IgG_1$ (clone 31716, MAB276) | 0.5 mg $mL^{-1}$ | R&D Systems |
| Mouse anti-hCXCL5 $IgG_1$ (clone 33170, MAB654) | 0.5 mg $mL^{-1}$ | R&D Systems |
| Mouse anti-hCXCL8 $IgG_1$ (clone 6217, MAB208) | 0.5 mg $mL^{-1}$ | R&D Systems |
| Rat anti-mCXCL1 $IgG_{2A}$ (clone 48415, MAB453) | 0.5 mg $mL^{-1}$ | R&D Systems |
| Rat anti-mCXCL2 $IgG_{2B}$ (clone 40605, MAB452) | 0.5 mg $mL^{-1}$ | R&D Systems |
| Mouse anti-hCXCL8 $IgG_1$ (clone E8N1) | 0.5 mg $mL^{-1}$ | BioLegend |
| Mouse anti-hCXCL8 $IgG_1$ (clone H8A5) | 0.5 mg $mL^{-1}$ | BioLegend |

| **Recombinant proteins** | **Concentration** | **Company** |
|---|---|---|
| hCXCL1 (275-GR-010) | 100 µg $mL^{-1}$ | R&D Systems |
| hCXCL5 (254-XB-025) | 100 µg $mL^{-1}$ | R&D Systems |
| hCXCL8 (208-IL-010) | 100 µg $mL^{-1}$ | R&D Systems |
| mCXCL1 (453-KC-010) | 100 µg $mL^{-1}$ | R&D Systems |
| mCXCL2 (452-M2-010) | 100 µg $mL^{-1}$ | R&D Systems |
| hCCL2-His Tag (10134-H08Y) | 100 µg $mL^{-1}$ | Sino Biological |
| rCCL5-His Tag (80038-R08Y) | 100 µg $mL^{-1}$ | Sino Biological |
| mCCL20-His Tag (50048-M08B) | 100 µg $mL^{-1}$ | Sino Biological |
| hCCL22-His Tag (10163-H07E) | 100 µg $mL^{-1}$ | Sino Biological |
| hCCL28-His Tag (10618-H07E) | 100 µg $mL^{-1}$ | Sino Biological |
| mC5a-His Tag (SRP4895A) | 100 µg $mL^{-1}$ | Sigma-Aldrich |

**Supplementary Table 13.** Commercial recombinant proteins and antibodies used for yeast surface display equilibrium binding titration, epitope mapping, competitive flow cytometry-

based binding assay, neutrophil characterization and competitive flow cytometry-based intracellular calcium mobilization assay. Neutralizing antibodies targeting human and murine $ELR^+$ CXC chemokines were supplied as sterile solution in 1X PBS pH 7.4 (R&D Systems) or 1X PBS pH 7.2 containing 0.09% w/v $NaN_3$ in the presence or absence of 0.2% w/v BSA (BioLegend). Recombinant endotoxin-free human and murine ELR+ CXCL chemokines were purchased from R&D Systems. Recombinant CCL chemokines were purchased from Sino Biological. Recombinant complement component C5a was purchased from Sigma-Aldrich. All recombinant proteins were reconstituted in sterile 1X PBS pH 7.4 containing 0.1% w/v BSA to a final concentration of 100 µg $mL^{-1}$. Goat anti-mouse IgG-Alexa Fluor 647 (2 mg $mL^{-1}$) and goat anti-rat IgG-Alexa Fluor 647 (2 mg $mL^{-1}$) antibodies (Thermo Fisher Scientific) have been used as secondary reagents for the detection of mouse and rat monoclonal antibodies, respectively.

## Supplementary References


1. Arevalo, J.H., Taussig, M.J. & Wilson, I.A. Molecular basis of crossreactivity and the limits of antibody-antigen complementarity. *Nature* **365**, 859-863 (1993).
2. James, L.C., Roversi, P. & Tawfik, D.S. Antibody multispecificity mediated by conformational diversity. *Science* **299**, 1362-1367 (2003).
3. James, L.C. & Tawfik, D.S. The specificity of cross-reactivity: promiscuous antibody binding involves specific hydrogen bonds rather than nonspecific hydrophobic stickiness. *Protein science : a publication of the Protein Society* **12**, 2183-2193 (2003).
4. Aharoni, A. et al. The 'evolvability' of promiscuous protein functions. *Nature genetics* **37**, 73-76 (2005).
5. James, L.C. & Tawfik, D.S. Conformational diversity and protein evolution--a 60-year-old hypothesis revisited. *Trends in biochemical sciences* **28**, 361-368 (2003).
6. Zimmermann, J. et al. Antibody evolution constrains conformational heterogeneity by tailoring protein dynamics. *Proc Natl Acad Sci U S A* **103**, 13722-13727 (2006).
7. MacArthur, M.W. & Thornton, J.M. Influence of proline residues on protein conformation. *J Mol Biol* **218**, 397-412 (1991).
8. Klein, F. et al. Somatic mutations of the immunoglobulin framework are generally required for broad and potent HIV-1 neutralization. *Cell* **153**, 126-138 (2013).
9. McFarland, B.J. & Strong, R.K. Thermodynamic analysis of degenerate recognition by the NKG2D immunoreceptor: not induced fit but rigid adaptation. *Immunity* **19**, 803-812 (2003).
10. McFarland, B.J., Kortemme, T., Yu, S.F., Baker, D. & Strong, R.K. Symmetry recognizing asymmetry: analysis of the interactions between the C-type lectin-like immunoreceptor NKG2D and MHC class I-like ligands. *Structure* **11**, 411-422 (2003).
11. Fields, B.A., Goldbaum, F.A., Ysern, X., Poljak, R.J. & Mariuzza, R.A. Molecular basis of antigen mimicry by an anti-idiotope. *Nature* **374**, 739-742 (1995).
12. Sethi, D.K., Agarwal, A., Manivel, V., Rao, K.V. & Salunke, D.M. Differential epitope positioning within the germline antibody paratope enhances promiscuity in the primary immune response. *Immunity* **24**, 429-438 (2006).
13. Wilkinson, I.C. et al. High resolution NMR-based model for the structure of a scFv-IL-1beta complex: potential for NMR as a key tool in therapeutic antibody design and development. *J Biol Chem* **284**, 31928-31935 (2009).
14. Reiter, Y. et al. Stabilization of the Fv fragments in recombinant immunotoxins by disulfide bonds engineered into conserved framework regions. *Biochemistry* **33**, 5451-5459 (1994).
15. Jung, S.H., Pastan, I. & Lee, B. Design of interchain disulfide bonds in the framework region of the Fv fragment of the monoclonal antibody B3. *Proteins* **19**, 35-47 (1994).
16. Weatherill, E.E. et al. Towards a universal disulphide stabilised single chain Fv format: importance of interchain disulphide bond location and vL-vH orientation. *Protein Eng Des Sel* **25**, 321-329 (2012).
17. Graff, C.P., Chester, K., Begent, R. & Wittrup, K.D. Directed evolution of an anti-carcinoembryonic antigen scFv with a 4-day monovalent dissociation half-time at 37 degrees C. *Protein Eng Des Sel* **17**, 293-304 (2004).

18. Fairbrother, W.J., Reilly, D., Colby, T.J., Hesselgesser, J. & Horuk, R. The solution structure of melanoma growth stimulating activity. *J Mol Biol* **242**, 252-270 (1994).
19. Ahmad, S., Gromiha, M., Fawareh, H. & Sarai, A. ASAView: database and tool for solvent accessibility representation in proteins. *BMC Bioinformatics* **5**, 51 (2004).
20. Kelly, R.L. et al. High throughput cross-interaction measures for human IgG1 antibodies correlate with clearance rates in mice. *MAbs* **7**, 770-777 (2015).
21. Prado, G.N. et al. Chemokine signaling specificity: essential role for the N-terminal domain of chemokine receptors. *Biochemistry* **46**, 8961-8968 (2007).
22. Counago, R.M. et al. Structures of Orf Virus Chemokine Binding Protein in Complex with Host Chemokines Reveal Clues to Broad Binding Specificity. *Structure* **23**, 1199-1213 (2015).
23. Ruiz-Arguello, M.B. et al. An ectromelia virus protein that interacts with chemokines through their glycosaminoglycan binding domain. *J Virol* **82**, 917-926 (2008).
24. Antonets, D.V., Nepomnyashchikh, T.S. & Shchelkunov, S.N. SECRET domain of variola virus CrmB protein can be a member of poxviral type II chemokine-binding proteins family. *BMC Res Notes* **3**, 271 (2010).
25. Ben-Baruch, A. et al. IL-8 and NAP-2 differ in their capacities to bind and chemoattract 293 cells transfected with either IL-8 receptor type A or type B. *Cytokine* **9**, 37-45 (1997).
