# Supplementary material for: Directed evolution of broadly crossreactive chemokine-blocking antibodies efficacious in arthritis
Source: Nat Commun. 2018 Apr 13;9:1461. doi: 10.1038/s41467-018-03687-x (PMC5899157; doi:10.1038/s41467-018-03687-x)
Supplement: Supplementary file 3 — Description of Additional Supplementary Files [file 41467_2018_3687_MOESM3_ESM.pdf]

Description of Additional Supplementary Files

File Name: Supplementary Data 1
Description: Protein accession numbers, oligonucleotide primers, DNA and amino-acid sequences of ^{N}Fc-CXCL^{C} fusion proteins.

File Name: Supplementary Data 2
Description: Protein accession numbers, oligonucleotide primers, DNA and amino-acid sequences of ^{N}CXCL-SA^{C} fusion proteins.

File Name: Supplementary Data 3
Description: Protein accession numbers, oligonucleotide primers, DNA and amino-acid sequences of serum albumin-antibody fusion proteins (SA-scFv).

File Name: Supplementary Data 4
Description: Oligonucleotide primers used for combinatorial site-directed mutagenesis.

File Name: Supplementary Data 5
Description: Protein accession numbers, oligonucleotide primers, DNA and amino-acid sequences of ^{N}CXCL-Aga2^{C} fusion proteins.

File Name: Supplementary Data 6
Description: Epitope mapping by Alanine scanning of hCXCL1.
