# Supplementary material for: Directed evolution of broadly crossreactive chemokine-blocking antibodies efficacious in arthritis
Source: Nat Commun. 2018 Apr 13;9:1461. doi: 10.1038/s41467-018-03687-x (PMC5899157; doi:10.1038/s41467-018-03687-x)
Supplement: Supplementary file 4 — Supplementary Data 1 [file 41467_2018_3687_MOESM4_ESM.pdf]

Supplementary Data 1 - Protein accession numbers, oligonucleotide primers, DNA and amino-acid sequences of ^{N}Fc-CXCL^{C} fusion proteins

CXCL protein (residues / accession No.)	Construct for expression	Fusion protein
hCXCL1/GROα (38-107 / P09341)	gWiz-LS-Fc(mIgG2)-His$_6$-linker-TEV-hCXCL1$^{38\text{-}107}$-G$_2$-AviTag	^{N}Fc-hCXCL1^{C}
hCXCL5/ ENA-78 (43-114 / P42830)	gWiz-LS-Fc(mIgG2)-His$_6$-linker-TEV-hCXCL5$^{43\text{-}114}$-G$_2$-AviTag	^{N}Fc-hCXCL5^{C}
hCXCL8/IL-8 (29-99 / P10145)	gWiz-LS-Fc(mIgG2)-His$_6$-linker-TEV-hCXCL8$^{29\text{-}99}$-G$_2$-AviTag	^{N}Fc-hCXCL8^{C}
mCXCL1/KC (28-96 / P12850)	gWiz-LS-Fc(mIgG2)-His$_6$-linker-TEV-mCXCL1$^{28\text{-}96}$-G$_2$-AviTag	^{N}Fc-mCXCL1^{C}
mCXCL2/MIP-2 (31-100 / P10889)	gWiz-LS-Fc(mIgG2)-His$_6$-linker-TEV-mCXCL2$^{31\text{-}100}$-G$_2$-AviTag	^{N}Fc-mCXCL2^{C}
mCXCL5/LIX (48–118 / P50228)	gWiz-LS-Fc(mIgG2)-His$_6$-linker-TEV-mCXCL5$^{48\text{-}118}$-G$_2$-AviTag	^{N}Fc-mCXCL5^{C}

Primer name	Primer sequence (5’ to 3’)
Forward-hCXCL1-Fc	5′-CTGGGTACCGAGAACCTGTACTTCCAAGCCACCGAGCTGAGATGCCAGTGCCTGC-3′
Forward-hCXCL5-Fc	5′-CTGGGTACCGAGAACCTGTACTTCCAAGTGCTGCGCGAGCTGAGATGCGTGTGC-3′
Forward-hCXCL8-Fc	5′-CTGGGTACCGAGAACCTGTACTTCCAAGCCAAAGAACTGCGGTGCCAGTGCATCAAG-3′
Forward-mCXCL1-Fc	5′-CTGGGTACCGAGAACCTGTACTTCCAAGCCAACGAGCTGCGGTGCCAGTGCCTGC-3′
Forward-mCXCL2-Fc	5′-CTGGGTACCGAGAACCTGTACTTCCAAGCCAGCGAGCTGCGGTGCCAGTGCCTG-3′
Forward-mCXCL5-Fc	5′-CTGGGTACCGAGAACCTGTACTTCCAAGCCACCGAGCTGAGATGCGTGTGCCTGACC-3′
Reverse-CXCL-Fc-AviTag	5′-GATCTTTTATTAGCCAGAAGTGATCTTTATCATCACTCGTGCCACTCGATTTTCTGG-3′

Sequences of genes coding for ELR^{+} CXCL chemokines fused to the C-terminus of Fc domain: ^{N}Fc-CXCL^{C}

DNA sequences of genes are provided. Start and stop codons are shown in black bold. The sequences include the secretory leader peptide (LS, in grey underlined), the Fc domain derived from a mouse IgG2 (mIgG2) heavy-chain C_H2 and C_H3 constant regions (in grey), the hexa-histidine tag (His_6, in red bold), a flexible linker (in black underlined), a TEV protease recognition site (in blue bold), a active CXCL (in black), a Gly-Gly dipeptide spacer (G_2, in black italic and underlined), and a BirA peptide substrate for site-specific biotinylation (AviTag, in green bold).

gWiz-LS-Fc(mIgG2)-His$_6$-linker-TEV-hCXCL1$^{38\text{-}107}$-G$_2$-AviTag

ATGAGGGTCCCCGCTCAGCTCCTGGGGCTCCTGCTGCTCTGGCTCCCAGGTGCACGATGTGAGCCCAGAGTGCCCATAACACAGAACCCCTGTCCTCCACT
CAAAGAGTGTCCCCCATGCGCAGCTCCAGACCTCTTGGGTGGACCATCCGTCTTCATCTTCCCTCCAAAGATCAAGGATGTACTCATGATCTCCCTGAGCC
CCATGGTCACATGTGTGGTGGTGGATGTGAGCGAGGATGACCCAGACGTCCAGATCAGCTGGTTTGTGAACAACGTGGAAGTACACACAGCTCAGACACAA
ACCCATAGAGAGGATTACAACAGTACTCTCCGGGTGGTCAGTGCCCTCCCCATCCAGCACCAGGACTGGATGAGTGGCAAGGAGTTCAAATGCAAGGTCAA
CAACAGAGCCCTCCCATCCCCCATCGAGAAAACCATCTCAAAACCCAGAGGGCCAGTAAGAGCTCCACAGGTATATGTCTTGCCTCCACCAGCAGAAGAGA
TGACTAAGAAAGAGTTCAGTCTGACCTGCATGATCACAGGCTTCTTACCTGCCGAAATTGCTGTGGACTGGACCAGCAATGGGCGTACAGAGCAAAACTAC
AAGAACACCGCAACAGTCCTGGACTCTGATGGTTCTTACTTCATGTACAGCAAGCTCAGAGTACAAAAGAGCACTTGGGAAAGAGGAAGTCTTTTCGCCTG
CTCAGTGGTCCACGAGGGTCTGCACAATCACCTTACGACTAAGACCATCTCCCGGTCTCTGGGTAAA**CACCATCACCATCATCAC**TCTTCTGGCGTGGATC
TGGGTACC**GAGAACCTGTACTTCCAA**GCCACCGAGCTGAGATGCCAGTGCCTGCAGACCCTGCAGGGCATCCACCCCAAGAACATCCAGAGCGTGAACGTG
AAGTCCCCTGGCCCCCACTGCGCCCAGACCGAAGTGATCGCCACCCTGAAGAACGGCCGGAAGGCCTGCCTGAACCCCGCCAGCCCCATCGTGAAGAAAAT
CATCGAGAAGATGCTGAACAGCGACAAGAGCAAC*GGCGGA***GGCCTGAACGACATCTTCGAGGCCCAGAAAATCGAGTGGCACGAG****TGATGATAA**

gWiz-LS-Fc(mIgG2)-His$_6$-linker-TEV-hCXCL5$^{43\text{-}114}$-G$_2$-AviTag

ATGAGGGTCCCCGCTCAGCTCCTGGGGCTCCTGCTGCTCTGGCTCCCAGGTGCACGATGTGAGCCCAGAGTGCCCATAACACAGAACCCCTGTCCTCCACT
CAAAGAGTGTCCCCCATGCGCAGCTCCAGACCTCTTGGGTGGACCATCCGTCTTCATCTTCCCTCCAAAGATCAAGGATGTACTCATGATCTCCCTGAGCC
CCATGGTCACATGTGTGGTGGTGGATGTGAGCGAGGATGACCCAGACGTCCAGATCAGCTGGTTTGTGAACAACGTGGAAGTACACACAGCTCAGACACAA
ACCCATAGAGAGGATTACAACAGTACTCTCCGGGTGGTCAGTGCCCTCCCCATCCAGCACCAGGACTGGATGAGTGGCAAGGAGTTCAAATGCAAGGTCAA
CAACAGAGCCCTCCCATCCCCCATCGAGAAAACCATCTCAAAACCCAGAGGGCCAGTAAGAGCTCCACAGGTATATGTCTTGCCTCCACCAGCAGAAGAGA
TGACTAAGAAAGAGTTCAGTCTGACCTGCATGATCACAGGCTTCTTACCTGCCGAAATTGCTGTGGACTGGACCAGCAATGGGCGTACAGAGCAAAACTAC
AAGAACACCGCAACAGTCCTGGACTCTGATGGTTCTTACTTCATGTACAGCAAGCTCAGAGTACAAAAGAGCACTTGGGAAAGAGGAAGTCTTTTCGCCTG

CTCAGTGGTCCACGAGGGTCTGCACAATCACCTTACGACTAAGACCATCTCCCGGTCTCTGGGTAAA**CACCATCACCATCATCAC**TCTTCTGGCGTGGATC
TGGGTACC**GAGAACCTGTACTTCCAA**GTGCTGCGCGAGCTGAGATGCGTGTGCCTGCAGACCACCCAGGGCGTGCACCCCAAGATGATCAGCAACCTCCAG
GTGTTCGCCATCGGCCCCCAGTGCAGCAAGGTGGAAGTGGTGGCCAGCCTGAAGAACGGCAAAGAGATCTGCCTGGACCCCGAGGCCCCATTCCTGAAGAA
AGTGATCCAGAAGATCCTGGACGGCGGCAACAAAGAGAAC*GGCGGA***GGCCTGAACGACATCTTCGAGGCCCAGAAAATCGAGTGGCACGAGTGATGATAA**

gWiz-LS-Fc(mIgG2)-His$_6$-linker-TEV-hCXCL8$^{29-99}$-G$_2$-AviTag

ATGAGGGTCCCCGCTCAGCTCCTGGGGCTCCTGCTGCTCTGGCTCCCAGGTGCACGATGTGAGCCCAGAGTGCCCATAACACAGAACCCCTGTCCTCCACT
CAAAGAGTGTCCCCCATGCGCAGCTCCAGACCTCTTGGGTGGACCATCCGTCTTCATCTTCCCTCCAAAGATCAAGGATGTACTCATGATCTCCCTGAGCC
CCATGGTCACATGTGTGGTGGTGGATGTGAGCGAGGATGACCCAGACGTCCAGATCAGCTGGTTTGTGAACAACGTGGAAGTACACACAGCTCAGACACAA
ACCCATAGAGAGGATTACAACAGTACTCTCCGGGTGGTCAGTGCCCTCCCCATCCAGCACCAGGACTGGATGAGTGGCAAGGAGTTCAAATGCAAGGTCAA
CAACAGAGCCCTCCCATCCCCCATCGAGAAAACCATCTCAAAACCCAGAGGGCCAGTAAGAGCTCCACAGGTATATGTCTTGCCTCCACCAGCAGAAGAGA
TGACTAAGAAAGAGTTCAGTCTGACCTGCATGATCACAGGCTTCTTACCTGCCGAAATTGCTGTGGACTGGACCAGCAATGGGCGTACAGAGCAAAACTAC
AAGAACACCGCAACAGTCCTGGACTCTGATGGTTCTTACTTCATGTACAGCAAGCTCAGAGTACAAAAGAGCACTTGGGAAAGAGGAAGTCTTTTCGCCTG
CTCAGTGGTCCACGAGGGTCTGCACAATCACCTTACGACTAAGACCATCTCCCGGTCTCTGGGTAAA**CACCATCACCATCATCAC**TCTTCTGGCGTGGATC
TGGGTACC**GAGAACCTGTACTTCCAA**GCCAAAGAACTGCGGTGCCAGTGCATCAAGACCTACAGCAAGCCCTTCCACCCCAAGTTCATCAAAGAACTGAGA
GTGATCGAGAGCGGCCCTCACTGCGCCAACACCGAGATCATCGTGAAGCTGAGCGACGGCAGAGAGCTGTGCCTGGACCCCAAAGAAAACTGGGTGCAGCG
GGTGGTGGAAAAGTTCCTGAAGCGGGCCGAGAACAGC*GGCGGA***GGCCTGAACGACATCTTCGAGGCCCAGAAAATCGAGTGGCACGAGTGATGATAA**

gWiz-LS-Fc(mIgG2)-His$_6$-linker-TEV-mCXCL1$^{28-96}$-G$_2$-AviTag

ATGAGGGTCCCCGCTCAGCTCCTGGGGCTCCTGCTGCTCTGGCTCCCAGGTGCACGATGTGAGCCCAGAGTGCCCATAACACAGAACCCCTGTCCTCCACT
CAAAGAGTGTCCCCCATGCGCAGCTCCAGACCTCTTGGGTGGACCATCCGTCTTCATCTTCCCTCCAAAGATCAAGGATGTACTCATGATCTCCCTGAGCC
CCATGGTCACATGTGTGGTGGTGGATGTGAGCGAGGATGACCCAGACGTCCAGATCAGCTGGTTTGTGAACAACGTGGAAGTACACACAGCTCAGACACAA
ACCCATAGAGAGGATTACAACAGTACTCTCCGGGTGGTCAGTGCCCTCCCCATCCAGCACCAGGACTGGATGAGTGGCAAGGAGTTCAAATGCAAGGTCAA
CAACAGAGCCCTCCCATCCCCCATCGAGAAAACCATCTCAAAACCCAGAGGGCCAGTAAGAGCTCCACAGGTATATGTCTTGCCTCCACCAGCAGAAGAGA
TGACTAAGAAAGAGTTCAGTCTGACCTGCATGATCACAGGCTTCTTACCTGCCGAAATTGCTGTGGACTGGACCAGCAATGGGCGTACAGAGCAAAACTAC
AAGAACACCGCAACAGTCCTGGACTCTGATGGTTCTTACTTCATGTACAGCAAGCTCAGAGTACAAAAGAGCACTTGGGAAAGAGGAAGTCTTTTCGCCTG
CTCAGTGGTCCACGAGGGTCTGCACAATCACCTTACGACTAAGACCATCTCCCGGTCTCTGGGTAAA**CACCATCACCATCATCAC**TCTTCTGGCGTGGATC
TGGGTACC**GAGAACCTGTACTTCCAA**GCCAACGAGCTGCGGTGCCAGTGCCTGCAGACCATGGCCGGCATCCACCTGAAGAACATCCAGAGCCTGAAGGTG
CTGCCCAGCGGCCCTCACTGCACCCAGACCGAAGTGATCGCCACCCTGAAGAACGGCAGAGAGGCCTGCCTGGATCCCGAGGCCCCCCTGGTGCAGAAAAT
CGTGCAGAAAATGCTGAAGGGCGTGCCCAAG*GGCGGA***GGCCTGAACGACATCTTCGAGGCCCAGAAAATCGAGTGGCACGAGTGATGATAA**

gWiz-LS-Fc(mIgG2)-His$_6$-linker-TEV-mCXCL2$^{31-100}$-G$_2$-AviTag

ATGAGGGTCCCCGCTCAGCTCCTGGGGCTCCTGCTGCTCTGGCTCCCAGGTGCACGATGTGAGCCCAGAGTGCCCATAACACAGAACCCCTGTCCTCCACT
CAAAGAGTGTCCCCCATGCGCAGCTCCAGACCTCTTGGGTGGACCATCCGTCTTCATCTTCCCTCCAAAGATCAAGGATGTACTCATGATCTCCCTGAGCC
CCATGGTCACATGTGTGGTGGTGGATGTGAGCGAGGATGACCCAGACGTCCAGATCAGCTGGTTTGTGAACAACGTGGAAGTACACACAGCTCAGACACAA
ACCCATAGAGAGGATTACAACAGTACTCTCCGGGTGGTCAGTGCCCTCCCCATCCAGCACCAGGACTGGATGAGTGGCAAGGAGTTCAAATGCAAGGTCAA
CAACAGAGCCCTCCCATCCCCCATCGAGAAAACCATCTCAAAACCCAGAGGGCCAGTAAGAGCTCCACAGGTATATGTCTTGCCTCCACCAGCAGAAGAGA
TGACTAAGAAAGAGTTCAGTCTGACCTGCATGATCACAGGCTTCTTACCTGCCGAAATTGCTGTGGACTGGACCAGCAATGGGCGTACAGAGCAAAACTAC
AAGAACACCGCAACAGTCCTGGACTCTGATGGTTCTTACTTCATGTACAGCAAGCTCAGAGTACAAAAGAGCACTTGGGAAAGAGGAAGTCTTTTCGCCTG
CTCAGTGGTCCACGAGGGTCTGCACAATCACCTTACGACTAAGACCATCTCCCGGTCTCTGGGTAAA**CACCATCACCATCATCAC**TCTTCTGGCGTGGATC
TGGGTACC**GAGAACCTGTACTTCCAA**GCCAGCGAGCTGCGGTGCCAGTGCCTGAAAACCCTGCCCCGGGTGGACTTCAAGAACATCCAGAGCCTGAGCGTG
ACCCCCCCTGGCCCTCACTGTGCCCAGACCGAAGTGATCGCCACCCTGAAGGGCGGCCAGAAAGTGTGCCTGGACCCCGAGGCCCCCCTGGTGCAGAAGAT
CATCCAGAAGATCCTGAACAAGGGCAAGGCCAAC*GGCGGA***GGCCTGAACGACATCTTCGAGGCCCAGAAAATCGAGTGGCACGAGTGATGATAA**

gWiz-LS-Fc(mIgG2)-His$_6$-linker-TEV-mCXCL5$^{48-118}$-G$_2$-AviTag

ATGAGGGTCCCCGCTCAGCTCCTGGGGCTCCTGCTGCTCTGGCTCCCAGGTGCACGATGTGAGCCCAGAGTGCCCATAACACAGAACCCCTGTCCTCCACT
CAAAGAGTGTCCCCCATGCGCAGCTCCAGACCTCTTGGGTGGACCATCCGTCTTCATCTTCCCTCCAAAGATCAAGGATGTACTCATGATCTCCCTGAGCC
CCATGGTCACATGTGTGGTGGTGGATGTGAGCGAGGATGACCCAGACGTCCAGATCAGCTGGTTTGTGAACAACGTGGAAGTACACACAGCTCAGACACAA
ACCCATAGAGAGGATTACAACAGTACTCTCCGGGTGGTCAGTGCCCTCCCCATCCAGCACCAGGACTGGATGAGTGGCAAGGAGTTCAAATGCAAGGTCAA
CAACAGAGCCCTCCCATCCCCCATCGAGAAAACCATCTCAAAACCCAGAGGGCCAGTAAGAGCTCCACAGGTATATGTCTTGCCTCCACCAGCAGAAGAGA
TGACTAAGAAAGAGTTCAGTCTGACCTGCATGATCACAGGCTTCTTACCTGCCGAAATTGCTGTGGACTGGACCAGCAATGGGCGTACAGAGCAAAACTAC
AAGAACACCGCAACAGTCCTGGACTCTGATGGTTCTTACTTCATGTACAGCAAGCTCAGAGTACAAAAGAGCACTTGGGAAAGAGGAAGTCTTTTCGCCTG
CTCAGTGGTCCACGAGGGTCTGCACAATCACCTTACGACTAAGACCATCTCCCGGTCTCTGGGTAAA**CACCATCACCATCATCAC**TCTTCTGGCGTGGATC
TGGGTACC**GAGAACCTGTACTTCCAA**GCCACCGAGCTGAGATGCGTGTGCCTGACCGTGACCCCCAAGATCAACCCCAAGCTGATCGCCAACCTGGAAGTG
ATCCCTGCCGGCCCTCAGTGCCCCACCGTGGAAGTGATTGCCAAGCTGAAGAACCAGAAAGAAGTGTGCCTGGACCCCGAGGCCCCCGTGATCAAGAAGAT
CATCCAGAAGATCCTGGGCAGCGACAAGAAGAAAGCC*GGCGGA***GGCCTGAACGACATCTTCGAGGCCCAGAAAATCGAGTGGCACGAGTGATGATAA**

Amino-acid sequences of ELR$^+$ CXCL chemokines fused to the C-terminus of Fc domain: ^{N}Fc-CXCL^{C}

Amino acid sequences of translated polypeptides are shown. The sequences include the secretory leader peptide (LS, in grey underlined), the Fc domain derived from a mouse IgG2 (mIgG2) heavy-chain C$_H$2 and C$_H$3 constant regions (in grey), the hexa-histidine tag (His$_6^{tag}$, in red bold), a flexible linker (in black underlined), a TEV protease recognition site (in blue bold), a active CXCL (in black), a Gly-Gly dipeptide spacer (G$_2$, in black italic and underlined), and a BirA peptide substrate for site-specific biotinylation (AviTag, in green bold).

LS-Fc-His$_6$-linker-TEV-hCXCL1$^{38\text{-}107}$-G$_2$-AviTag

MRVPAQLLGLLLLWLPGARCEPRVPITQNPCPPLKECPPCAAPDLLGGPSVFIFPPKIKDVLMISLSPMVTCVVVDVSEDDPDVQISWFVNNVEVHTAQTQ
THREDYNSTLRVVSALPIQHQDWMSGKEFKCKVNNRALPSPIEKTISKPRGPVRAPQVYVLPPPAEEMTKKEFSLTCMITGFLPAEIAVDWTSNGRTEQNY
KNTATVLDSDGSYFMYSKLRVQKSTWERGSLFACSVVHEGLHNHLTTKTISRSLGK**HHHHHH**SSGVDLGT**ENLYFQ**ATELRCQCLQTLQGIHPKNIQSVNV
KSPGPHCAQTEVIATLKNGRKACLNPASPIVKKIIEKMLNSDKSN*GG***GLNDIFEAQKIEWHE**--

LS-Fc-His$_6$-linker-TEV-hCXCL5$^{43\text{-}114}$-G$_2$-AviTag

MRVPAQLLGLLLLWLPGARCEPRVPITQNPCPPLKECPPCAAPDLLGGPSVFIFPPKIKDVLMISLSPMVTCVVVDVSEDDPDVQISWFVNNVEVHTAQTQ
THREDYNSTLRVVSALPIQHQDWMSGKEFKCKVNNRALPSPIEKTISKPRGPVRAPQVYVLPPPAEEMTKKEFSLTCMITGFLPAEIAVDWTSNGRTEQNY
KNTATVLDSDGSYFMYSKLRVQKSTWERGSLFACSVVHEGLHNHLTTKTISRSLGK**HHHHHH**SSGVDLGT**ENLYFQ**VLRELRCVCLQTTQGVHPKMISNLQ
VFAIGPQCSKVEVVASLKNGKEICLDPEAPFLKKVIQKILDGGNKEN*GG***GLNDIFEAQKIEWHE**--

LS-Fc-His$_6$-linker-TEV-hCXCL8$^{29\text{-}99}$-G$_2$-AviTag

MRVPAQLLGLLLLWLPGARCEPRVPITQNPCPPLKECPPCAAPDLLGGPSVFIFPPKIKDVLMISLSPMVTCVVVDVSEDDPDVQISWFVNNVEVHTAQTQ
THREDYNSTLRVVSALPIQHQDWMSGKEFKCKVNNRALPSPIEKTISKPRGPVRAPQVYVLPPPAEEMTKKEFSLTCMITGFLPAEIAVDWTSNGRTEQNY
KNTATVLDSDGSYFMYSKLRVQKSTWERGSLFACSVVHEGLHNHLTTKTISRSLGK**HHHHHH**SSGVDLGT**ENLYFQ**AKELRCQCIKTYSKPFHPKFIKELR
VIESGPHCANTEIIVKLSDGRELCLDPKENWVQRVVEKFLKRAENS*GG***GLNDIFEAQKIEWHE**--

LS-Fc-His$_6$-linker-TEV-mCXCL1$^{28\text{-}96}$-G$_2$-AviTag

MRVPAQLLGLLLLWLPGARCEPRVPITQNPCPPLKECPPCAAPDLLGGPSVFIFPPKIKDVLMISLSPMVTCVVVDVSEDDPDVQISWFVNNVEVHTAQTQ
THREDYNSTLRVVSALPIQHQDWMSGKEFKCKVNNRALPSPIEKTISKPRGPVRAPQVYVLPPPAEEMTKKEFSLTCMITGFLPAEIAVDWTSNGRTEQNY
KNTATVLDSDGSYFMYSKLRVQKSTWERGSLFACSVVHEGLHNHLTTKTISRSLGK**HHHHHH**SSGVDLGT**ENLYFQ**ANELRCQCLQTMAGIHLKNIQSLKV
LPSGPHCTQTEVIATLKNGREACLDPEAPLVQKIVQKMLKGVPK*GG***GLNDIFEAQKIEWHE**--

LS-Fc-His$_6$-linker-TEV-mCXCL2$^{31\text{-}100}$-G$_2$-AviTag

MRVPAQLLGLLLLWLPGARCEPRVPITQNPCPPLKECPPCAAPDLLGGPSVFIFPPKIKDVLMISLSPMVTCVVVDVSEDDPDVQISWFVNNVEVHTAQTQ
THREDYNSTLRVVSALPIQHQDWMSGKEFKCKVNNRALPSPIEKTISKPRGPVRAPQVYVLPPPAEEMTKKEFSLTCMITGFLPAEIAVDWTSNGRTEQNY
KNTATVLDSDGSYFMYSKLRVQKSTWERGSLFACSVVHEGLHNHLTTKTISRSLGK**HHHHHH**SSGVDLGT**ENLYFQ**ASELRCQCLKTLPRVDFKNIQSLSV
TPPGPHCAQTEVIATLKGGQKVCLDPEAPLVQKIIQKILNKGKAN*GG***GLNDIFEAQKIEWHE**--

LS-Fc-His$_6$-linker-TEV-mCXCL5$^{48\text{-}118}$-G$_2$-AviTag

MRVPAQLLGLLLLWLPGARCEPRVPITQNPCPPLKECPPCAAPDLLGGPSVFIFPPKIKDVLMISLSPMVTCVVVDVSEDDPDVQISWFVNNVEVHTAQTQ
THREDYNSTLRVVSALPIQHQDWMSGKEFKCKVNNRALPSPIEKTISKPRGPVRAPQVYVLPPPAEEMTKKEFSLTCMITGFLPAEIAVDWTSNGRTEQNY
KNTATVLDSDGSYFMYSKLRVQKSTWERGSLFACSVVHEGLHNHLTTKTISRSLGK**HHHHHH**SSGVDLGT**ENLYFQ**ATELRCVCLTVTPKINPKLIANLEV
IPAGPQCPTVEVIAKLKNQKEVCLDPEAPVIKKIIQKILGSDKKKA*GG***GLNDIFEAQKIEWHE**--
