# Supplementary material for: Directed evolution of broadly crossreactive chemokine-blocking antibodies efficacious in arthritis
Source: Nat Commun. 2018 Apr 13;9:1461. doi: 10.1038/s41467-018-03687-x (PMC5899157; doi:10.1038/s41467-018-03687-x)
Supplement: Supplementary file 5 — Supplementary Data 2 [file 41467_2018_3687_MOESM5_ESM.pdf]

Supplementary Data 2 - Protein accession numbers, oligonucleotide primers, DNA and amino-acid sequences of ^{N}CXCL-SA^{C} fusion proteins

CXCL protein (residues / accession No.)	Construct for expression	Fusion protein
hCXCL1/GROα (35–107 / P09341)	gWiz-LS-hCXCL1$^{35\text{-}107}$-(Gly_4Ser)$_2$-mouse SA-(Gly_4Ser)-His_6	^{N}hCXCL1-SA^{C}
hCXCL2/GROβ (35–107 / P19875)	gWiz-LS-hCXCL2$^{35\text{-}107}$-(Gly_4Ser)$_2$-mouse SA-(Gly_4Ser)-His_6	^{N}hCXCL2-SA^{C}
hCXCL3/GROγ (35–107 / P19876)	gWiz-LS-hCXCL3$^{35\text{-}107}$-(Gly_4Ser)$_2$-mouse SA-(Gly_4Ser)-His_6	^{N}hCXCL3-SA^{C}
hCXCL4/PF4 (32–101 / P02776)	gWiz-LS-hCXCL4$^{32\text{-}101}$-(Gly_4Ser)$_2$-mouse SA-(Gly_4Ser)-His_6	^{N}hCXCL4-SA^{C}
hCXCL5/ENA-78 (44–114 / P42830)	gWiz-LS-hCXCL5$^{44\text{-}114}$-(Gly_4Ser)$_2$-mouse SA-(Gly_4Ser)-His_6	^{N}hCXCL5-SA^{C}
hCXCL6/GCP-2 (43–114 / P80162)	gWiz-LS-hCXCL6$^{43\text{-}114}$-(Gly_4Ser)$_2$-mouse SA-(Gly_4Ser)-His_6	^{N}hCXCL6-SA^{C}
hCXCL7/NAP-2 (59–121 / P02775)	gWiz-LS-hCXCL7$^{59\text{-}121}$-(Gly_4Ser)$_2$-mouse SA-(Gly_4Ser)-His_6	^{N}hCXCL7-SA^{C}
hCXCL8/IL-8 (28–99 / P10145)	gWiz-LS-hCXCL8$^{28\text{-}99}$-(Gly_4Ser)$_2$-mouse SA-(Gly_4Ser)-His_6	^{N}hCXCL8-SA^{C}
hCXCL9/MIG (23–125 / Q07325)	gWiz-LS-hCXCL9$^{23\text{-}125}$-(Gly_4Ser)$_2$-mouse SA-(Gly_4Ser)-His_6	^{N}hCXCL9-SA^{C}
hCXCL10/IP-10 (22–98 / P02778)	gWiz-LS-hCXCL10$^{22\text{-}98}$-(Gly_4Ser)$_2$-mouse SA-(Gly_4Ser)-His_6	^{N}hCXCL10-SA^{C}
hCXCL11/I-TAC (22–94 / O14625)	gWiz-LS-hCXCL11$^{22\text{-}94}$-(Gly_4Ser)$_2$-mouse SA-(Gly_4Ser)-His_6	^{N}hCXCL11-SA^{C}
mCXCL1/KC (25–96 / P12850)	gWiz-LS-mCXCL1$^{25\text{-}96}$-(Gly_4Ser)$_2$-mouse SA-(Gly_4Ser)-His_6	^{N}mCXCL1-SA^{C}
mCXCL2/MIP-2 (28–100 / P10889)	gWiz-LS-mCXCL2$^{28\text{-}100}$-(Gly_4Ser)$_2$-mouse SA-(Gly_4Ser)-His_6	^{N}mCXCL2-SA^{C}
mCXCL3/DCIP-1 (28–100 / Q6W5C0)	gWiz-LS-mCXCL3$^{28\text{-}100}$-(Gly_4Ser)$_2$-mouse SA-(Gly_4Ser)-His_6	^{N}mCXCL3-SA^{C}

mCXCL4/PF4 (30–105 / Q9Z126)	gWiz-LS-mCXCL4$^{30-105}$-(Gly$_4$Ser)$_2$-mouse SA-(Gly$_4$Ser)-His$_6$	^{N}mCXCL4-SA^{C}
mCXCL5/LIX (48–118 / P50228)	gWiz-LS-mCXCL5$^{48-118}$-(Gly$_4$Ser)$_2$-mouse SA-(Gly$_4$Ser)-His$_6$	^{N}mCXCL5-SA^{C}
mCXCL7/NAP-2 (48–113 / Q9EQI5)	gWiz-LS-mCXCL7$^{48-113}$-(Gly$_4$Ser)$_2$-mouse SA-(Gly$_4$Ser)-His$_6$	^{N}mCXCL7-SA^{C}
mCXCL9/MIG (22–126 / P18340)	gWiz-LS-mCXCL9$^{22-126}$-(Gly$_4$Ser)$_2$-mouse SA-(Gly$_4$Ser)-His$_6$	^{N}mCXCL9-SA^{C}
mCXCL10/IP-10 (22–98 / P17515)	gWiz-LS-mCXCL10$^{22-98}$-(Gly$_4$Ser)$_2$-mouse SA-(Gly$_4$Ser)-His$_6$	^{N}mCXCL10-SA^{C}
ImCXCL11/I-TAC (22–100 / Q9JHH5)	gWiz-LS-mCXCL11$^{22-100}$-(Gly$_4$Ser)$_2$-mouse SA-(Gly$_4$Ser)-His$_6$	^{N}mCXCL11-SA^{C}

**Primer name**	**Primer sequence (5' to 3')**
Forward-linker-SA-linker-His$_6$-gWiz	5′-GGTGGAGGCGGTAGCGGAGG-3′
Reverse-LS-gWiz	5′-ACATCGTGCACCTGGGAGC-3′
Forward-hCXCL1-SA	5′-CCAGGTGCACGATGTGCCTCTGTCGCCACCGAGCTGAGATGCC-3′
Reverse-hCXCL1-SA	5′-GCTACCGCCTCCACCGTTGCTCTTGTCGCTGTTCAGC-3′
Forward-hCXCL2-SA	5′-CCAGGTGCACGATGTGCTCCTCTGGCCACAGAGC-3′
Reverse-hCXCL2-SA	5′-GCTACCGCCTCCACCGTTGCTCTTGCCGTTCTTCAGC-3′
Forward-hCXCL3-SA	5′-CCAGGTGCACGATGTGCCTCTGTCGTGACCGAGC-3′
Reverse-hCXCL3-SA	5′-GCTACCGCCTCCACCGTTGGTGCTGCCCTTGTTCAGG-3′
Forward-hCXCL4-SA	5′-CCAGGTGCACGATGTGAGGCTGAAGAGGACGGCG-3′
Reverse-hCXCL4-SA	5′-GCTACCGCCTCCACCGCTTTCCAGCAGCTTCTTG-3′

Forward-hCXCL5-SA	5′-CCAGGTGCACGATGTCTGCGCGAGCTGAGATGCG-3′
Reverse-hCXCL5-SA	5′-GCTACCGCCTCCACCGTTCTCTTTGTTGCCGCCG-3′
Forward-hCXCL6-SA	5′-CCAGGTGCACGATGTGTGCTGACCGAGCTGCGG-3′
Reverse-hCXCL6-SA	5′-GCTACCGCCTCCACCGTTCTTCTTGTTGCCGCTGTCC-3′
Forward-hCXCL7-SA	5′-CCAGGTGCACGATGTGCCGAGCTGCGGTGCATGTGC-3′
Reverse-hCXCL7-SA	5′-GCTACCGCCTCCACCCAGCTTTTTCTGCACGATTTTC-3′
Forward-hCXCL8-SA	5′-CCAGGTGCACGATGTAGCGCCAAAGAACTGCGGTGCC-3′
Reverse-hCXCL8-SA	5′-GCTACCGCCTCCACCGCTGTTCTCGGCCCGCTTCAGG-3′
Forward-hCXCL9-SA	5′-CCAGGTGCACGATGTACCCCCGTCGTGCGGAAGG-3′
Reverse-hCXCL9-SA	5′-GCTACCGCCTCCACCTGTGGTTTTCTTCTGCCGGC-3′
Forward-hCXCL10-SA	5′-CCAGGTGCACGATGTGTGCCTCTGAGCAGAACCG-3′
Reverse-hCXCL10-SA	5′-GCTACCGCCTCCACCTGGGCTCCGCTTGCTCCGC-3′
Forward-hCXCL11-SA	5′-CCAGGTGCACGATGTTTCCCCATGTTCAAGCGGG-3′
Reverse-hCXCL11-SA	5′-GCTACCGCCTCCACCGAAGTTCTTCCGTTCCACC-3′
Forward-mCXCL1-SA	5′-CCAGGTGCACGATGTGCCCCTATTGCCAACGAGCTGCGGTGCC-3′
Reverse-mCXCL1-SA	5′-GCTACCGCCTCCACCCTTGGGCACGCCCTTCAGC-3′
Forward-mCXCL2-SA	5′-CCAGGTGCACGATGTGCCGTCGTGGCCAGCGAGCTGCGG-3′
Reverse-mCXCL2-SA	5′-GCTACCGCCTCCACCGTTGGCCTTGCCCTTGTTCAGG-3′

Forward-mCXCL3-SA	5′-CCAGGTGCACGATGTGCTGTGGTGGCCTCTGAGC-3′
Reverse-mCXCL3-SA	5′-GCTACCGCCTCCACCGCTGCTCTTGCCGGACTTCAGG-3′
Forward-mCXCL4-SA	5′-CCAGGTGCACGATGTGTGACATCTGCCGGCCCTGAGG-3′
Reverse-mCXCL4-SA	5′-GCTACCGCCTCCACCGCTTTCCAGGATCTTCTTAATC-3′
Forward-mCXCL5-SA	5′-CCAGGTGCACGATGTGCCACCGAGCTGAGATGCG-3′
Reverse-mCXCL5-SA	5′-GCTACCGCCTCCACCGGCTTTCTTCTTGTCGCTGC-3′
Forward-mCXCL7-SA	5′-CCAGGTGCACGATGTATCGAGCTGCGGTGCCGGTGC-3′
Reverse-mCXCL7-SA	5′-GCTACCGCCTCCACCGTAGCCTTCCAGAATCTTCATC-3′
Forward-mCXCL9-SA	5′-CCAGGTGCACGATGTACCCTCGTGATCCGGAACGC-3′
Reverse-mCXCL9-SA	5′-GCTACCGCCTCCACCTGTGGTCTTTCTGGATCTCC-3′
Forward-mCXCL10-SA	5′-CCAGGTGCACGATGTATCCCACTGGCCAGAACCG-3′
Reverse-mCXCL10-SA	5′-GCTACCGCCTCCACCTGGGGCCCTCTTGCTCCGC-3′
Forward-mCXCL11-SA	5′-CCAGGTGCACGATGTTTCCTGATGTTCAAGCAGGG-3′
Reverse-mCXCL11-SA	5′-GCTACCGCCTCCACCCATGTTCTGCCGCCGCAGG-3′

Sequences of genes coding for CXCL chemokines fused to the N-terminus of mouse serum albumin (^{N}CXCL-SA^{C})

DNA sequences of genes are provided. Start and stop codons are shown in black bold. The sequences include the secretory leader peptide (LS, in grey underlined), an active form of CXCL (in blue bold), a decapeptide $(Gly_4Ser)_2$ flexible linker (in green bold), mouse serum albumin (SA, in grey), a pentapeptide (Gly_4Ser) flexible linker (in black italic and underlined) and the hexa-histidine tag (His_6, in red bold).

gWiz-LS-hCXCL1$^{35\text{-}107}$-$(Gly_4Ser)_2$-mouse SA-(Gly_4Ser)-His_6

ATGAGGGTCCCCGCTCAGCTCCTGGGGCTCCTGCTGCTCTGGCTCCCAGGTGCACGATGT**GCCTCTGTCGCCACCGAGCTGAGATGCCAGTGCCTGCAGAC**
CCTGCAGGGCATCCACCCCAAGAACATCCAGAGCGTGAACGTGAAGTCCCCTGGCCCCCACTGCGCCCAGACCGAAGTGATCGCCACCCTGAAGAACGGCC
GGAAGGCCTGCCTGAACCCCGCCAGCCCCATCGTGAAGAAAATCATCGAGAAGATGCTGAACAGCGACAAGAGCAACGGTGGAGGCGGTAGCGGAGGCGGA
GGGTCGGAAGCACACAAGAGTGAGATCGCCCATCGGTATAATGATTTGGGAGAACAACATTTCAAAGGCCTAGTCCTGATTGCCTTTTCCCAGTATCTCCA
GAAATGCTCATACGATGAGCATGCCAAATTAGTGCAGGAAGTAACAGACTTTGCAAAGACGTGTGTTGCCGATGAGTCTGCCGCCAACTGTGACAAATCCC
TTCACACTCTTTTTGGAGATAAGTTGTGTGCCATTCCAAACCTCCGTGAAAACTATGGTGAACTGGCTGACTGCTGTACAAAACAAGAGCCCGAAAGAAAC
GAATGTTTCCTGCAACACAAAGATGACAACCCCAGCCTGCCACCATTTGAAAGGCCAGAGGCTGAGGCCATGTGCACCTCCTTTAAGGAAAACCCAACCAC
CTTTATGGGACACTATTTGCATGAAGTTGCCAGAAGACATCCTTATTTCTATGCCCCAGAACTTCTTTACTATGCTGAGCAGTACAATGAGATTCTGACCC
AGTGTTGTGCAGAGGCTGACAAGGAAAGCTGCCTGACCCCGAAGCTTGATGGTGTGAAGGAGAAAGCATTGGTCTCATCTGTCCGTCAGAGAATGAAGTGC
TCCAGTATGCAGAAGTTTGGAGAGAGAGCTTTTAAAGCATGGGCAGTAGCTCGTCTGAGCCAGACATTCCCCAATGCTGACTTTGCAGAAATCACCAAATT
GGCAACAGACCTGACCAAAGTCAACAAGGAGTGCTGCCATGGTGACCTGCTGGAATGCGCAGATGACAGGGCGGAACTTGCCAAGTACATGTGTGAAAACC
AGGCGACTATCTCCAGCAAACTGCAGACTTGCTGCGATAAACCACTGTTGAAGAAAGCCCACTGTCTTAGTGAGGTGGAGCATGACACCATGCCTGCTGAT
CTGCCTGCCATTGCTGCTGATTTTGTTGAGGACCAGGAAGTGTGCAAGAACTATGCTGAGGCCAAGGATGTCTTCCTGGGCACGTTCTTGTATGAATATTC
AAGAAGACACCCTGATTACTCTGTATCCCTGTTGCTGAGACTTGCTAAGAAATATGAAGCCACTCTGGAAAAGTGCTGCGCTGAAGCCAATCCTCCCGCAT
GCTACGGCACAGTGCTTGCTGAATTTCAGCCTCTTGTAGAAGAGCCTAAGAACTTGGTCAAAACCAACTGTGATCTTTACGAGAAGCTTGGAGAATATGGA
TTCCAAAATGCCATTCTAGTTCGCTACACCCAGAAAGCACCTCAGGTGTCAACCCCAACTCTCGTGGAGGCTGCAAGAAACCTAGGAAGAGTGGGCACCAA
GTGTTGTACACTTCCTGAAGATCAGAGACTGCCTTGTGTGGAAGACTATCTGTCTGCAATCCTGAACCGTGTGTGTCTGCTGCATGAGAAGACCCCAGTGA
GTGAGCATGTTACCAAGTGCTGTAGTGGATCCCTGGTGGAAAGGCGGCCATGCTTCTCTGCTCTGACAGTTGATGAAACATATGTCCCCAAAGAGTTTAAA
GCTGAGACCTTCACCTTCCACTCTGATATCTGCACACTTCCAGAGAAGGAGAAGCAGATTAAGAAACAAACGGCTCTTGCTGAGCTGGTGAAGCACAAGCC
CAAGGCTACAGCGGAGCAACTGAAGACTGTCATGGATGACTTTGCACAGTTCCTGGATACATGTTGCAAGGCTGCTGACAAGGACACCTGCTTCTCGACTG
AGGGTCCAAACCTTGTCACTAGATGCAAAGACGCCTTAGCC*GGAGGGGGCGGTTCC***CACCATCACCACCATCACTGATAA**

gWiz-LS-hCXCL2$^{35\text{-}107}$*-(Gly*$_4$*Ser)*$_2$*-mouse SA-(Gly*$_4$*Ser)-His*$_6$

ATGAGGGTCCCCGCTCAGCTCCTGGGGCTCCTGCTGCTCTGGCTCCCAGGTGCACGATGT**GCTCCTCTGGCCACAGAGCTGAGATGCCAGTGCCTCCAGAC**
ACTCCAGGGCATCCACCTGAAGAACATCCAGAGCGTGAAAGTGAAGTCCCCTGGCCCCCACTGCGCCCAGACAGAAGTGATCGCCACCCTGAAGAATGGCC
AGAAGGCCTGCCTGAACCCCGCCAGCCCTATGGTCAAGAAAATCATCGAGAAGATGCTGAAGAACGGCAAGAGCAACGGTGGAGGCGGTAGCGGAGGCGGA
GGGTCGGAAGCACACAAGAGTGAGATCGCCCATCGGTATAATGATTTGGGAGAACAACATTTCAAAGGCCTAGTCCTGATTGCCTTTTCCCAGTATCTCCA
GAAATGCTCATACGATGAGCATGCCAAATTAGTGCAGGAAGTAACAGACTTTGCAAAGACGTGTGTTGCCGATGAGTCTGCCGCCAACTGTGACAAATCCC
TTCACACTCTTTTTGGAGATAAGTTGTGTGCCATTCCAAACCTCCGTGAAAACTATGGTGAACTGGCTGACTGCTGTACAAAACAAGAGCCCGAAAGAAAC
GAATGTTTCCTGCAACACAAAGATGACAACCCCAGCCTGCCACCATTTGAAAGGCCAGAGGCTGAGGCCATGTGCACCTCCTTTAAGGAAAACCCAACCAC
CTTTATGGGACACTATTTGCATGAAGTTGCCAGAAGACATCCTTATTTCTATGCCCCAGAACTTCTTTACTATGCTGAGCAGTACAATGAGATTCTGACCC
AGTGTTGTGCAGAGGCTGACAAGGAAAGCTGCCTGACCCCGAAGCTTGATGGTGTGAAGGAGAAAGCATTGGTCTCATCTGTCCGTCAGAGAATGAAGTGC
TCCAGTATGCAGAAGTTTGGAGAGAGAGCTTTTAAAGCATGGGCAGTAGCTCGTCTGAGCCAGACATTCCCCAATGCTGACTTTGCAGAAATCACCAAATT
GGCAACAGACCTGACCAAAGTCAACAAGGAGTGCTGCCATGGTGACCTGCTGGAATGCGCAGATGACAGGGCGGAACTTGCCAAGTACATGTGTGAAAACC
AGGCGACTATCTCCAGCAAACTGCAGACTTGCTGCGATAAACCACTGTTGAAGAAAGCCCACTGTCTTAGTGAGGTGGAGCATGACACCATGCCTGCTGAT
CTGCCTGCCATTGCTGCTGATTTTGTTGAGGACCAGGAAGTGTGCAAGAACTATGCTGAGGCCAAGGATGTCTTCCTGGGCACGTTCTTGTATGAATATTC
AAGAAGACACCCTGATTACTCTGTATCCCTGTTGCTGAGACTTGCTAAGAAATATGAAGCCACTCTGGAAAAGTGCTGCGCTGAAGCCAATCCTCCCGCAT
GCTACGGCACAGTGCTTGCTGAATTTCAGCCTCTTGTAGAAGAGCCTAAGAACTTGGTCAAAACCAACTGTGATCTTTACGAGAAGCTTGGAGAATATGGA
TTCCAAAATGCCATTCTAGTTCGCTACACCCAGAAAGCACCTCAGGTGTCAACCCCAACTCTCGTGGAGGCTGCAAGAAACCTAGGAAGAGTGGGCACCAA
GTGTTGTACACTTCCTGAAGATCAGAGACTGCCTTGTGTGGAAGACTATCTGTCTGCAATCCTGAACCGTGTGTGTCTGCTGCATGAGAAGACCCCAGTGA
GTGAGCATGTTACCAAGTGCTGTAGTGGATCCCTGGTGGAAAGGCGGCCATGCTTCTCTGCTCTGACAGTTGATGAAACATATGTCCCCAAAGAGTTTAAA
GCTGAGACCTTCACCTTCCACTCTGATATCTGCACACTTCCAGAGAAGGAGAAGCAGATTAAGAAACAAACGGCTCTTGCTGAGCTGGTGAAGCACAAGCC
CAAGGCTACAGCGGAGCAACTGAAGACTGTCATGGATGACTTTGCACAGTTCCTGGATACATGTTGCAAGGCTGCTGACAAGGACACCTGCTTCTCGACTG
AGGGTCCAAACCTTGTCACTAGATGCAAAGACGCCTTAGCC*GGAGGGGGCGGTTCC*CACCATCACCACCATCAC**TGATAA**

gWiz-LS-hCXCL3$^{35\text{-}107}$*-(Gly*$_4$*Ser)*$_2$*-mouse SA-(Gly*$_4$*Ser)-His*$_6$

ATGAGGGTCCCCGCTCAGCTCCTGGGGCTCCTGCTGCTCTGGCTCCCAGGTGCACGATGT**GCCTCTGTCGTGACCGAGCTGAGATGCCAGTGCCTCCAGAC**
ACTCCAGGGCATCCACCTGAAGAACATCCAGAGCGTGAACGTGCGGAGCCCTGGCCCTCATTGTGCCCAGACAGAAGTGATCGCCACCCTGAAGAATGGCA
AGAAGGCCTGCCTGAACCCCGCCAGCCCTATGGTGCAGAAGATCATCGAGAAGATCCTGAACAAGGGCAGCACCAACGGTGGAGGCGGTAGCGGAGGCGGA
GGGTCGGAAGCACACAAGAGTGAGATCGCCCATCGGTATAATGATTTGGGAGAACAACATTTCAAAGGCCTAGTCCTGATTGCCTTTTCCCAGTATCTCCA
GAAATGCTCATACGATGAGCATGCCAAATTAGTGCAGGAAGTAACAGACTTTGCAAAGACGTGTGTTGCCGATGAGTCTGCCGCCAACTGTGACAAATCCC
TTCACACTCTTTTTGGAGATAAGTTGTGTGCCATTCCAAACCTCCGTGAAAACTATGGTGAACTGGCTGACTGCTGTACAAAACAAGAGCCCGAAAGAAAC
GAATGTTTCCTGCAACACAAAGATGACAACCCCAGCCTGCCACCATTTGAAAGGCCAGAGGCTGAGGCCATGTGCACCTCCTTTAAGGAAAACCCAACCAC
CTTTATGGGACACTATTTGCATGAAGTTGCCAGAAGACATCCTTATTTCTATGCCCCAGAACTTCTTTACTATGCTGAGCAGTACAATGAGATTCTGACCC
AGTGTTGTGCAGAGGCTGACAAGGAAAGCTGCCTGACCCCGAAGCTTGATGGTGTGAAGGAGAAAGCATTGGTCTCATCTGTCCGTCAGAGAATGAAGTGC
TCCAGTATGCAGAAGTTTGGAGAGAGAGCTTTTAAAGCATGGGCAGTAGCTCGTCTGAGCCAGACATTCCCCAATGCTGACTTTGCAGAAATCACCAAATT

GGCAACAGACCTGACCAAAGTCAACAAGGAGTGCTGCCATGGTGACCTGCTGGAATGCGCAGATGACAGGGCGGAACTTGCCAAGTACATGTGTGAAAACC
AGGCGACTATCTCCAGCAAACTGCAGACTTGCTGCGATAAACCACTGTTGAAGAAAGCCCACTGTCTTAGTGAGGTGGAGCATGACACCATGCCTGCTGAT
CTGCCTGCCATTGCTGCTGATTTTGTTGAGGACCAGGAAGTGTGCAAGAACTATGCTGAGGCCAAGGATGTCTTCCTGGGCACGTTCTTGTATGAATATTC
AAGAAGACACCCTGATTACTCTGTATCCCTGTTGCTGAGACTTGCTAAGAAATATGAAGCCACTCTGGAAAAGTGCTGCGCTGAAGCCAATCCTCCCGCAT
GCTACGGCACAGTGCTTGCTGAATTTCAGCCTCTTGTAGAAGAGCCTAAGAACTTGGTCAAAACCAACTGTGATCTTTACGAGAAGCTTGGAGAATATGGA
TTCCAAAATGCCATTCTAGTTCGCTACACCCAGAAAGCACCTCAGGTGTCAACCCCAACTCTCGTGGAGGCTGCAAGAAACCTAGGAAGAGTGGGCACCAA
GTGTTGTACACTTCCTGAAGATCAGAGACTGCCTTGTGTGGAAGACTATCTGTCTGCAATCCTGAACCGTGTGTGTCTGCTGCATGAGAAGACCCCAGTGA
GTGAGCATGTTACCAAGTGCTGTAGTGGATCCCTGGTGGAAAGGCGGCCATGCTTCTCTGCTCTGACAGTTGATGAAACATATGTCCCCAAAGAGTTTAAA
GCTGAGACCTTCACCTTCCACTCTGATATCTGCACACTTCCAGAGAAGGAGAAGCAGATTAAGAAACAAACGGCTCTTGCTGAGCTGGTGAAGCACAAGCC
CAAGGCTACAGCGGAGCAACTGAAGACTGTCATGGATGACTTTGCACAGTTCCTGGATACATGTTGCAAGGCTGCTGACAAGGACACCTGCTTCTCGACTG
AGGGTCCAAACCTTGTCACTAGATGCAAAGACGCCTTAGCC*GGAGGGGGCGGTTCC***CACCATCACCACCATCACTGATAA**

gWiz-LS-hCXCL4$^{32-101}$-(Gly$_4$Ser)$_2$-mouse SA-(Gly$_4$Ser)-His$_6$

ATGAGGGTCCCCGCTCAGCTCCTGGGGCTCCTGCTGCTCTGGCTCCCAGGTGCACGATGT**GAGGCTGAAGAGGACGGCGATCTCCAGTGCCTGTGCGTGAA**
AACCACCAGCCAAGTGCGGCCCAGACACATCACCAGCCTGGAAGTGATCAAGGCCGGACCCCACTGTCCTACCGCCCAGCTGATTGCCACCCTGAAGAACG
GCCGGAAGATCTGCCTGGACCTCCAGGCCCCCCTGTACAAGAAGATCATCAAGAAGCTGCTGGAAAGCGGTGGAGGCGGTAGCGGAGGCGGAGGGTCGGAA
GCACACAAGAGTGAGATCGCCCATCGGTATAATGATTTGGGAGAACAACATTTCAAAGGCCTAGTCCTGATTGCCTTTTCCCAGTATCTCCAGAAATGCTC
ATACGATGAGCATGCCAAATTAGTGCAGGAAGTAACAGACTTTGCAAAGACGTGTGTTGCCGATGAGTCTGCCGCCAACTGTGACAAATCCCTTCACACTC
TTTTTGGAGATAAGTTGTGTGCCATTCCAAACCTCCGTGAAAACTATGGTGAACTGGCTGACTGCTGTACAAAACAAGAGCCCGAAAGAAACGAATGTTTC
CTGCAACACAAAGATGACAACCCCAGCCTGCCACCATTTGAAAGGCCAGAGGCTGAGGCCATGTGCACCTCCTTTAAGGAAAACCCAACCACCTTTATGGG
ACACTATTTGCATGAAGTTGCCAGAAGACATCCTTATTTCTATGCCCCAGAACTTCTTTACTATGCTGAGCAGTACAATGAGATTCTGACCCAGTGTTGTG
CAGAGGCTGACAAGGAAAGCTGCCTGACCCCGAAGCTTGATGGTGTGAAGGAGAAAGCATTGGTCTCATCTGTCCGTCAGAGAATGAAGTGCTCCAGTATG
CAGAAGTTTGGAGAGAGAGCTTTTAAAGCATGGGCAGTAGCTCGTCTGAGCCAGACATTCCCCAATGCTGACTTTGCAGAAATCACCAAATTGGCAACAGA
CCTGACCAAAGTCAACAAGGAGTGCTGCCATGGTGACCTGCTGGAATGCGCAGATGACAGGGCGGAACTTGCCAAGTACATGTGTGAAAACCAGGCGACTA
TCTCCAGCAAACTGCAGACTTGCTGCGATAAACCACTGTTGAAGAAAGCCCACTGTCTTAGTGAGGTGGAGCATGACACCATGCCTGCTGATCTGCCTGCC
ATTGCTGCTGATTTTGTTGAGGACCAGGAAGTGTGCAAGAACTATGCTGAGGCCAAGGATGTCTTCCTGGGCACGTTCTTGTATGAATATTCAAGAAGACA
CCCTGATTACTCTGTATCCCTGTTGCTGAGACTTGCTAAGAAATATGAAGCCACTCTGGAAAAGTGCTGCGCTGAAGCCAATCCTCCCGCATGCTACGGCA
CAGTGCTTGCTGAATTTCAGCCTCTTGTAGAAGAGCCTAAGAACTTGGTCAAAACCAACTGTGATCTTTACGAGAAGCTTGGAGAATATGGATTCCAAAAT
GCCATTCTAGTTCGCTACACCCAGAAAGCACCTCAGGTGTCAACCCCAACTCTCGTGGAGGCTGCAAGAAACCTAGGAAGAGTGGGCACCAAGTGTTGTAC
ACTTCCTGAAGATCAGAGACTGCCTTGTGTGGAAGACTATCTGTCTGCAATCCTGAACCGTGTGTGTCTGCTGCATGAGAAGACCCCAGTGAGTGAGCATG
TTACCAAGTGCTGTAGTGGATCCCTGGTGGAAAGGCGGCCATGCTTCTCTGCTCTGACAGTTGATGAAACATATGTCCCCAAAGAGTTTAAAGCTGAGACC
TTCACCTTCCACTCTGATATCTGCACACTTCCAGAGAAGGAGAAGCAGATTAAGAAACAAACGGCTCTTGCTGAGCTGGTGAAGCACAAGCCCAAGGCTAC
AGCGGAGCAACTGAAGACTGTCATGGATGACTTTGCACAGTTCCTGGATACATGTTGCAAGGCTGCTGACAAGGACACCTGCTTCTCGACTGAGGGTCCAA
ACCTTGTCACTAGATGCAAAGACGCCTTAGCC*GGAGGGGGCGGTTCC***CACCATCACCACCATCACTGATAA**

gWiz-LS-hCXCL5$^{44\text{-}114}$-(Gly$_4$Ser)$_2$-mouse SA-(Gly$_4$Ser)-His$_6$

ATGAGGGTCCCCGCTCAGCTCCTGGGGCTCCTGCTGCTCTGGCTCCCAGGTGCACGATGT**CTGCGCGAGCTGAGATGCGTGTGCCTGCAGACCACCCAGGG**
CGTGCACCCCAAGATGATCAGCAACCTCCAGGTGTTCGCCATCGGCCCCCAGTGCAGCAAGGTGGAAGTGGTGGCCAGCCTGAAGAACGGCAAAGAGATCT
GCCTGGACCCCGAGGCCCCATTCCTGAAGAAAGTGATCCAGAAGATCCTGGACGGCGGCAACAAAGAGAACGGTGGAGGCGGTAGCGGAGGCGGAGGGTCG
GAAGCACACAAGAGTGAGATCGCCCATCGGTATAATGATTTGGGAGAACAACATTTCAAAGGCCTAGTCCTGATTGCCTTTTCCCAGTATCTCCAGAAATG
CTCATACGATGAGCATGCCAAATTAGTGCAGGAAGTAACAGACTTTGCAAAGACGTGTGTTGCCGATGAGTCTGCCGCCAACTGTGACAAATCCCTTCACA
CTCTTTTTGGAGATAAGTTGTGTGCCATTCCAAACCTCCGTGAAAACTATGGTGAACTGGCTGACTGCTGTACAAAACAAGAGCCCGAAAGAAACGAATGT
TTCCTGCAACACAAAGATGACAACCCCAGCCTGCCACCATTTGAAAGGCCAGAGGCTGAGGCCATGTGCACCTCCTTTAAGGAAAACCCAACCACCTTTAT
GGGACACTATTTGCATGAAGTTGCCAGAAGACATCCTTATTTCTATGCCCCAGAACTTCTTTACTATGCTGAGCAGTACAATGAGATTCTGACCCAGTGTT
GTGCAGAGGCTGACAAGGAAAGCTGCCTGACCCCGAAGCTTGATGGTGTGAAGGAGAAAGCATTGGTCTCATCTGTCCGTCAGAGAATGAAGTGCTCCAGT
ATGCAGAAGTTTGGAGAGAGAGCTTTTAAAGCATGGGCAGTAGCTCGTCTGAGCCAGACATTCCCCAATGCTGACTTTGCAGAAATCACCAAATTGGCAAC
AGACCTGACCAAAGTCAACAAGGAGTGCTGCCATGGTGACCTGCTGGAATGCGCAGATGACAGGGCGGAACTTGCCAAGTACATGTGTGAAAACCAGGCGA
CTATCTCCAGCAAACTGCAGACTTGCTGCGATAAACCACTGTTGAAGAAAGCCCACTGTCTTAGTGAGGTGGAGCATGACACCATGCCTGCTGATCTGCCT
GCCATTGCTGCTGATTTTGTTGAGGACCAGGAAGTGTGCAAGAACTATGCTGAGGCCAAGGATGTCTTCCTGGGCACGTTCTTGTATGAATATTCAAGAAG
ACACCCTGATTACTCTGTATCCCTGTTGCTGAGACTTGCTAAGAAATATGAAGCCACTCTGGAAAAGTGCTGCGCTGAAGCCAATCCTCCCGCATGCTACG
GCACAGTGCTTGCTGAATTTCAGCCTCTTGTAGAAGAGCCTAAGAACTTGGTCAAAACCAACTGTGATCTTTACGAGAAGCTTGGAGAATATGGATTCCAA
AATGCCATTCTAGTTCGCTACACCCAGAAAGCACCTCAGGTGTCAACCCCAACTCTCGTGGAGGCTGCAAGAAACCTAGGAAGAGTGGGCACCAAGTGTTG
TACACTTCCTGAAGATCAGAGACTGCCTTGTGTGGAAGACTATCTGTCTGCAATCCTGAACCGTGTGTGTCTGCTGCATGAGAAGACCCCAGTGAGTGAGC
ATGTTACCAAGTGCTGTAGTGGATCCCTGGTGGAAAGGCGGCCATGCTTCTCTGCTCTGACAGTTGATGAAACATATGTCCCCAAAGAGTTTAAAGCTGAG
ACCTTCACCTTCCACTCTGATATCTGCACACTTCCAGAGAAGGAGAAGCAGATTAAGAAACAAACGGCTCTTGCTGAGCTGGTGAAGCACAAGCCCAAGGC
TACAGCGGAGCAACTGAAGACTGTCATGGATGACTTTGCACAGTTCCTGGATACATGTTGCAAGGCTGCTGACAAGGACACCTGCTTCTCGACTGAGGGTC
CAAACCTTGTCACTAGATGCAAAGACGCCTTAGCC*GGAGGGGGCGGTTCC***CACCATCACCACCATCACTGATAA**

gWiz-LS-hCXCL6$^{43\text{-}114}$-(Gly$_4$Ser)$_2$-mouse SA-(Gly$_4$Ser)-His$_6$

ATGAGGGTCCCCGCTCAGCTCCTGGGGCTCCTGCTGCTCTGGCTCCCAGGTGCACGATGT**GTGCTGACCGAGCTGCGGTGCACCTGTCTGAGAGTGACCCT**
GCGCGTGAACCCCAAGACCATCGGCAAGCTCCAGGTGTTCCCTGCCGGCCCTCAGTGCAGCAAGGTGGAAGTGGTGGCCAGCCTGAAAAACGGAAAACAAG
TGTGCCTGGACCCCGAGGCCCCATTCCTGAAGAAAGTGATCCAGAAGATCCTGGACAGCGGCAACAAGAAGAACGGTGGAGGCGGTAGCGGAGGCGGAGGG
TCGGAAGCACACAAGAGTGAGATCGCCCATCGGTATAATGATTTGGGAGAACAACATTTCAAAGGCCTAGTCCTGATTGCCTTTTCCCAGTATCTCCAGAA
ATGCTCATACGATGAGCATGCCAAATTAGTGCAGGAAGTAACAGACTTTGCAAAGACGTGTGTTGCCGATGAGTCTGCCGCCAACTGTGACAAATCCCTTC
ACACTCTTTTTGGAGATAAGTTGTGTGCCATTCCAAACCTCCGTGAAAACTATGGTGAACTGGCTGACTGCTGTACAAAACAAGAGCCCGAAAGAAACGAA
TGTTTCCTGCAACACAAAGATGACAACCCCAGCCTGCCACCATTTGAAAGGCCAGAGGCTGAGGCCATGTGCACCTCCTTTAAGGAAAACCCAACCACCTT
TATGGGACACTATTTGCATGAAGTTGCCAGAAGACATCCTTATTTCTATGCCCCAGAACTTCTTTACTATGCTGAGCAGTACAATGAGATTCTGACCCAGT
GTTGTGCAGAGGCTGACAAGGAAAGCTGCCTGACCCCGAAGCTTGATGGTGTGAAGGAGAAAGCATTGGTCTCATCTGTCCGTCAGAGAATGAAGTGCTCC
AGTATGCAGAAGTTTGGAGAGAGAGCTTTTAAAGCATGGGCAGTAGCTCGTCTGAGCCAGACATTCCCCAATGCTGACTTTGCAGAAATCACCAAATTGGC

AACAGACCTGACCAAAGTCAACAAGGAGTGCTGCCATGGTGACCTGCTGGAATGCGCAGATGACAGGGCGGAACTTGCCAAGTACATGTGTGAAAACCAGG
CGACTATCTCCAGCAAACTGCAGACTTGCTGCGATAAACCACTGTTGAAGAAAGCCCACTGTCTTAGTGAGGTGGAGCATGACACCATGCCTGCTGATCTG
CCTGCCATTGCTGCTGATTTTGTTGAGGACCAGGAAGTGTGCAAGAACTATGCTGAGGCCAAGGATGTCTTCCTGGGCACGTTCTTGTATGAATATTCAAG
AAGACACCCTGATTACTCTGTATCCCTGTTGCTGAGACTTGCTAAGAAATATGAAGCCACTCTGGAAAAGTGCTGCGCTGAAGCCAATCCTCCCGCATGCT
ACGGCACAGTGCTTGCTGAATTTCAGCCTCTTGTAGAAGAGCCTAAGAACTTGGTCAAAACCAACTGTGATCTTTACGAGAAGCTTGGAGAATATGGATTC
CAAAATGCCATTCTAGTTCGCTACACCCAGAAAGCACCTCAGGTGTCAACCCCAACTCTCGTGGAGGCTGCAAGAAACCTAGGAAGAGTGGGCACCAAGTG
TTGTACACTTCCTGAAGATCAGAGACTGCCTTGTGTGGAAGACTATCTGTCTGCAATCCTGAACCGTGTGTGTCTGCTGCATGAGAAGACCCCAGTGAGTG
AGCATGTTACCAAGTGCTGTAGTGGATCCCTGGTGGAAAGGCGGCCATGCTTCTCTGCTCTGACAGTTGATGAAACATATGTCCCCAAAGAGTTTAAAGCT
GAGACCTTCACCTTCCACTCTGATATCTGCACACTTCCAGAGAAGGAGAAGCAGATTAAGAAACAAACGGCTCTTGCTGAGCTGGTGAAGCACAAGCCCAA
GGCTACAGCGGAGCAACTGAAGACTGTCATGGATGACTTTGCACAGTTCCTGGATACATGTTGCAAGGCTGCTGACAAGGACACCTGCTTCTCGACTGAGG
GTCCAAACCTTGTCACTAGATGCAAAGACGCCTTAGCC*GGAGGGGGCGGTTCC***CACCATCACCACCATCACTGATAA**

gWiz-LS-hCXCL7$^{59-121}$-(Gly$_4$Ser)$_2$-mouse SA-(Gly$_4$Ser)-His$_6$

ATGAGGGTCCCCGCTCAGCTCCTGGGGCTCCTGCTGCTCTGGCTCCCAGGTGCACGATGT**GCCGAGCTGCGGTGCATGTGCATCAAGACCACCAGCGGAAT
CCACCCCAAGAATATCCAGTCCCTGGAAGTGATTGGCAAGGGCACCCACTGCAACCAGGTGGAAGTGATTGCCACACTGAAAGACGGCCGGAAGATCTGCC
TGGACCCTGACGCCCCCAGAATCAAGAAAATCGTGCAGAAAAAGCTGGGTGGAGGCGGTAGCGGAGGCGGAGGGTCG**GAAGCACACAAGAGTGAGATCGCC
CATCGGTATAATGATTTGGGAGAACAACATTTCAAAGGCCTAGTCCTGATTGCCTTTTCCCAGTATCTCCAGAAATGCTCATACGATGAGCATGCCAAATT
AGTGCAGGAAGTAACAGACTTTGCAAAGACGTGTGTTGCCGATGAGTCTGCCGCCAACTGTGACAAATCCCTTCACACTCTTTTTGGAGATAAGTTGTGTG
CCATTCCAAACCTCCGTGAAAACTATGGTGAACTGGCTGACTGCTGTACAAAACAAGAGCCCGAAAGAAACGAATGTTTCCTGCAACACAAAGATGACAAC
CCCAGCCTGCCACCATTTGAAAGGCCAGAGGCTGAGGCCATGTGCACCTCCTTTAAGGAAAACCCAACCACCTTTATGGGACACTATTTGCATGAAGTTGC
CAGAAGACATCCTTATTTCTATGCCCCAGAACTTCTTTACTATGCTGAGCAGTACAATGAGATTCTGACCCAGTGTTGTGCAGAGGCTGACAAGGAAAGCT
GCCTGACCCCGAAGCTTGATGGTGTGAAGGAGAAAGCATTGGTCTCATCTGTCCGTCAGAGAATGAAGTGCTCCAGTATGCAGAAGTTTGGAGAGAGAGCT
TTTAAAGCATGGGCAGTAGCTCGTCTGAGCCAGACATTCCCCAATGCTGACTTTGCAGAAATCACCAAATTGGCAACAGACCTGACCAAAGTCAACAAGGA
GTGCTGCCATGGTGACCTGCTGGAATGCGCAGATGACAGGGCGGAACTTGCCAAGTACATGTGTGAAAACCAGGCGACTATCTCCAGCAAACTGCAGACTT
GCTGCGATAAACCACTGTTGAAGAAAGCCCACTGTCTTAGTGAGGTGGAGCATGACACCATGCCTGCTGATCTGCCTGCCATTGCTGCTGATTTTGTTGAG
GACCAGGAAGTGTGCAAGAACTATGCTGAGGCCAAGGATGTCTTCCTGGGCACGTTCTTGTATGAATATTCAAGAAGACACCCTGATTACTCTGTATCCCT
GTTGCTGAGACTTGCTAAGAAATATGAAGCCACTCTGGAAAAGTGCTGCGCTGAAGCCAATCCTCCCGCATGCTACGGCACAGTGCTTGCTGAATTTCAGC
CTCTTGTAGAAGAGCCTAAGAACTTGGTCAAAACCAACTGTGATCTTTACGAGAAGCTTGGAGAATATGGATTCCAAAATGCCATTCTAGTTCGCTACACC
CAGAAAGCACCTCAGGTGTCAACCCCAACTCTCGTGGAGGCTGCAAGAAACCTAGGAAGAGTGGGCACCAAGTGTTGTACACTTCCTGAAGATCAGAGACT
GCCTTGTGTGGAAGACTATCTGTCTGCAATCCTGAACCGTGTGTGTCTGCTGCATGAGAAGACCCCAGTGAGTGAGCATGTTACCAAGTGCTGTAGTGGAT
CCCTGGTGGAAAGGCGGCCATGCTTCTCTGCTCTGACAGTTGATGAAACATATGTCCCCAAAGAGTTTAAAGCTGAGACCTTCACCTTCCACTCTGATATC
TGCACACTTCCAGAGAAGGAGAAGCAGATTAAGAAACAAACGGCTCTTGCTGAGCTGGTGAAGCACAAGCCCAAGGCTACAGCGGAGCAACTGAAGACTGT
CATGGATGACTTTGCACAGTTCCTGGATACATGTTGCAAGGCTGCTGACAAGGACACCTGCTTCTCGACTGAGGGTCCAAACCTTGTCACTAGATGCAAAG
ACGCCTTAGCC*GGAGGGGGCGGTTCC***CACCATCACCACCATCACTGATAA**

gWiz-LS-hCXCL8$^{28-99}$-(Gly$_4$Ser)$_2$-mouse SA-(Gly$_4$Ser)-His$_6$

ATGAGGGTCCCCGCTCAGCTCCTGGGGCTCCTGCTGCTCTGGCTCCCAGGTGCACGATGT**AGCGCCAAAGAACTGCGGTGCCAGTGCATCAAGACCTACAG**
CAAGCCCTTCCACCCCAAGTTCATCAAAGAACTGAGAGTGATCGAGAGCGGCCCTCACTGCGCCAACACCGAGATCATCGTGAAGCTGAGCGACGGCAGAG
AGCTGTGCCTGGACCCCAAAGAAAACTGGGTGCAGCGGGTGGTGGAAAAGTTCCTGAAGCGGGCCGAGAACAGCGGTGGAGGCGGTAGCGGAGGCGGAGGG
TCGGAAGCACACAAGAGTGAGATCGCCCATCGGTATAATGATTTGGGAGAACAACATTTCAAAGGCCTAGTCCTGATTGCCTTTTCCCAGTATCTCCAGAA
ATGCTCATACGATGAGCATGCCAAATTAGTGCAGGAAGTAACAGACTTTGCAAAGACGTGTGTTGCCGATGAGTCTGCCGCCAACTGTGACAAATCCCTTC
ACACTCTTTTTGGAGATAAGTTGTGTGCCATTCCAAACCTCCGTGAAAACTATGGTGAACTGGCTGACTGCTGTACAAAACAAGAGCCCGAAAGAAACGAA
TGTTTCCTGCAACACAAAGATGACAACCCCAGCCTGCCACCATTTGAAAGGCCAGAGGCTGAGGCCATGTGCACCTCCTTTAAGGAAAACCCAACCACCTT
TATGGGACACTATTTGCATGAAGTTGCCAGAAGACATCCTTATTTCTATGCCCCAGAACTTCTTTACTATGCTGAGCAGTACAATGAGATTCTGACCCAGT
GTTGTGCAGAGGCTGACAAGGAAAGCTGCCTGACCCCGAAGCTTGATGGTGTGAAGGAGAAAGCATTGGTCTCATCTGTCCGTCAGAGAATGAAGTGCTCC
AGTATGCAGAAGTTTGGAGAGAGAGCTTTTAAAGCATGGGCAGTAGCTCGTCTGAGCCAGACATTCCCCAATGCTGACTTTGCAGAAATCACCAAATTGGC
AACAGACCTGACCAAAGTCAACAAGGAGTGCTGCCATGGTGACCTGCTGGAATGCGCAGATGACAGGGCGGAACTTGCCAAGTACATGTGTGAAAACCAGG
CGACTATCTCCAGCAAACTGCAGACTTGCTGCGATAAACCACTGTTGAAGAAAGCCCACTGTCTTAGTGAGGTGGAGCATGACACCATGCCTGCTGATCTG
CCTGCCATTGCTGCTGATTTTGTTGAGGACCAGGAAGTGTGCAAGAACTATGCTGAGGCCAAGGATGTCTTCCTGGGCACGTTCTTGTATGAATATTCAAG
AAGACACCCTGATTACTCTGTATCCCTGTTGCTGAGACTTGCTAAGAAATATGAAGCCACTCTGGAAAAGTGCTGCGCTGAAGCCAATCCTCCCGCATGCT
ACGGCACAGTGCTTGCTGAATTTCAGCCTCTTGTAGAAGAGCCTAAGAACTTGGTCAAAACCAACTGTGATCTTTACGAGAAGCTTGGAGAATATGGATTC
CAAAATGCCATTCTAGTTCGCTACACCCAGAAAGCACCTCAGGTGTCAACCCCAACTCTCGTGGAGGCTGCAAGAAACCTAGGAAGAGTGGGCACCAAGTG
TTGTACACTTCCTGAAGATCAGAGACTGCCTTGTGTGGAAGACTATCTGTCTGCAATCCTGAACCGTGTGTGTCTGCTGCATGAGAAGACCCCAGTGAGTG
AGCATGTTACCAAGTGCTGTAGTGGATCCCTGGTGGAAAGGCGGCCATGCTTCTCTGCTCTGACAGTTGATGAAACATATGTCCCCAAAGAGTTTAAAGCT
GAGACCTTCACCTTCCACTCTGATATCTGCACACTTCCAGAGAAGGAGAAGCAGATTAAGAAACAAACGGCTCTTGCTGAGCTGGTGAAGCACAAGCCCAA
GGCTACAGCGGAGCAACTGAAGACTGTCATGGATGACTTTGCACAGTTCCTGGATACATGTTGCAAGGCTGCTGACAAGGACACCTGCTTCTCGACTGAGG
GTCCAAACCTTGTCACTAGATGCAAAGACGCCTTAGCC*GGAGGGGGCGGTTCC***CACCATCACCACCATCACTGATAA**

gWiz-LS-hCXCL9$^{23-125}$-(Gly$_4$Ser)$_2$-mouse SA-(Gly$_4$Ser)-His$_6$

ATGAGGGTCCCCGCTCAGCTCCTGGGGCTCCTGCTGCTCTGGCTCCCAGGTGCACGATGT**ACCCCCGTCGTGCGGAAGGGCAGATGCAGCTGTATCAGCAC**
CAACCAGGGCACCATCCATCTCCAGTCTCTGAAGGACCTGAAGCAGTTCGCCCCCAGCCCCAGCTGCGAGAAGATCGAGATTATCGCCACACTGAAAAACG
GGGTGCAGACCTGCCTGAACCCCGACAGCGCCGACGTGAAAGAACTGATCAAGAAATGGGAGAAACAGGTGTCCCAGAAGAAGAAGCAGAAGAACGGAAAG
AAGCACCAGAAAAAGAAAGTGCTGAAAGTGCGGAAGTCCCAGCGGAGCCGGCAGAAGAAAACCACAGGTGGAGGCGGTAGCGGAGGCGGAGGGTCGGAAGC
ACACAAGAGTGAGATCGCCCATCGGTATAATGATTTGGGAGAACAACATTTCAAAGGCCTAGTCCTGATTGCCTTTTCCCAGTATCTCCAGAAATGCTCAT
ACGATGAGCATGCCAAATTAGTGCAGGAAGTAACAGACTTTGCAAAGACGTGTGTTGCCGATGAGTCTGCCGCCAACTGTGACAAATCCCTTCACACTCTT
TTTGGAGATAAGTTGTGTGCCATTCCAAACCTCCGTGAAAACTATGGTGAACTGGCTGACTGCTGTACAAAACAAGAGCCCGAAAGAAACGAATGTTTCCT
GCAACACAAAGATGACAACCCCAGCCTGCCACCATTTGAAAGGCCAGAGGCTGAGGCCATGTGCACCTCCTTTAAGGAAAACCCAACCACCTTTATGGGAC
ACTATTTGCATGAAGTTGCCAGAAGACATCCTTATTTCTATGCCCCAGAACTTCTTTACTATGCTGAGCAGTACAATGAGATTCTGACCCAGTGTTGTGCA
GAGGCTGACAAGGAAAGCTGCCTGACCCCGAAGCTTGATGGTGTGAAGGAGAAAGCATTGGTCTCATCTGTCCGTCAGAGAATGAAGTGCTCCAGTATGCA

GAAGTTTGGAGAGAGAGCTTTTAAAGCATGGGCAGTAGCTCGTCTGAGCCAGACATTCCCCAATGCTGACTTTGCAGAAATCACCAAATTGGCAACAGACC
TGACCAAAGTCAACAAGGAGTGCTGCCATGGTGACCTGCTGGAATGCGCAGATGACAGGGCGGAACTTGCCAAGTACATGTGTGAAAACCAGGCGACTATC
TCCAGCAAACTGCAGACTTGCTGCGATAAACCACTGTTGAAGAAAGCCCACTGTCTTAGTGAGGTGGAGCATGACACCATGCCTGCTGATCTGCCTGCCAT
TGCTGCTGATTTTGTTGAGGACCAGGAAGTGTGCAAGAACTATGCTGAGGCCAAGGATGTCTTCCTGGGCACGTTCTTGTATGAATATTCAAGAAGACACC
CTGATTACTCTGTATCCCTGTTGCTGAGACTTGCTAAGAAATATGAAGCCACTCTGGAAAAGTGCTGCGCTGAAGCCAATCCTCCCGCATGCTACGGCACA
GTGCTTGCTGAATTTCAGCCTCTTGTAGAAGAGCCTAAGAACTTGGTCAAAACCAACTGTGATCTTTACGAGAAGCTTGGAGAATATGGATTCCAAAATGC
CATTCTAGTTCGCTACACCCAGAAAGCACCTCAGGTGTCAACCCCAACTCTCGTGGAGGCTGCAAGAAACCTAGGAAGAGTGGGCACCAAGTGTTGTACAC
TTCCTGAAGATCAGAGACTGCCTTGTGTGGAAGACTATCTGTCTGCAATCCTGAACCGTGTGTGTCTGCTGCATGAGAAGACCCCAGTGAGTGAGCATGTT
ACCAAGTGCTGTAGTGGATCCCTGGTGGAAAGGCGGCCATGCTTCTCTGCTCTGACAGTTGATGAAACATATGTCCCCAAAGAGTTTAAAGCTGAGACCTT
CACCTTCCACTCTGATATCTGCACACTTCCAGAGAAGGAGAAGCAGATTAAGAAACAAACGGCTCTTGCTGAGCTGGTGAAGCACAAGCCCAAGGCTACAG
CGGAGCAACTGAAGACTGTCATGGATGACTTTGCACAGTTCCTGGATACATGTTGCAAGGCTGCTGACAAGGACACCTGCTTCTCGACTGAGGGTCCAAAC
CTTGTCACTAGATGCAAAGACGCCTTAGCC*GGAGGGGGCGGTTCC***CACCATCACCACCATCACTGATAA**

gWiz-LS-hCXCL10$^{22\text{-}98}$-(Gly$_4$Ser)$_2$-mouse SA-(Gly$_4$Ser)-His$_6$

ATGAGGGTCCCCGCTCAGCTCCTGGGGCTCCTGCTGCTCTGGCTCCCAGGTGCACGATGT**GTGCCTCTGAGCAGAACCGTGCGGTGCACCTGTATCAGCAT**
CAGCAACCAGCCCGTGAACCCCAGAAGCCTGGAAAAGCTGGAAATCATCCCCGCCAGCCAGTTCTGCCCCAGAGTGGAAATTATCGCCACCATGAAGAAGA
AAGGCGAGAAGCGGTGCCTGAACCCCGAGAGCAAGGCCATCAAGAACCTGCTGAAGGCCGTGTCCAAAGAGCGGAGCAAGCGGAGCCCAGGTGGAGGCGGT
AGCGGAGGCGGAGGGTCGGAAGCACACAAGAGTGAGATCGCCCATCGGTATAATGATTTGGGAGAACAACATTTCAAAGGCCTAGTCCTGATTGCCTTTTC
CCAGTATCTCCAGAAATGCTCATACGATGAGCATGCCAAATTAGTGCAGGAAGTAACAGACTTTGCAAAGACGTGTGTTGCCGATGAGTCTGCCGCCAACT
GTGACAAATCCCTTCACACTCTTTTTGGAGATAAGTTGTGTGCCATTCCAAACCTCCGTGAAAACTATGGTGAACTGGCTGACTGCTGTACAAAACAAGAG
CCCGAAAGAAACGAATGTTTCCTGCAACACAAAGATGACAACCCCAGCCTGCCACCATTTGAAAGGCCAGAGGCTGAGGCCATGTGCACCTCCTTTAAGGA
AAACCCAACCACCTTTATGGGACACTATTTGCATGAAGTTGCCAGAAGACATCCTTATTTCTATGCCCCAGAACTTCTTTACTATGCTGAGCAGTACAATG
AGATTCTGACCCAGTGTTGTGCAGAGGCTGACAAGGAAAGCTGCCTGACCCCGAAGCTTGATGGTGTGAAGGAGAAAGCATTGGTCTCATCTGTCCGTCAG
AGAATGAAGTGCTCCAGTATGCAGAAGTTTGGAGAGAGAGCTTTTAAAGCATGGGCAGTAGCTCGTCTGAGCCAGACATTCCCCAATGCTGACTTTGCAGA
AATCACCAAATTGGCAACAGACCTGACCAAAGTCAACAAGGAGTGCTGCCATGGTGACCTGCTGGAATGCGCAGATGACAGGGCGGAACTTGCCAAGTACA
TGTGTGAAAACCAGGCGACTATCTCCAGCAAACTGCAGACTTGCTGCGATAAACCACTGTTGAAGAAAGCCCACTGTCTTAGTGAGGTGGAGCATGACACC
ATGCCTGCTGATCTGCCTGCCATTGCTGCTGATTTTGTTGAGGACCAGGAAGTGTGCAAGAACTATGCTGAGGCCAAGGATGTCTTCCTGGGCACGTTCTT
GTATGAATATTCAAGAAGACACCCTGATTACTCTGTATCCCTGTTGCTGAGACTTGCTAAGAAATATGAAGCCACTCTGGAAAAGTGCTGCGCTGAAGCCA
ATCCTCCCGCATGCTACGGCACAGTGCTTGCTGAATTTCAGCCTCTTGTAGAAGAGCCTAAGAACTTGGTCAAAACCAACTGTGATCTTTACGAGAAGCTT
GGAGAATATGGATTCCAAAATGCCATTCTAGTTCGCTACACCCAGAAAGCACCTCAGGTGTCAACCCCAACTCTCGTGGAGGCTGCAAGAAACCTAGGAAG
AGTGGGCACCAAGTGTTGTACACTTCCTGAAGATCAGAGACTGCCTTGTGTGGAAGACTATCTGTCTGCAATCCTGAACCGTGTGTGTCTGCTGCATGAGA
AGACCCCAGTGAGTGAGCATGTTACCAAGTGCTGTAGTGGATCCCTGGTGGAAAGGCGGCCATGCTTCTCTGCTCTGACAGTTGATGAAACATATGTCCCC
AAAGAGTTTAAAGCTGAGACCTTCACCTTCCACTCTGATATCTGCACACTTCCAGAGAAGGAGAAGCAGATTAAGAAACAAACGGCTCTTGCTGAGCTGGT
GAAGCACAAGCCCAAGGCTACAGCGGAGCAACTGAAGACTGTCATGGATGACTTTGCACAGTTCCTGGATACATGTTGCAAGGCTGCTGACAAGGACACCT

GCTTCTCGACTGAGGGTCCAAACCTTGTCACTAGATGCAAAGACGCCTTAGCC*GGAGGGGGCGGTTCC***CACCATCACCACCATCACTGATAA**

gWiz-LS-hCXCL11$^{22-94}$-(Gly$_4$Ser)$_2$-mouse SA-(Gly$_4$Ser)-His$_6$

ATGAGGGTCCCCGCTCAGCTCCTGGGGCTCCTGCTGCTCTGGCTCCCAGGTGCACGATGT**TTCCCCATGTTCAAGCGGGGCAGATGCCTGTGCATCGGCCC**
TGGCGTGAAAGCCGTGAAGGTGGCCGATATCGAGAAGGCCAGCATCATGTACCCCAGCAACAACTGCGACAAGATCGAAGTGATCATCACCCTGAAAGAGA
ACAAGGGCCAGAGATGCCTGAATCCCAAGTCCAAGCAGGCCCGGCTGATCATCAAGAAGGTGGAACGGAAGAACTTCGGTGGAGGCGGTAGCGGAGGCGGA
GGGTCGGAAGCACACAAGAGTGAGATCGCCCATCGGTATAATGATTTGGGAGAACAACATTTCAAAGGCCTAGTCCTGATTGCCTTTTCCCAGTATCTCCA
GAAATGCTCATACGATGAGCATGCCAAATTAGTGCAGGAAGTAACAGACTTTGCAAAGACGTGTGTTGCCGATGAGTCTGCCGCCAACTGTGACAAATCCC
TTCACACTCTTTTTGGAGATAAGTTGTGTGCCATTCCAAACCTCCGTGAAAACTATGGTGAACTGGCTGACTGCTGTACAAAACAAGAGCCCGAAAGAAAC
GAATGTTTCCTGCAACACAAAGATGACAACCCCAGCCTGCCACCATTTGAAAGGCCAGAGGCTGAGGCCATGTGCACCTCCTTTAAGGAAAACCCAACCAC
CTTTATGGGACACTATTTGCATGAAGTTGCCAGAAGACATCCTTATTTCTATGCCCCAGAACTTCTTTACTATGCTGAGCAGTACAATGAGATTCTGACCC
AGTGTTGTGCAGAGGCTGACAAGGAAAGCTGCCTGACCCCGAAGCTTGATGGTGTGAAGGAGAAAGCATTGGTCTCATCTGTCCGTCAGAGAATGAAGTGC
TCCAGTATGCAGAAGTTTGGAGAGAGAGCTTTTAAAGCATGGGCAGTAGCTCGTCTGAGCCAGACATTCCCCAATGCTGACTTTGCAGAAATCACCAAATT
GGCAACAGACCTGACCAAAGTCAACAAGGAGTGCTGCCATGGTGACCTGCTGGAATGCGCAGATGACAGGGCGGAACTTGCCAAGTACATGTGTGAAAACC
AGGCGACTATCTCCAGCAAACTGCAGACTTGCTGCGATAAACCACTGTTGAAGAAAGCCCACTGTCTTAGTGAGGTGGAGCATGACACCATGCCTGCTGAT
CTGCCTGCCATTGCTGCTGATTTTGTTGAGGACCAGGAAGTGTGCAAGAACTATGCTGAGGCCAAGGATGTCTTCCTGGGCACGTTCTTGTATGAATATTC
AAGAAGACACCCTGATTACTCTGTATCCCTGTTGCTGAGACTTGCTAAGAAATATGAAGCCACTCTGGAAAAGTGCTGCGCTGAAGCCAATCCTCCCGCAT
GCTACGGCACAGTGCTTGCTGAATTTCAGCCTCTTGTAGAAGAGCCTAAGAACTTGGTCAAAACCAACTGTGATCTTTACGAGAAGCTTGGAGAATATGGA
TTCCAAAATGCCATTCTAGTTCGCTACACCCAGAAAGCACCTCAGGTGTCAACCCCAACTCTCGTGGAGGCTGCAAGAAACCTAGGAAGAGTGGGCACCAA
GTGTTGTACACTTCCTGAAGATCAGAGACTGCCTTGTGTGGAAGACTATCTGTCTGCAATCCTGAACCGTGTGTGTCTGCTGCATGAGAAGACCCCAGTGA
GTGAGCATGTTACCAAGTGCTGTAGTGGATCCCTGGTGGAAAGGCGGCCATGCTTCTCTGCTCTGACAGTTGATGAAACATATGTCCCCAAAGAGTTTAAA
GCTGAGACCTTCACCTTCCACTCTGATATCTGCACACTTCCAGAGAAGGAGAAGCAGATTAAGAAACAAACGGCTCTTGCTGAGCTGGTGAAGCACAAGCC
CAAGGCTACAGCGGAGCAACTGAAGACTGTCATGGATGACTTTGCACAGTTCCTGGATACATGTTGCAAGGCTGCTGACAAGGACACCTGCTTCTCGACTG
AGGGTCCAAACCTTGTCACTAGATGCAAAGACGCCTTAGCC*GGAGGGGGCGGTTCC***CACCATCACCACCATCACTGATAA**

gWiz-LS-mCXCL1$^{25-96}$-(Gly$_4$Ser)$_2$-mouse SA-(Gly$_4$Ser)-His$_6$

ATGAGGGTCCCCGCTCAGCTCCTGGGGCTCCTGCTGCTCTGGCTCCCAGGTGCACGATGT**GCCCCTATTGCCAACGAGCTGCGGTGCCAGTGCCTGCAGAC**
CATGGCCGGCATCCACCTGAAGAACATCCAGAGCCTGAAGGTGCTGCCCAGCGGCCCTCACTGCACCCAGACCGAAGTGATCGCCACCCTGAAGAACGGCA
GAGAGGCCTGCCTGGATCCCGAGGCCCCCCTGGTGCAGAAAATCGTGCAGAAAATGCTGAAGGGCGTGCCCAAGGGTGGAGGCGGTAGCGGAGGCGGAGGG
TCGGAAGCACACAAGAGTGAGATCGCCCATCGGTATAATGATTTGGGAGAACAACATTTCAAAGGCCTAGTCCTGATTGCCTTTTCCCAGTATCTCCAGAA
ATGCTCATACGATGAGCATGCCAAATTAGTGCAGGAAGTAACAGACTTTGCAAAGACGTGTGTTGCCGATGAGTCTGCCGCCAACTGTGACAAATCCCTTC
ACACTCTTTTTGGAGATAAGTTGTGTGCCATTCCAAACCTCCGTGAAAACTATGGTGAACTGGCTGACTGCTGTACAAAACAAGAGCCCGAAAGAAACGAA
TGTTTCCTGCAACACAAAGATGACAACCCCAGCCTGCCACCATTTGAAAGGCCAGAGGCTGAGGCCATGTGCACCTCCTTTAAGGAAAACCCAACCACCTT

TATGGGACACTATTTGCATGAAGTTGCCAGAAGACATCCTTATTTCTATGCCCCAGAACTTCTTTACTATGCTGAGCAGTACAATGAGATTCTGACCCAGT
GTTGTGCAGAGGCTGACAAGGAAAGCTGCCTGACCCCGAAGCTTGATGGTGTGAAGGAGAAAGCATTGGTCTCATCTGTCCGTCAGAGAATGAAGTGCTCC
AGTATGCAGAAGTTTGGAGAGAGAGCTTTTAAAGCATGGGCAGTAGCTCGTCTGAGCCAGACATTCCCCAATGCTGACTTTGCAGAAATCACCAAATTGGC
AACAGACCTGACCAAAGTCAACAAGGAGTGCTGCCATGGTGACCTGCTGGAATGCGCAGATGACAGGGCGGAACTTGCCAAGTACATGTGTGAAAACCAGG
CGACTATCTCCAGCAAACTGCAGACTTGCTGCGATAAACCACTGTTGAAGAAAGCCCACTGTCTTAGTGAGGTGGAGCATGACACCATGCCTGCTGATCTG
CCTGCCATTGCTGCTGATTTTGTTGAGGACCAGGAAGTGTGCAAGAACTATGCTGAGGCCAAGGATGTCTTCCTGGGCACGTTCTTGTATGAATATTCAAG
AAGACACCCTGATTACTCTGTATCCCTGTTGCTGAGACTTGCTAAGAAATATGAAGCCACTCTGGAAAAGTGCTGCGCTGAAGCCAATCCTCCCGCATGCT
ACGGCACAGTGCTTGCTGAATTTCAGCCTCTTGTAGAAGAGCCTAAGAACTTGGTCAAAACCAACTGTGATCTTTACGAGAAGCTTGGAGAATATGGATTC
CAAAATGCCATTCTAGTTCGCTACACCCAGAAAGCACCTCAGGTGTCAACCCCAACTCTCGTGGAGGCTGCAAGAAACCTAGGAAGAGTGGGCACCAAGTG
TTGTACACTTCCTGAAGATCAGAGACTGCCTTGTGTGGAAGACTATCTGTCTGCAATCCTGAACCGTGTGTGTCTGCTGCATGAGAAGACCCCAGTGAGTG
AGCATGTTACCAAGTGCTGTAGTGGATCCCTGGTGGAAAGGCGGCCATGCTTCTCTGCTCTGACAGTTGATGAAACATATGTCCCCAAAGAGTTTAAAGCT
GAGACCTTCACCTTCCACTCTGATATCTGCACACTTCCAGAGAAGGAGAAGCAGATTAAGAAACAAACGGCTCTTGCTGAGCTGGTGAAGCACAAGCCCAA
GGCTACAGCGGAGCAACTGAAGACTGTCATGGATGACTTTGCACAGTTCCTGGATACATGTTGCAAGGCTGCTGACAAGGACACCTGCTTCTCGACTGAGG
GTCCAAACCTTGTCACTAGATGCAAAGACGCCTTAGCC*GGAGGGGGCGGTTCC***CACCATCACCACCATCAC****TGATAA**

gWiz-LS-mCXCL2$^{28\text{-}100}$-(Gly$_4$Ser)$_2$-mouse SA-(Gly$_4$Ser)-His$_6$

ATGAGGGTCCCCGCTCAGCTCCTGGGGCTCCTGCTGCTCTGGCTCCCAGGTGCACGATGT**GCCGTCGTGGCCAGCGAGCTGCGGTGCCAGTGCCTGAAAAC**
CCTGCCCCGGGTGGACTTCAAGAACATCCAGAGCCTGAGCGTGACCCCCCCTGGCCCTCACTGTGCCCAGACCGAAGTGATCGCCACCCTGAAGGGCGGCC
AGAAAGTGTGCCTGGACCCCGAGGCCCCCCTGGTGCAGAAGATCATCCAGAAGATCCTGAACAAGGGCAAGGCCAACGGTGGAGGCGGTAGCGGAGGCGGA
GGGTCGGAAGCACACAAGAGTGAGATCGCCCATCGGTATAATGATTTGGGAGAACAACATTTCAAAGGCCTAGTCCTGATTGCCTTTTCCCAGTATCTCCA
GAAATGCTCATACGATGAGCATGCCAAATTAGTGCAGGAAGTAACAGACTTTGCAAAGACGTGTGTTGCCGATGAGTCTGCCGCCAACTGTGACAAATCCC
TTCACACTCTTTTTGGAGATAAGTTGTGTGCCATTCCAAACCTCCGTGAAAACTATGGTGAACTGGCTGACTGCTGTACAAAACAAGAGCCCGAAAGAAAC
GAATGTTTCCTGCAACACAAAGATGACAACCCCAGCCTGCCACCATTTGAAAGGCCAGAGGCTGAGGCCATGTGCACCTCCTTTAAGGAAAACCCAACCAC
CTTTATGGGACACTATTTGCATGAAGTTGCCAGAAGACATCCTTATTTCTATGCCCCAGAACTTCTTTACTATGCTGAGCAGTACAATGAGATTCTGACCC
AGTGTTGTGCAGAGGCTGACAAGGAAAGCTGCCTGACCCCGAAGCTTGATGGTGTGAAGGAGAAAGCATTGGTCTCATCTGTCCGTCAGAGAATGAAGTGC
TCCAGTATGCAGAAGTTTGGAGAGAGAGCTTTTAAAGCATGGGCAGTAGCTCGTCTGAGCCAGACATTCCCCAATGCTGACTTTGCAGAAATCACCAAATT
GGCAACAGACCTGACCAAAGTCAACAAGGAGTGCTGCCATGGTGACCTGCTGGAATGCGCAGATGACAGGGCGGAACTTGCCAAGTACATGTGTGAAAACC
AGGCGACTATCTCCAGCAAACTGCAGACTTGCTGCGATAAACCACTGTTGAAGAAAGCCCACTGTCTTAGTGAGGTGGAGCATGACACCATGCCTGCTGAT
CTGCCTGCCATTGCTGCTGATTTTGTTGAGGACCAGGAAGTGTGCAAGAACTATGCTGAGGCCAAGGATGTCTTCCTGGGCACGTTCTTGTATGAATATTC
AAGAAGACACCCTGATTACTCTGTATCCCTGTTGCTGAGACTTGCTAAGAAATATGAAGCCACTCTGGAAAAGTGCTGCGCTGAAGCCAATCCTCCCGCAT
GCTACGGCACAGTGCTTGCTGAATTTCAGCCTCTTGTAGAAGAGCCTAAGAACTTGGTCAAAACCAACTGTGATCTTTACGAGAAGCTTGGAGAATATGGA
TTCCAAAATGCCATTCTAGTTCGCTACACCCAGAAAGCACCTCAGGTGTCAACCCCAACTCTCGTGGAGGCTGCAAGAAACCTAGGAAGAGTGGGCACCAA
GTGTTGTACACTTCCTGAAGATCAGAGACTGCCTTGTGTGGAAGACTATCTGTCTGCAATCCTGAACCGTGTGTGTCTGCTGCATGAGAAGACCCCAGTGA
GTGAGCATGTTACCAAGTGCTGTAGTGGATCCCTGGTGGAAAGGCGGCCATGCTTCTCTGCTCTGACAGTTGATGAAACATATGTCCCCAAAGAGTTTAAA

GCTGAGACCTTCACCTTCCACTCTGATATCTGCACACTTCCAGAGAAGGAGAAGCAGATTAAGAAACAAACGGCTCTTGCTGAGCTGGTGAAGCACAAGCC
CAAGGCTACAGCGGAGCAACTGAAGACTGTCATGGATGACTTTGCACAGTTCCTGGATACATGTTGCAAGGCTGCTGACAAGGACACCTGCTTCTCGACTG
AGGGTCCAAACCTTGTCACTAGATGCAAAGACGCCTTAGCC*GGAGGGGGCGGTTCC***CACCATCACCACCATCACTGATAA**

gWiz-LS-mCXCL3$^{28\text{-}100}$-(Gly$_4$Ser)$_2$-mouse SA-(Gly$_4$Ser)-His$_6$

ATGAGGGTCCCCGCTCAGCTCCTGGGGCTCCTGCTGCTCTGGCTCCCAGGTGCACGATGT**GCTGTGGTGGCCTCTGAGCTGAGATGCCAGTGCCTGAACAC**
CCTGCCCCGGGTGGACTTCGAGACAATCCAGAGCCTGACCGTGACCCCCCCTGGCCCTCACTGTACCCAGACAGAAGTGATCGCCACCCTGAAGGACGGCC
AGGAAGTGTGCCTGAATCCCCAGGGCCCCAGACTCCAGATCATCATCAAGAAGATCCTGAAGTCCGGCAAGAGCAGCGGTGGAGGCGGTAGCGGAGGCGGA
GGGTCGGAAGCACACAAGAGTGAGATCGCCCATCGGTATAATGATTTGGGAGAACAACATTTCAAAGGCCTAGTCCTGATTGCCTTTTCCCAGTATCTCCA
GAAATGCTCATACGATGAGCATGCCAAATTAGTGCAGGAAGTAACAGACTTTGCAAAGACGTGTGTTGCCGATGAGTCTGCCGCCAACTGTGACAAATCCC
TTCACACTCTTTTTGGAGATAAGTTGTGTGCCATTCCAAACCTCCGTGAAAACTATGGTGAACTGGCTGACTGCTGTACAAAACAAGAGCCCGAAAGAAAC
GAATGTTTCCTGCAACACAAAGATGACAACCCCAGCCTGCCACCATTTGAAAGGCCAGAGGCTGAGGCCATGTGCACCTCCTTTAAGGAAAACCCAACCAC
CTTTATGGGACACTATTTGCATGAAGTTGCCAGAAGACATCCTTATTTCTATGCCCCAGAACTTCTTTACTATGCTGAGCAGTACAATGAGATTCTGACCC
AGTGTTGTGCAGAGGCTGACAAGGAAAGCTGCCTGACCCCGAAGCTTGATGGTGTGAAGGAGAAAGCATTGGTCTCATCTGTCCGTCAGAGAATGAAGTGC
TCCAGTATGCAGAAGTTTGGAGAGAGAGCTTTTAAAGCATGGGCAGTAGCTCGTCTGAGCCAGACATTCCCCAATGCTGACTTTGCAGAAATCACCAAATT
GGCAACAGACCTGACCAAAGTCAACAAGGAGTGCTGCCATGGTGACCTGCTGGAATGCGCAGATGACAGGGCGGAACTTGCCAAGTACATGTGTGAAAACC
AGGCGACTATCTCCAGCAAACTGCAGACTTGCTGCGATAAACCACTGTTGAAGAAAGCCCACTGTCTTAGTGAGGTGGAGCATGACACCATGCCTGCTGAT
CTGCCTGCCATTGCTGCTGATTTTGTTGAGGACCAGGAAGTGTGCAAGAACTATGCTGAGGCCAAGGATGTCTTCCTGGGCACGTTCTTGTATGAATATTC
AAGAAGACACCCTGATTACTCTGTATCCCTGTTGCTGAGACTTGCTAAGAAATATGAAGCCACTCTGGAAAAGTGCTGCGCTGAAGCCAATCCTCCCGCAT
GCTACGGCACAGTGCTTGCTGAATTTCAGCCTCTTGTAGAAGAGCCTAAGAACTTGGTCAAAACCAACTGTGATCTTTACGAGAAGCTTGGAGAATATGGA
TTCCAAAATGCCATTCTAGTTCGCTACACCCAGAAAGCACCTCAGGTGTCAACCCCAACTCTCGTGGAGGCTGCAAGAAACCTAGGAAGAGTGGGCACCAA
GTGTTGTACACTTCCTGAAGATCAGAGACTGCCTTGTGTGGAAGACTATCTGTCTGCAATCCTGAACCGTGTGTGTCTGCTGCATGAGAAGACCCCAGTGA
GTGAGCATGTTACCAAGTGCTGTAGTGGATCCCTGGTGGAAAGGCGGCCATGCTTCTCTGCTCTGACAGTTGATGAAACATATGTCCCCAAAGAGTTTAAA
GCTGAGACCTTCACCTTCCACTCTGATATCTGCACACTTCCAGAGAAGGAGAAGCAGATTAAGAAACAAACGGCTCTTGCTGAGCTGGTGAAGCACAAGCC
CAAGGCTACAGCGGAGCAACTGAAGACTGTCATGGATGACTTTGCACAGTTCCTGGATACATGTTGCAAGGCTGCTGACAAGGACACCTGCTTCTCGACTG
AGGGTCCAAACCTTGTCACTAGATGCAAAGACGCCTTAGCC*GGAGGGGGCGGTTCC***CACCATCACCACCATCACTGATAA**

gWiz-LS-mCXCL4$^{30\text{-}105}$-(Gly$_4$Ser)$_2$-mouse SA-(Gly$_4$Ser)-His$_6$

ATGAGGGTCCCCGCTCAGCTCCTGGGGCTCCTGCTGCTCTGGCTCCCAGGTGCACGATGT**GTGACATCTGCCGGCCCTGAGGAAAGCGACGGCGATCTGTC**
TTGCGTGTGCGTGAAAACCATCAGCAGCGGCATCCACCTGAAGCACATCACCAGCCTGGAAGTGATCAAGGCCGGCAGGCACTGTGCCGTGCCTCAGCTGA
TTGCCACCCTGAAGAACGGCCGGAAGATCTGCCTGGACAGACAGGCCCCCCTGTACAAGAAAGTGATTAAGAAGATCCTGGAAAGCGGTGGAGGCGGTAGC
GGAGGCGGAGGGTCGGAAGCACACAAGAGTGAGATCGCCCATCGGTATAATGATTTGGGAGAACAACATTTCAAAGGCCTAGTCCTGATTGCCTTTTCCCA
GTATCTCCAGAAATGCTCATACGATGAGCATGCCAAATTAGTGCAGGAAGTAACAGACTTTGCAAAGACGTGTGTTGCCGATGAGTCTGCCGCCAACTGTG
ACAAATCCCTTCACACTCTTTTTGGAGATAAGTTGTGTGCCATTCCAAACCTCCGTGAAAACTATGGTGAACTGGCTGACTGCTGTACAAAACAAGAGCCC
GAAAGAAACGAATGTTTCCTGCAACACAAAGATGACAACCCCAGCCTGCCACCATTTGAAAGGCCAGAGGCTGAGGCCATGTGCACCTCCTTTAAGGAAAA
CCCAACCACCTTTATGGGACACTATTTGCATGAAGTTGCCAGAAGACATCCTTATTTCTATGCCCCAGAACTTCTTTACTATGCTGAGCAGTACAATGAGA
TTCTGACCCAGTGTTGTGCAGAGGCTGACAAGGAAAGCTGCCTGACCCCGAAGCTTGATGGTGTGAAGGAGAAAGCATTGGTCTCATCTGTCCGTCAGAGA
ATGAAGTGCTCCAGTATGCAGAAGTTTGGAGAGAGAGCTTTTAAAGCATGGGCAGTAGCTCGTCTGAGCCAGACATTCCCCAATGCTGACTTTGCAGAAAT

CACCAAATTGGCAACAGACCTGACCAAAGTCAACAAGGAGTGCTGCCATGGTGACCTGCTGGAATGCGCAGATGACAGGGCGGAACTTGCCAAGTACATGT
GTGAAAACCAGGCGACTATCTCCAGCAAACTGCAGACTTGCTGCGATAAACCACTGTTGAAGAAAGCCCACTGTCTTAGTGAGGTGGAGCATGACACCATG
CCTGCTGATCTGCCTGCCATTGCTGCTGATTTTGTTGAGGACCAGGAAGTGTGCAAGAACTATGCTGAGGCCAAGGATGTCTTCCTGGGCACGTTCTTGTA
TGAATATTCAAGAAGACACCCTGATTACTCTGTATCCCTGTTGCTGAGACTTGCTAAGAAATATGAAGCCACTCTGGAAAAGTGCTGCGCTGAAGCCAATC
CTCCCGCATGCTACGGCACAGTGCTTGCTGAATTTCAGCCTCTTGTAGAAGAGCCTAAGAACTTGGTCAAAACCAACTGTGATCTTTACGAGAAGCTTGGA
GAATATGGATTCCAAAATGCCATTCTAGTTCGCTACACCCAGAAAGCACCTCAGGTGTCAACCCCAACTCTCGTGGAGGCTGCAAGAAACCTAGGAAGAGT
GGGCACCAAGTGTTGTACACTTCCTGAAGATCAGAGACTGCCTTGTGTGGAAGACTATCTGTCTGCAATCCTGAACCGTGTGTGTCTGCTGCATGAGAAGA
CCCCAGTGAGTGAGCATGTTACCAAGTGCTGTAGTGGATCCCTGGTGGAAAGGCGGCCATGCTTCTCTGCTCTGACAGTTGATGAAACATATGTCCCCAAA
GAGTTTAAAGCTGAGACCTTCACCTTCCACTCTGATATCTGCACACTTCCAGAGAAGGAGAAGCAGATTAAGAAACAAACGGCTCTTGCTGAGCTGGTGAA
GCACAAGCCCAAGGCTACAGCGGAGCAACTGAAGACTGTCATGGATGACTTTGCACAGTTCCTGGATACATGTTGCAAGGCTGCTGACAAGGACACCTGCT
TCTCGACTGAGGGTCCAAACCTTGTCACTAGATGCAAAGACGCCTTAGCC*GGAGGGGGCGGTTCC***CACCATCACCACCATCACTGATAA**

gWiz-LS-mCXCL5$^{48-118}$-(Gly$_4$Ser)$_2$-mouse SA-(Gly$_4$Ser)-His$_6$

ATGAGGGTCCCCGCTCAGCTCCTGGGGCTCCTGCTGCTCTGGCTCCCAGGTGCACGATGT**GCCACCGAGCTGAGATGCGTGTGCCTGACCGTGACCCCCAA**
GATCAACCCCAAGCTGATCGCCAACCTGGAAGTGATCCCTGCCGGCCCTCAGTGCCCCACCGTGGAAGTGATTGCCAAGCTGAAGAACCAGAAAGAAGTGT
GCCTGGACCCCGAGGCCCCCGTGATCAAGAAGATCATCCAGAAGATCCTGGGCAGCGACAAGAAGAAAGCCGGTGGAGGCGGTAGCGGAGGCGGAGGGTCG
GAAGCACACAAGAGTGAGATCGCCCATCGGTATAATGATTTGGGAGAACAACATTTCAAAGGCCTAGTCCTGATTGCCTTTTCCCAGTATCTCCAGAAATG
CTCATACGATGAGCATGCCAAATTAGTGCAGGAAGTAACAGACTTTGCAAAGACGTGTGTTGCCGATGAGTCTGCCGCCAACTGTGACAAATCCCTTCACA
CTCTTTTTGGAGATAAGTTGTGTGCCATTCCAAACCTCCGTGAAAACTATGGTGAACTGGCTGACTGCTGTACAAAACAAGAGCCCGAAAGAAACGAATGT
TTCCTGCAACACAAAGATGACAACCCCAGCCTGCCACCATTTGAAAGGCCAGAGGCTGAGGCCATGTGCACCTCCTTTAAGGAAAACCCAACCACCTTTAT
GGGACACTATTTGCATGAAGTTGCCAGAAGACATCCTTATTTCTATGCCCCAGAACTTCTTTACTATGCTGAGCAGTACAATGAGATTCTGACCCAGTGTT
GTGCAGAGGCTGACAAGGAAAGCTGCCTGACCCCGAAGCTTGATGGTGTGAAGGAGAAAGCATTGGTCTCATCTGTCCGTCAGAGAATGAAGTGCTCCAGT
ATGCAGAAGTTTGGAGAGAGAGCTTTTAAAGCATGGGCAGTAGCTCGTCTGAGCCAGACATTCCCCAATGCTGACTTTGCAGAAATCACCAAATTGGCAAC
AGACCTGACCAAAGTCAACAAGGAGTGCTGCCATGGTGACCTGCTGGAATGCGCAGATGACAGGGCGGAACTTGCCAAGTACATGTGTGAAAACCAGGCGA
CTATCTCCAGCAAACTGCAGACTTGCTGCGATAAACCACTGTTGAAGAAAGCCCACTGTCTTAGTGAGGTGGAGCATGACACCATGCCTGCTGATCTGCCT
GCCATTGCTGCTGATTTTGTTGAGGACCAGGAAGTGTGCAAGAACTATGCTGAGGCCAAGGATGTCTTCCTGGGCACGTTCTTGTATGAATATTCAAGAAG
ACACCCTGATTACTCTGTATCCCTGTTGCTGAGACTTGCTAAGAAATATGAAGCCACTCTGGAAAAGTGCTGCGCTGAAGCCAATCCTCCCGCATGCTACG
GCACAGTGCTTGCTGAATTTCAGCCTCTTGTAGAAGAGCCTAAGAACTTGGTCAAAACCAACTGTGATCTTTACGAGAAGCTTGGAGAATATGGATTCCAA
AATGCCATTCTAGTTCGCTACACCCAGAAAGCACCTCAGGTGTCAACCCCAACTCTCGTGGAGGCTGCAAGAAACCTAGGAAGAGTGGGCACCAAGTGTTG
TACACTTCCTGAAGATCAGAGACTGCCTTGTGTGGAAGACTATCTGTCTGCAATCCTGAACCGTGTGTGTCTGCTGCATGAGAAGACCCCAGTGAGTGAGC
ATGTTACCAAGTGCTGTAGTGGATCCCTGGTGGAAAGGCGGCCATGCTTCTCTGCTCTGACAGTTGATGAAACATATGTCCCCAAAGAGTTTAAAGCTGAG
ACCTTCACCTTCCACTCTGATATCTGCACACTTCCAGAGAAGGAGAAGCAGATTAAGAAACAAACGGCTCTTGCTGAGCTGGTGAAGCACAAGCCCAAGGC
TACAGCGGAGCAACTGAAGACTGTCATGGATGACTTTGCACAGTTCCTGGATACATGTTGCAAGGCTGCTGACAAGGACACCTGCTTCTCGACTGAGGGTC
CAAACCTTGTCACTAGATGCAAAGACGCCTTAGCC*GGAGGGGGCGGTTCC***CACCATCACCACCATCACTGATAA**

gWiz-LS-mCXCL7$^{48-113}$-(Gly$_4$Ser)$_2$-mouse SA-(Gly$_4$Ser)-His$_6$

ATGAGGGTCCCCGCTCAGCTCCTGGGGCTCCTGCTGCTCTGGCTCCCAGGTGCACGATGT**ATCGAGCTGCGGTGCCGGTGCACCAACACCATCAGCGGCAT**
CCCTTTCAACAGCATCAGCCTCGTGAACGTGTACAGACCCGGCGTGCACTGCGCCGACGTGGAAGTGATTGCTACACTGAAGAATGGGCAGAAAACCTGCC

TGGACCCCAACGCCCCTGGCGTGAAGCGGATCGTGATGAAGATTCTGGAAGGCTACGGTGGAGGCGGTAGCGGAGGCGGAGGGTCGGAAGCACACAAGAGT
GAGATCGCCCATCGGTATAATGATTTGGGAGAACAACATTTCAAAGGCCTAGTCCTGATTGCCTTTTCCCAGTATCTCCAGAAATGCTCATACGATGAGCA
TGCCAAATTAGTGCAGGAAGTAACAGACTTTGCAAAGACGTGTGTTGCCGATGAGTCTGCCGCCAACTGTGACAAATCCCTTCACACTCTTTTTGGAGATA
AGTTGTGTGCCATTCCAAACCTCCGTGAAAACTATGGTGAACTGGCTGACTGCTGTACAAAACAAGAGCCCGAAAGAAACGAATGTTTCCTGCAACACAAA
GATGACAACCCCAGCCTGCCACCATTTGAAAGGCCAGAGGCTGAGGCCATGTGCACCTCCTTTAAGGAAAACCCAACCACCTTTATGGGACACTATTTGCA
TGAAGTTGCCAGAAGACATCCTTATTTCTATGCCCCAGAACTTCTTTACTATGCTGAGCAGTACAATGAGATTCTGACCCAGTGTTGTGCAGAGGCTGACA
AGGAAAGCTGCCTGACCCCGAAGCTTGATGGTGTGAAGGAGAAAGCATTGGTCTCATCTGTCCGTCAGAGAATGAAGTGCTCCAGTATGCAGAAGTTTGGA
GAGAGAGCTTTTAAAGCATGGGCAGTAGCTCGTCTGAGCCAGACATTCCCCAATGCTGACTTTGCAGAAATCACCAAATTGGCAACAGACCTGACCAAAGT
CAACAAGGAGTGCTGCCATGGTGACCTGCTGGAATGCGCAGATGACAGGGCGGAACTTGCCAAGTACATGTGTGAAAACCAGGCGACTATCTCCAGCAAAC
TGCAGACTTGCTGCGATAAACCACTGTTGAAGAAAGCCCACTGTCTTAGTGAGGTGGAGCATGACACCATGCCTGCTGATCTGCCTGCCATTGCTGCTGAT
TTTGTTGAGGACCAGGAAGTGTGCAAGAACTATGCTGAGGCCAAGGATGTCTTCCTGGGCACGTTCTTGTATGAATATTCAAGAAGACACCCTGATTACTC
TGTATCCCTGTTGCTGAGACTTGCTAAGAAATATGAAGCCACTCTGGAAAAGTGCTGCGCTGAAGCCAATCCTCCCGCATGCTACGGCACAGTGCTTGCTG
AATTTCAGCCTCTTGTAGAAGAGCCTAAGAACTTGGTCAAAACCAACTGTGATCTTTACGAGAAGCTTGGAGAATATGGATTCCAAAATGCCATTCTAGTT
CGCTACACCCAGAAAGCACCTCAGGTGTCAACCCCAACTCTCGTGGAGGCTGCAAGAAACCTAGGAAGAGTGGGCACCAAGTGTTGTACACTTCCTGAAGA
TCAGAGACTGCCTTGTGTGGAAGACTATCTGTCTGCAATCCTGAACCGTGTGTGTCTGCTGCATGAGAAGACCCCAGTGAGTGAGCATGTTACCAAGTGCT
GTAGTGGATCCCTGGTGGAAAGGCGGCCATGCTTCTCTGCTCTGACAGTTGATGAAACATATGTCCCCAAAGAGTTTAAAGCTGAGACCTTCACCTTCCAC
TCTGATATCTGCACACTTCCAGAGAAGGAGAAGCAGATTAAGAAACAAACGGCTCTTGCTGAGCTGGTGAAGCACAAGCCCAAGGCTACAGCGGAGCAACT
GAAGACTGTCATGGATGACTTTGCACAGTTCCTGGATACATGTTGCAAGGCTGCTGACAAGGACACCTGCTTCTCGACTGAGGGTCCAAACCTTGTCACTA
GATGCAAAGACGCCTTAGCC*GGAGGGGGCGGTTCC***CACCATCACCACCATCACTGATAA**

gWiz-LS-mCXCL9$^{22-126}$-(Gly$_4$Ser)$_2$-mouse SA-(Gly$_4$Ser)-His$_6$

ATGAGGGTCCCCGCTCAGCTCCTGGGGCTCCTGCTGCTCTGGCTCCCAGGTGCACGATGT**ACCCTCGTGATCCGGAACGCCCGGTGCAGCTGTATCAGCAC**
CAGCAGAGGCACCATCCACTACAAGAGCCTGAAGGATCTGAAGCAGTTCGCCCCCAGCCCCAACTGCAACAAGACCGAGATTATCGCCACACTGAAAAACG
GGGACCAGACCTGTCTGGACCCCGACAGCGCCAACGTGAAGAAACTGATGAAGGAATGGGAGAAGAAGATCAGCCAGAAGAAGAAGCAGAAGCGGGGCAAG
AAACACCAGAAAAACATGAAGAACCGGAAGCCCAAGACCCCCCAGAGCCGGCGGAGATCCAGAAAGACCACAGGTGGAGGCGGTAGCGGAGGCGGAGGGTC
GGAAGCACACAAGAGTGAGATCGCCCATCGGTATAATGATTTGGGAGAACAACATTTCAAAGGCCTAGTCCTGATTGCCTTTTCCCAGTATCTCCAGAAAT
GCTCATACGATGAGCATGCCAAATTAGTGCAGGAAGTAACAGACTTTGCAAAGACGTGTGTTGCCGATGAGTCTGCCGCCAACTGTGACAAATCCCTTCAC
ACTCTTTTTGGAGATAAGTTGTGTGCCATTCCAAACCTCCGTGAAAACTATGGTGAACTGGCTGACTGCTGTACAAAACAAGAGCCCGAAAGAAACGAATG
TTTCCTGCAACACAAAGATGACAACCCCAGCCTGCCACCATTTGAAAGGCCAGAGGCTGAGGCCATGTGCACCTCCTTTAAGGAAAACCCAACCACCTTTA
TGGGACACTATTTGCATGAAGTTGCCAGAAGACATCCTTATTTCTATGCCCCAGAACTTCTTTACTATGCTGAGCAGTACAATGAGATTCTGACCCAGTGT
TGTGCAGAGGCTGACAAGGAAAGCTGCCTGACCCCGAAGCTTGATGGTGTGAAGGAGAAAGCATTGGTCTCATCTGTCCGTCAGAGAATGAAGTGCTCCAG
TATGCAGAAGTTTGGAGAGAGAGCTTTTAAAGCATGGGCAGTAGCTCGTCTGAGCCAGACATTCCCCAATGCTGACTTTGCAGAAATCACCAAATTGGCAA
CAGACCTGACCAAAGTCAACAAGGAGTGCTGCCATGGTGACCTGCTGGAATGCGCAGATGACAGGGCGGAACTTGCCAAGTACATGTGTGAAAACCAGGCG
ACTATCTCCAGCAAACTGCAGACTTGCTGCGATAAACCACTGTTGAAGAAAGCCCACTGTCTTAGTGAGGTGGAGCATGACACCATGCCTGCTGATCTGCC
TGCCATTGCTGCTGATTTTGTTGAGGACCAGGAAGTGTGCAAGAACTATGCTGAGGCCAAGGATGTCTTCCTGGGCACGTTCTTGTATGAATATTCAAGAA
GACACCCTGATTACTCTGTATCCCTGTTGCTGAGACTTGCTAAGAAATATGAAGCCACTCTGGAAAAGTGCTGCGCTGAAGCCAATCCTCCCGCATGCTAC
GGCACAGTGCTTGCTGAATTTCAGCCTCTTGTAGAAGAGCCTAAGAACTTGGTCAAAACCAACTGTGATCTTTACGAGAAGCTTGGAGAATATGGATTCCA
AAATGCCATTCTAGTTCGCTACACCCAGAAAGCACCTCAGGTGTCAACCCCAACTCTCGTGGAGGCTGCAAGAAACCTAGGAAGAGTGGGCACCAAGTGTT
GTACACTTCCTGAAGATCAGAGACTGCCTTGTGTGGAAGACTATCTGTCTGCAATCCTGAACCGTGTGTGTCTGCTGCATGAGAAGACCCCAGTGAGTGAG

CATGTTACCAAGTGCTGTAGTGGATCCCTGGTGGAAAGGCGGCCATGCTTCTCTGCTCTGACAGTTGATGAAACATATGTCCCCAAAGAGTTTAAAGCTGA
GACCTTCACCTTCCACTCTGATATCTGCACACTTCCAGAGAAGGAGAAGCAGATTAAGAAACAAACGGCTCTTGCTGAGCTGGTGAAGCACAAGCCCAAGG
CTACAGCGGAGCAACTGAAGACTGTCATGGATGACTTTGCACAGTTCCTGGATACATGTTGCAAGGCTGCTGACAAGGACACCTGCTTCTCGACTGAGGGT
CCAAACCTTGTCACTAGATGCAAAGACGCCTTAGCC*GGAGGGGGCGGTTCC***CACCATCACCACCATCACTGATAA**

gWiz-LS-mCXCL10$^{22-98}$-(Gly$_4$Ser)$_2$-mouse SA-(Gly$_4$Ser)-His$_6$

ATGAGGGTCCCCGCTCAGCTCCTGGGGCTCCTGCTGCTCTGGCTCCCAGGTGCACGATGT**ATCCCACTGGCCAGAACCGTGCGGTGCAACTGCATCCACAT**
CGACGATGGCCCCGTGCGGATGAGAGCCATCGGCAAGCTGGAAATCATCCCCGCCAGCCTGAGCTGCCCCAGAGTGGAAATTATCGCCACCATGAAGAAGA
ACGACGAGCAGCGGTGCCTGAACCCCGAGAGCAAGACCATCAAGAACCTGATGAAGGCCTTTAGCCAGAAGCGGAGCAAGAGGGCCCCAGGTGGAGGCGGT
AGCGGAGGCGGAGGGTCGGAAGCACACAAGAGTGAGATCGCCCATCGGTATAATGATTTGGGAGAACAACATTTCAAAGGCCTAGTCCTGATTGCCTTTTC
CCAGTATCTCCAGAAATGCTCATACGATGAGCATGCCAAATTAGTGCAGGAAGTAACAGACTTTGCAAAGACGTGTGTTGCCGATGAGTCTGCCGCCAACT
GTGACAAATCCCTTCACACTCTTTTTGGAGATAAGTTGTGTGCCATTCCAAACCTCCGTGAAAACTATGGTGAACTGGCTGACTGCTGTACAAAACAAGAG
CCCGAAAGAAACGAATGTTTCCTGCAACACAAAGATGACAACCCCAGCCTGCCACCATTTGAAAGGCCAGAGGCTGAGGCCATGTGCACCTCCTTTAAGGA
AAACCCAACCACCTTTATGGGACACTATTTGCATGAAGTTGCCAGAAGACATCCTTATTTCTATGCCCCAGAACTTCTTTACTATGCTGAGCAGTACAATG
AGATTCTGACCCAGTGTTGTGCAGAGGCTGACAAGGAAAGCTGCCTGACCCCGAAGCTTGATGGTGTGAAGGAGAAAGCATTGGTCTCATCTGTCCGTCAG
AGAATGAAGTGCTCCAGTATGCAGAAGTTTGGAGAGAGAGCTTTTAAAGCATGGGCAGTAGCTCGTCTGAGCCAGACATTCCCCAATGCTGACTTTGCAGA
AATCACCAAATTGGCAACAGACCTGACCAAAGTCAACAAGGAGTGCTGCCATGGTGACCTGCTGGAATGCGCAGATGACAGGGCGGAACTTGCCAAGTACA
TGTGTGAAAACCAGGCGACTATCTCCAGCAAACTGCAGACTTGCTGCGATAAACCACTGTTGAAGAAAGCCCACTGTCTTAGTGAGGTGGAGCATGACACC
ATGCCTGCTGATCTGCCTGCCATTGCTGCTGATTTTGTTGAGGACCAGGAAGTGTGCAAGAACTATGCTGAGGCCAAGGATGTCTTCCTGGGCACGTTCTT
GTATGAATATTCAAGAAGACACCCTGATTACTCTGTATCCCTGTTGCTGAGACTTGCTAAGAAATATGAAGCCACTCTGGAAAAGTGCTGCGCTGAAGCCA
ATCCTCCCGCATGCTACGGCACAGTGCTTGCTGAATTTCAGCCTCTTGTAGAAGAGCCTAAGAACTTGGTCAAAACCAACTGTGATCTTTACGAGAAGCTT
GGAGAATATGGATTCCAAAATGCCATTCTAGTTCGCTACACCCAGAAAGCACCTCAGGTGTCAACCCCAACTCTCGTGGAGGCTGCAAGAAACCTAGGAAG
AGTGGGCACCAAGTGTTGTACACTTCCTGAAGATCAGAGACTGCCTTGTGTGGAAGACTATCTGTCTGCAATCCTGAACCGTGTGTGTCTGCTGCATGAGA
AGACCCCAGTGAGTGAGCATGTTACCAAGTGCTGTAGTGGATCCCTGGTGGAAAGGCGGCCATGCTTCTCTGCTCTGACAGTTGATGAAACATATGTCCCC
AAAGAGTTTAAAGCTGAGACCTTCACCTTCCACTCTGATATCTGCACACTTCCAGAGAAGGAGAAGCAGATTAAGAAACAAACGGCTCTTGCTGAGCTGGT
GAAGCACAAGCCCAAGGCTACAGCGGAGCAACTGAAGACTGTCATGGATGACTTTGCACAGTTCCTGGATACATGTTGCAAGGCTGCTGACAAGGACACCT
GCTTCTCGACTGAGGGTCCAAACCTTGTCACTAGATGCAAAGACGCCTTAGCC*GGAGGGGGCGGTTCC***CACCATCACCACCATCACTGATAA**

gWiz-LS-mCXCL11$^{22-100}$-(Gly$_4$Ser)$_2$-mouse SA-(Gly$_4$Ser)-His$_6$

ATGAGGGTCCCCGCTCAGCTCCTGGGGCTCCTGCTGCTCTGGCTCCCAGGTGCACGATGT**TTCCTGATGTTCAAGCAGGGCCGGTGCCTGTGCATCGGCCC**
TGGAATGAAGGCCGTGAAGATGGCCGAGATCGAGAAGGCCAGCGTGATCTACCCCAGCAACGGCTGCGACAAGGTGGAAGTGATCGTGACCATGAAGGCCC
ACAAGCGGCAGAGATGCCTGGACCCCAGATCCAAGCAGGCCCGGCTGATCATGCAGGCTATCGAGAAGAAGAATTTCCTGCGGCGGCAGAACATGGGTGGA
GGCGGTAGCGGAGGCGGAGGGTCGGAAGCACACAAGAGTGAGATCGCCCATCGGTATAATGATTTGGGAGAACAACATTTCAAAGGCCTAGTCCTGATTGC
CTTTTCCCAGTATCTCCAGAAATGCTCATACGATGAGCATGCCAAATTAGTGCAGGAAGTAACAGACTTTGCAAAGACGTGTGTTGCCGATGAGTCTGCCG
CCAACTGTGACAAATCCCTTCACACTCTTTTTGGAGATAAGTTGTGTGCCATTCCAAACCTCCGTGAAAACTATGGTGAACTGGCTGACTGCTGTACAAAA
CAAGAGCCCGAAAGAAACGAATGTTTCCTGCAACACAAAGATGACAACCCCAGCCTGCCACCATTTGAAAGGCCAGAGGCTGAGGCCATGTGCACCTCCTT
TAAGGAAAACCCAACCACCTTTATGGGACACTATTTGCATGAAGTTGCCAGAAGACATCCTTATTTCTATGCCCCAGAACTTCTTTACTATGCTGAGCAGT
ACAATGAGATTCTGACCCAGTGTTGTGCAGAGGCTGACAAGGAAAGCTGCCTGACCCCGAAGCTTGATGGTGTGAAGGAGAAAGCATTGGTCTCATCTGTC

CGTCAGAGAATGAAGTGCTCCAGTATGCAGAAGTTTGGAGAGAGAGCTTTTAAAGCATGGGCAGTAGCTCGTCTGAGCCAGACATTCCCCAATGCTGACTT
TGCAGAAATCACCAAATTGGCAACAGACCTGACCAAAGTCAACAAGGAGTGCTGCCATGGTGACCTGCTGGAATGCGCAGATGACAGGGCGGAACTTGCCA
AGTACATGTGTGAAAACCAGGCGACTATCTCCAGCAAACTGCAGACTTGCTGCGATAAACCACTGTTGAAGAAAGCCCACTGTCTTAGTGAGGTGGAGCAT
GACACCATGCCTGCTGATCTGCCTGCCATTGCTGCTGATTTTGTTGAGGACCAGGAAGTGTGCAAGAACTATGCTGAGGCCAAGGATGTCTTCCTGGGCAC
GTTCTTGTATGAATATTCAAGAAGACACCCTGATTACTCTGTATCCCTGTTGCTGAGACTTGCTAAGAAATATGAAGCCACTCTGGAAAAGTGCTGCGCTG
AAGCCAATCCTCCCGCATGCTACGGCACAGTGCTTGCTGAATTTCAGCCTCTTGTAGAAGAGCCTAAGAACTTGGTCAAAACCAACTGTGATCTTTACGAG
AAGCTTGGAGAATATGGATTCCAAAATGCCATTCTAGTTCGCTACACCCAGAAAGCACCTCAGGTGTCAACCCCAACTCTCGTGGAGGCTGCAAGAAACCT
AGGAAGAGTGGGCACCAAGTGTTGTACACTTCCTGAAGATCAGAGACTGCCTTGTGTGGAAGACTATCTGTCTGCAATCCTGAACCGTGTGTGTCTGCTGC
ATGAGAAGACCCCAGTGAGTGAGCATGTTACCAAGTGCTGTAGTGGATCCCTGGTGGAAAGGCGGCCATGCTTCTCTGCTCTGACAGTTGATGAAACATAT
GTCCCCAAAGAGTTTAAAGCTGAGACCTTCACCTTCCACTCTGATATCTGCACACTTCCAGAGAAGGAGAAGCAGATTAAGAAACAAACGGCTCTTGCTGA
GCTGGTGAAGCACAAGCCCAAGGCTACAGCGGAGCAACTGAAGACTGTCATGGATGACTTTGCACAGTTCCTGGATACATGTTGCAAGGCTGCTGACAAGG
ACACCTGCTTCTCGACTGAGGGTCCAAACCTTGTCACTAGATGCAAAGACGCCTTAGCC*GGAGGGGGCGGTTCC***CACCATCACCACCATCACTGATAA**

Amino-acid sequences of CXCL chemokines fused to the N-terminus of mouse serum albumin (^{N}CXCL-SA^{C})

Amino acid sequences of translated polypeptides are shown. The sequences include the secretory leader peptide (LS, in grey underlined), an active form of CXCL (in blue bold), a decapeptide $(Gly_4Ser)_2$ flexible linker (in green bold), mouse serum albumin (SA, in grey), a pentapeptide (Gly_4Ser) flexible linker (in black italic and underlined) and the hexa-histidine tag (His_6, in red bold).

LS-hCXCL1$^{35\text{-}107}$-$(Gly_4Ser)_2$-mouse SA-(Gly_4Ser)-His_6

MRVPAQLLGLLLLWLPGARC**ASVATELRCQCLQTLQGIHPKNIQSVNVKSPGPHCAQTEVIATLKNGRKACLNPASPIVKKIIEKMLNSDKSNGGGGSGGG**
GSEAHKSEIAHRYNDLGEQHFKGLVLIAFSQYLQKCSYDEHAKLVQEVTDFAKTCVADESAANCDKSLHTLFGDKLCAIPNLRENYGELADCCTKQEPERN
ECFLQHKDDNPSLPPFERPEAEAMCTSFKENPTTFMGHYLHEVARRHPYFYAPELLYYAEQYNEILTQCCAEADKESCLTPKLDGVKEKALVSSVRQRMKC
SSMQKFGERAFKAWAVARLSQTFPNADFAEITKLATDLTKVNKECCHGDLLECADDRAELAKYMCENQATISSKLQTCCDKPLLKKAHCLSEVEHDTMPAD
LPAIAADFVEDQEVCKNYAEAKDVFLGTFLYEYSRRHPDYSVSLLLRLAKKYEATLEKCCAEANPPACYGTVLAEFQPLVEEPKNLVKTNCDLYEKLGEYG
FQNAILVRYTQKAPQVSTPTLVEAARNLGRVGTKCCTLPEDQRLPCVEDYLSAILNRVCLLHEKTPVSEHVTKCCSGSLVERRPCFSALTVDETYVPKEFK
AETFTFHSDICTLPEKEKQIKKQTALAELVKHKPKATAEQLKTVMDDFAQFLDTCCKAADKDTCFSTEGPNLVTRCKDALA*GGGGS***HHHHHH**--

LS-hCXCL2$^{35\text{-}107}$-$(Gly_4Ser)_2$-mouse SA-(Gly_4Ser)-His_6

MRVPAQLLGLLLLWLPGARC**APLATELRCQCLQTLQGIHLKNIQSVKVKSPGPHCAQTEVIATLKNGQKACLNPASPMVKKIIEKMLKNGKSNGGGGSGGG**
GSEAHKSEIAHRYNDLGEQHFKGLVLIAFSQYLQKCSYDEHAKLVQEVTDFAKTCVADESAANCDKSLHTLFGDKLCAIPNLRENYGELADCCTKQEPERN
ECFLQHKDDNPSLPPFERPEAEAMCTSFKENPTTFMGHYLHEVARRHPYFYAPELLYYAEQYNEILTQCCAEADKESCLTPKLDGVKEKALVSSVRQRMKC
SSMQKFGERAFKAWAVARLSQTFPNADFAEITKLATDLTKVNKECCHGDLLECADDRAELAKYMCENQATISSKLQTCCDKPLLKKAHCLSEVEHDTMPAD
LPAIAADFVEDQEVCKNYAEAKDVFLGTFLYEYSRRHPDYSVSLLLRLAKKYEATLEKCCAEANPPACYGTVLAEFQPLVEEPKNLVKTNCDLYEKLGEYG
FQNAILVRYTQKAPQVSTPTLVEAARNLGRVGTKCCTLPEDQRLPCVEDYLSAILNRVCLLHEKTPVSEHVTKCCSGSLVERRPCFSALTVDETYVPKEFK
AETFTFHSDICTLPEKEKQIKKQTALAELVKHKPKATAEQLKTVMDDFAQFLDTCCKAADKDTCFSTEGPNLVTRCKDALA*GGGGS***HHHHHH**--

LS-hCXCL3$^{35\text{-}107}$-$(Gly_4Ser)_2$-mouse SA-(Gly_4Ser)-His_6

MRVPAQLLGLLLLWLPGARC**ASVVTELRCQCLQTLQGIHLKNIQSVNVRSPGPHCAQTEVIATLKNGKKACLNPASPMVQKIIEKILNKGSTNGGGGSGGG**
GSEAHKSEIAHRYNDLGEQHFKGLVLIAFSQYLQKCSYDEHAKLVQEVTDFAKTCVADESAANCDKSLHTLFGDKLCAIPNLRENYGELADCCTKQEPERN

ECFLQHKDDNPSLPPFERPEAEAMCTSFKENPTTFMGHYLHEVARRHPYFYAPELLYYAEQYNEILTQCCAEADKESCLTPKLDGVKEKALVSSVRQRMKC
SSMQKFGERAFKAWAVARLSQTFPNADFAEITKLATDLTKVNKECCHGDLLECADDRAELAKYMCENQATISSKLQTCCDKPLLKKAHCLSEVEHDTMPAD
LPAIAADFVEDQEVCKNYAEAKDVFLGTFLYEYSRRHPDYSVSLLLRLAKKYEATLEKCCAEANPPACYGTVLAEFQPLVEEPKNLVKTNCDLYEKLGEYG
FQNAILVRYTQKAPQVSTPTLVEAARNLGRVGTKCCTLPEDQRLPCVEDYLSAILNRVCLLHEKTPVSEHVTKCCSGSLVERRPCFSALTVDETYVPKEFK
AETFTFHSDICTLPEKEKQIKKQTALAELVKHKPKATAEQLKTVMDDFAQFLDTCCKAADKDTCFSTEGPNLVTRCKDALA*GGGGS***HHHHHH**--

LS-hCXCL4$^{32\text{-}101}$-(Gly$_4$Ser)$_2$-mouse SA-(Gly$_4$Ser)-His$_6$

MRVPAQLLGLLLLWLPGARC**EAEEDGDLQCLCVKTTSQVRPRHITSLEVIKAGPHCPTAQLIATLKNGRKICLDLQAPLYKKIIKKLLESGGGGSGGGGS**E
AHKSEIAHRYNDLGEQHFKGLVLIAFSQYLQKCSYDEHAKLVQEVTDFAKTCVADESAANCDKSLHTLFGDKLCAIPNLRENYGELADCCTKQEPERNECF
LQHKDDNPSLPPFERPEAEAMCTSFKENPTTFMGHYLHEVARRHPYFYAPELLYYAEQYNEILTQCCAEADKESCLTPKLDGVKEKALVSSVRQRMKCSSM
QKFGERAFKAWAVARLSQTFPNADFAEITKLATDLTKVNKECCHGDLLECADDRAELAKYMCENQATISSKLQTCCDKPLLKKAHCLSEVEHDTMPADLPA
IAADFVEDQEVCKNYAEAKDVFLGTFLYEYSRRHPDYSVSLLLRLAKKYEATLEKCCAEANPPACYGTVLAEFQPLVEEPKNLVKTNCDLYEKLGEYGFQN
AILVRYTQKAPQVSTPTLVEAARNLGRVGTKCCTLPEDQRLPCVEDYLSAILNRVCLLHEKTPVSEHVTKCCSGSLVERRPCFSALTVDETYVPKEFKAET
FTFHSDICTLPEKEKQIKKQTALAELVKHKPKATAEQLKTVMDDFAQFLDTCCKAADKDTCFSTEGPNLVTRCKDALA*GGGGS***HHHHHH**--

LS-hCXCL5$^{44\text{-}114}$-(Gly$_4$Ser)$_2$-mouse SA-(Gly$_4$Ser)-His$_6$

MRVPAQLLGLLLLWLPGARC**LRELRCVCLQTTQGVHPKMISNLQVFAIGPQCSKVEVVASLKNGKEICLDPEAPFLKKVIQKILDGGNKENGGGGSGGGGS**
EAHKSEIAHRYNDLGEQHFKGLVLIAFSQYLQKCSYDEHAKLVQEVTDFAKTCVADESAANCDKSLHTLFGDKLCAIPNLRENYGELADCCTKQEPERNEC
FLQHKDDNPSLPPFERPEAEAMCTSFKENPTTFMGHYLHEVARRHPYFYAPELLYYAEQYNEILTQCCAEADKESCLTPKLDGVKEKALVSSVRQRMKCSS
MQKFGERAFKAWAVARLSQTFPNADFAEITKLATDLTKVNKECCHGDLLECADDRAELAKYMCENQATISSKLQTCCDKPLLKKAHCLSEVEHDTMPADLP
AIAADFVEDQEVCKNYAEAKDVFLGTFLYEYSRRHPDYSVSLLLRLAKKYEATLEKCCAEANPPACYGTVLAEFQPLVEEPKNLVKTNCDLYEKLGEYGFQ
NAILVRYTQKAPQVSTPTLVEAARNLGRVGTKCCTLPEDQRLPCVEDYLSAILNRVCLLHEKTPVSEHVTKCCSGSLVERRPCFSALTVDETYVPKEFKAE
TFTFHSDICTLPEKEKQIKKQTALAELVKHKPKATAEQLKTVMDDFAQFLDTCCKAADKDTCFSTEGPNLVTRCKDALA*GGGGS***HHHHHH**--

LS-hCXCL6$^{43\text{-}114}$-(Gly$_4$Ser)$_2$-mouse SA-(Gly$_4$Ser)-His$_6$

MRVPAQLLGLLLLWLPGARC**VLTELRCTCLRVTLRVNPKTIGKLQVFPAGPQCSKVEVVASLKNGKQVCLDPEAPFLKKVIQKILDSGNKKNGGGGSGGGG**
SEAHKSEIAHRYNDLGEQHFKGLVLIAFSQYLQKCSYDEHAKLVQEVTDFAKTCVADESAANCDKSLHTLFGDKLCAIPNLRENYGELADCCTKQEPERNE
CFLQHKDDNPSLPPFERPEAEAMCTSFKENPTTFMGHYLHEVARRHPYFYAPELLYYAEQYNEILTQCCAEADKESCLTPKLDGVKEKALVSSVRQRMKCS
SMQKFGERAFKAWAVARLSQTFPNADFAEITKLATDLTKVNKECCHGDLLECADDRAELAKYMCENQATISSKLQTCCDKPLLKKAHCLSEVEHDTMPADL
PAIAADFVEDQEVCKNYAEAKDVFLGTFLYEYSRRHPDYSVSLLLRLAKKYEATLEKCCAEANPPACYGTVLAEFQPLVEEPKNLVKTNCDLYEKLGEYGF
QNAILVRYTQKAPQVSTPTLVEAARNLGRVGTKCCTLPEDQRLPCVEDYLSAILNRVCLLHEKTPVSEHVTKCCSGSLVERRPCFSALTVDETYVPKEFKA
ETFTFHSDICTLPEKEKQIKKQTALAELVKHKPKATAEQLKTVMDDFAQFLDTCCKAADKDTCFSTEGPNLVTRCKDALA*GGGGS***HHHHHH**--

LS-hCXCL7$^{59-121}$*-(Gly*$_4$*Ser)*$_2$*-mouse SA-(Gly*$_4$*Ser)-His*$_6$

MRVPAQLLGLLLLWLPGARC**AELRCMCIKTTSGIHPKNIQSLEVIGKGTHCNQVEVIATLKDGRKICLDPDAPRIKKIVQKKLGGGGSGGGGS**EAHKSEIA
HRYNDLGEQHFKGLVLIAFSQYLQKCSYDEHAKLVQEVTDFAKTCVADESAANCDKSLHTLFGDKLCAIPNLRENYGELADCCTKQEPERNECFLQHKDDN
PSLPPFERPEAEAMCTSFKENPTTFMGHYLHEVARRHPYFYAPELLYYAEQYNEILTQCCAEADKESCLTPKLDGVKEKALVSSVRQRMKCSSMQKFGERA
FKAWAVARLSQTFPNADFAEITKLATDLTKVNKECCHGDLLECADDRAELAKYMCENQATISSKLQTCCDKPLLKKAHCLSEVEHDTMPADLPAIAADFVE
DQEVCKNYAEAKDVFLGTFLYEYSRRHPDYSVSLLLRLAKKYEATLEKCCAEANPPACYGTVLAEFQPLVEEPKNLVKTNCDLYEKLGEYGFQNAILVRYT
QKAPQVSTPTLVEAARNLGRVGTKCCTLPEDQRLPCVEDYLSAILNRVCLLHEKTPVSEHVTKCCSGSLVERRPCFSALTVDETYVPKEFKAETFTFHSDI
CTLPEKEKQIKKQTALAELVKHKPKATAEQLKTVMDDFAQFLDTCCKAADKDTCFSTEGPNLVTRCKDALA*GGGGS***HHHHHH**--

LS-hCXCL8$^{28-99}$*-(Gly*$_4$*Ser)*$_2$*-mouse SA-(Gly*$_4$*Ser)-His*$_6$

MRVPAQLLGLLLLWLPGARC**SAKELRCQCIKTYSKPFHPKFIKELRVIESGPHCANTEIIVKLSDGRELCLDPKENWVQRVVEKFLKRAENSGGGGSGGGG**
SEAHKSEIAHRYNDLGEQHFKGLVLIAFSQYLQKCSYDEHAKLVQEVTDFAKTCVADESAANCDKSLHTLFGDKLCAIPNLRENYGELADCCTKQEPERNE
CFLQHKDDNPSLPPFERPEAEAMCTSFKENPTTFMGHYLHEVARRHPYFYAPELLYYAEQYNEILTQCCAEADKESCLTPKLDGVKEKALVSSVRQRMKCS
SMQKFGERAFKAWAVARLSQTFPNADFAEITKLATDLTKVNKECCHGDLLECADDRAELAKYMCENQATISSKLQTCCDKPLLKKAHCLSEVEHDTMPADL
PAIAADFVEDQEVCKNYAEAKDVFLGTFLYEYSRRHPDYSVSLLLRLAKKYEATLEKCCAEANPPACYGTVLAEFQPLVEEPKNLVKTNCDLYEKLGEYGF
QNAILVRYTQKAPQVSTPTLVEAARNLGRVGTKCCTLPEDQRLPCVEDYLSAILNRVCLLHEKTPVSEHVTKCCSGSLVERRPCFSALTVDETYVPKEFKA
ETFTFHSDICTLPEKEKQIKKQTALAELVKHKPKATAEQLKTVMDDFAQFLDTCCKAADKDTCFSTEGPNLVTRCKDALA*GGGGS***HHHHHH**--

LS-hCXCL9$^{23-115}$*-(Gly*$_4$*Ser)*$_2$*-mouse SA-(Gly*$_4$*Ser)-His*$_6$

MRVPAQLLGLLLLWLPGARC**TPVVRKGRCSCISTNQGTIHLQSLKDLKQFAPSPSCEKIEIIATLKNGVQTCLNPDSADVKELIKKWEKQVSQKKKQNGK**
KHQKKKVLKVRKSQRSRQKKTTGGGGSGGGGSEAHKSEIAHRYNDLGEQHFKGLVLIAFSQYLQKCSYDEHAKLVQEVTDFAKTCVADESAANCDKSLHTL
FGDKLCAIPNLRENYGELADCCTKQEPERNECFLQHKDDNPSLPPFERPEAEAMCTSFKENPTTFMGHYLHEVARRHPYFYAPELLYYAEQYNEILTQCCA
EADKESCLTPKLDGVKEKALVSSVRQRMKCSSMQKFGERAFKAWAVARLSQTFPNADFAEITKLATDLTKVNKECCHGDLLECADDRAELAKYMCENQATI
SSKLQTCCDKPLLKKAHCLSEVEHDTMPADLPAIAADFVEDQEVCKNYAEAKDVFLGTFLYEYSRRHPDYSVSLLLRLAKKYEATLEKCCAEANPPACYGT
VLAEFQPLVEEPKNLVKTNCDLYEKLGEYGFQNAILVRYTQKAPQVSTPTLVEAARNLGRVGTKCCTLPEDQRLPCVEDYLSAILNRVCLLHEKTPVSEHV
TKCCSGSLVERRPCFSALTVDETYVPKEFKAETFTFHSDICTLPEKEKQIKKQTALAELVKHKPKATAEQLKTVMDDFAQFLDTCCKAADKDTCFSTEGPN
LVTRCKDALA*GGGGS***HHHHHH**--

LS-hCXCL10$^{22-98}$*-(Gly*$_4$*Ser)*$_2$*-mouse SA-(Gly*$_4$*Ser)-His*$_6$

MRVPAQLLGLLLLWLPGARC**VPLSRTVRCTCISISNQPVNPRSLEKLEIIPASQFCPRVEIIATMKKKGEKRCLNPESKAIKNLLKAVSKERSKRSPGGGG**
SGGGGSEAHKSEIAHRYNDLGEQHFKGLVLIAFSQYLQKCSYDEHAKLVQEVTDFAKTCVADESAANCDKSLHTLFGDKLCAIPNLRENYGELADCCTKQE
PERNECFLQHKDDNPSLPPFERPEAEAMCTSFKENPTTFMGHYLHEVARRHPYFYAPELLYYAEQYNEILTQCCAEADKESCLTPKLDGVKEKALVSSVRQ

RMKCSSMQKFGERAFKAWAVARLSQTFPNADFAEITKLATDLTKVNKECCHGDLLECADDRAELAKYMCENQATISSKLQTCCDKPLLKKAHCLSEVEHDT
MPADLPAIAADFVEDQEVCKNYAEAKDVFLGTFLYEYSRRHPDYSVSLLLRLAKKYEATLEKCCAEANPPACYGTVLAEFQPLVEEPKNLVKTNCDLYEKL
GEYGFQNAILVRYTQKAPQVSTPTLVEAARNLGRVGTKCCTLPEDQRLPCVEDYLSAILNRVCLLHEKTPVSEHVTKCCSGSLVERRPCFSALTVDETYVP
KEFKAETFTFHSDICTLPEKEKQIKKQTALAELVKHKPKATAEQLKTVMDDFAQFLDTCCKAADKDTCFSTEGPNLVTRCKDALA*GGGGS***HHHHHH**--

LS-hCXCL11$^{22-94}$*-(Gly*$_4$*Ser)*$_2$*-mouse SA-(Gly*$_4$*Ser)-His*$_6$

MRVPAQLLGLLLLWLPGARC**FPMFKRGRCLCIGPGVKAVKVADIEKASIMYPSNNCDKIEVIITLKENKGQRCLNPKSKQARLIIKKVERKNFGGGGSGGG**
GSEAHKSEIAHRYNDLGEQHFKGLVLIAFSQYLQKCSYDEHAKLVQEVTDFAKTCVADESAANCDKSLHTLFGDKLCAIPNLRENYGELADCCTKQEPERN
ECFLQHKDDNPSLPPFERPEAEAMCTSFKENPTTFMGHYLHEVARRHPYFYAPELLYYAEQYNEILTQCCAEADKESCLTPKLDGVKEKALVSSVRQRMKC
SSMQKFGERAFKAWAVARLSQTFPNADFAEITKLATDLTKVNKECCHGDLLECADDRAELAKYMCENQATISSKLQTCCDKPLLKKAHCLSEVEHDTMPAD
LPAIAADFVEDQEVCKNYAEAKDVFLGTFLYEYSRRHPDYSVSLLLRLAKKYEATLEKCCAEANPPACYGTVLAEFQPLVEEPKNLVKTNCDLYEKLGEYG
FQNAILVRYTQKAPQVSTPTLVEAARNLGRVGTKCCTLPEDQRLPCVEDYLSAILNRVCLLHEKTPVSEHVTKCCSGSLVERRPCFSALTVDETYVPKEFK
AETFTFHSDICTLPEKEKQIKKQTALAELVKHKPKATAEQLKTVMDDFAQFLDTCCKAADKDTCFSTEGPNLVTRCKDALA*GGGGS***HHHHHH**--

LS-mCXCL1$^{25-96}$*-(Gly*$_4$*Ser)*$_2$*-mouse SA-(Gly*$_4$*Ser)-His*$_6$

MRVPAQLLGLLLLWLPGARC**APIANELRCQCLQTMAGIHLKNIQSLKVLPSGPHCTQTEVIATLKNGREACLDPEAPLVQKIVQKMLKGVPKGGGGSGGGG**
SEAHKSEIAHRYNDLGEQHFKGLVLIAFSQYLQKCSYDEHAKLVQEVTDFAKTCVADESAANCDKSLHTLFGDKLCAIPNLRENYGELADCCTKQEPERNE
CFLQHKDDNPSLPPFERPEAEAMCTSFKENPTTFMGHYLHEVARRHPYFYAPELLYYAEQYNEILTQCCAEADKESCLTPKLDGVKEKALVSSVRQRMKCS
SMQKFGERAFKAWAVARLSQTFPNADFAEITKLATDLTKVNKECCHGDLLECADDRAELAKYMCENQATISSKLQTCCDKPLLKKAHCLSEVEHDTMPADL
PAIAADFVEDQEVCKNYAEAKDVFLGTFLYEYSRRHPDYSVSLLLRLAKKYEATLEKCCAEANPPACYGTVLAEFQPLVEEPKNLVKTNCDLYEKLGEYGF
QNAILVRYTQKAPQVSTPTLVEAARNLGRVGTKCCTLPEDQRLPCVEDYLSAILNRVCLLHEKTPVSEHVTKCCSGSLVERRPCFSALTVDETYVPKEFKA
ETFTFHSDICTLPEKEKQIKKQTALAELVKHKPKATAEQLKTVMDDFAQFLDTCCKAADKDTCFSTEGPNLVTRCKDALA*GGGGS***HHHHHH**--

LS-mCXCL2$^{28-100}$*-(Gly*$_4$*Ser)*$_2$*-mouse SA-(Gly*$_4$*Ser)-His*$_6$

MRVPAQLLGLLLLWLPGARC**AVVASELRCQCLKTLPRVDFKNIQSLSVTPPGPHCAQTEVIATLKGGQKVCLDPEAPLVQKIIQKILNKGKANGGGGSGGG**
GSEAHKSEIAHRYNDLGEQHFKGLVLIAFSQYLQKCSYDEHAKLVQEVTDFAKTCVADESAANCDKSLHTLFGDKLCAIPNLRENYGELADCCTKQEPERN
ECFLQHKDDNPSLPPFERPEAEAMCTSFKENPTTFMGHYLHEVARRHPYFYAPELLYYAEQYNEILTQCCAEADKESCLTPKLDGVKEKALVSSVRQRMKC
SSMQKFGERAFKAWAVARLSQTFPNADFAEITKLATDLTKVNKECCHGDLLECADDRAELAKYMCENQATISSKLQTCCDKPLLKKAHCLSEVEHDTMPAD
LPAIAADFVEDQEVCKNYAEAKDVFLGTFLYEYSRRHPDYSVSLLLRLAKKYEATLEKCCAEANPPACYGTVLAEFQPLVEEPKNLVKTNCDLYEKLGEYG
FQNAILVRYTQKAPQVSTPTLVEAARNLGRVGTKCCTLPEDQRLPCVEDYLSAILNRVCLLHEKTPVSEHVTKCCSGSLVERRPCFSALTVDETYVPKEFK
AETFTFHSDICTLPEKEKQIKKQTALAELVKHKPKATAEQLKTVMDDFAQFLDTCCKAADKDTCFSTEGPNLVTRCKDALA*GGGGS***HHHHHH**--

LS-mCXCL3$^{28-100}$-$(Gly_4Ser)_2$-mouse SA-(Gly_4Ser)-His_6

MRVPAQLLGLLLLWLPGARC**AVVASELRCQCLNTLPRVDFETIQSLTVTPPGPHCTQTEVIATLKDGQEVCLNPQGPRLQIIIKKILKSGKSSGGGGSGGG**
GSEAHKSEIAHRYNDLGEQHFKGLVLIAFSQYLQKCSYDEHAKLVQEVTDFAKTCVADESAANCDKSLHTLFGDKLCAIPNLRENYGELADCCTKQEPERN
ECFLQHKDDNPSLPPFERPEAEAMCTSFKENPTTFMGHYLHEVARRHPYFYAPELLYYAEQYNEILTQCCAEADKESCLTPKLDGVKEKALVSSVRQRMKC
SSMQKFGERAFKAWAVARLSQTFPNADFAEITKLATDLTKVNKECCHGDLLECADDRAELAKYMCENQATISSKLQTCCDKPLLKKAHCLSEVEHDTMPAD
LPAIAADFVEDQEVCKNYAEAKDVFLGTFLYEYSRRHPDYSVSLLLRLAKKYEATLEKCCAEANPPACYGTVLAEFQPLVEEPKNLVKTNCDLYEKLGEYG
FQNAILVRYTQKAPQVSTPTLVEAARNLGRVGTKCCTLPEDQRLPCVEDYLSAILNRVCLLHEKTPVSEHVTKCCSGSLVERRPCFSALTVDETYVPKEFK
AETFTFHSDICTLPEKEKQIKKQTALAELVKHKPKATAEQLKTVMDDFAQFLDTCCKAADKDTCFSTEGPNLVTRCKDALA*GGGGS***HHHHHH**--

LS-mCXCL4$^{30-105}$-$(Gly_4Ser)_2$-mouse SA-(Gly_4Ser)-His_6

MRVPAQLLGLLLLWLPGARC**VTSAGPEESDGDLSCVCVKTISSGIHLKHITSLEVIKAGRHCAVPQLIATLKNGRKICLDRQAPLYKKVIKKILESGGGGS**
GGGGSEAHKSEIAHRYNDLGEQHFKGLVLIAFSQYLQKCSYDEHAKLVQEVTDFAKTCVADESAANCDKSLHTLFGDKLCAIPNLRENYGELADCCTKQEP
ERNECFLQHKDDNPSLPPFERPEAEAMCTSFKENPTTFMGHYLHEVARRHPYFYAPELLYYAEQYNEILTQCCAEADKESCLTPKLDGVKEKALVSSVRQR
MKCSSMQKFGERAFKAWAVARLSQTFPNADFAEITKLATDLTKVNKECCHGDLLECADDRAELAKYMCENQATISSKLQTCCDKPLLKKAHCLSEVEHDTM
PADLPAIAADFVEDQEVCKNYAEAKDVFLGTFLYEYSRRHPDYSVSLLLRLAKKYEATLEKCCAEANPPACYGTVLAEFQPLVEEPKNLVKTNCDLYEKLG
EYGFQNAILVRYTQKAPQVSTPTLVEAARNLGRVGTKCCTLPEDQRLPCVEDYLSAILNRVCLLHEKTPVSEHVTKCCSGSLVERRPCFSALTVDETYVPK
EFKAETFTFHSDICTLPEKEKQIKKQTALAELVKHKPKATAEQLKTVMDDFAQFLDTCCKAADKDTCFSTEGPNLVTRCKDALA*GGGGS***HHHHHH**--

LS-mCXCL5$^{48-118}$-$(Gly_4Ser)_2$-mouse SA-(Gly_4Ser)-His_6

MRVPAQLLGLLLLWLPGARC**ATELRCVCLTVTPKINPKLIANLEVIPAGPQCPTVEVIAKLKNQKEVCLDPEAPVIKKIIQKILGSDKKKAGGGGSGGGGS**
EAHKSEIAHRYNDLGEQHFKGLVLIAFSQYLQKCSYDEHAKLVQEVTDFAKTCVADESAANCDKSLHTLFGDKLCAIPNLRENYGELADCCTKQEPERNEC
FLQHKDDNPSLPPFERPEAEAMCTSFKENPTTFMGHYLHEVARRHPYFYAPELLYYAEQYNEILTQCCAEADKESCLTPKLDGVKEKALVSSVRQRMKCSS
MQKFGERAFKAWAVARLSQTFPNADFAEITKLATDLTKVNKECCHGDLLECADDRAELAKYMCENQATISSKLQTCCDKPLLKKAHCLSEVEHDTMPADLP
AIAADFVEDQEVCKNYAEAKDVFLGTFLYEYSRRHPDYSVSLLLRLAKKYEATLEKCCAEANPPACYGTVLAEFQPLVEEPKNLVKTNCDLYEKLGEYGFQ
NAILVRYTQKAPQVSTPTLVEAARNLGRVGTKCCTLPEDQRLPCVEDYLSAILNRVCLLHEKTPVSEHVTKCCSGSLVERRPCFSALTVDETYVPKEFKAE
TFTFHSDICTLPEKEKQIKKQTALAELVKHKPKATAEQLKTVMDDFAQFLDTCCKAADKDTCFSTEGPNLVTRCKDALA*GGGGS***HHHHHH**--

LS-mCXCL7$^{48-113}$-$(Gly_4Ser)_2$-mouse SA-(Gly_4Ser)-His_6

MRVPAQLLGLLLLWLPGARC**IELRCRCTNTISGIPFNSISLVNVYRPGVHCADVEVIATLKNGQKTCLDPNAPGVKRIVMKILEGYGGGGSGGGGS**EAHKS
EIAHRYNDLGEQHFKGLVLIAFSQYLQKCSYDEHAKLVQEVTDFAKTCVADESAANCDKSLHTLFGDKLCAIPNLRENYGELADCCTKQEPERNECFLQHK
DDNPSLPPFERPEAEAMCTSFKENPTTFMGHYLHEVARRHPYFYAPELLYYAEQYNEILTQCCAEADKESCLTPKLDGVKEKALVSSVRQRMKCSSMQKFG
ERAFKAWAVARLSQTFPNADFAEITKLATDLTKVNKECCHGDLLECADDRAELAKYMCENQATISSKLQTCCDKPLLKKAHCLSEVEHDTMPADLPAIAAD

FVEDQEVCKNYAEAKDVFLGTFLYEYSRRHPDYSVSLLLRLAKKYEATLEKCCAEANPPACYGTVLAEFQPLVEEPKNLVKTNCDLYEKLGEYGFQNAILV
RYTQKAPQVSTPTLVEAARNLGRVGTKCCTLPEDQRLPCVEDYLSAILNRVCLLHEKTPVSEHVTKCCSGSLVERRPCFSALTVDETYVPKEFKAETFTFH
SDICTLPEKEKQIKKQTALAELVKHKPKATAEQLKTVMDDFAQFLDTCCKAADKDTCFSTEGPNLVTRCKDALA*GGGGS***HHHHHH**--

LS-mCXCL9$^{22\text{-}126}$*-(Gly*$_4$*Ser)*$_2$*-mouse SA-(Gly*$_4$*Ser)-His*$_6$

MRVPAQLLGLLLLWLPGARC**TLVIRNARCSCISTSRGTIHYKSLKDLKQFAPSPNCNKTEIIATLKNGDQTCLDPDSANVKKLMKEWEKKISQKKKQKRGK**
KHQKNMKNRKPKTPQSRRRSRKTTGGGGSGGGGSEAHKSEIAHRYNDLGEQHFKGLVLIAFSQYLQKCSYDEHAKLVQEVTDFAKTCVADESAANCDKSLH
TLFGDKLCAIPNLRENYGELADCCTKQEPERNECFLQHKDDNPSLPPFERPEAEAMCTSFKENPTTFMGHYLHEVARRHPYFYAPELLYYAEQYNEILTQC
CAEADKESCLTPKLDGVKEKALVSSVRQRMKCSSMQKFGERAFKAWAVARLSQTFPNADFAEITKLATDLTKVNKECCHGDLLECADDRAELAKYMCENQA
TISSKLQTCCDKPLLKKAHCLSEVEHDTMPADLPAIAADFVEDQEVCKNYAEAKDVFLGTFLYEYSRRHPDYSVSLLLRLAKKYEATLEKCCAEANPPACY
GTVLAEFQPLVEEPKNLVKTNCDLYEKLGEYGFQNAILVRYTQKAPQVSTPTLVEAARNLGRVGTKCCTLPEDQRLPCVEDYLSAILNRVCLLHEKTPVSE
HVTKCCSGSLVERRPCFSALTVDETYVPKEFKAETFTFHSDICTLPEKEKQIKKQTALAELVKHKPKATAEQLKTVMDDFAQFLDTCCKAADKDTCFSTEG
PNLVTRCKDALA*GGGGS***HHHHHH**--

LS-mCXCL10$^{22\text{-}98}$*-(Gly*$_4$*Ser)*$_2$*-mouse SA-(Gly*$_4$*Ser)-His*$_6$

MRVPAQLLGLLLLWLPGARC**IPLARTVRCNCIHIDDGPVRMRAIGKLEIIPASLSCPRVEIIATMKKNDEQRCLNPESKTIKNLMKAFSQKRSKRAPGGGG**
SGGGGSEAHKSEIAHRYNDLGEQHFKGLVLIAFSQYLQKCSYDEHAKLVQEVTDFAKTCVADESAANCDKSLHTLFGDKLCAIPNLRENYGELADCCTKQE
PERNECFLQHKDDNPSLPPFERPEAEAMCTSFKENPTTFMGHYLHEVARRHPYFYAPELLYYAEQYNEILTQCCAEADKESCLTPKLDGVKEKALVSSVRQ
RMKCSSMQKFGERAFKAWAVARLSQTFPNADFAEITKLATDLTKVNKECCHGDLLECADDRAELAKYMCENQATISSKLQTCCDKPLLKKAHCLSEVEHDT
MPADLPAIAADFVEDQEVCKNYAEAKDVFLGTFLYEYSRRHPDYSVSLLLRLAKKYEATLEKCCAEANPPACYGTVLAEFQPLVEEPKNLVKTNCDLYEKL
GEYGFQNAILVRYTQKAPQVSTPTLVEAARNLGRVGTKCCTLPEDQRLPCVEDYLSAILNRVCLLHEKTPVSEHVTKCCSGSLVERRPCFSALTVDETYVP
KEFKAETFTFHSDICTLPEKEKQIKKQTALAELVKHKPKATAEQLKTVMDDFAQFLDTCCKAADKDTCFSTEGPNLVTRCKDALA*GGGGS***HHHHHH**--

LS-mCXCL11$^{22\text{-}100}$*-(Gly*$_4$*Ser)*$_2$*-mouse SA-(Gly*$_4$*Ser)-His*$_6$

MRVPAQLLGLLLLWLPGARC**FLMFKQGRCLCIGPGMKAVKMAEIEKASVIYPSNGCDKVEVIVTMKAHKRQRCLDPRSKQARLIMQAIEKKNFLRRQNMGG**
GGSGGGGSEAHKSEIAHRYNDLGEQHFKGLVLIAFSQYLQKCSYDEHAKLVQEVTDFAKTCVADESAANCDKSLHTLFGDKLCAIPNLRENYGELADCCTK
QEPERNECFLQHKDDNPSLPPFERPEAEAMCTSFKENPTTFMGHYLHEVARRHPYFYAPELLYYAEQYNEILTQCCAEADKESCLTPKLDGVKEKALVSSV
RQRMKCSSMQKFGERAFKAWAVARLSQTFPNADFAEITKLATDLTKVNKECCHGDLLECADDRAELAKYMCENQATISSKLQTCCDKPLLKKAHCLSEVEH
DTMPADLPAIAADFVEDQEVCKNYAEAKDVFLGTFLYEYSRRHPDYSVSLLLRLAKKYEATLEKCCAEANPPACYGTVLAEFQPLVEEPKNLVKTNCDLYE
KLGEYGFQNAILVRYTQKAPQVSTPTLVEAARNLGRVGTKCCTLPEDQRLPCVEDYLSAILNRVCLLHEKTPVSEHVTKCCSGSLVERRPCFSALTVDETY
VPKEFKAETFTFHSDICTLPEKEKQIKKQTALAELVKHKPKATAEQLKTVMDDFAQFLDTCCKAADKDTCFSTEGPNLVTRCKDALA*GGGGS***HHHHHH**--
