# Supplementary material for: Directed evolution of broadly crossreactive chemokine-blocking antibodies efficacious in arthritis
Source: Nat Commun. 2018 Apr 13;9:1461. doi: 10.1038/s41467-018-03687-x (PMC5899157; doi:10.1038/s41467-018-03687-x)
Supplement: Supplementary file 6 — Supplementary Data 3 [file 41467_2018_3687_MOESM6_ESM.pdf]

Supplementary Data 3 - Protein accession numbers, oligonucleotide primers, DNA and amino-acid sequences of serum albumin-antibody fusion proteins (SA-scFv)

Fusion protein (code name)	Construct for expression
^{N}SA-CK138^{C} (SA138)	gWiz-LS-mouse SA-(Gly$_4$Ser)$_3$-scFv (V$_L$-V$_H$) CK138-(Gly$_4$Ser)-His$_6$
^{N}SA-CK157^{C} (SA157)	gWiz-LS-mouse SA-(Gly$_4$Ser)$_3$-scFv (V$_L$-V$_H$) CK157-(Gly$_4$Ser)-His$_6$
^{N}SA-CK129^{C} (SA129)	gWiz-LS-mouse SA-(Gly$_4$Ser)$_3$-scFv (V$_L$-V$_H$) CK129-(Gly$_4$Ser)-His$_6$
^{N}SA-CK138-ds1^{C} (SA138-ds1)	gWiz-LS-mouse SA-(Gly$_4$Ser)$_3$-scFv (V$_L$-V$_H$) CK138-ds1 (V$_L$100$^{Q>C}$ / V$_H$44$^{G>C}$)-(Gly$_4$Ser)-His$_6$
^{N}SA-CK138-ds2^{C} (SA138-ds2)	gWiz-LS-mouse SA-(Gly$_4$Ser)$_3$-scFv (V$_L$-V$_H$) CK138-ds2 (V$_L$43$^{A>C}$ / V$_H$105$^{Q>C}$)-(Gly$_4$Ser)-His$_6$
^{N}SA-CK157-ds1^{C} (SA157-ds1)	gWiz-LS-mouse SA-(Gly$_4$Ser)$_3$-scFv (V$_L$-V$_H$) CK157-ds1 (V$_L$100$^{Q>C}$ / V$_H$44$^{E>C}$)-(Gly$_4$Ser)-His$_6$
^{N}SA-CK157-ds2^{C} (SA157-ds2)	gWiz-LS-mouse SA-(Gly$_4$Ser)$_3$-scFv (V$_L$-V$_H$) CK157-ds2 (V$_L$43$^{A>C}$ / V$_H$105$^{Q>C}$)-(Gly$_4$Ser)-His$_6$
^{N}SA-CK157-VL^{C} (SA157-VL)	gWiz-LS-mouse SA-(Gly$_4$Ser)-V$_L$ CK157-His$_6$
^{N}SA-CK157-VH^{C} (SA157-VH)	gWiz-LS-mouse SA-(Gly$_4$Ser)-V$_H$ CK157-His$_6$
^{N}SA-CK129-ds1^{C} (SA129-ds1)	gWiz-LS-mouse SA-(Gly$_4$Ser)$_3$-scFv (V$_L$-V$_H$) CK129-ds1 (V$_L$100$^{Q>C}$ / V$_H$44$^{G>C}$)-(Gly$_4$Ser)-His$_6$
^{N}SA-CK129-ds2^{C} (SA129-ds2)	gWiz-LS-mouse SA-(Gly$_4$Ser)$_3$-scFv (V$_L$-V$_H$) CK129-ds2 (V$_L$43$^{A>C}$ / V$_H$105$^{Q>C}$)-(Gly$_4$Ser)-His$_6$
^{N}SA-sm3e-ds^{C} (SA^{CTR})	gWiz-LS-mouse SA-(Gly$_4$Ser)$_3$-scFv (V$_H$-V$_L$) sm3E-ds (V$_H$44$^{R>C}$ / V$_L$100$^{G>C}$)-(Gly$_4$Ser)-His$_6$

Primer name	Primer sequence (5' to 3')
Forward-linker-His$_6$-gWiz	5′-GGAGGGGGCGGTTCCC-3′
Reverse-LS-SA-linker-gWiz	5′-CGACCCTCCGCCTCCGC-3′
Forward-SA138 (scFv)	5′-GGAGGCGGAGGGTCGGCTAGCGCTATCCAGATGACCC-3′
Reverse-SA138 (scFv)	5′-GGAACCGCCCCCTCCCGAGGAGACGGTGACCAGG-3′
Forward-SA157 (scFv)	5′-GGAGGCGGAGGGTCGGCCAGCGATATCCAGATGACCCAGTC-3′
Reverse-SA157 (scFv)	5′-GGAACCGCCCCCTCCCGAGGAGACGGTGACCAGGG-3′
Forward-SA129 (scFv)	5′-GGAGGCGGAGGGTCGGCTAGCGATATCCAGATGACCCAG-3′
Reverse-SA129 (scFv)	5′-GGAACCGCCCCCTCCCGAGGAGACGGTGACCAGG-3′
Forward-Mut-SA138-ds1 (V_L100)	5′-CTGATCACGTTCGGATGCGGTACCAAGGTGGAGATCAAAGGTACTACTGC-3′
Reverse-Mut-SA138-ds1 (V_L100)	5′-TCCGAACGTGATCAGAGAATAAGAAGATTGCTGAC-3′
Forward-Mut-SA138-ds1 (V_H44)	5′-CAGGCCCCGGGTAAGTGCCTGGAATGGGTTGCATACATTGCTTCTTAC-3′
Reverse-Mut-SA138-ds1 (V_H44)	5′-CTTACCCGGGGCCTGACG-3′
Forward-Mut-SA138-ds2 (V_L43)	5′-CAGAAACCAGGAAAATGCCCGAAGCTTCTGATTTACGGTGCATCC-3′
Reverse-Mut-SA138-ds2 (V_L43)	5′-TTTTCCTGGTTTCTGTTGATACCAGGCTGC-3′
Forward-Mut-SA138-ds2 (V_H105)	5′-ATGAACTACTGGGGTTGCGGAGCCCTGGTCACCGTC-3′
Reverse-Mut-SA138-ds2 (V_H105)	5′-ACCCCAGTAGTTCATACCAGCAGAGAAC-3′
Forward-Mut-SA157-ds1 (V_L100)	5′-CTGATCACGTTCGGATGCGGTACCGAGGTGGAGATCAAAGGTACTACTGC -3′

Reverse-Mut-SA157-ds1 (V_L100)	5′-TCCGAACGTGATCAGATGAGACGGTTGC-3′
Forward-Mut-SA157-ds1 (V_H44)	5′-CAGGCCCCGGGTGAGTGCCTGGAATGGGTTGCATCTATTGGTTCTTACCCTG-3′
Reverse-Mut-SA157-ds1 (V_H44)	5′-CTCACCCGGGGCCTGAC-3′
Forward-Mut-SA157-ds2 (V_L43)	5′-CAGAAACCAGGAAAATGCCCGAAGCTTCTGATTTACTCTGCATCC-3′
Reverse-Mut-SA157-ds2 (V_L43)	5′-TTTTCCTGGTTTCTGTTGATACCAGGCTAC-3′
Forward-Mut-SA157-ds2 (V_H105)	5′-ACTGACTACTGGGGTTGCGGAACCCTGGTCACCGTCTCC-3′
Reverse-Mut-SA157-ds2 (V_H105)	5′-ACCCCAGTAGTCAGTAGCATCGTACC-3′
Forward-SA157 (V_L)	5′-GGAGGCGGAGGGTCGGCCAGCGATATCCAGATGACCCAGTC-3′
Reverse-SA157 (V_L)	5′-GGAACCGCCCCCTCCTTTGATCTCCACCTCGGTACCCTGTC-3′
Forward-SA157 (V_H)	5′-GGAGGCGGAGGGTCGGCCGAGGTTCAGCTGGTGG-3′
Reverse-SA157 (V_H)	5′-GGAACCGCCCCCTCCCGAGGAGACGGTGACCAGGG-3′
Forward-Mut-SA129-ds1 (V_L100)	5′-CTGATCACGTTCGGATGCGGTACCAAGGTGGAGATCGAAGGTACTACTG-3′
Reverse-Mut-SA129-ds1 (V_L100)	5′-TCCGAACGTGATCAGAGCATGACCTC-3′
Forward-Mut-SA129-ds1 (V_H44)	5′-CAGGCCCCGGGTAAGTGCCTGGAATGGGTTGCATCTATTTACCC-3′
Reverse-Mut-SA129-ds1 (V_H44)	5′-CTTACCCGGGGCCTGACG-3′
Forward-Mut-SA129-ds2 (V_L43)	5′-CAGAAACCAGGAAAATGCCCGAAGCTTCTGATTTACGGTGCATCC-3′
Reverse-Mut-SA129-ds2 (V_L43)	5′-TTTTCCTGGTTTCTGTTGATACCAGGCTAC-3′
Forward-Mut-SA129-ds2 (V_H105)	5′-TTGGACTACTGGGGTTGCGGAACCCTGGTCACCGTCTCC-3′

Reverse-Mut-SA129-ds2 (V_H105)	5′-ACCCCAGTAGTCCAAAGCGAAGTAAGAG-3′
Forward-SA^{CTR} (scFv)	5′-GGAGGCGGAGGGTCGGCTAGCCAAGTTAAACTGGAACAGTCCGGTGC-3′
Reverse-SA^{CTR} (scFv)	5′-GGAACCGCCCCCTCCCTTGATTTCCAACTTCGTTCCACACCC-3′

Sequences of genes coding for antibody single-chain variable fragments (scFv) fused to the C-terminus of mouse serum albumin (SA): ^{N}SA-scFv^{C}

DNA sequences of genes are provided. Start and stop codons are shown in black bold. The sequences include the secretory leader peptide (LS, in grey underlined), the mouse serum albumin (SA, in grey), a long flexible (Gly$_4$Ser)$_3$ linker (in black italic and underlined), the antibody single-chain variable fragment (scFv, in blue bold), a short flexible (Gly$_4$Ser) linker (in black italic and underlined) and the hexa-histidine tag (His$_6$, in red bold). Cysteine residues introduced to form stabilizing intermolecular disulfide bridges (ds1 and ds2) are shown in in orange italic bold.

gWiz-LS-mouse SA-(Gly$_4$Ser)$_3$-scFv (V$_L$-V$_H$) CK138-(Gly$_4$Ser)-His$_6$

ATGGACATGAGAGTGCCTGCTCAGCTGCTGGGCCTGCTGCTGCTGTGGCTGCCTGGTGCTAGATGCGAAGCACACAAGAGTGAGATCGCCCATCGGTATAA
TGATTTGGGAGAACAACATTTCAAAGGCCTAGTCCTGATTGCCTTTTCCCAGTATCTCCAGAAATGCTCATACGATGAGCATGCCAAATTAGTGCAGGAAG
TAACAGACTTTGCAAAGACGTGTGTTGCCGATGAGTCTGCCGCCAACTGTGACAAATCCCTTCACACTCTTTTTGGAGATAAGTTGTGTGCCATTCCAAAC
CTCCGTGAAAACTATGGTGAACTGGCTGACTGCTGTACAAAACAAGAGCCCGAAAGAAACGAATGTTTCCTGCAACACAAAGATGACAACCCCAGCCTACC
ACCATTTGAAAGGCCAGAGGCTGAGGCCATGTGCACCTCCTTTAAGGAAAACCCAACCACCTTTATGGGACACTATTTGCATGAAGTTGCCAGAAGACATC
CTTATTTCTATGCCCCAGAACTTCTTTACTATGCTGAGCAGTACAATGAGATTCTGACCCAGTGTTGTGCAGAGGCTGACAAGGAAAGCTGCCTGACCCCG
AAGCTTGATGGTGTGAAGGAGAAAGCATTGGTCTCATCTGTCCGTCAGAGAATGAAGTGCTCCAGTATGCAGAAGTTTGGAGAGAGAGCTTTTAAAGCATG
GGCAGTAGCTCGTCTGAGCCAGACATTCCCCAATGCTGACTTTGCAGAAATCACCAAATTGGCAACAGACCTGACCAAAGTCAACAAGGAGTGCTGCCATG
GTGACCTGCTGGAATGCGCAGATGACAGGGCGGAACTTGCCAAGTACATGTGTGAAAACCAGGCGACTATCTCCAGCAAACTGCAGACTTGCTGCGATAAA
CCACTGTTGAAGAAAGCCCACTGTCTTAGTGAGGTGGAGCATGACACCATGCCTGCTGATCTGCCTGCCATTGCTGCTGATTTTGTTGAGGACCAGGAAGT
GTGCAAGAACTATGCTGAGGCCAAGGATGTCTTCCTGGGCACGTTCTTGTATGAATATTCAAGAAGACACCCTGATTACTCTGTATCCCTGTTGCTGAGAC
TTGCTAAGAAATATGAAGCCACTCTGGAAAAGTGCTGCGCTGAAGCCAATCCTCCCGCATGCTACGGCACAGTGCTTGCTGAATTTCAGCCTCTTGTAGAA
GAGCCTAAGAACTTGGTCAAAACCAACTGTGATCTTTACGAGAAGCTTGGAGAATATGGATTCCAAAATGCCATTCTAGTTCGCTACACCCAGAAAGCACC
TCAGGTGTCAACCCCAACTCTCGTGGAGGCTGCAAGAAACCTAGGAAGAGTGGGCACCAAGTGTTGTACACTTCCTGAAGATCAGAGACTGCCTTGTGTGG
AAGACTATCTGTCTGCAATCCTGAACCGTGTGTGTCTGCTGCATGAGAAGACCCCAGTGAGTGAGCATGTTACCAAGTGCTGTAGTGGATCCCTGGTGGAA
AGGCGGCCATGCTTCTCTGCTCTGACAGTTGATGAAACATATGTCCCCAAAGAGTTTAAAGCTGAGACCTTCACCTTCCACTCTGATATCTGCACACTTCC
AGAGAAGGAGAAGCAGATTAAGAAACAAACGGCTCTTGCTGAGCTGGTGAAGCACAAGCCCAAGGCTACAGCGGAGCAACTGAAGACTGTCATGGATGACT
TTGCACAGTTCCTGGATACATGTTGCAAGGCTGCTGACAAGGACACCTGCTTCTCGACTGAGGGTCCAAACCTTGTCACTAGATGCAAAGACGCCTTAGCC
*GGTGGAGGAGGCTCTGGTGGAGGCGGTAGCGGAGGCGGAGGGTCG***GCTATCCAGATGACCCGGTCCCCGAGCTCCCTGTCCGCCTCTGTGGGCGATAGGGT**
CACCATCACCTGCCGTGCCAGTCAGTACCACGACGGTTCTGCAGCCTGGTATCAACAGAAACCAGGAAAAGCTCCGAAGCTTCTGATTTACGGTGCATCCT

ACCTCTACTCTGGAGTCCCTTCCCGCTTCTCTGGTAGCCGTTCCGGGACGGATTTCACTCTGACCATCAGCAGTCTGCAGCCGGAAGACTTCGCAACTTAT
TACTGTCAGCAATCTTCTTATTCTCTGATCACGTTCGGACAGGGTACCAAGGTGGAGATCAAAGGTACTACTGCCGCTAGTGGTAGTAGTGGTGGCAGTAG
CAGTGGTGCCGAGGTTCAGCTGGTGGAGTCTGACGGTGGCCTGGTGCAGCCAGGGGGCTCACTCCGTTTGTCCTGTGCAGCTTCTGGCTTCAACCTCTCTT
ACTACGGTATGCACTGGGTGCGTCAGGCCCCGGGTAAGGGCCTGGAATGGGTTGCATACATTGCTTCTTACCCTGGCTACACTTCTTATGCCGATAGCGTC
AAGGGCCGTTTCACTATAAGCGCAGACACATCCAAAAACACAGCCTACCTACAAATGAACAGCTTAAGAGCTGAGGACACTGCCGTCTACTATTGTGCTCG
CTCTGGTTACAGTTACTCTCCGTATTATTCTTGGTTCTCTGCTGGTATGAACTACTGGGGTCAAGGAGCCCTGGTCACCGTCTCCTCG*GGAGGGGGCGGTT*
*CC***CACCATCACCACCATCACTGATAG**

gWiz-LS-mouse SA-(Gly$_4$Ser)$_3$-scFv (V_L-V_H) CK157-(Gly$_4$Ser)-His$_6$

ATGGACATGAGAGTGCCTGCTCAGCTGCTGGGCCTGCTGCTGCTGTGGCTGCCTGGTGCTAGATGCGAAGCACACAAGAGTGAGATCGCCCATCGGTATAA
TGATTTGGGAGAACAACATTTCAAAGGCCTAGTCCTGATTGCCTTTTCCCAGTATCTCCAGAAATGCTCATACGATGAGCATGCCAAATTAGTGCAGGAAG
TAACAGACTTTGCAAAGACGTGTGTTGCCGATGAGTCTGCCGCCAACTGTGACAAATCCCTTCACACTCTTTTTGGAGATAAGTTGTGTGCCATTCCAAAC
CTCCGTGAAAACTATGGTGAACTGGCTGACTGCTGTACAAAACAAGAGCCCGAAAGAAACGAATGTTTCCTGCAACACAAAGATGACAACCCCAGCCTACC
ACCATTTGAAAGGCCAGAGGCTGAGGCCATGTGCACCTCCTTTAAGGAAAACCCAACCACCTTTATGGGACACTATTTGCATGAAGTTGCCAGAAGACATC
CTTATTTCTATGCCCCAGAACTTCTTTACTATGCTGAGCAGTACAATGAGATTCTGACCCAGTGTTGTGCAGAGGCTGACAAGGAAAGCTGCCTGACCCCG
AAGCTTGATGGTGTGAAGGAGAAAGCATTGGTCTCATCTGTCCGTCAGAGAATGAAGTGCTCCAGTATGCAGAAGTTTGGAGAGAGAGCTTTTAAAGCATG
GGCAGTAGCTCGTCTGAGCCAGACATTCCCCAATGCTGACTTTGCAGAAATCACCAAATTGGCAACAGACCTGACCAAAGTCAACAAGGAGTGCTGCCATG
GTGACCTGCTGGAATGCGCAGATGACAGGGCGGAACTTGCCAAGTACATGTGTGAAAACCAGGCGACTATCTCCAGCAAACTGCAGACTTGCTGCGATAAA
CCACTGTTGAAGAAAGCCCACTGTCTTAGTGAGGTGGAGCATGACACCATGCCTGCTGATCTGCCTGCCATTGCTGCTGATTTTGTTGAGGACCAGGAAGT
GTGCAAGAACTATGCTGAGGCCAAGGATGTCTTCCTGGGCACGTTCTTGTATGAATATTCAAGAAGACACCCTGATTACTCTGTATCCCTGTTGCTGAGAC
TTGCTAAGAAATATGAAGCCACTCTGGAAAAGTGCTGCGCTGAAGCCAATCCTCCCGCATGCTACGGCACAGTGCTTGCTGAATTTCAGCCTCTTGTAGAA
GAGCCTAAGAACTTGGTCAAAACCAACTGTGATCTTTACGAGAAGCTTGGAGAATATGGATTCCAAAATGCCATTCTAGTTCGCTACACCCAGAAAGCACC
TCAGGTGTCAACCCCAACTCTCGTGGAGGCTGCAAGAAACCTAGGAAGAGTGGGCACCAAGTGTTGTACACTTCCTGAAGATCAGAGACTGCCTTGTGTGG
AAGACTATCTGTCTGCAATCCTGAACCGTGTGTGTCTGCTGCATGAGAAGACCCCAGTGAGTGAGCATGTTACCAAGTGCTGTAGTGGATCCCTGGTGGAA
AGGCGGCCATGCTTCTCTGCTCTGACAGTTGATGAAACATATGTCCCCAAAGAGTTTAAAGCTGAGACCTTCACCTTCCACTCTGATATCTGCACACTTCC
AGAGAAGGAGAAGCAGATTAAGAAACAAACGGCTCTTGCTGAGCTGGTGAAGCACAAGCCCAAGGCTACAGCGGAGCAACTGAAGACTGTCATGGATGACT
TTGCACAGTTCCTGGATACATGTTGCAAGGCTGCTGACAAGGACACCTGCTTCTCGACTGAGGGTCCAAACCTTGTCACTAGATGCAAAGACGCCTTAGCC
*GGTGGAGGAGGCTCTGGTGGAGGCGGTAGCGGAGGCGGAGGGTCG***GATATCCAGATGACCCAGTCCCCGAGCTCCCTGTCCGCCTCTGTGGGCGATAGGGT**
CACCATCACCTGCCGTGCCAGTCAGTCTTACGGTGGTGTAGCCTGGTATCAACAGAAACCAGGAAAAGCCCCGAAGCTTCTGATTTACTCTGCATCCTACC
TCTACTCTGGAGTCCCTTCTCGCTTCTCTGGTAGCCGTTCCGGGACGGATTTCACTCTGACCATCAGCAGTCTGCAGCCGGAAGACTTCGCAACTTATTAC
TGTCAGCAACCATCTCATCTGATCACGTTCGGACAGGGTACCGAGGTGGAGATCAAAGGTACTACTGCCGCTAGTGGTAGTAGTGGTGGCAGTAGCAGTGG
TGCCGAGGTTCAGCTGGTGGAGTCTGGCGGTGGCCTGGTGCAGCCAGGGGGCTCACTCCGTTTGTCCTGTGCAGCTTCTGGCTCCAACCCCTACTACTACG
GTGGTACGCACTGGGTGCGTCAGGCCCCGGGTGAGGAGCTGGAATGGGTTGCATCTATTGGTTCTTACCCTGGCTACACTGACTATGCCGATAGCGTCAAG
GGCCGTTTCACTATAAGCGCAGACACATCCAAAAACACAGCCTACCTACAAATGAACAGCTTAAGAGCTGAGGACACTGCCGTCTATTATTGTGCTCGCCA

TTACTACTGGTACGATGCTACTGACTACTGGGGTCAAGGAACCCTGGTCACCGTCTCCTCG*GGAGGGGGCGGTTCC***CACCATCACCACCATCACTGATAG**

gWiz-LS-mouse SA-(Gly$_4$Ser)$_3$-scFv (V$_L$-V$_H$) CK129-(Gly$_4$Ser)-His$_6$

ATGGACATGAGAGTGCCTGCTCAGCTGCTGGGCCTGCTGCTGCTGTGGCTGCCTGGTGCTAGATGCGAAGCACACAAGAGTGAGATCGCCCATCGGTATAA
TGATTTGGGAGAACAACATTTCAAAGGCCTAGTCCTGATTGCCTTTTCCCAGTATCTCCAGAAATGCTCATACGATGAGCATGCCAAATTAGTGCAGGAAG
TAACAGACTTTGCAAAGACGTGTGTTGCCGATGAGTCTGCCGCCAACTGTGACAAATCCCTTCACACTCTTTTTGGAGATAAGTTGTGTGCCATTCCAAAC
CTCCGTGAAAACTATGGTGAACTGGCTGACTGCTGTACAAAACAAGAGCCCGAAAGAAACGAATGTTTCCTGCAACACAAAGATGACAACCCCAGCCTACC
ACCATTTGAAAGGCCAGAGGCTGAGGCCATGTGCACCTCCTTTAAGGAAAACCCAACCACCTTTATGGGACACTATTTGCATGAAGTTGCCAGAAGACATC
CTTATTTCTATGCCCCAGAACTTCTTTACTATGCTGAGCAGTACAATGAGATTCTGACCCAGTGTTGTGCAGAGGCTGACAAGGAAAGCTGCCTGACCCCG
AAGCTTGATGGTGTGAAGGAGAAAGCATTGGTCTCATCTGTCCGTCAGAGAATGAAGTGCTCCAGTATGCAGAAGTTTGGAGAGAGAGCTTTTAAAGCATG
GGCAGTAGCTCGTCTGAGCCAGACATTCCCCAATGCTGACTTTGCAGAAATCACCAAATTGGCAACAGACCTGACCAAAGTCAACAAGGAGTGCTGCCATG
GTGACCTGCTGGAATGCGCAGATGACAGGGCGGAACTTGCCAAGTACATGTGTGAAAACCAGGCGACTATCTCCAGCAAACTGCAGACTTGCTGCGATAAA
CCACTGTTGAAGAAAGCCCACTGTCTTAGTGAGGTGGAGCATGACACCATGCCTGCTGATCTGCCTGCCATTGCTGCTGATTTTGTTGAGGACCAGGAAGT
GTGCAAGAACTATGCTGAGGCCAAGGATGTCTTCCTGGGCACGTTCTTGTATGAATATTCAAGAAGACACCCTGATTACTCTGTATCCCTGTTGCTGAGAC
TTGCTAAGAAATATGAAGCCACTCTGGAAAAGTGCTGCGCTGAAGCCAATCCTCCCGCATGCTACGGCACAGTGCTTGCTGAATTTCAGCCTCTTGTAGAA
GAGCCTAAGAACTTGGTCAAAACCAACTGTGATCTTTACGAGAAGCTTGGAGAATATGGATTCCAAAATGCCATTCTAGTTCGCTACACCCAGAAAGCACC
TCAGGTGTCAACCCCAACTCTCGTGGAGGCTGCAAGAAACCTAGGAAGAGTGGGCACCAAGTGTTGTACACTTCCTGAAGATCAGAGACTGCCTTGTGTGG
AAGACTATCTGTCTGCAATCCTGAACCGTGTGTGTCTGCTGCATGAGAAGACCCCAGTGAGTGAGCATGTTACCAAGTGCTGTAGTGGATCCCTGGTGGAA
AGGCGGCCATGCTTCTCTGCTCTGACAGTTGATGAAACATATGTCCCCAAAGAGTTTAAAGCTGAGACCTTCACCTTCCACTCTGATATCTGCACACTTCC
AGAGAAGGAGAAGCAGATTAAGAAACAAACGGCTCTTGCTGAGCTGGTGAAGCACAAGCCCAAGGCTACAGCGGAGCAACTGAAGACTGTCATGGATGACT
TTGCACAGTTCCTGGATACATGTTGCAAGGCTGCTGACAAGGACACCTGCTTCTCGACTGAGGGTCCAAACCTTGTCACTAGATGCAAAGACGCCTTAGCC
*GGTGGAGGAGGCTCTGGTGGAGGCGGTAGCGGAGGCGGAGGGTCG***GCTAGCGATATCCAGATGACCCAGTCCCCGAGCCCCCTGTCCGCCTCTGTGGGCGA**
TAGGGTCACCATCACCTGCCGTGCCAGTCAGTACGGTGGTTACGTAGCCTGGTATCAACAGAAACCAGGAAAAGCTCCGAAGCTTCTGATTTACGGTGCAT
CCCTTCTCTACTCTGGAGTCCCTTCTCGCTTCTCTGGTGGCCGTTCCGGGACGGATTTCACTCTGACCATCAGCAGTCTGCAGCCGGAAGACTTCGCAACT
TATTACTGTCAGCGAGGTCATGCTCTGATCACGTTCGGACAGGGTACCAAGGTGGAGATCGAAGGTACTACTGCCGCTAGTGGTAGTAGTGGTGGCAGTAG
CAGTGGTGCCGAGGTTCAGCTGGTGGAGTCTGGCGGTGGCCTGGTGCAGCCAGGGGGCTCACTCCGTTTATCCTGTGCAGCTTCTGGCTTCAACATCTCTT
CTTACGGTTCTATGCACTGGGTGCGTCAGGCCCCGGGTAAGGGCCTGGAATGGGTTGCATCTATTTACCCTTACTCTAGCTCTACTTACTATGCCGATAGC
GTCAAGGGCCGTTTCACTATAAGCGCAGACACATCCAAAAACACAGCCTACCTACAAATGAACAGCTTAAGAGCTGAGGACACTGCCGTCTATTATTGTGC
TCGTGGTTACGGTCCGTGGTACGCTTACTCTTACTTCGCTTTGGACTACTGGGGTCAAGGAACCCTGGTCACCGTCTCCTCG*GGAGGGGGCGGTTCC***CACC**
ATCACCACCATCACTGATAG

gWiz-LS-mouse SA-(Gly$_4$Ser)$_3$-scFv (V$_L$-V$_H$) CK138-ds1 (V$_L$100$^{Q>C}$ / V$_H$44$^{G>C}$)-(Gly$_4$Ser)-His$_6$

ATGGACATGAGAGTGCCTGCTCAGCTGCTGGGCCTGCTGCTGCTGTGGCTGCCTGGTGCTAGATGCGAAGCACACAAGAGTGAGATCGCCCATCGGTATAA

TGATTTGGGAGAACAACATTTCAAAGGCCTAGTCCTGATTGCCTTTTCCCAGTATCTCCAGAAATGCTCATACGATGAGCATGCCAAATTAGTGCAGGAAG
TAACAGACTTTGCAAAGACGTGTGTTGCCGATGAGTCTGCCGCCAACTGTGACAAATCCCTTCACACTCTTTTTGGAGATAAGTTGTGTGCCATTCCAAAC
CTCCGTGAAAACTATGGTGAACTGGCTGACTGCTGTACAAAACAAGAGCCCGAAAGAAACGAATGTTTCCTGCAACACAAAGATGACAACCCCAGCCTACC
ACCATTTGAAAGGCCAGAGGCTGAGGCCATGTGCACCTCCTTTAAGGAAAACCCAACCACCTTTATGGGACACTATTTGCATGAAGTTGCCAGAAGACATC
CTTATTTCTATGCCCCAGAACTTCTTTACTATGCTGAGCAGTACAATGAGATTCTGACCCAGTGTTGTGCAGAGGCTGACAAGGAAAGCTGCCTGACCCCG
AAGCTTGATGGTGTGAAGGAGAAAGCATTGGTCTCATCTGTCCGTCAGAGAATGAAGTGCTCCAGTATGCAGAAGTTTGGAGAGAGAGCTTTTAAAGCATG
GGCAGTAGCTCGTCTGAGCCAGACATTCCCCAATGCTGACTTTGCAGAAATCACCAAATTGGCAACAGACCTGACCAAAGTCAACAAGGAGTGCTGCCATG
GTGACCTGCTGGAATGCGCAGATGACAGGGCGGAACTTGCCAAGTACATGTGTGAAAACCAGGCGACTATCTCCAGCAAACTGCAGACTTGCTGCGATAAA
CCACTGTTGAAGAAAGCCCACTGTCTTAGTGAGGTGGAGCATGACACCATGCCTGCTGATCTGCCTGCCATTGCTGCTGATTTTGTTGAGGACCAGGAAGT
GTGCAAGAACTATGCTGAGGCCAAGGATGTCTTCCTGGGCACGTTCTTGTATGAATATTCAAGAAGACACCCTGATTACTCTGTATCCCTGTTGCTGAGAC
TTGCTAAGAAATATGAAGCCACTCTGGAAAAGTGCTGCGCTGAAGCCAATCCTCCCGCATGCTACGGCACAGTGCTTGCTGAATTTCAGCCTCTTGTAGAA
GAGCCTAAGAACTTGGTCAAAACCAACTGTGATCTTTACGAGAAGCTTGGAGAATATGGATTCCAAAATGCCATTCTAGTTCGCTACACCCAGAAAGCACC
TCAGGTGTCAACCCCAACTCTCGTGGAGGCTGCAAGAAACCTAGGAAGAGTGGGCACCAAGTGTTGTACACTTCCTGAAGATCAGAGACTGCCTTGTGTGG
AAGACTATCTGTCTGCAATCCTGAACCGTGTGTGTCTGCTGCATGAGAAGACCCCAGTGAGTGAGCATGTTACCAAGTGCTGTAGTGGATCCCTGGTGGAA
AGGCGGCCATGCTTCTCTGCTCTGACAGTTGATGAAACATATGTCCCCAAAGAGTTTAAAGCTGAGACCTTCACCTTCCACTCTGATATCTGCACACTTCC
AGAGAAGGAGAAGCAGATTAAGAAACAAACGGCTCTTGCTGAGCTGGTGAAGCACAAGCCCAAGGCTACAGCGGAGCAACTGAAGACTGTCATGGATGACT
TTGCACAGTTCCTGGATACATGTTGCAAGGCTGCTGACAAGGACACCTGCTTCTCGACTGAGGGTCCAAACCTTGTCACTAGATGCAAAGACGCCTTAGCC
*GGTGGAGGAGGCTCTGGTGGAGGCGGTAGCGGAGGCGGAGGGTCG***GCTATCCAGATGACCCGGTCCCCGAGCTCCCTGTCCGCCTCTGTGGGCGATAGGGT**
CACCATCACCTGCCGTGCCAGTCAGTACCACGACGGTTCTGCAGCCTGGTATCAACAGAAACCAGGAAAAGCTCCGAAGCTTCTGATTTACGGTGCATCCT
ACCTCTACTCTGGAGTCCCTTCCCGCTTCTCTGGTAGCCGTTCCGGGACGGATTTCACTCTGACCATCAGCAGTCTGCAGCCGGAAGACTTCGCAACTTAT
TACTGTCAGCAATCTTCTTATTCTCTGATCACGTTCGGA*TGC*GGTACCAAGGTGGAGATCAAAGGTACTACTGCCGCTAGTGGTAGTAGTGGTGGCAGTAG
CAGTGGTGCCGAGGTTCAGCTGGTGGAGTCTGACGGTGGCCTGGTGCAGCCAGGGGGCTCACTCCGTTTGTCCTGTGCAGCTTCTGGCTTCAACCTCTCTT
ACTACGGTATGCACTGGGTGCGTCAGGCCCCGGGTAAG*T*GCCTGGAATGGGTTGCATACATTGCTTCTTACCCTGGCTACACTTCTTATGCCGATAGCGTC
AAGGGCCGTTTCACTATAAGCGCAGACACATCCAAAAACACAGCCTACCTACAAATGAACAGCTTAAGAGCTGAGGACACTGCCGTCTACTATTGTGCTCG
CTCTGGTTACAGTTACTCTCCGTATTATTCTTGGTTCTCTGCTGGTATGAACTACTGGGGTCAAGGAGCCCTGGTCACCGTCTCCTCG*GGAGGGGGCGGTT*
*CC***CACCATCACCACCATCACTGATAG**

gWiz-LS-mouse SA-$(Gly_4Ser)_3$-scFv (V_L-V_H) CK138-ds2 ($V_L43^{A>C}$ / $V_H105^{Q>C}$)-(Gly_4Ser)-His_6

ATGGACATGAGAGTGCCTGCTCAGCTGCTGGGCCTGCTGCTGCTGTGGCTGCCTGGTGCTAGATGCGAAGCACACAAGAGTGAGATCGCCCATCGGTATAA
TGATTTGGGAGAACAACATTTCAAAGGCCTAGTCCTGATTGCCTTTTCCCAGTATCTCCAGAAATGCTCATACGATGAGCATGCCAAATTAGTGCAGGAAG
TAACAGACTTTGCAAAGACGTGTGTTGCCGATGAGTCTGCCGCCAACTGTGACAAATCCCTTCACACTCTTTTTGGAGATAAGTTGTGTGCCATTCCAAAC
CTCCGTGAAAACTATGGTGAACTGGCTGACTGCTGTACAAAACAAGAGCCCGAAAGAAACGAATGTTTCCTGCAACACAAAGATGACAACCCCAGCCTACC
ACCATTTGAAAGGCCAGAGGCTGAGGCCATGTGCACCTCCTTTAAGGAAAACCCAACCACCTTTATGGGACACTATTTGCATGAAGTTGCCAGAAGACATC
CTTATTTCTATGCCCCAGAACTTCTTTACTATGCTGAGCAGTACAATGAGATTCTGACCCAGTGTTGTGCAGAGGCTGACAAGGAAAGCTGCCTGACCCCG

AAGCTTGATGGTGTGAAGGAGAAAGCATTGGTCTCATCTGTCCGTCAGAGAATGAAGTGCTCCAGTATGCAGAAGTTTGGAGAGAGAGCTTTTAAAGCATG
GGCAGTAGCTCGTCTGAGCCAGACATTCCCCAATGCTGACTTTGCAGAAATCACCAAATTGGCAACAGACCTGACCAAAGTCAACAAGGAGTGCTGCCATG
GTGACCTGCTGGAATGCGCAGATGACAGGGCGGAACTTGCCAAGTACATGTGTGAAAACCAGGCGACTATCTCCAGCAAACTGCAGACTTGCTGCGATAAA
CCACTGTTGAAGAAAGCCCACTGTCTTAGTGAGGTGGAGCATGACACCATGCCTGCTGATCTGCCTGCCATTGCTGCTGATTTTGTTGAGGACCAGGAAGT
GTGCAAGAACTATGCTGAGGCCAAGGATGTCTTCCTGGGCACGTTCTTGTATGAATATTCAAGAAGACACCCTGATTACTCTGTATCCCTGTTGCTGAGAC
TTGCTAAGAAATATGAAGCCACTCTGGAAAAGTGCTGCGCTGAAGCCAATCCTCCCGCATGCTACGGCACAGTGCTTGCTGAATTTCAGCCTCTTGTAGAA
GAGCCTAAGAACTTGGTCAAAACCAACTGTGATCTTTACGAGAAGCTTGGAGAATATGGATTCCAAAATGCCATTCTAGTTCGCTACACCCAGAAAGCACC
TCAGGTGTCAACCCCAACTCTCGTGGAGGCTGCAAGAAACCTAGGAAGAGTGGGCACCAAGTGTTGTACACTTCCTGAAGATCAGAGACTGCCTTGTGTGG
AAGACTATCTGTCTGCAATCCTGAACCGTGTGTGTCTGCTGCATGAGAAGACCCCAGTGAGTGAGCATGTTACCAAGTGCTGTAGTGGATCCCTGGTGGAA
AGGCGGCCATGCTTCTCTGCTCTGACAGTTGATGAAACATATGTCCCCAAAGAGTTTAAAGCTGAGACCTTCACCTTCCACTCTGATATCTGCACACTTCC
AGAGAAGGAGAAGCAGATTAAGAAACAAACGGCTCTTGCTGAGCTGGTGAAGCACAAGCCCAAGGCTACAGCGGAGCAACTGAAGACTGTCATGGATGACT
TTGCACAGTTCCTGGATACATGTTGCAAGGCTGCTGACAAGGACACCTGCTTCTCGACTGAGGGTCCAAACCTTGTCACTAGATGCAAAGACGCCTTAGCC
*GGTGGAGGAGGCTCTGGTGGAGGCGGTAGCGGAGGCGGAGGGTCG*GCTAGCGCTATCCAGATGACCCGGTCCCCGAGCTCCCTGTCCGCCTCTGTGGGCGA
TAGGGTCACCATCACCTGCCGTGCCAGTCAGTACCACGACGGTTCTGCAGCCTGGTATCAACAGAAACCAGGAAAA*TGC*CCGAAGCTTCTGATTTACGGTG
CATCCTACCTCTACTCTGGAGTCCCTTCCCGCTTCTCTGGTAGCCGTTCCGGGACGGATTTCACTCTGACCATCAGCAGTCTGCAGCCGGAAGACTTCGCA
ACTTATTACTGTCAGCAATCTTCTTATTCTCTGATCACGTTCGGACAGGGTACCAAGGTGGAGATCAAAGGTACTACTGCCGCTAGTGGTAGTAGTGGTGG
CAGTAGCAGTGGTGCCGAGGTTCAGCTGGTGGAGTCTGACGGTGGCCTGGTGCAGCCAGGGGGCTCACTCCGTTTGTCCTGTGCAGCTTCTGGCTTCAACC
TCTCTTACTACGGTATGCACTGGGTGCGTCAGGCCCCGGGTAAGGGCCTGGAATGGGTTGCATACATTGCTTCTTACCCTGGCTACACTTCTTATGCCGAT
AGCGTCAAGGGCCGTTTCACTATAAGCGCAGACACATCCAAAAACACAGCCTACCTACAAATGAACAGCTTAAGAGCTGAGGACACTGCCGTCTACTATTG
TGCTCGCTCTGGTTACAGTTACTCTCCGTATTATTCTTGGTTCTCTGCTGGTATGAACTACTGGGGT*TGC*GGAGCCCTGGTCACCGTCTCCTCG*GGAGGGG*
*GCGGTTCC*CACCATCACCACCATCACTGATAG

gWiz-LS-mouse SA-$(Gly_4Ser)_3$-scFv (V_L-V_H) CK157-ds1 ($V_L100^{Q>C}$ / $V_H44^{E>C}$)-(Gly_4Ser)-His_6

ATGGACATGAGAGTGCCTGCTCAGCTGCTGGGCCTGCTGCTGCTGTGGCTGCCTGGTGCTAGATGCGAAGCACACAAGAGTGAGATCGCCCATCGGTATAA
TGATTTGGGAGAACAACATTTCAAAGGCCTAGTCCTGATTGCCTTTTCCCAGTATCTCCAGAAATGCTCATACGATGAGCATGCCAAATTAGTGCAGGAAG
TAACAGACTTTGCAAAGACGTGTGTTGCCGATGAGTCTGCCGCCAACTGTGACAAATCCCTTCACACTCTTTTTGGAGATAAGTTGTGTGCCATTCCAAAC
CTCCGTGAAAACTATGGTGAACTGGCTGACTGCTGTACAAAACAAGAGCCCGAAAGAAACGAATGTTTCCTGCAACACAAAGATGACAACCCCAGCCTACC
ACCATTTGAAAGGCCAGAGGCTGAGGCCATGTGCACCTCCTTTAAGGAAAACCCAACCACCTTTATGGGACACTATTTGCATGAAGTTGCCAGAAGACATC
CTTATTTCTATGCCCCAGAACTTCTTTACTATGCTGAGCAGTACAATGAGATTCTGACCCAGTGTTGTGCAGAGGCTGACAAGGAAAGCTGCCTGACCCCG
AAGCTTGATGGTGTGAAGGAGAAAGCATTGGTCTCATCTGTCCGTCAGAGAATGAAGTGCTCCAGTATGCAGAAGTTTGGAGAGAGAGCTTTTAAAGCATG
GGCAGTAGCTCGTCTGAGCCAGACATTCCCCAATGCTGACTTTGCAGAAATCACCAAATTGGCAACAGACCTGACCAAAGTCAACAAGGAGTGCTGCCATG
GTGACCTGCTGGAATGCGCAGATGACAGGGCGGAACTTGCCAAGTACATGTGTGAAAACCAGGCGACTATCTCCAGCAAACTGCAGACTTGCTGCGATAAA
CCACTGTTGAAGAAAGCCCACTGTCTTAGTGAGGTGGAGCATGACACCATGCCTGCTGATCTGCCTGCCATTGCTGCTGATTTTGTTGAGGACCAGGAAGT
GTGCAAGAACTATGCTGAGGCCAAGGATGTCTTCCTGGGCACGTTCTTGTATGAATATTCAAGAAGACACCCTGATTACTCTGTATCCCTGTTGCTGAGAC

TTGCTAAGAAATATGAAGCCACTCTGGAAAAGTGCTGCGCTGAAGCCAATCCTCCCGCATGCTACGGCACAGTGCTTGCTGAATTTCAGCCTCTTGTAGAA
GAGCCTAAGAACTTGGTCAAAACCAACTGTGATCTTTACGAGAAGCTTGGAGAATATGGATTCCAAAATGCCATTCTAGTTCGCTACACCCAGAAAGCACC
TCAGGTGTCAACCCCAACTCTCGTGGAGGCTGCAAGAAACCTAGGAAGAGTGGGCACCAAGTGTTGTACACTTCCTGAAGATCAGAGACTGCCTTGTGTGG
AAGACTATCTGTCTGCAATCCTGAACCGTGTGTGTCTGCTGCATGAGAAGACCCCAGTGAGTGAGCATGTTACCAAGTGCTGTAGTGGATCCCTGGTGGAA
AGGCGGCCATGCTTCTCTGCTCTGACAGTTGATGAAACATATGTCCCCAAAGAGTTTAAAGCTGAGACCTTCACCTTCCACTCTGATATCTGCACACTTCC
AGAGAAGGAGAAGCAGATTAAGAAACAAACGGCTCTTGCTGAGCTGGTGAAGCACAAGCCCAAGGCTACAGCGGAGCAACTGAAGACTGTCATGGATGACT
TTGCACAGTTCCTGGATACATGTTGCAAGGCTGCTGACAAGGACACCTGCTTCTCGACTGAGGGTCCAAACCTTGTCACTAGATGCAAAGACGCCTTAGCC
*GGTGGAGGAGGCTCTGGTGGAGGCGGTAGCGGAGGCGGAGGGTCG***GATATCCAGATGACCCAGTCCCCGAGCTCCCTGTCCGCCTCTGTGGGCGATAGGGT**
CACCATCACCTGCCGTGCCAGTCAGTCTTACGGTGGTGTAGCCTGGTATCAACAGAAACCAGGAAAAGCCCCGAAGCTTCTGATTTACTCTGCATCCTACC
TCTACTCTGGAGTCCCTTCTCGCTTCTCTGGTAGCCGTTCCGGGACGGATTTCACTCTGACCATCAGCAGTCTGCAGCCGGAAGACTTCGCAACTTATTAC
TGTCAGCAACCATCTCATCTGATCACGTTCGGA*TGC*GGTACCGAGGTGGAGATCAAAGGTACTACTGCCGCTAGTGGTAGTAGTGGTGGCAGTAGCAGTGG
TGCCGAGGTTCAGCTGGTGGAGTCTGGCGGTGGCCTGGTGCAGCCAGGGGGCTCACTCCGTTTGTCCTGTGCAGCTTCTGGCTCCAACCCCTACTACTACG
GTGGTACGCACTGGGTGCGTCAGGCCCCGGGTGAG*TGC*CTGGAATGGGTTGCATCTATTGGTTCTTACCCTGGCTACACTGACTATGCCGATAGCGTCAAG
GGCCGTTTCACTATAAGCGCAGACACATCCAAAAACACAGCCTACCTACAAATGAACAGCTTAAGAGCTGAGGACACTGCCGTCTATTATTGTGCTCGCCA
TTACTACTGGTACGATGCTACTGACTACTGGGGTCAAGGAACCCTGGTCACCGTCTCCTCG*GGAGGGGGCGGTTCC***CACCATCACCACCATCAC****TGATAG**

gWiz-LS-mouse SA-(Gly$_4$Ser)$_3$-scFv (V_L-V_H) CK157-ds2 ($V_L43^{A>C}$ / $V_H105^{Q>C}$)-(Gly$_4$Ser)-His$_6$

ATGGACATGAGAGTGCCTGCTCAGCTGCTGGGCCTGCTGCTGCTGTGGCTGCCTGGTGCTAGATGCGAAGCACACAAGAGTGAGATCGCCCATCGGTATAA
TGATTTGGGAGAACAACATTTCAAAGGCCTAGTCCTGATTGCCTTTTCCCAGTATCTCCAGAAATGCTCATACGATGAGCATGCCAAATTAGTGCAGGAAG
TAACAGACTTTGCAAAGACGTGTGTTGCCGATGAGTCTGCCGCCAACTGTGACAAATCCCTTCACACTCTTTTTGGAGATAAGTTGTGTGCCATTCCAAAC
CTCCGTGAAAACTATGGTGAACTGGCTGACTGCTGTACAAAACAAGAGCCCGAAAGAAACGAATGTTTCCTGCAACACAAAGATGACAACCCCAGCCTACC
ACCATTTGAAAGGCCAGAGGCTGAGGCCATGTGCACCTCCTTTAAGGAAAACCCAACCACCTTTATGGGACACTATTTGCATGAAGTTGCCAGAAGACATC
CTTATTTCTATGCCCCAGAACTTCTTTACTATGCTGAGCAGTACAATGAGATTCTGACCCAGTGTTGTGCAGAGGCTGACAAGGAAAGCTGCCTGACCCCG
AAGCTTGATGGTGTGAAGGAGAAAGCATTGGTCTCATCTGTCCGTCAGAGAATGAAGTGCTCCAGTATGCAGAAGTTTGGAGAGAGAGCTTTTAAAGCATG
GGCAGTAGCTCGTCTGAGCCAGACATTCCCCAATGCTGACTTTGCAGAAATCACCAAATTGGCAACAGACCTGACCAAAGTCAACAAGGAGTGCTGCCATG
GTGACCTGCTGGAATGCGCAGATGACAGGGCGGAACTTGCCAAGTACATGTGTGAAAACCAGGCGACTATCTCCAGCAAACTGCAGACTTGCTGCGATAAA
CCACTGTTGAAGAAAGCCCACTGTCTTAGTGAGGTGGAGCATGACACCATGCCTGCTGATCTGCCTGCCATTGCTGCTGATTTTGTTGAGGACCAGGAAGT
GTGCAAGAACTATGCTGAGGCCAAGGATGTCTTCCTGGGCACGTTCTTGTATGAATATTCAAGAAGACACCCTGATTACTCTGTATCCCTGTTGCTGAGAC
TTGCTAAGAAATATGAAGCCACTCTGGAAAAGTGCTGCGCTGAAGCCAATCCTCCCGCATGCTACGGCACAGTGCTTGCTGAATTTCAGCCTCTTGTAGAA
GAGCCTAAGAACTTGGTCAAAACCAACTGTGATCTTTACGAGAAGCTTGGAGAATATGGATTCCAAAATGCCATTCTAGTTCGCTACACCCAGAAAGCACC
TCAGGTGTCAACCCCAACTCTCGTGGAGGCTGCAAGAAACCTAGGAAGAGTGGGCACCAAGTGTTGTACACTTCCTGAAGATCAGAGACTGCCTTGTGTGG
AAGACTATCTGTCTGCAATCCTGAACCGTGTGTGTCTGCTGCATGAGAAGACCCCAGTGAGTGAGCATGTTACCAAGTGCTGTAGTGGATCCCTGGTGGAA
AGGCGGCCATGCTTCTCTGCTCTGACAGTTGATGAAACATATGTCCCCAAAGAGTTTAAAGCTGAGACCTTCACCTTCCACTCTGATATCTGCACACTTCC
AGAGAAGGAGAAGCAGATTAAGAAACAAACGGCTCTTGCTGAGCTGGTGAAGCACAAGCCCAAGGCTACAGCGGAGCAACTGAAGACTGTCATGGATGACT

TTGCACAGTTCCTGGATACATGTTGCAAGGCTGCTGACAAGGACACCTGCTTCTCGACTGAGGGTCCAAACCTTGTCACTAGATGCAAAGACGCCTTAGCC
*GGTGGAGGAGGCTCTGGTGGAGGCGGTAGCGGAGGCGGAGGGTCG*GATATCCAGATGACCCAGTCCCCGAGCTCCCTGTCCGCCTCTGTGGGCGATAGGGT
CACCATCACCTGCCGTGCCAGTCAGTCTTACGGTGGTGTAGCCTGGTATCAACAGAAACCAGGAAAA*TG*CCCGAAGCTTCTGATTTACTCTGCATCCTACC
TCTACTCTGGAGTCCCTTCTCGCTTCTCTGGTAGCCGTTCCGGGACGGATTTCACTCTGACCATCAGCAGTCTGCAGCCGGAAGACTTCGCAACTTATTAC
TGTCAGCAACCATCTCATCTGATCACGTTCGGACAGGGTACCGAGGTGGAGATCAAAGGTACTACTGCCGCTAGTGGTAGTAGTGGTGGCAGTAGCAGTGG
TGCCGAGGTTCAGCTGGTGGAGTCTGGCGGTGGCCTGGTGCAGCCAGGGGGCTCACTCCGTTTGTCCTGTGCAGCTTCTGGCTCCAACCCCTACTACTACG
GTGGTACGCACTGGGTGCGTCAGGCCCCGGGTGAGGAGCTGGAATGGGTTGCATCTATTGGTTCTTACCCTGGCTACACTGACTATGCCGATAGCGTCAAG
GGCCGTTTCACTATAAGCGCAGACACATCCAAAAACACAGCCTACCTACAAATGAACAGCTTAAGAGCTGAGGACACTGCCGTCTATTATTGTGCTCGCCA
TTACTACTGGTACGATGCTACTGACTACTGGGGT*TGC*GGAACCCTGGTCACCGTCTCCTCG*GGAGGGGGCGGTTCC*CACCATCACCACCATCACTGATAG

gWiz-LS-mouse SA-(Gly$_4$Ser)-V_L CK157-His$_6$

ATGGACATGAGAGTGCCTGCTCAGCTGCTGGGCCTGCTGCTGCTGTGGCTGCCTGGTGCTAGATGCGAAGCACACAAGAGTGAGATCGCCCATCGGTATAA
TGATTTGGGAGAACAACATTTCAAAGGCCTAGTCCTGATTGCCTTTTCCCAGTATCTCCAGAAATGCTCATACGATGAGCATGCCAAATTAGTGCAGGAAG
TAACAGACTTTGCAAAGACGTGTGTTGCCGATGAGTCTGCCGCCAACTGTGACAAATCCCTTCACACTCTTTTTGGAGATAAGTTGTGTGCCATTCCAAAC
CTCCGTGAAAACTATGGTGAACTGGCTGACTGCTGTACAAAACAAGAGCCCGAAAGAAACGAATGTTTCCTGCAACACAAAGATGACAACCCCAGCCTACC
ACCATTTGAAAGGCCAGAGGCTGAGGCCATGTGCACCTCCTTTAAGGAAAACCCAACCACCTTTATGGGACACTATTTGCATGAAGTTGCCAGAAGACATC
CTTATTTCTATGCCCCAGAACTTCTTTACTATGCTGAGCAGTACAATGAGATTCTGACCCAGTGTTGTGCAGAGGCTGACAAGGAAAGCTGCCTGACCCCG
AAGCTTGATGGTGTGAAGGAGAAAGCATTGGTCTCATCTGTCCGTCAGAGAATGAAGTGCTCCAGTATGCAGAAGTTTGGAGAGAGAGCTTTTAAAGCATG
GGCAGTAGCTCGTCTGAGCCAGACATTCCCCAATGCTGACTTTGCAGAAATCACCAAATTGGCAACAGACCTGACCAAAGTCAACAAGGAGTGCTGCCATG
GTGACCTGCTGGAATGCGCAGATGACAGGGCGGAACTTGCCAAGTACATGTGTGAAAACCAGGCGACTATCTCCAGCAAACTGCAGACTTGCTGCGATAAA
CCACTGTTGAAGAAAGCCCACTGTCTTAGTGAGGTGGAGCATGACACCATGCCTGCTGATCTGCCTGCCATTGCTGCTGATTTTGTTGAGGACCAGGAAGT
GTGCAAGAACTATGCTGAGGCCAAGGATGTCTTCCTGGGCACGTTCTTGTATGAATATTCAAGAAGACACCCTGATTACTCTGTATCCCTGTTGCTGAGAC
TTGCTAAGAAATATGAAGCCACTCTGGAAAAGTGCTGCGCTGAAGCCAATCCTCCCGCATGCTACGGCACAGTGCTTGCTGAATTTCAGCCTCTTGTAGAA
GAGCCTAAGAACTTGGTCAAAACCAACTGTGATCTTTACGAGAAGCTTGGAGAATATGGATTCCAAAATGCCATTCTAGTTCGCTACACCCAGAAAGCACC
TCAGGTGTCAACCCCAACTCTCGTGGAGGCTGCAAGAAACCTAGGAAGAGTGGGCACCAAGTGTTGTACACTTCCTGAAGATCAGAGACTGCCTTGTGTGG
AAGACTATCTGTCTGCAATCCTGAACCGTGTGTGTCTGCTGCATGAGAAGACCCCAGTGAGTGAGCATGTTACCAAGTGCTGTAGTGGATCCCTGGTGGAA
AGGCGGCCATGCTTCTCTGCTCTGACAGTTGATGAAACATATGTCCCCAAAGAGTTTAAAGCTGAGACCTTCACCTTCCACTCTGATATCTGCACACTTCC
AGAGAAGGAGAAGCAGATTAAGAAACAAACGGCTCTTGCTGAGCTGGTGAAGCACAAGCCCAAGGCTACAGCGGAGCAACTGAAGACTGTCATGGATGACT
TTGCACAGTTCCTGGATACATGTTGCAAGGCTGCTGACAAGGACACCTGCTTCTCGACTGAGGGTCCAAACCTTGTCACTAGATGCAAAGACGCCTTAGCC
*GGTGGAGGAGGCTCTGGTGGAGGCGGTAGCGGAGGCGGAGGGTCG*GATATCCAGATGACCCAGTCCCCGAGCTCCCTGTCCGCCTCTGTGGGCGATAGGGT
CACCATCACCTGCCGTGCCAGTCAGTCTTACGGTGGTGTAGCCTGGTATCAACAGAAACCAGGAAAAGCCCCGAAGCTTCTGATTTACTCTGCATCCTACC
TCTACTCTGGAGTCCCTTCTCGCTTCTCTGGTAGCCGTTCCGGGACGGATTTCACTCTGACCATCAGCAGTCTGCAGCCGGAAGACTTCGCAACTTATTAC
TGTCAGCAACCATCTCATCTGATCACGTTCGGACAGGGTACCGAGGTGGAGATCAAA*GGAGGGGGCGGTTCC*CACCATCACCACCATCACTGATAG

gWiz-LS-mouse SA-(Gly$_4$Ser)-V_H CK157-His$_6$

ATGGACATGAGAGTGCCTGCTCAGCTGCTGGGCCTGCTGCTGCTGTGGCTGCCTGGTGCTAGATGCGAAGCACACAAGAGTGAGATCGCCCATCGGTATAA
TGATTTGGGAGAACAACATTTCAAAGGCCTAGTCCTGATTGCCTTTTCCCAGTATCTCCAGAAATGCTCATACGATGAGCATGCCAAATTAGTGCAGGAAG
TAACAGACTTTGCAAAGACGTGTGTTGCCGATGAGTCTGCCGCCAACTGTGACAAATCCCTTCACACTCTTTTTGGAGATAAGTTGTGTGCCATTCCAAAC
CTCCGTGAAAACTATGGTGAACTGGCTGACTGCTGTACAAAACAAGAGCCCGAAAGAAACGAATGTTTCCTGCAACACAAAGATGACAACCCCAGCCTACC
ACCATTTGAAAGGCCAGAGGCTGAGGCCATGTGCACCTCCTTTAAGGAAAACCCAACCACCTTTATGGGACACTATTTGCATGAAGTTGCCAGAAGACATC
CTTATTTCTATGCCCCAGAACTTCTTTACTATGCTGAGCAGTACAATGAGATTCTGACCCAGTGTTGTGCAGAGGCTGACAAGGAAAGCTGCCTGACCCCG
AAGCTTGATGGTGTGAAGGAGAAAGCATTGGTCTCATCTGTCCGTCAGAGAATGAAGTGCTCCAGTATGCAGAAGTTTGGAGAGAGAGCTTTTAAAGCATG
GGCAGTAGCTCGTCTGAGCCAGACATTCCCCAATGCTGACTTTGCAGAAATCACCAAATTGGCAACAGACCTGACCAAAGTCAACAAGGAGTGCTGCCATG
GTGACCTGCTGGAATGCGCAGATGACAGGGCGGAACTTGCCAAGTACATGTGTGAAAACCAGGCGACTATCTCCAGCAAACTGCAGACTTGCTGCGATAAA
CCACTGTTGAAGAAAGCCCACTGTCTTAGTGAGGTGGAGCATGACACCATGCCTGCTGATCTGCCTGCCATTGCTGCTGATTTTGTTGAGGACCAGGAAGT
GTGCAAGAACTATGCTGAGGCCAAGGATGTCTTCCTGGGCACGTTCTTGTATGAATATTCAAGAAGACACCCTGATTACTCTGTATCCCTGTTGCTGAGAC
TTGCTAAGAAATATGAAGCCACTCTGGAAAAGTGCTGCGCTGAAGCCAATCCTCCCGCATGCTACGGCACAGTGCTTGCTGAATTTCAGCCTCTTGTAGAA
GAGCCTAAGAACTTGGTCAAAACCAACTGTGATCTTTACGAGAAGCTTGGAGAATATGGATTCCAAAATGCCATTCTAGTTCGCTACACCCAGAAAGCACC
TCAGGTGTCAACCCCAACTCTCGTGGAGGCTGCAAGAAACCTAGGAAGAGTGGGCACCAAGTGTTGTACACTTCCTGAAGATCAGAGACTGCCTTGTGTGG
AAGACTATCTGTCTGCAATCCTGAACCGTGTGTGTCTGCTGCATGAGAAGACCCCAGTGAGTGAGCATGTTACCAAGTGCTGTAGTGGATCCCTGGTGGAA
AGGCGGCCATGCTTCTCTGCTCTGACAGTTGATGAAACATATGTCCCCAAAGAGTTTAAAGCTGAGACCTTCACCTTCCACTCTGATATCTGCACACTTCC
AGAGAAGGAGAAGCAGATTAAGAAACAAACGGCTCTTGCTGAGCTGGTGAAGCACAAGCCCAAGGCTACAGCGGAGCAACTGAAGACTGTCATGGATGACT
TTGCACAGTTCCTGGATACATGTTGCAAGGCTGCTGACAAGGACACCTGCTTCTCGACTGAGGGTCCAAACCTTGTCACTAGATGCAAAGACGCCTTAGCC
*GGTGGAGGAGGCTCTGGTGGAGGCGGTAGCGGAGGCGGAGGGTCG***GCCGAGGTTCAGCTGGTGGAGTCTGGCGGTGGCCTGGTGCAGCCAGGGGGCTCACT**
CCGTTTGTCCTGTGCAGCTTCTGGCTCCAACCCCTACTACTACGGTGGTACGCACTGGGTGCGTCAGGCCCCGGGTGAGGAGCTGGAATGGGTTGCATCTA
TTGGTTCTTACCCTGGCTACACTGACTATGCCGATAGCGTCAAGGGCCGTTTCACTATAAGCGCAGACACATCCAAAAACACAGCCTACCTACAAATGAAC
AGCTTAAGAGCTGAGGACACTGCCGTCTATTATTGTGCTCGCCATTACTACTGGTACGATGCTACTGACTACTGGGGTCAAGGAACCCTGGTCACCGTCTC
CTCG*GGAGGGGGCGGTTCC***CACCATCACCACCATCAC****TGATAG**

gWiz-LS-mouse SA-(Gly$_4$Ser)$_3$-scFv (V_L-V_H) CK129-ds1 ($V_L100^{Q>C}$ / $V_H44^{G>C}$)-(Gly$_4$Ser)-His$_6$

ATGGACATGAGAGTGCCTGCTCAGCTGCTGGGCCTGCTGCTGCTGTGGCTGCCTGGTGCTAGATGCGAAGCACACAAGAGTGAGATCGCCCATCGGTATAA
TGATTTGGGAGAACAACATTTCAAAGGCCTAGTCCTGATTGCCTTTTCCCAGTATCTCCAGAAATGCTCATACGATGAGCATGCCAAATTAGTGCAGGAAG
TAACAGACTTTGCAAAGACGTGTGTTGCCGATGAGTCTGCCGCCAACTGTGACAAATCCCTTCACACTCTTTTTGGAGATAAGTTGTGTGCCATTCCAAAC
CTCCGTGAAAACTATGGTGAACTGGCTGACTGCTGTACAAAACAAGAGCCCGAAAGAAACGAATGTTTCCTGCAACACAAAGATGACAACCCCAGCCTACC
ACCATTTGAAAGGCCAGAGGCTGAGGCCATGTGCACCTCCTTTAAGGAAAACCCAACCACCTTTATGGGACACTATTTGCATGAAGTTGCCAGAAGACATC
CTTATTTCTATGCCCCAGAACTTCTTTACTATGCTGAGCAGTACAATGAGATTCTGACCCAGTGTTGTGCAGAGGCTGACAAGGAAAGCTGCCTGACCCCG
AAGCTTGATGGTGTGAAGGAGAAAGCATTGGTCTCATCTGTCCGTCAGAGAATGAAGTGCTCCAGTATGCAGAAGTTTGGAGAGAGAGCTTTTAAAGCATG
GGCAGTAGCTCGTCTGAGCCAGACATTCCCCAATGCTGACTTTGCAGAAATCACCAAATTGGCAACAGACCTGACCAAAGTCAACAAGGAGTGCTGCCATG

GTGACCTGCTGGAATGCGCAGATGACAGGGCGGAACTTGCCAAGTACATGTGTGAAAACCAGGCGACTATCTCCAGCAAACTGCAGACTTGCTGCGATAAA
CCACTGTTGAAGAAAGCCCACTGTCTTAGTGAGGTGGAGCATGACACCATGCCTGCTGATCTGCCTGCCATTGCTGCTGATTTTGTTGAGGACCAGGAAGT
GTGCAAGAACTATGCTGAGGCCAAGGATGTCTTCCTGGGCACGTTCTTGTATGAATATTCAAGAAGACACCCTGATTACTCTGTATCCCTGTTGCTGAGAC
TTGCTAAGAAATATGAAGCCACTCTGGAAAAGTGCTGCGCTGAAGCCAATCCTCCCGCATGCTACGGCACAGTGCTTGCTGAATTTCAGCCTCTTGTAGAA
GAGCCTAAGAACTTGGTCAAAACCAACTGTGATCTTTACGAGAAGCTTGGAGAATATGGATTCCAAAATGCCATTCTAGTTCGCTACACCCAGAAAGCACC
TCAGGTGTCAACCCCAACTCTCGTGGAGGCTGCAAGAAACCTAGGAAGAGTGGGCACCAAGTGTTGTACACTTCCTGAAGATCAGAGACTGCCTTGTGTGG
AAGACTATCTGTCTGCAATCCTGAACCGTGTGTGTCTGCTGCATGAGAAGACCCCAGTGAGTGAGCATGTTACCAAGTGCTGTAGTGGATCCCTGGTGGAA
AGGCGGCCATGCTTCTCTGCTCTGACAGTTGATGAAACATATGTCCCCAAAGAGTTTAAAGCTGAGACCTTCACCTTCCACTCTGATATCTGCACACTTCC
AGAGAAGGAGAAGCAGATTAAGAAACAAACGGCTCTTGCTGAGCTGGTGAAGCACAAGCCCAAGGCTACAGCGGAGCAACTGAAGACTGTCATGGATGACT
TTGCACAGTTCCTGGATACATGTTGCAAGGCTGCTGACAAGGACACCTGCTTCTCGACTGAGGGTCCAAACCTTGTCACTAGATGCAAAGACGCCTTAGCC
*GGTGGAGGAGGCTCTGGTGGAGGCGGTAGCGGAGGCGGAGGGTCG*GATATCCAGATGACCCAGTCCCCGAGCCCCCTGTCCGCCTCTGTGGGCGATAGGGT
CACCATCACCTGCCGTGCCAGTCAGTACGGTGGTTACGTAGCCTGGTATCAACAGAAACCAGGAAAAGCTCCGAAGCTTCTGATTTACGGTGCATCCCTTC
TCTACTCTGGAGTCCCTTCTCGCTTCTCTGGTGGCCGTTCCGGGACGGATTTCACTCTGACCATCAGCAGTCTGCAGCCGGAAGACTTCGCAACTTATTAC
TGTCAGCGAGGTCATGCTCTGATCACGTTCGGA*TGC*GGTACCAAGGTGGAGATCGAAGGTACTACTGCCGCTAGTGGTAGTAGTGGTGGCAGTAGCAGTGG
TGCCGAGGTTCAGCTGGTGGAGTCTGGCGGTGGCCTGGTGCAGCCAGGGGGCTCACTCCGTTTATCCTGTGCAGCTTCTGGCTTCAACATCTCTTCTTACG
GTTCTATGCACTGGGTGCGTCAGGCCCCGGGTAAG*T*GCCTGGAATGGGTTGCATCTATTTACCCTTACTCTAGCTCTACTTACTATGCCGATAGCGTCAAG
GGCCGTTTCACTATAAGCGCAGACACATCCAAAAACACAGCCTACCTACAAATGAACAGCTTAAGAGCTGAGGACACTGCCGTCTATTATTGTGCTCGTGG
TTACGGTCCGTGGTACGCTTACTCTTACTTCGCTTTGGACTACTGGGGTCAAGGAACCCTGGTCACCGTCTCCTCG*GGAGGGGGCGGTTCC*CACCATCACC
ACCATCACTGATAG

gWiz-LS-mouse SA-$(Gly_4Ser)_3$*-scFv* $(V_L\text{-}V_H)$ *CK129-ds2* $(V_L43^{A>C}\ /\ V_H105^{Q>C})$*-*$(Gly_4Ser)$*-*$His_6$

ATGGACATGAGAGTGCCTGCTCAGCTGCTGGGCCTGCTGCTGCTGTGGCTGCCTGGTGCTAGATGCGAAGCACACAAGAGTGAGATCGCCCATCGGTATAA
TGATTTGGGAGAACAACATTTCAAAGGCCTAGTCCTGATTGCCTTTTCCCAGTATCTCCAGAAATGCTCATACGATGAGCATGCCAAATTAGTGCAGGAAG
TAACAGACTTTGCAAAGACGTGTGTTGCCGATGAGTCTGCCGCCAACTGTGACAAATCCCTTCACACTCTTTTTGGAGATAAGTTGTGTGCCATTCCAAAC
CTCCGTGAAAACTATGGTGAACTGGCTGACTGCTGTACAAAACAAGAGCCCGAAAGAAACGAATGTTTCCTGCAACACAAAGATGACAACCCCAGCCTACC
ACCATTTGAAAGGCCAGAGGCTGAGGCCATGTGCACCTCCTTTAAGGAAAACCCAACCACCTTTATGGGACACTATTTGCATGAAGTTGCCAGAAGACATC
CTTATTTCTATGCCCCAGAACTTCTTTACTATGCTGAGCAGTACAATGAGATTCTGACCCAGTGTTGTGCAGAGGCTGACAAGGAAAGCTGCCTGACCCCG
AAGCTTGATGGTGTGAAGGAGAAAGCATTGGTCTCATCTGTCCGTCAGAGAATGAAGTGCTCCAGTATGCAGAAGTTTGGAGAGAGAGCTTTTAAAGCATG
GGCAGTAGCTCGTCTGAGCCAGACATTCCCCAATGCTGACTTTGCAGAAATCACCAAATTGGCAACAGACCTGACCAAAGTCAACAAGGAGTGCTGCCATG
GTGACCTGCTGGAATGCGCAGATGACAGGGCGGAACTTGCCAAGTACATGTGTGAAAACCAGGCGACTATCTCCAGCAAACTGCAGACTTGCTGCGATAAA
CCACTGTTGAAGAAAGCCCACTGTCTTAGTGAGGTGGAGCATGACACCATGCCTGCTGATCTGCCTGCCATTGCTGCTGATTTTGTTGAGGACCAGGAAGT
GTGCAAGAACTATGCTGAGGCCAAGGATGTCTTCCTGGGCACGTTCTTGTATGAATATTCAAGAAGACACCCTGATTACTCTGTATCCCTGTTGCTGAGAC
TTGCTAAGAAATATGAAGCCACTCTGGAAAAGTGCTGCGCTGAAGCCAATCCTCCCGCATGCTACGGCACAGTGCTTGCTGAATTTCAGCCTCTTGTAGAA
GAGCCTAAGAACTTGGTCAAAACCAACTGTGATCTTTACGAGAAGCTTGGAGAATATGGATTCCAAAATGCCATTCTAGTTCGCTACACCCAGAAAGCACC

TCAGGTGTCAACCCCAACTCTCGTGGAGGCTGCAAGAAACCTAGGAAGAGTGGGCACCAAGTGTTGTACACTTCCTGAAGATCAGAGACTGCCTTGTGTGG
AAGACTATCTGTCTGCAATCCTGAACCGTGTGTGTCTGCTGCATGAGAAGACCCCAGTGAGTGAGCATGTTACCAAGTGCTGTAGTGGATCCCTGGTGGAA
AGGCGGCCATGCTTCTCTGCTCTGACAGTTGATGAAACATATGTCCCCAAAGAGTTTAAAGCTGAGACCTTCACCTTCCACTCTGATATCTGCACACTTCC
AGAGAAGGAGAAGCAGATTAAGAAACAAACGGCTCTTGCTGAGCTGGTGAAGCACAAGCCCAAGGCTACAGCGGAGCAACTGAAGACTGTCATGGATGACT
TTGCACAGTTCCTGGATACATGTTGCAAGGCTGCTGACAAGGACACCTGCTTCTCGACTGAGGGTCCAAACCTTGTCACTAGATGCAAAGACGCCTTAGCC
*GGTGGAGGAGGCTCTGGTGGAGGCGGTAGCGGAGGCGGAGGGTCG***GATATCCAGATGACCCAGTCCCCGAGCCCCCTGTCCGCCTCTGTGGGCGATAGGGT**
CACCATCACCTGCCGTGCCAGTCAGTACGGTGGTTACGTAGCCTGGTATCAACAGAAACCAGGAAAA***TGC*****CCGAAGCTTCTGATTTACGGTGCATCCCTTC**
TCTACTCTGGAGTCCCTTCTCGCTTCTCTGGTGGCCGTTCCGGGACGGATTTCACTCTGACCATCAGCAGTCTGCAGCCGGAAGACTTCGCAACTTATTAC
TGTCAGCGAGGTCATGCTCTGATCACGTTCGGACAGGGTACCAAGGTGGAGATCGAAGGTACTACTGCCGCTAGTGGTAGTAGTGGTGGCAGTAGCAGTGG
TGCCGAGGTTCAGCTGGTGGAGTCTGGCGGTGGCCTGGTGCAGCCAGGGGGCTCACTCCGTTTATCCTGTGCAGCTTCTGGCTTCAACATCTCTTCTTACG
GTTCTATGCACTGGGTGCGTCAGGCCCCGGGTAAGGGCCTGGAATGGGTTGCATCTATTTACCCTTACTCTAGCTCTACTTACTATGCCGATAGCGTCAAG
GGCCGTTTCACTATAAGCGCAGACACATCCAAAAACACAGCCTACCTACAAATGAACAGCTTAAGAGCTGAGGACACTGCCGTCTATTATTGTGCTCGTGG
TTACGGTCCGTGGTACGCTTACTCTTACTTCGCTTTGGACTACTGGGGT***TGC*****GGAACCCTGGTCACCGTCTCCTCG***GGAGGGGGCGGTTCC***CACCATCACC**
ACCATCACTGATAG

gWiz-LS-mouse SA-(Gly$_4$Ser)$_3$-scFv (V$_H$-V$_L$) sm3E-ds (V$_H$44$^{R>G}$ / V$_L$100$^{G>C}$)-(Gly$_4$Ser)-His$_6$

ATGGACATGAGAGTGCCTGCTCAGCTGCTGGGCCTGCTGCTGCTGTGGCTGCCTGGTGCTAGATGCGAAGCACACAAGAGTGAGATCGCCCATCGGTATAA
TGATTTGGGAGAACAACATTTCAAAGGCCTAGTCCTGATTGCCTTTTCCCAGTATCTCCAGAAATGCTCATACGATGAGCATGCCAAATTAGTGCAGGAAG
TAACAGACTTTGCAAAGACGTGTGTTGCCGATGAGTCTGCCGCCAACTGTGACAAATCCCTTCACACTCTTTTTGGAGATAAGTTGTGTGCCATTCCAAAC
CTCCGTGAAAACTATGGTGAACTGGCTGACTGCTGTACAAAACAAGAGCCCGAAAGAAACGAATGTTTCCTGCAACACAAAGATGACAACCCCAGCCTACC
ACCATTTGAAAGGCCAGAGGCTGAGGCCATGTGCACCTCCTTTAAGGAAAACCCAACCACCTTTATGGGACACTATTTGCATGAAGTTGCCAGAAGACATC
CTTATTTCTATGCCCCAGAACTTCTTTACTATGCTGAGCAGTACAATGAGATTCTGACCCAGTGTTGTGCAGAGGCTGACAAGGAAAGCTGCCTGACCCCG
AAGCTTGATGGTGTGAAGGAGAAAGCATTGGTCTCATCTGTCCGTCAGAGAATGAAGTGCTCCAGTATGCAGAAGTTTGGAGAGAGAGCTTTTAAAGCATG
GGCAGTAGCTCGTCTGAGCCAGACATTCCCCAATGCTGACTTTGCAGAAATCACCAAATTGGCAACAGACCTGACCAAAGTCAACAAGGAGTGCTGCCATG
GTGACCTGCTGGAATGCGCAGATGACAGGGCGGAACTTGCCAAGTACATGTGTGAAAACCAGGCGACTATCTCCAGCAAACTGCAGACTTGCTGCGATAAA
CCACTGTTGAAGAAAGCCCACTGTCTTAGTGAGGTGGAGCATGACACCATGCCTGCTGATCTGCCTGCCATTGCTGCTGATTTTGTTGAGGACCAGGAAGT
GTGCAAGAACTATGCTGAGGCCAAGGATGTCTTCCTGGGCACGTTCTTGTATGAATATTCAAGAAGACACCCTGATTACTCTGTATCCCTGTTGCTGAGAC
TTGCTAAGAAATATGAAGCCACTCTGGAAAAGTGCTGCGCTGAAGCCAATCCTCCCGCATGCTACGGCACAGTGCTTGCTGAATTTCAGCCTCTTGTAGAA
GAGCCTAAGAACTTGGTCAAAACCAACTGTGATCTTTACGAGAAGCTTGGAGAATATGGATTCCAAAATGCCATTCTAGTTCGCTACACCCAGAAAGCACC
TCAGGTGTCAACCCCAACTCTCGTGGAGGCTGCAAGAAACCTAGGAAGAGTGGGCACCAAGTGTTGTACACTTCCTGAAGATCAGAGACTGCCTTGTGTGG
AAGACTATCTGTCTGCAATCCTGAACCGTGTGTGTCTGCTGCATGAGAAGACCCCAGTGAGTGAGCATGTTACCAAGTGCTGTAGTGGATCCCTGGTGGAA
AGGCGGCCATGCTTCTCTGCTCTGACAGTTGATGAAACATATGTCCCCAAAGAGTTTAAAGCTGAGACCTTCACCTTCCACTCTGATATCTGCACACTTCC
AGAGAAGGAGAAGCAGATTAAGAAACAAACGGCTCTTGCTGAGCTGGTGAAGCACAAGCCCAAGGCTACAGCGGAGCAACTGAAGACTGTCATGGATGACT
TTGCACAGTTCCTGGATACATGTTGCAAGGCTGCTGACAAGGACACCTGCTTCTCGACTGAGGGTCCAAACCTTGTCACTAGATGCAAAGACGCCTTAGCC

*GGTGGAGGAGGCTCTGGTGGAGGCGGTAGCGGAGGCGGAGGGTCG***CAAGTTAAACTGGAACAGTCCGGTGCTGAAGTTGTCAAACCAGGTGCTTCCGTGAA**
GTTGTCCTGTAAAGCCTCTGGTTTTAACATCAAGGATTCGTATATGCATTGGTTGAGACAAGGGCCAGGACAA***TGT*****TTGGAATGGATTGGCTGGATTGATC**
CAGAGAATGGTGATACCGAGTACGCTCCTAAATTTCAGGGAAAGGCTACTTTTACTACCGACACTTCCGCTAATACCGCATACTTGGGCTTATCTTCCTTG
AGACCAGAGGACACTGCCGTATACTACTGCAACGAAGGGACACCAACTGGTCCTTACTATTTCGACTACTGGGGACAAGGTACCTTAGTTACTGTCTCTAG
CGGTGGCGGAGGTTCAGGCGGTGGAGGGTCTGGAGGTGGCGGTAGTGAAAATGTGCTGACCCAATCTCCAAGCTCCATGTCTGTTTCTGTTGGCGATAGAG
TAACCATCGCTTGTAGCGCATCCTCTAGTGTCCCATATATGCACTGGCTTCAACAGAAGCCAGGTAAAAGCCCAAAGTTGTTGATTTATTTGACATCCAAC
TTGGCTTCTGGAGTGCCTTCAAGGTTTTCTGGTTCCGGCTCAGGAACCGATTATAGTTTGACTATTAGCTCAGTGCAGCCAGAGGATGCTGCAACCTACTA
TTGCCAGCAAAGGTCCTCATATCCACTGACTTTCGGG***TGT*****GGAACGAAGTTGGAAATCAAG***GGAGGGGGCGGTTCC***CACCATCACCACCATCACTGATAG**

Amino-acid sequences of antibody single-chain variable fragments (scFv) fused to the C-terminus of mouse serum albumin (SA): ^{N}SA-scFv^{C}

Amino acid sequences of translated polypeptides are shown. The sequences include the secretory leader peptide (LS, in grey underlined), the mouse serum albumin (SA, in grey), a long flexible $(Gly_4Ser)_3$ linker (in black italic and underlined), the antibody single-chain variable fragment (scFv, in blue bold), a short flexible (Gly_4Ser) linker (in black italic and underlined) and the hexa-histidine tag (His_6, in red bold). Cysteine residues introduced to form stabilizing intermolecular disulfide bridges (ds1 and ds2) are shown in in orange italic bold.

LS-mouse SA-$(Gly_4Ser)_3$-scFv $(V_L$-$V_H)$ CK138-(Gly_4Ser)-His_6

MDMRVPAQLLGLLLLWLPGARCEAHKSEIAHRYNDLGEQHFKGLVLIAFSQYLQKCSYDEHAKLVQEVTDFAKTCVADESAANCDKSLHTLFGDKLCAIPN
LRENYGELADCCTKQEPERNECFLQHKDDNPSLPPFERPEAEAMCTSFKENPTTFMGHYLHEVARRHPYFYAPELLYYAEQYNEILTQCCAEADKESCLTP
KLDGVKEKALVSSVRQRMKCSSMQKFGERAFKAWAVARLSQTFPNADFAEITKLATDLTKVNKECCHGDLLECADDRAELAKYMCENQATISSKLQTCCDK
PLLKKAHCLSEVEHDTMPADLPAIAADFVEDQEVCKNYAEAKDVFLGTFLYEYSRRHPDYSVSLLLRLAKKYEATLEKCCAEANPPACYGTVLAEFQPLVE
EPKNLVKTNCDLYEKLGEYGFQNAILVRYTQKAPQVSTPTLVEAARNLGRVGTKCCTLPEDQRLPCVEDYLSAILNRVCLLHEKTPVSEHVTKCCSGSLVE
RRPCFSALTVDETYVPKEFKAETFTFHSDICTLPEKEKQIKKQTALAELVKHKPKATAEQLKTVMDDFAQFLDTCCKAADKDTCFSTEGPNLVTRCKDALA
*GGGGSGGGGSGGGGS*AS**AIQMTRSPSSLSASVGDRVTITCRASQYHDGSAAWYQQKPGKAPKLLIYGASYLYSGVPSRFSGSRSGTDFTLTISSLQPEDFA**
TYYCQQSSYSLITFGQGTKVEIKGTTAASGSSGGSSSGAEVQLVESDGGLVQPGGSLRLSCAASGFNLSYYGMHWVRQAPGKGLEWVAYIASYPGYTSYAD
SVKGRFTISADTSKNTAYLQMNSLRAEDTAVYYCARSGYSYSPYYSWFSAGMNYWGQGALVTVSS*GGGGS***HHHHHH**--

LS-mouse SA-$(Gly_4Ser)_3$-scFv $(V_L$-$V_H)$ CK157-(Gly_4Ser)-His_6

MDMRVPAQLLGLLLLWLPGARCEAHKSEIAHRYNDLGEQHFKGLVLIAFSQYLQKCSYDEHAKLVQEVTDFAKTCVADESAANCDKSLHTLFGDKLCAIPN
LRENYGELADCCTKQEPERNECFLQHKDDNPSLPPFERPEAEAMCTSFKENPTTFMGHYLHEVARRHPYFYAPELLYYAEQYNEILTQCCAEADKESCLTP
KLDGVKEKALVSSVRQRMKCSSMQKFGERAFKAWAVARLSQTFPNADFAEITKLATDLTKVNKECCHGDLLECADDRAELAKYMCENQATISSKLQTCCDK
PLLKKAHCLSEVEHDTMPADLPAIAADFVEDQEVCKNYAEAKDVFLGTFLYEYSRRHPDYSVSLLLRLAKKYEATLEKCCAEANPPACYGTVLAEFQPLVE
EPKNLVKTNCDLYEKLGEYGFQNAILVRYTQKAPQVSTPTLVEAARNLGRVGTKCCTLPEDQRLPCVEDYLSAILNRVCLLHEKTPVSEHVTKCCSGSLVE
RRPCFSALTVDETYVPKEFKAETFTFHSDICTLPEKEKQIKKQTALAELVKHKPKATAEQLKTVMDDFAQFLDTCCKAADKDTCFSTEGPNLVTRCKDALA
*GGGGSGGGGSGGGGS*AS**DIQMTQSPSSLSASVGDRVTITCRASQSYGGVAWYQQKPGKAPKLLIYSASYLYSGVPSRFSGSRSGTDFTLTISSLQPEDFAT**

YYCQQPSHLITFGQGTEVEIKGTTAASGSSGGSSSGAEVQLVESGGGLVQPGGSLRLSCAASGSNPYYYGGTHWVRQAPGEELEWVASIGSYPGYTDYADS
VKGRFTISADTSKNTAYLQMNSLRAEDTAVYYCARHYYWYDATDYWGQGTLVTVSS*GGGGS***HHHHHH**--

LS-mouse SA-(Gly$_4$Ser)$_3$-scFv (V_L-V_H) CK129-(Gly$_4$Ser)-His$_6$

MDMRVPAQLLGLLLLWLPGARCEAHKSEIAHRYNDLGEQHFKGLVLIAFSQYLQKCSYDEHAKLVQEVTDFAKTCVADESAANCDKSLHTLFGDKLCAIPN
LRENYGELADCCTKQEPERNECFLQHKDDNPSLPPFERPEAEAMCTSFKENPTTFMGHYLHEVARRHPYFYAPELLYYAEQYNEILTQCCAEADKESCLTP
KLDGVKEKALVSSVRQRMKCSSMQKFGERAFKAWAVARLSQTFPNADFAEITKLATDLTKVNKECCHGDLLECADDRAELAKYMCENQATISSKLQTCCDK
PLLKKAHCLSEVEHDTMPADLPAIAADFVEDQEVCKNYAEAKDVFLGTFLYEYSRRHPDYSVSLLLRLAKKYEATLEKCCAEANPPACYGTVLAEFQPLVE
EPKNLVKTNCDLYEKLGEYGFQNAILVRYTQKAPQVSTPTLVEAARNLGRVGTKCCTLPEDQRLPCVEDYLSAILNRVCLLHEKTPVSEHVTKCCSGSLVE
RRPCFSALTVDETYVPKEFKAETFTFHSDICTLPEKEKQIKKQTALAELVKHKPKATAEQLKTVMDDFAQFLDTCCKAADKDTCFSTEGPNLVTRCKDALA
*GGGGSGGGGSGGGGS*AS**DIQMTQSPSPLSASVGDRVTITCRASQYGGYVAWYQQKPGKAPKLLIYGASLLYSGVPSRFSGGRSGTDFTLTISSLQPEDFAT**
YYCQRGHALITFGQGTKVEIEGTTAASGSSGGSSSGAEVQLVESGGGLVQPGGSLRLSCAASGFNISSYGSMHWVRQAPGKGLEWVASIYPYSSSTYYADS
VKGRFTISADTSKNTAYLQMNSLRAEDTAVYYCARGYGPWYAYSYFALDYWGQGTLVTVSS*GGGGS***HHHHHH**--

LS-mouse SA-(Gly$_4$Ser)$_3$-scFv (V_L-V_H) CK138-ds1 ($V_L100^{Q>C}$ / $V_H44^{G>C}$)-(Gly$_4$Ser)-His$_6$

MDMRVPAQLLGLLLLWLPGARCEAHKSEIAHRYNDLGEQHFKGLVLIAFSQYLQKCSYDEHAKLVQEVTDFAKTCVADESAANCDKSLHTLFGDKLCAIPN
LRENYGELADCCTKQEPERNECFLQHKDDNPSLPPFERPEAEAMCTSFKENPTTFMGHYLHEVARRHPYFYAPELLYYAEQYNEILTQCCAEADKESCLTP
KLDGVKEKALVSSVRQRMKCSSMQKFGERAFKAWAVARLSQTFPNADFAEITKLATDLTKVNKECCHGDLLECADDRAELAKYMCENQATISSKLQTCCDK
PLLKKAHCLSEVEHDTMPADLPAIAADFVEDQEVCKNYAEAKDVFLGTFLYEYSRRHPDYSVSLLLRLAKKYEATLEKCCAEANPPACYGTVLAEFQPLVE
EPKNLVKTNCDLYEKLGEYGFQNAILVRYTQKAPQVSTPTLVEAARNLGRVGTKCCTLPEDQRLPCVEDYLSAILNRVCLLHEKTPVSEHVTKCCSGSLVE
RRPCFSALTVDETYVPKEFKAETFTFHSDICTLPEKEKQIKKQTALAELVKHKPKATAEQLKTVMDDFAQFLDTCCKAADKDTCFSTEGPNLVTRCKDALA
*GGGGSGGGGSGGGGS*AS**AIQMTRSPSSLSASVGDRVTITCRASQYHDGSAAWYQQKPGKAPKLLIYGASYLYSGVPSRFSGSRSGTDFTLTISSLQPEDFA**
TYYCQQSSYSLITFG*C*GTKVEIKGTTAASGSSGGSSSGAEVQLVESDGGLVQPGGSLRLSCAASGFNLSYYGMHWVRQAPGK*C*LEWVAYIASYPGYTSYAD
SVKGRFTISADTSKNTAYLQMNSLRAEDTAVYYCARSGYSYSPYYSWFSAGMNYWGQGALVTVSS*GGGGS***HHHHHH**--

LS-mouse SA-(Gly$_4$Ser)$_3$-scFv (V_L-V_H) CK138-ds2 ($V_L43^{A>C}$ / $V_H105^{Q>C}$)-(Gly$_4$Ser)-His$_6$

MDMRVPAQLLGLLLLWLPGARCEAHKSEIAHRYNDLGEQHFKGLVLIAFSQYLQKCSYDEHAKLVQEVTDFAKTCVADESAANCDKSLHTLFGDKLCAIPN
LRENYGELADCCTKQEPERNECFLQHKDDNPSLPPFERPEAEAMCTSFKENPTTFMGHYLHEVARRHPYFYAPELLYYAEQYNEILTQCCAEADKESCLTP
KLDGVKEKALVSSVRQRMKCSSMQKFGERAFKAWAVARLSQTFPNADFAEITKLATDLTKVNKECCHGDLLECADDRAELAKYMCENQATISSKLQTCCDK
PLLKKAHCLSEVEHDTMPADLPAIAADFVEDQEVCKNYAEAKDVFLGTFLYEYSRRHPDYSVSLLLRLAKKYEATLEKCCAEANPPACYGTVLAEFQPLVE
EPKNLVKTNCDLYEKLGEYGFQNAILVRYTQKAPQVSTPTLVEAARNLGRVGTKCCTLPEDQRLPCVEDYLSAILNRVCLLHEKTPVSEHVTKCCSGSLVE
RRPCFSALTVDETYVPKEFKAETFTFHSDICTLPEKEKQIKKQTALAELVKHKPKATAEQLKTVMDDFAQFLDTCCKAADKDTCFSTEGPNLVTRCKDALA

GGGGSGGGGSGGGGSASAIQMTRSPSSLSASVGDRVTITCRASQYHDGSAAWYQQKPGKCPKLLIYGASYLYSGVPSRFSGSRSGTDFTLTISSLQPEDFA
TYYCQQSSYSLITFGQGTKVEIKGTTAASGSSGGSSSGAEVQLVESDGGLVQPGGSLRLSCAASGFNLSYYGMHWVRQAPGKGLEWVAYIASYPGYTSYAD
SVKGRFTISADTSKNTAYLQMNSLRAEDTAVYYCARSGYSYSPYYSWFSAGMNYWGCGALVTVSSGGGGSHHHHHH--

LS-mouse SA-(Gly$_4$Ser)$_3$-scFv (V$_L$-V$_H$) CK157-ds1 (V$_L$100$^{Q>C}$ / V$_H$44$^{E>C}$)-(Gly$_4$Ser)-His$_6$

MDMRVPAQLLGLLLLWLPGARCEAHKSEIAHRYNDLGEQHFKGLVLIAFSQYLQKCSYDEHAKLVQEVTDFAKTCVADESAANCDKSLHTLFGDKLCAIPN
LRENYGELADCCTKQEPERNECFLQHKDDNPSLPPFERPEAEAMCTSFKENPTTFMGHYLHEVARRHPYFYAPELLYYAEQYNEILTQCCAEADKESCLTP
KLDGVKEKALVSSVRQRMKCSSMQKFGERAFKAWAVARLSQTFPNADFAEITKLATDLTKVNKECCHGDLLECADDRAELAKYMCENQATISSKLQTCCDK
PLLKKAHCLSEVEHDTMPADLPAIAADFVEDQEVCKNYAEAKDVFLGTFLYEYSRRHPDYSVSLLLRLAKKYEATLEKCCAEANPPACYGTVLAEFQPLVE
EPKNLVKTNCDLYEKLGEYGFQNAILVRYTQKAPQVSTPTLVEAARNLGRVGTKCCTLPEDQRLPCVEDYLSAILNRVCLLHEKTPVSEHVTKCCSGSLVE
RRPCFSALTVDETYVPKEFKAETFTFHSDICTLPEKEKQIKKQTALAELVKHKPKATAEQLKTVMDDFAQFLDTCCKAADKDTCFSTEGPNLVTRCKDALA
GGGGSGGGGSGGGGSASDIQMTQSPSSLSASVGDRVTITCRASQSYGGVAWYQQKPGKAPKLLIYSASYLYSGVPSRFSGSRSGTDFTLTISSLQPEDFAT
YYCQQPSHLITFGCGTEVEIKGTTAASGSSGGSSSGAEVQLVESGGGLVQPGGSLRLSCAASGSNPYYYGGTHWVRQAPGECLEWVASIGSYPGYTDYADS
VKGRFTISADTSKNTAYLQMNSLRAEDTAVYYCARHYYWYDATDYWGQGTLVTVSSGGGGSHHHHHH--

LS-mouse SA-(Gly$_4$Ser)$_3$-scFv (V$_L$-V$_H$) CK157-ds2 (V$_L$43$^{A>C}$ / V$_H$105$^{Q>C}$)-(Gly$_4$Ser)-His$_6$

MDMRVPAQLLGLLLLWLPGARCEAHKSEIAHRYNDLGEQHFKGLVLIAFSQYLQKCSYDEHAKLVQEVTDFAKTCVADESAANCDKSLHTLFGDKLCAIPN
LRENYGELADCCTKQEPERNECFLQHKDDNPSLPPFERPEAEAMCTSFKENPTTFMGHYLHEVARRHPYFYAPELLYYAEQYNEILTQCCAEADKESCLTP
KLDGVKEKALVSSVRQRMKCSSMQKFGERAFKAWAVARLSQTFPNADFAEITKLATDLTKVNKECCHGDLLECADDRAELAKYMCENQATISSKLQTCCDK
PLLKKAHCLSEVEHDTMPADLPAIAADFVEDQEVCKNYAEAKDVFLGTFLYEYSRRHPDYSVSLLLRLAKKYEATLEKCCAEANPPACYGTVLAEFQPLVE
EPKNLVKTNCDLYEKLGEYGFQNAILVRYTQKAPQVSTPTLVEAARNLGRVGTKCCTLPEDQRLPCVEDYLSAILNRVCLLHEKTPVSEHVTKCCSGSLVE
RRPCFSALTVDETYVPKEFKAETFTFHSDICTLPEKEKQIKKQTALAELVKHKPKATAEQLKTVMDDFAQFLDTCCKAADKDTCFSTEGPNLVTRCKDALA
GGGGSGGGGSGGGGSASDIQMTQSPSSLSASVGDRVTITCRASQSYGGVAWYQQKPGKCPKLLIYSASYLYSGVPSRFSGSRSGTDFTLTISSLQPEDFAT
YYCQQPSHLITFGQGTEVEIKGTTAASGSSGGSSSGAEVQLVESGGGLVQPGGSLRLSCAASGSNPYYYGGTHWVRQAPGEELEWVASIGSYPGYTDYADS
VKGRFTISADTSKNTAYLQMNSLRAEDTAVYYCARHYYWYDATDYWGCGTLVTVSSGGGGSHHHHHH--

LS-mouse SA-(Gly$_4$Ser)-V$_L$ CK157-His$_6$

MDMRVPAQLLGLLLLWLPGARCEAHKSEIAHRYNDLGEQHFKGLVLIAFSQYLQKCSYDEHAKLVQEVTDFAKTCVADESAANCDKSLHTLFGDKLCAIPN
LRENYGELADCCTKQEPERNECFLQHKDDNPSLPPFERPEAEAMCTSFKENPTTFMGHYLHEVARRHPYFYAPELLYYAEQYNEILTQCCAEADKESCLTP
KLDGVKEKALVSSVRQRMKCSSMQKFGERAFKAWAVARLSQTFPNADFAEITKLATDLTKVNKECCHGDLLECADDRAELAKYMCENQATISSKLQTCCDK
PLLKKAHCLSEVEHDTMPADLPAIAADFVEDQEVCKNYAEAKDVFLGTFLYEYSRRHPDYSVSLLLRLAKKYEATLEKCCAEANPPACYGTVLAEFQPLVE
EPKNLVKTNCDLYEKLGEYGFQNAILVRYTQKAPQVSTPTLVEAARNLGRVGTKCCTLPEDQRLPCVEDYLSAILNRVCLLHEKTPVSEHVTKCCSGSLVE

RRPCFSALTVDETYVPKEFKAETFTFHSDICTLPEKEKQIKKQTALAELVKHKPKATAEQLKTVMDDFAQFLDTCCKAADKDTCFSTEGPNLVTRCKDALA
*GGGGSGGGGSGGGGS*AS**DIQMTQSPSSLSASVGDRVTITCRASQSYGGVAWYQQKPGKAPKLLIYSASYLYSGVPSRFSGSRSGTDFTLTISSLQPEDFAT**
YYCQQPSHLITFGQGTEVEIK*GGGGS***HHHHHH**--

LS-mouse SA-(Gly$_4$Ser)-V$_H$ CK157-His$_6$

MDMRVPAQLLGLLLLWLPGARCEAHKSEIAHRYNDLGEQHFKGLVLIAFSQYLQKCSYDEHAKLVQEVTDFAKTCVADESAANCDKSLHTLFGDKLCAIPN
LRENYGELADCCTKQEPERNECFLQHKDDNPSLPPFERPEAEAMCTSFKENPTTFMGHYLHEVARRHPYFYAPELLYYAEQYNEILTQCCAEADKESCLTP
KLDGVKEKALVSSVRQRMKCSSMQKFGERAFKAWAVARLSQTFPNADFAEITKLATDLTKVNKECCHGDLLECADDRAELAKYMCENQATISSKLQTCCDK
PLLKKAHCLSEVEHDTMPADLPAIAADFVEDQEVCKNYAEAKDVFLGTFLYEYSRRHPDYSVSLLLRLAKKYEATLEKCCAEANPPACYGTVLAEFQPLVE
EPKNLVKTNCDLYEKLGEYGFQNAILVRYTQKAPQVSTPTLVEAARNLGRVGTKCCTLPEDQRLPCVEDYLSAILNRVCLLHEKTPVSEHVTKCCSGSLVE
RRPCFSALTVDETYVPKEFKAETFTFHSDICTLPEKEKQIKKQTALAELVKHKPKATAEQLKTVMDDFAQFLDTCCKAADKDTCFSTEGPNLVTRCKDALA
*GGGGSGGGGSGGGGS*AS**AEVQLVESGGGLVQPGGSLRLSCAASGSNPYYYGGTHWVRQAPGEELEWVASIGSYPGYTDYADSVKGRFTISADTSKNTAYLQ**
MNSLRAEDTAVYYCARHYYWYDATDYWGQGTLVTVSS*GGGGS***HHHHHH**--

LS-mouse SA-(Gly$_4$Ser)$_3$-scFv (V$_L$-V$_H$) CK129-ds1 (V$_L$100$^{Q>C}$ / V$_H$44$^{G>C}$)-(Gly$_4$Ser)-His$_6$

MDMRVPAQLLGLLLLWLPGARCEAHKSEIAHRYNDLGEQHFKGLVLIAFSQYLQKCSYDEHAKLVQEVTDFAKTCVADESAANCDKSLHTLFGDKLCAIPN
LRENYGELADCCTKQEPERNECFLQHKDDNPSLPPFERPEAEAMCTSFKENPTTFMGHYLHEVARRHPYFYAPELLYYAEQYNEILTQCCAEADKESCLTP
KLDGVKEKALVSSVRQRMKCSSMQKFGERAFKAWAVARLSQTFPNADFAEITKLATDLTKVNKECCHGDLLECADDRAELAKYMCENQATISSKLQTCCDK
PLLKKAHCLSEVEHDTMPADLPAIAADFVEDQEVCKNYAEAKDVFLGTFLYEYSRRHPDYSVSLLLRLAKKYEATLEKCCAEANPPACYGTVLAEFQPLVE
EPKNLVKTNCDLYEKLGEYGFQNAILVRYTQKAPQVSTPTLVEAARNLGRVGTKCCTLPEDQRLPCVEDYLSAILNRVCLLHEKTPVSEHVTKCCSGSLVE
RRPCFSALTVDETYVPKEFKAETFTFHSDICTLPEKEKQIKKQTALAELVKHKPKATAEQLKTVMDDFAQFLDTCCKAADKDTCFSTEGPNLVTRCKDALA
*GGGGSGGGGSGGGGS*AS**DIQMTQSPSPLSASVGDRVTITCRASQYGGYVAWYQQKPGKAPKLLIYGASLLYSGVPSRFSGGRSGTDFTLTISSLQPEDFAT**
YYCQRGHALITFG*C*GTKVEIEGTTAASGSSGGSSSGAEVQLVESGGGLVQPGGSLRLSCAASGFNISSYGSMHWVRQAPGK*C*LEWVASIYPYSSSTYYADS
VKGRFTISADTSKNTAYLQMNSLRAEDTAVYYCARGYGPWYAYSYFALDYWGQGTLVTVSS*GGGGS***HHHHHH**--

LS-mouse SA-(Gly$_4$Ser)$_3$-scFv (V$_L$-V$_H$) CK129-ds2 (V$_L$43$^{A>C}$ / V$_H$105$^{Q>C}$)-(Gly$_4$Ser)-His$_6$

MDMRVPAQLLGLLLLWLPGARCEAHKSEIAHRYNDLGEQHFKGLVLIAFSQYLQKCSYDEHAKLVQEVTDFAKTCVADESAANCDKSLHTLFGDKLCAIPN
LRENYGELADCCTKQEPERNECFLQHKDDNPSLPPFERPEAEAMCTSFKENPTTFMGHYLHEVARRHPYFYAPELLYYAEQYNEILTQCCAEADKESCLTP
KLDGVKEKALVSSVRQRMKCSSMQKFGERAFKAWAVARLSQTFPNADFAEITKLATDLTKVNKECCHGDLLECADDRAELAKYMCENQATISSKLQTCCDK
PLLKKAHCLSEVEHDTMPADLPAIAADFVEDQEVCKNYAEAKDVFLGTFLYEYSRRHPDYSVSLLLRLAKKYEATLEKCCAEANPPACYGTVLAEFQPLVE
EPKNLVKTNCDLYEKLGEYGFQNAILVRYTQKAPQVSTPTLVEAARNLGRVGTKCCTLPEDQRLPCVEDYLSAILNRVCLLHEKTPVSEHVTKCCSGSLVE
RRPCFSALTVDETYVPKEFKAETFTFHSDICTLPEKEKQIKKQTALAELVKHKPKATAEQLKTVMDDFAQFLDTCCKAADKDTCFSTEGPNLVTRCKDALA

*GGGGSGGGGSGGGGS*AS**DIQMTQSPSPLSASVGDRVTITCRASQYGGYVAWYQQKPGK*C*PKLLIYGASLLYSGVPSRFSGGRSGTDFTLTISSLQPEDFAT**
YYCQRGHALITFGQGTKVEIEGTTAASGSSGGSSSGAEVQLVESGGGLVQPGGSLRLSCAASGFNISSYGSMHWVRQAPGKGLEWVASIYPYSSSTYYADS
VKGRFTISADTSKNTAYLQMNSLRAEDTAVYYCARGYGPWYAYSYFALDYWG*C*GTLVTVSS*GGGGS***HHHHHH**--

LS-mouse SA-(Gly$_4$Ser)$_3$-scFv (V_H-V_L) sm3E-ds ($V_H44^{R>C}$ / $V_L100^{G>C}$)-(Gly$_4$Ser)-His$_6$

MDMRVPAQLLGLLLLWLPGARCEAHKSEIAHRYNDLGEQHFKGLVLIAFSQYLQKCSYDEHAKLVQEVTDFAKTCVADESAANCDKSLHTLFGDKLCAIPN
LRENYGELADCCTKQEPERNECFLQHKDDNPSLPPFERPEAEAMCTSFKENPTTFMGHYLHEVARRHPYFYAPELLYYAEQYNEILTQCCAEADKESCLTP
KLDGVKEKALVSSVRQRMKCSSMQKFGERAFKAWAVARLSQTFPNADFAEITKLATDLTKVNKECCHGDLLECADDRAELAKYMCENQATISSKLQTCCDK
PLLKKAHCLSEVEHDTMPADLPAIAADFVEDQEVCKNYAEAKDVFLGTFLYEYSRRHPDYSVSLLLRLAKKYEATLEKCCAEANPPACYGTVLAEFQPLVE
EPKNLVKTNCDLYEKLGEYGFQNAILVRYTQKAPQVSTPTLVEAARNLGRVGTKCCTLPEDQRLPCVEDYLSAILNRVCLLHEKTPVSEHVTKCCSGSLVE
RRPCFSALTVDETYVPKEFKAETFTFHSDICTLPEKEKQIKKQTALAELVKHKPKATAEQLKTVMDDFAQFLDTCCKAADKDTCFSTEGPNLVTRCKDALA
*GGGGSGGGGSGGGGS*AS**QVKLEQSGAEVVKPGASVKLSCKASGFNIKDSYMHWLRQGPGQ*C*LEWIGWIDPENGDTEYAPKFQGKATFTTDTSANTAYLGLS**
SLRPEDTAVYYCNEGTPTGPYYFDYWGQGTLVTVSSGGGGSGGGGSGGGGSENVLTQSPSSMSVSVGDRVTIACSASSSVPYMHWLQQKPGKSPKLLIYLT
SNLASGVPSRFSGSGSGTDYSLTISSVQPEDAATYYCQQRSSYPLTFG*C*GTKLEIK*GGGGS***HHHHHH**--
