# Supplementary material for: Directed evolution of broadly crossreactive chemokine-blocking antibodies efficacious in arthritis
Source: Nat Commun. 2018 Apr 13;9:1461. doi: 10.1038/s41467-018-03687-x (PMC5899157; doi:10.1038/s41467-018-03687-x)
Supplement: Supplementary file 7 — Supplementary Data 4 [file 41467_2018_3687_MOESM7_ESM.pdf]

Supplementary Data 4 - Oligonucleotide primers used for combinatorial site-directed mutagenesis

Product	Template	Primer forward sequence (5’ to 3’)	Primer reverse sequence (5’ to 3’)
**CK131**	CK63	CAGTTACTCTCCGTATTATACTTGGTTCTCTGCTGGTATG	CATACCAGCAGAGAACCAAGTATAATACGGAGAGTAACTG
**CK132**	CK63	CAGTTACTCTCCGTATTATACTTGGTTCTCTGCTGGTATG<br>GATTTACGGTGCATCCTACCCGTACTCCGGAGTCCCTTCCCG	CATACCAGCAGAGAACCAAGTATAATACGGAGAGTAACTG<br>CGGGAAGGGACTCCGGAGTACGGGTAGGATGCACCGTAAATC
**CK133**	CK63	GATTTACGGTGCATCCTACCCGTACTCCGGAGTCCCTTCCCG	CGGGAAGGGACTCCGGAGTACGGGTAGGATGCACCGTAAATC
**CK134**	CK66	CAGTTACTCTCCGTATTATTCTTGGTTCTCTGCTGGTATG	CATACCAGCAGAGAACCAAGAATAATACGGAGAGTAACTG
**CK135**	CK66	GATTTACGGTGCATCCTACCTGTACTCTGGAGTCCCTTCCCG<br>CAGTTACTCTCCGTATTATTCTTGGTTCTCTGCTGGTATG	CGGGAAGGGACTCCAGAGTACAGGTAGGATGCACCGTAAATC<br>CATACCAGCAGAGAACCAAGAATAATACGGAGAGTAACTG
**CK136**	CK66	GATTTACGGTGCATCCTACCTGTACTCTGGAGTCCCTTCCCG	CGGGAAGGGACTCCAGAGTACAGGTAGGATGCACCGTAAATC
**CK137**	CK72	GATTTACGGTGCATCCTACCCGTACTCTGGAGTCCCTTCCCG	CGGGAAGGGACTCCAGAGTACGGGTAGGATGCACCGTAAATC
**CK138**	CK72	GGTTACAGTTACTCTCCGTATTATTCTTGGTTCTCTGCTGG<br>CTTGGTTCTCTGCTGGTATGAACTACTGGGGTCAAGGAGCC	CCAGCAGAGAACCAAGAATAATACGGAGAGTAACTGTAACC<br>GGCTCCTTGACCCCAGTAGTTCATACCAGCAGAGAACCAAG
**CK139**	CK72	CTTGGTTCTCTGCTGGTATGAACTACTGGGGTCAAGGAGCC<br>CAGTTACTCTCCGTATTATACTTGGTTCTCTGCTGGTATG<br>GGTTACAGTTACTCTCCGTATTATTCTTGGTTCTCTGCTGG	GGCTCCTTGACCCCAGTAGTTCATACCAGCAGAGAACCAAG<br>CATACCAGCAGAGAACCAAGTATAATACGGAGAGTAACTG<br>CCAGCAGAGAACCAAGAATAATACGGAGAGTAACTGTAACC
**CK140**	CK72	GATTTACGGTGCATCCTACCCGTACTCTGGAGTCCCTTCCCG<br>GGTTACAGTTACTCTCCGTATTATTCTTGGTTCTCTGCTGG<br>CTTGGTTCTCTGCTGGTATGAACTACTGGGGTCAAGGAGCC	CGGGAAGGGACTCCAGAGTACGGGTAGGATGCACCGTAAATC<br>CCAGCAGAGAACCAAGAATAATACGGAGAGTAACTGTAACC<br>GGCTCCTTGACCCCAGTAGTTCATACCAGCAGAGAACCAAG
**CK141**	CK72	GATTTACGGTGCATCCTACCCGTACTCTGGAGTCCCTTCCCG	CGGGAAGGGACTCCAGAGTACGGGTAGGATGCACCGTAAATC

		CTTGGTTCTCTGCTGGTATGAACTACTGGGGTCAAGGAGCC<br>CAGTTACTCTCCGTATTATACTTGGTTCTCTGCTGGTATG<br>GGTTACAGTTACTCTCCGTATTATTCTTGGTTCTCTGCTGG	GGCTCCTTGACCCCAGTAGTTCATACCAGCAGAGAACCAAG<br>CATACCAGCAGAGAACCAAGTATAATACGGAGAGTAACTG<br>CCAGCAGAGAACCAAGAATAATACGGAGAGTAACTGTAACC
**CK142**	CK72	GGTTACAGTTACTCTCCGTATTATTCTTGGTTCTCTGCTGG	CCAGCAGAGAACCAAGAATAATACGGAGAGTAACTGTAACC
**CK143**	CK72	CAGTTACTCTCCGTATTATACTTGGTTCTCTGCTGGTATG<br>GGTTACAGTTACTCTCCGTATTATTCTTGGTTCTCTGCTGG	CATACCAGCAGAGAACCAAGTATAATACGGAGAGTAACTG<br>CCAGCAGAGAACCAAGAATAATACGGAGAGTAACTGTAACC
**CK144**	CK72	GATTTACGGTGCATCCTACCCGTACTCTGGAGTCCCTTCCCG<br>GGTTACAGTTACTCTCCGTATTATTCTTGGTTCTCTGCTGG	CGGGAAGGGACTCCAGAGTACGGGTAGGATGCACCGTAAATC<br>CCAGCAGAGAACCAAGAATAATACGGAGAGTAACTGTAACC
**CK145**	CK72	GATTTACGGTGCATCCTACCCGTACTCTGGAGTCCCTTCCCG<br>CAGTTACTCTCCGTATTATACTTGGTTCTCTGCTGGTATG<br>GGTTACAGTTACTCTCCGTATTATTCTTGGTTCTCTGCTGG	CGGGAAGGGACTCCAGAGTACGGGTAGGATGCACCGTAAATC<br>CATACCAGCAGAGAACCAAGTATAATACGGAGAGTAACTG<br>CCAGCAGAGAACCAAGAATAATACGGAGAGTAACTGTAACC
**CK146**	CK111	GGTGCGTCAGGCCCCGGGTGAGGAGCTGGAATGGGTTGC	GCAACCCATTCCAGCTCCTCACCCGGGGCCTGACGCACC
**CK147**	CK111	GCATCTATTGGTTCTTACCCTGGCTACACTGACTATGC	GCATAGTCAGTGTAGCCAGGGTAAGAACCAATAGATGC
**CK148**	CK111	GGTGCGTCAGGCCCCGGGTGAGGAGCTGGAATGGGTTGC<br>GCATCTATTGGTTCTTACCCTGGCTACACTGACTATGC	GCAACCCATTCCAGCTCCTCACCCGGGGCCTGACGCACC<br>GCATAGTCAGTGTAGCCAGGGTAAGAACCAATAGATGC
**CK149**	CK111	GCAGCTTCTGGCTCCAACCCCTACTACTACGGTGGTACG	CGTACCACCGTAGTAGTAGGGGTTGGAGCCAGAAGCTGC
**CK150**	CK108	CACGTTCGGACAGGGTACCGAGGTGGAGATCAAAGGTAC	GTACCTTTGATCTCCACCTCGGTACCCTGTCCGAACGTG
**CK151**	CK111	GGTGCGTCAGGCCCCGGGTGAGGAGCTGGAATGGGTTGC<br>GCAGCTTCTGGCTCCAACCCCTACTACTACGGTGGTACG	GCAACCCATTCCAGCTCCTCACCCGGGGCCTGACGCACC<br>CGTACCACCGTAGTAGTAGGGGTTGGAGCCAGAAGCTGC
**CK152**	CK108	CACGTTCGGACAGGGTACCGAGGTGGAGATCAAAGGTAC<br>GGTGCGTCAGGCCCCGGGTGAGGAGCTGGAATGGGTCGC	GTACCTTTGATCTCCACCTCGGTACCCTGTCCGAACGTG<br>GCGACCCATTCCAGCTCCTCACCCGGGGCCTGACGCACC
**CK153**	CK119	CACGTTCGGACAGGGTACCGAGGTGGAGATCAAAGGTAC	GTACCTTTGATCTCCACCTCGGTACCCTGTCCGAACGTG

**CK154**	CK119	GCATCTATTGGTTCTTACCCTGGCTACACTGACTATGC	GCATAGTCAGTGTAGCCAGGGTAAGAACCAATAGATGC
**CK155**	CK119	GCATCTATTGGTTCTTACCCTGGCTACACTGACTATGC<br>CACGTTCGGACAGGGTACCGAGGTGGAGATCAAAGGTAC	GCATAGTCAGTGTAGCCAGGGTAAGAACCAATAGATGC<br>GTACCTTTGATCTCCACCTCGGTACCCTGTCCGAACGTG
**CK156**	CK119	GCATCTATTGGTTCTTACCCTGGCTACACTGACTATGC<br>GCAGCTTCTGGCTCCAACCCCTACTACTACGGTGGTACG	GCATAGTCAGTGTAGCCAGGGTAAGAACCAATAGATGC<br>CGTACCACCGTAGTAGTAGGGGTTGGAGCCAGAAGCTGC
**CK157**	CK119	GCATCTATTGGTTCTTACCCTGGCTACACTGACTATGC<br>GCAGCTTCTGGCTCCAACCCCTACTACTACGGTGGTACG<br>CACGTTCGGACAGGGTACCGAGGTGGAGATCAAAGGTAC	GCATAGTCAGTGTAGCCAGGGTAAGAACCAATAGATGC<br>CGTACCACCGTAGTAGTAGGGGTTGGAGCCAGAAGCTGC<br>GTACCTTTGATCTCCACCTCGGTACCCTGTCCGAACGTG
**CK158**	CK108	GGTGCGTCAGGCCCCGGGTGAGGAGCTGGAATGGGTTGC	GCAACCCATTCCAGCTCCTCACCCGGGGCCTGACGCACC
