# Supplementary material for: Directed evolution of broadly crossreactive chemokine-blocking antibodies efficacious in arthritis
Source: Nat Commun. 2018 Apr 13;9:1461. doi: 10.1038/s41467-018-03687-x (PMC5899157; doi:10.1038/s41467-018-03687-x)
Supplement: Supplementary file 8 — Supplementary Data 5 [file 41467_2018_3687_MOESM8_ESM.pdf]

Supplementary Data 5 - Protein accession numbers, oligonucleotide primers, DNA and amino-acid sequences of ^{N}CXCL-Aga2^{C} fusion proteins

CXCL protein (residues / accession No.)	Construct for expression	Fusion protein
hCXCL1/GROα (38–107 / P09341)	pCHA-LS-hCXCL1$^{38\text{-}107}$-G_3-c-myc-Aga2	^{N}hCXCL1-Aga2^{C}
hCXCL2/GROβ (38–107 / P19875)	pCHA-LS-hCXCL2$^{38\text{-}107}$-G_3-c-myc-Aga2	^{N}hCXCL2-Aga2^{C}
hCXCL3/GROγ (38–107 / P19876)	pCHA-LS-hCXCL3$^{38\text{-}107}$-G_3-c-myc-Aga2	^{N}hCXCL3-Aga2^{C}
hCXCL4/PF4 (32–101 / P02776)	pCHA-LS-hCXCL4$^{32\text{-}101}$-G_3-c-myc-Aga2	^{N}hCXCL4-Aga2^{C}
hCXCL5/ENA-78 (44–114 / P42830)	pCHA-LS-hCXCL5$^{44\text{-}114}$-G_3-c-myc-Aga2	^{N}hCXCL5-Aga2^{C}
hCXCL6/GCP-2 (44–114 / P80162)	pCHA-LS-hCXCL6$^{44\text{-}114}$-G_3-c-myc-Aga2	^{N}hCXCL6-Aga2^{C}
hCXCL7/NAP-2 (59–121 / P02775)	pCHA-LS-hCXCL7$^{59\text{-}121}$-G_3-c-myc-Aga2	^{N}hCXCL7-Aga2^{C}
hCXCL8/IL-8 (29–99 / P10145)	pCHA-LS-hCXCL8$^{29\text{-}99}$-G_3-c-myc-Aga2	^{N}hCXCL8-Aga2^{C}
hCXCL9/MIG (23–125 / Q07325)	pCHA-LS-hCXCL9$^{23\text{-}125}$-G_3-c-myc-Aga2	^{N}hCXCL9-Aga2^{C}
hCXCL10/IP-10 (22–98 / P02778)	pCHA-LS-hCXCL10$^{22\text{-}98}$-G_3-c-myc-Aga2	^{N}hCXCL10-Aga2^{C}
hCXCL11/I-TAC (22–94 / O14625)	pCHA-LS-hCXCL11$^{22\text{-}94}$-G_3-c-myc-Aga2	^{N}hCXCL11-Aga2^{C}
mCXCL1/KC (28–96 / P12850)	pCHA-LS-mCXCL1$^{28\text{-}96}$-G_3-c-myc-Aga2	^{N}mCXCL1-Aga2^{C}
mCXCL2/MIP-2 (31–100 / P10889)	pCHA-LS-mCXCL2$^{31\text{-}100}$-G_3-c-myc-Aga2	^{N}mCXCL2-Aga2^{C}
mCXCL3/DCIP-1 (31–100 / Q6W5C0)	pCHA-LS-mCXCL3$^{31\text{-}100}$-G_3-c-myc-Aga2	^{N}mCXCL3-Aga2^{C}

mCXCL4/PF4 (30–105 / Q9Z126)	pCHA-LS-mCXCL4$^{30\text{-}105}$-G_3-c-myc-Aga2	^{N}mCXCL4-Aga2^{C}
mCXCL5LIX (48–118 / P50228)	pCHA-LS-mCXCL5$^{48\text{-}118}$-G_3-c-myc-Aga2	^{N}mCXCL5-Aga2^{C}
mCXCL7/NAP-2 (48–113 / Q9EQI5)	pCHA-LS-mCXCL7$^{48\text{-}113}$-G_3-c-myc-Aga2	^{N}mCXCL7-Aga2^{C}
mCXCL9/MIG (22–126 / P18340)	pCHA-LS-mCXCL9$^{22\text{-}126}$-G_3-c-myc-Aga2	^{N}mCXCL9-Aga2^{C}
mCXCL10/IP-10 (22–98 / P17515)	pCHA-LS-mCXCL10$^{22\text{-}98}$-G_3-c-myc-Aga2	^{N}mCXCL10-Aga2^{C}
mCXCL11/I-TAC (22–100 / Q9JHH5)	pCHA-LS-mCXCL11$^{22\text{-}100}$-G_3-c-myc-Aga2	^{N}mCXCL11-Aga2^{C}

**Primer name**	**Primer sequence (5' to 3')**
Forward-linker-Aga2-pCHA	5′-GGCGGAGGCGAACAAAAGC-3′
Reverse-LS-pCHA	5′-TCTCTTGTCCAAAGAGCCTTCTGC-3′
Forward-hCXCL1-Aga2	5′-TCTTTGGACAAGAGAGCCACCGAGCTGAGATGCC-3′
Reverse-hCXCL1-Aga2	5′-GATAAGCTTTTGTTCGCCTCCGCCGTTGCTC-3′
Forward-hCXCL2-Aga2	5′-TCTTTGGACAAGAGAGCCACAGAGCTGAGATGCC-3′
Reverse-hCXCL2-Aga2	5′-TTGTTCGCCTCCGCCGTTGCTCTTGCCGTTCTTCAGC-3′
Forward-hCXCL3-Aga2	5′-TCTTTGGACAAGAGAGTGACCGAGCTGAGATGCC-3′
Reverse-hCXCL3-Aga2	5′-TTGTTCGCCTCCGCCGTTGGTGCTGCCCTTGTTCAGG-3′
Forward-hCXCL4-Aga2	5′-TCTTTGGACAAGAGAGAGGCTGAAGAGGACGGCG-3′
Reverse-hCXCL4-Aga2	5′-TTGTTCGCCTCCGCCGCTTTCCAGCAGCTTCTTGATG-3′

Forward-hCXCL5-Aga2	5′-TCTTTGGACAAGAGACTGCGCGAGCTGAGATGC-3′
Reverse-hCXCL5-Aga2	5′-GATAAGCTTTTGTTCGCCTCCGCCGTTCTCTTTG-3′
Forward-hCXCL6-Aga2	5′-TCTTTGGACAAGAGACTGACCGAGCTGCGGTGCACC-3′
Reverse-hCXCL6-Aga2	5′-TTGTTCGCCTCCGCCGTTCTTCTTGTTGCCGCTGTCC-3′
Forward-hCXCL7-Aga2	5′-TCTTTGGACAAGAGAGCCGAGCTGCGGTGCATGTGC-3′
Reverse-hCXCL7-Aga2	5′-TTGTTCGCCTCCGCCCAGCTTTTTCTGCACG-3′
Forward-hCXCL8-Aga2	5′-TCTTTGGACAAGAGAGCCAAAGAACTGCGGTGCC-3′
Reverse-hCXCL8-Aga2	5′-GATAAGCTTTTGTTCGCCTCCGCCGCTGTTC-3′
Forward-hCXCL9-Aga2	5′-TCTTTGGACAAGAGAACCCCCGTCGTGCGGAAGG-3′
Reverse-hCXCL9-Aga2	5′-TTGTTCGCCTCCGCCTGTGGTTTTCTTCTGCCGGC-3′
Forward-hCXCL10-Aga2	5′-TCTTTGGACAAGAGAGTGCCTCTGAGCAGAACCG-3′
Reverse-hCXCL10-Aga2	5′-TTGTTCGCCTCCGCCTGGGCTCCGCTTGCTCCGC-3′
Forward-hCXCL11-Aga2	5′-TCTTTGGACAAGAGATTCCCCATGTTCAAGCGG-3′
Reverse-hCXCL11-Aga2	5′-TTGTTCGCCTCCGCCGAAGTTCTTCCGTTCCACC-3′
Forward-mCXCL1-Aga2	5′-TCTTTGGACAAGAGAGCCAACGAGCTGCGGTG-3′
Reverse-mCXCL1-Aga2	5′-GATAAGCTTTTGTTCGCCTCCGCCCTTGGGC-3′
Forward-mCXCL2-Aga2	5′-TCTTTGGACAAGAGAGCCAGCGAGCTGCGG-3′
Reverse-mCXCL2-Aga2	5′-GATAAGCTTTTGTTCGCCTCCGCCGTTGGC-3′

Forward-mCXCL3-Aga2	5′-TCTTTGGACAAGAGAGCCTCTGAGCTGAGATGCC-3′
Reverse-mCXCL3-Aga2	5′-TTGTTCGCCTCCGCCGCTGCTCTTGCCGGACTTCAGG-3′
Forward-mCXCL4-Aga2	5′-TCTTTGGACAAGAGAGTGACATCTGCCGGCCCTG-3′
Reverse-mCXCL4-Aga2	5′-TTGTTCGCCTCCGCCGCTTTCCAGGATCTTCTTAATC-3′
Forward-mCXCL5-Aga2	5′-TCTTTGGACAAGAGAGCCACCGAGCTGAGATGC-3′
Reverse-mCXCL5-Aga2	5′-GATAAGCTTTTGTTCGCCTCCGCCGGCTTTC-3′
Forward-mCXCL7-Aga2	5′-TCTTTGGACAAGAGAATCGAGCTGCGGTGCCGGTGC-3′
Reverse-mCXCL7-Aga2	5′-TTGTTCGCCTCCGCCGTAGCCTTCCAGAATCTTCATC-3′
Forward-mCXCL9-Aga2	5′-TCTTTGGACAAGAGAACCCTCGTGATCCGGAACG-3′
Reverse-mCXCL9-Aga2	5′-TTGTTCGCCTCCGCCTGTGGTCTTTCTGGATCTCC-3′
Forward-mCXCL10-Aga2	5′-TCTTTGGACAAGAGAATCCCACTGGCCAGAACCG-3′
Reverse-mCXCL10-Aga2	5′-TTGTTCGCCTCCGCCTGGGGCCCTCTTGCTCCG-3′
Forward-mCXCL11-Aga2	5′-TCTTTGGACAAGAGATTCCTGATGTTCAAGCAGG-3′
Reverse-mCXCL11-Aga2	5′-TTGTTCGCCTCCGCCCATGTTCTGCCGCCGCAGG-3′

Sequences of genes coding for ELR$^+$ and ELR$^-$ CXC chemokines fused to the N-terminus of a-agglutinin Aga2 protein (Aga2p): ^{N}CXCL-Aga2^{C}

DNA sequences of genes are provided. Start and stop codons are shown in black bold. The sequences include the secretory leader peptide (LS, in grey underlined), an active form of CXCL (in blue bold), a tripeptide $(Gly)_3$ flexible linker (G_3, in black italic and underlined), the c-myc tag (c-myc, in red bold) and the a-agglutinin Aga2 protein (Aga2p, in grey).

pCHA-LS-hCXCL1$^{38\text{-}107}$-G_3-c-myc-Aga2

ATGAAGGTTTTGATTGTCTTGTTGGCTATCTTCGCTGCTTTGCCATTGGCCTTAGCTCAACCGGTTATTTCTACTACCGTCGGTTCCGCTGCAGAAGGCTC
TTTGGACAAGAGA**GCCACCGAGCTGAGATGCCAGTGCCTGCAGACCCTGCAGGGCATCCACCCCAAGAACATCCAGAGCGTGAACGTGAAGTCCCCTGGCC**
CCCACTGCGCCCAGACCGAAGTGATCGCCACCCTGAAGAACGGCCGGAAGGCCTGCCTGAACCCCGCCAGCCCCATCGTGAAGAAAATCATCGAGAAGATG
CTGAACAGCGACAAGAGCAAC*GGCGGAGGC***GAACAAAAGCTTATCTCCGAAGAAGACTTG**CAGGAACTGACAACTATATGCGAGCAAATCCCCTCACCAAC
TTTAGAATCGACGCCGTACTCTTTGTCAACGACTACTATTTTGGCCAACGGGAAGGCAATGCAAGGAGTTTTTGAATATTACAAATCAGTAACGTTTGTCA
GTAATTGCGGTTCTCACCCCTCAACAACTAGCAAAGGCAGCCCCATAAACACACAGTATGTTTTT**TAA**

pCHA-LS-hCXCL2$^{38\text{-}107}$-G_3-c-myc-Aga2

ATGAAGGTTTTGATTGTCTTGTTGGCTATCTTCGCTGCTTTGCCATTGGCCTTAGCTCAACCGGTTATTTCTACTACCGTCGGTTCCGCTGCAGAAGGCTC
TTTGGACAAGAGA**GCCACAGAGCTGAGATGCCAGTGCCTCCAGACACTCCAGGGCATCCACCTGAAGAACATCCAGAGCGTGAAAGTGAAGTCCCCTGGCC**
CCCACTGCGCCCAGACAGAAGTGATCGCCACCCTGAAGAATGGCCAGAAGGCCTGCCTGAACCCCGCCAGCCCTATGGTCAAGAAAATCATCGAGAAGATG
CTGAAGAACGGCAAGAGCAAC*GGCGGAGGC***GAACAAAAGCTTATCTCCGAAGAAGACTTG**CAGGAACTGACAACTATATGCGAGCAAATCCCCTCACCAAC
TTTAGAATCGACGCCGTACTCTTTGTCAACGACTACTATTTTGGCCAACGGGAAGGCAATGCAAGGAGTTTTTGAATATTACAAATCAGTAACGTTTGTCA
GTAATTGCGGTTCTCACCCCTCAACAACTAGCAAAGGCAGCCCCATAAACACACAGTATGTTTTT**TAA**

pCHA-LS-hCXCL3$^{38\text{-}107}$-G_3-c-myc-Aga2

ATGAAGGTTTTGATTGTCTTGTTGGCTATCTTCGCTGCTTTGCCATTGGCCTTAGCTCAACCGGTTATTTCTACTACCGTCGGTTCCGCTGCAGAAGGCTC
TTTGGACAAGAGA**GTGACCGAGCTGAGATGCCAGTGCCTCCAGACACTCCAGGGCATCCACCTGAAGAACATCCAGAGCGTGAACGTGCGGAGCCCTGGCC**
CTCATTGTGCCCAGACAGAAGTGATCGCCACCCTGAAGAATGGCAAGAAGGCCTGCCTGAACCCCGCCAGCCCTATGGTGCAGAAGATCATCGAGAAGATC
CTGAACAAGGGCAGCACCAAC*GGCGGAGGC***GAACAAAAGCTTATCTCCGAAGAAGACTTG**CAGGAACTGACAACTATATGCGAGCAAATCCCCTCACCAAC

TTTAGAATCGACGCCGTACTCTTTGTCAACGACTACTATTTTGGCCAACGGGAAGGCAATGCAAGGAGTTTTTGAATATTACAAATCAGTAACGTTTGTCA
GTAATTGCGGTTCTCACCCCTCAACAACTAGCAAAGGCAGCCCCATAAACACACAGTATGTTTTT**TAA**

pCHA-LS-hCXCL4$^{32-101}$*-G*$_3$*-c-myc-Aga2*

ATGAAGGTTTTGATTGTCTTGTTGGCTATCTTCGCTGCTTTGCCATTGGCCTTAGCTCAACCGGTTATTTCTACTACCGTCGGTTCCGCTGCAGAAGGCTC
TTTGGACAAGAGA**GAGGCTGAAGAGGACGGCGATCTCCAGTGCCTGTGCGTGAAAACCACCAGCCAAGTGCGGCCCAGACACATCACCAGCCTGGAAGTGA**
TCAAGGCCGGACCCCACTGTCCTACCGCCCAGCTGATTGCCACCCTGAAGAACGGCCGGAAGATCTGCCTGGACCTCCAGGCCCCCCTGTACAAGAAGATC
ATCAAGAAGCTGCTGGAAAGC*GGCGGAGGC***GAACAAAAGCTTATCTCCGAAGAAGACTTG**CAGGAACTGACAACTATATGCGAGCAAATCCCCTCACCAAC
TTTAGAATCGACGCCGTACTCTTTGTCAACGACTACTATTTTGGCCAACGGGAAGGCAATGCAAGGAGTTTTTGAATATTACAAATCAGTAACGTTTGTCA
GTAATTGCGGTTCTCACCCCTCAACAACTAGCAAAGGCAGCCCCATAAACACACAGTATGTTTTT**TAA**

pCHA-LS-hCXCL5$^{44-114}$*-G*$_3$*-c-myc-Aga2*

ATGAAGGTTTTGATTGTCTTGTTGGCTATCTTCGCTGCTTTGCCATTGGCCTTAGCTCAACCGGTTATTTCTACTACCGTCGGTTCCGCTGCAGAAGGCTC
TTTGGACAAGAGA**CTGCGCGAGCTGAGATGCGTGTGCCTGCAGACCACCCAGGGCGTGCACCCCAAGATGATCAGCAACCTCCAGGTGTTCGCCATCGGCC**
CCCAGTGCAGCAAGGTGGAAGTGGTGGCCAGCCTGAAGAACGGCAAAGAGATCTGCCTGGACCCCGAGGCCCCATTCCTGAAGAAAGTGATCCAGAAGATC
CTGGACGGCGGCAACAAAGAGAAC*GGCGGAGGC***GAACAAAAGCTTATCTCCGAAGAAGACTTG**CAGGAACTGACAACTATATGCGAGCAAATCCCCTCACC
AACTTTAGAATCGACGCCGTACTCTTTGTCAACGACTACTATTTTGGCCAACGGGAAGGCAATGCAAGGAGTTTTTGAATATTACAAATCAGTAACGTTTG
TCAGTAATTGCGGTTCTCACCCCTCAACAACTAGCAAAGGCAGCCCCATAAACACACAGTATGTTTTT**TAA**

pCHA-LS-hCXCL6$^{44-114}$*-G*$_3$*-c-myc-Aga2*

ATGAAGGTTTTGATTGTCTTGTTGGCTATCTTCGCTGCTTTGCCATTGGCCTTAGCTCAACCGGTTATTTCTACTACCGTCGGTTCCGCTGCAGAAGGCTC
TTTGGACAAGAGA**CTGACCGAGCTGCGGTGCACCTGTCTGAGAGTGACCCTGCGCGTGAACCCCAAGACCATCGGCAAGCTCCAGGTGTTCCCTGCCGGCC**
CTCAGTGCAGCAAGGTGGAAGTGGTGGCCAGCCTGAAAAACGGAAAACAAGTGTGCCTGGACCCCGAGGCCCCATTCCTGAAGAAAGTGATCCAGAAGATC
CTGGACAGCGGCAACAAGAAGAAC*GGCGGAGGC***GAACAAAAGCTTATCTCCGAAGAAGACTTG**CAGGAACTGACAACTATATGCGAGCAAATCCCCTCACC
AACTTTAGAATCGACGCCGTACTCTTTGTCAACGACTACTATTTTGGCCAACGGGAAGGCAATGCAAGGAGTTTTTGAATATTACAAATCAGTAACGTTTG
TCAGTAATTGCGGTTCTCACCCCTCAACAACTAGCAAAGGCAGCCCCATAAACACACAGTATGTTTTT**TAA**

pCHA-LS-hCXCL7$^{59\text{-}121}$*-G*$_3$*-c-myc-Aga2*

ATGAAGGTTTTGATTGTCTTGTTGGCTATCTTCGCTGCTTTGCCATTGGCCTTAGCTCAACCGGTTATTTCTACTACCGTCGGTTCCGCTGCAGAAGGCTC
TTTGGACAAGAGA**GCCGAGCTGCGGTGCATGTGCATCAAGACCACCAGCGGAATCCACCCCAAGAATATCCAGTCCCTGGAAGTGATTGGCAAGGGCACCC**
ACTGCAACCAGGTGGAAGTGATTGCCACACTGAAAGACGGCCGGAAGATCTGCCTGGACCCTGACGCCCCCAGAATCAAGAAAATCGTGCAGAAAAAGCTG
*GGCGGAGGC***GAACAAAAGCTTATCTCCGAAGAAGACTTG**CAGGAACTGACAACTATATGCGAGCAAATCCCCTCACCAACTTTAGAATCGACGCCGTACTC
TTTGTCAACGACTACTATTTTGGCCAACGGGAAGGCAATGCAAGGAGTTTTTGAATATTACAAATCAGTAACGTTTGTCAGTAATTGCGGTTCTCACCCCT
CAACAACTAGCAAAGGCAGCCCCATAAACACACAGTATGTTTTT**TAA**

pCHA-LS-hCXCL8$^{29\text{-}99}$*-G*$_3$*-c-myc-Aga2*

ATGAAGGTTTTGATTGTCTTGTTGGCTATCTTCGCTGCTTTGCCATTGGCCTTAGCTCAACCGGTTATTTCTACTACCGTCGGTTCCGCTGCAGAAGGCTC
TTTGGACAAGAGA**GCCAAAGAACTGCGGTGCCAGTGCATCAAGACCTACAGCAAGCCCTTCCACCCCAAGTTCATCAAAGAACTGAGAGTGATCGAGAGCG**
GCCCTCACTGCGCCAACACCGAGATCATCGTGAAGCTGAGCGACGGCAGAGAGCTGTGCCTGGACCCCAAAGAAAACTGGGTGCAGCGGGTGGTGGAAAAG
TTCCTGAAGCGGGCCGAGAACAGC*GGCGGAGGC***GAACAAAAGCTTATCTCCGAAGAAGACTTG**CAGGAACTGACAACTATATGCGAGCAAATCCCCTCACC
AACTTTAGAATCGACGCCGTACTCTTTGTCAACGACTACTATTTTGGCCAACGGGAAGGCAATGCAAGGAGTTTTTGAATATTACAAATCAGTAACGTTTG
TCAGTAATTGCGGTTCTCACCCCTCAACAACTAGCAAAGGCAGCCCCATAAACACACAGTATGTTTTT**TAA**

pCHA-LS-hCXCL9$^{23\text{-}115}$*-G*$_3$*-c-myc-Aga2*

ATGAAGGTTTTGATTGTCTTGTTGGCTATCTTCGCTGCTTTGCCATTGGCCTTAGCTCAACCGGTTATTTCTACTACCGTCGGTTCCGCTGCAGAAGGCTC
TTTGGACAAGAGA**ACCCCCGTCGTGCGGAAGGGCAGATGCAGCTGTATCAGCACCAACCAGGGCACCATCCATCTCCAGTCTCTGAAGGACCTGAAGCAGT**
TCGCCCCCAGCCCCAGCTGCGAGAAGATCGAGATTATCGCCACACTGAAAAACGGGGTGCAGACCTGCCTGAACCCCGACAGCGCCGACGTGAAAGAACTG
ATCAAGAAATGGGAGAAACAGGTGTCCCAGAAGAAGAAGCAGAAGAACGGAAAGAAGCACCAGAAAAAGAAAGTGCTGAAAGTGCGGAAGTCCCAGCGGAG
CCGGCAGAAGAAAACCACA*GGCGGAGGC***GAACAAAAGCTTATCTCCGAAGAAGACTTG**CAGGAACTGACAACTATATGCGAGCAAATCCCCTCACCAACTT
TAGAATCGACGCCGTACTCTTTGTCAACGACTACTATTTTGGCCAACGGGAAGGCAATGCAAGGAGTTTTTGAATATTACAAATCAGTAACGTTTGTCAGT
AATTGCGGTTCTCACCCCTCAACAACTAGCAAAGGCAGCCCCATAAACACACAGTATGTTTTT**TAA**

pCHA-LS-hCXCL10$^{22\text{-}98}$*-G*$_3$*-c-myc-Aga2*

ATGAAGGTTTTGATTGTCTTGTTGGCTATCTTCGCTGCTTTGCCATTGGCCTTAGCTCAACCGGTTATTTCTACTACCGTCGGTTCCGCTGCAGAAGGCTC
TTTGGACAAGAGA**GTGCCTCTGAGCAGAACCGTGCGGTGCACCTGTATCAGCATCAGCAACCAGCCCGTGAACCCCAGAAGCCTGGAAAAGCTGGAAATCA**
TCCCCGCCAGCCAGTTCTGCCCCAGAGTGGAAATTATCGCCACCATGAAGAAGAAAGGCGAGAAGCGGTGCCTGAACCCCGAGAGCAAGGCCATCAAGAAC
CTGCTGAAGGCCGTGTCCAAAGAGCGGAGCAAGCGGAGCCCA*GGCGGAGGC***GAACAAAAGCTTATCTCCGAAGAAGACTTG**CAGGAACTGACAACTATATG
CGAGCAAATCCCCTCACCAACTTTAGAATCGACGCCGTACTCTTTGTCAACGACTACTATTTTGGCCAACGGGAAGGCAATGCAAGGAGTTTTTGAATATT
ACAAATCAGTAACGTTTGTCAGTAATTGCGGTTCTCACCCCTCAACAACTAGCAAAGGCAGCCCCATAAACACACAGTATGTTTTT**TAA**

pCHA-LS-hCXCL11$^{22-94}$*-G*$_{3}$*-c-myc-Aga2*

ATGAAGGTTTTGATTGTCTTGTTGGCTATCTTCGCTGCTTTGCCATTGGCCTTAGCTCAACCGGTTATTTCTACTACCGTCGGTTCCGCTGCAGAAGGCTC
TTTGGACAAGAGA**TTCCCCATGTTCAAGCGGGGCAGATGCCTGTGCATCGGCCCTGGCGTGAAAGCCGTGAAGGTGGCCGATATCGAGAAGGCCAGCATCA**
TGTACCCCAGCAACAACTGCGACAAGATCGAAGTGATCATCACCCTGAAAGAGAACAAGGGCCAGAGATGCCTGAATCCCAAGTCCAAGCAGGCCCGGCTG
ATCATCAAGAAGGTGGAACGGAAGAACTTC*GGCGGAGGC***GAACAAAAGCTTATCTCCGAAGAAGACTTG**CAGGAACTGACAACTATATGCGAGCAAATCCC
CTCACCAACTTTAGAATCGACGCCGTACTCTTTGTCAACGACTACTATTTTGGCCAACGGGAAGGCAATGCAAGGAGTTTTTGAATATTACAAATCAGTAA
CGTTTGTCAGTAATTGCGGTTCTCACCCCTCAACAACTAGCAAAGGCAGCCCCATAAACACACAGTATGTTTTT**TAA**

pCHA-LS-mCXCL1$^{28-96}$*-G*$_{3}$*-c-myc-Aga2*

ATGAAGGTTTTGATTGTCTTGTTGGCTATCTTCGCTGCTTTGCCATTGGCCTTAGCTCAACCGGTTATTTCTACTACCGTCGGTTCCGCTGCAGAAGGCTC
TTTGGACAAGAGA**GCCAACGAGCTGCGGTGCCAGTGCCTGCAGACCATGGCCGGCATCCACCTGAAGAACATCCAGAGCCTGAAGGTGCTGCCCAGCGGCC**
CTCACTGCACCCAGACCGAAGTGATCGCCACCCTGAAGAACGGCAGAGAGGCCTGCCTGGATCCCGAGGCCCCCCTGGTGCAGAAAATCGTGCAGAAAATG
CTGAAGGGCGTGCCCAAG*GGCGGAGGC***GAACAAAAGCTTATCTCCGAAGAAGACTTG**CAGGAACTGACAACTATATGCGAGCAAATCCCCTCACCAACTTT
AGAATCGACGCCGTACTCTTTGTCAACGACTACTATTTTGGCCAACGGGAAGGCAATGCAAGGAGTTTTTGAATATTACAAATCAGTAACGTTTGTCAGTA
ATTGCGGTTCTCACCCCTCAACAACTAGCAAAGGCAGCCCCATAAACACACAGTATGTTTTT**TAA**

pCHA-LS-mCXCL2$^{31-100}$*-G*$_{3}$*-c-myc-Aga2*

ATGAAGGTTTTGATTGTCTTGTTGGCTATCTTCGCTGCTTTGCCATTGGCCTTAGCTCAACCGGTTATTTCTACTACCGTCGGTTCCGCTGCAGAAGGCTC
TTTGGACAAGAGA**GCCAGCGAGCTGCGGTGCCAGTGCCTGAAAACCCTGCCCCGGGTGGACTTCAAGAACATCCAGAGCCTGAGCGTGACCCCCCCTGGCC**
CTCACTGTGCCCAGACCGAAGTGATCGCCACCCTGAAGGGCGGCCAGAAAGTGTGCCTGGACCCCGAGGCCCCCCTGGTGCAGAAGATCATCCAGAAGATC
CTGAACAAGGGCAAGGCCAAC*GGCGGAGGC***GAACAAAAGCTTATCTCCGAAGAAGACTTG**CAGGAACTGACAACTATATGCGAGCAAATCCCCTCACCAAC
TTTAGAATCGACGCCGTACTCTTTGTCAACGACTACTATTTTGGCCAACGGGAAGGCAATGCAAGGAGTTTTTGAATATTACAAATCAGTAACGTTTGTCA
GTAATTGCGGTTCTCACCCCTCAACAACTAGCAAAGGCAGCCCCATAAACACACAGTATGTTTTT**TAA**

pCHA-LS-mCXCL3$^{31-100}$*-G*$_{3}$*-c-myc-Aga2*

ATGAAGGTTTTGATTGTCTTGTTGGCTATCTTCGCTGCTTTGCCATTGGCCTTAGCTCAACCGGTTATTTCTACTACCGTCGGTTCCGCTGCAGAAGGCTC
TTTGGACAAGAGA**GCCTCTGAGCTGAGATGCCAGTGCCTGAACACCCTGCCCCGGGTGGACTTCGAGACAATCCAGAGCCTGACCGTGACCCCCCCTGGCC**
CTCACTGTACCCAGACAGAAGTGATCGCCACCCTGAAGGACGGCCAGGAAGTGTGCCTGAATCCCCAGGGCCCCAGACTCCAGATCATCATCAAGAAGATC
CTGAAGTCCGGCAAGAGCAGC*GGCGGAGGC***GAACAAAAGCTTATCTCCGAAGAAGACTTG**CAGGAACTGACAACTATATGCGAGCAAATCCCCTCACCAAC
TTTAGAATCGACGCCGTACTCTTTGTCAACGACTACTATTTTGGCCAACGGGAAGGCAATGCAAGGAGTTTTTGAATATTACAAATCAGTAACGTTTGTCA
GTAATTGCGGTTCTCACCCCTCAACAACTAGCAAAGGCAGCCCCATAAACACACAGTATGTTTTT**TAA**

pCHA-LS-mCXCL4$^{30-105}$*-G*$_3$*-c-myc-Aga2*

ATGAAGGTTTTGATTGTCTTGTTGGCTATCTTCGCTGCTTTGCCATTGGCCTTAGCTCAACCGGTTATTTCTACTACCGTCGGTTCCGCTGCAGAAGGCTC
TTTGGACAAGAGA**GTGACATCTGCCGGCCCTGAGGAAAGCGACGGCGATCTGTCTTGCGTGTGCGTGAAAACCATCAGCAGCGGCATCCACCTGAAGCACA**
TCACCAGCCTGGAAGTGATCAAGGCCGGCAGGCACTGTGCCGTGCCTCAGCTGATTGCCACCCTGAAGAACGGCCGGAAGATCTGCCTGGACAGACAGGCC
CCCCTGTACAAGAAAGTGATTAAGAAGATCCTGGAAAGC*GGCGGAGGC***GAACAAAAGCTTATCTCCGAAGAAGACTTG**CAGGAACTGACAACTATATGCGA
GCAAATCCCCTCACCAACTTTAGAATCGACGCCGTACTCTTTGTCAACGACTACTATTTTGGCCAACGGGAAGGCAATGCAAGGAGTTTTTGAATATTACA
AATCAGTAACGTTTGTCAGTAATTGCGGTTCTCACCCCTCAACAACTAGCAAAGGCAGCCCCATAAACACACAGTATGTTTTT**TAA**

pCHA-LS-mCXCL5$^{48-118}$*-G*$_3$*-c-myc-Aga2*

ATGAAGGTTTTGATTGTCTTGTTGGCTATCTTCGCTGCTTTGCCATTGGCCTTAGCTCAACCGGTTATTTCTACTACCGTCGGTTCCGCTGCAGAAGGCTC
TTTGGACAAGAGA**GCCACCGAGCTGAGATGCGTGTGCCTGACCGTGACCCCCAAGATCAACCCCAAGCTGATCGCCAACCTGGAAGTGATCCCTGCCGGCC**
CTCAGTGCCCCACCGTGGAAGTGATTGCCAAGCTGAAGAACCAGAAAGAAGTGTGCCTGGACCCCGAGGCCCCCGTGATCAAGAAGATCATCCAGAAGATC
CTGGGCAGCGACAAGAAGAAAGCC*GGCGGAGGC***GAACAAAAGCTTATCTCCGAAGAAGACTTG**CAGGAACTGACAACTATATGCGAGCAAATCCCCTCACC
AACTTTAGAATCGACGCCGTACTCTTTGTCAACGACTACTATTTTGGCCAACGGGAAGGCAATGCAAGGAGTTTTTGAATATTACAAATCAGTAACGTTTG
TCAGTAATTGCGGTTCTCACCCCTCAACAACTAGCAAAGGCAGCCCCATAAACACACAGTATGTTTTT**TAA**

pCHA-LS-mCXCL7$^{48-113}$*-G*$_3$*-c-myc-Aga2*

ATGAAGGTTTTGATTGTCTTGTTGGCTATCTTCGCTGCTTTGCCATTGGCCTTAGCTCAACCGGTTATTTCTACTACCGTCGGTTCCGCTGCAGAAGGCTC
TTTGGACAAGAGA**ATCGAGCTGCGGTGCCGGTGCACCAACACCATCAGCGGCATCCCTTTCAACAGCATCAGCCTCGTGAACGTGTACAGACCCGGCGTGC**
ACTGCGCCGACGTGGAAGTGATTGCTACACTGAAGAATGGGCAGAAAACCTGCCTGGACCCCAACGCCCCTGGCGTGAAGCGGATCGTGATGAAGATTCTG
GAAGGCTAC*GGCGGAGGC***GAACAAAAGCTTATCTCCGAAGAAGACTTG**CAGGAACTGACAACTATATGCGAGCAAATCCCCTCACCAACTTTAGAATCGAC
GCCGTACTCTTTGTCAACGACTACTATTTTGGCCAACGGGAAGGCAATGCAAGGAGTTTTTGAATATTACAAATCAGTAACGTTTGTCAGTAATTGCGGTT
CTCACCCCTCAACAACTAGCAAAGGCAGCCCCATAAACACACAGTATGTTTTT**TAA**

pCHA-LS-mCXCL9$^{22-126}$*-G*$_3$*-c-myc-Aga2*

ATGAAGGTTTTGATTGTCTTGTTGGCTATCTTCGCTGCTTTGCCATTGGCCTTAGCTCAACCGGTTATTTCTACTACCGTCGGTTCCGCTGCAGAAGGCTC
TTTGGACAAGAGA**ACCCTCGTGATCCGGAACGCCCGGTGCAGCTGTATCAGCACCAGCAGAGGCACCATCCACTACAAGAGCCTGAAGGATCTGAAGCAGT**
TCGCCCCCAGCCCCAACTGCAACAAGACCGAGATTATCGCCACACTGAAAAACGGGGACCAGACCTGTCTGGACCCCGACAGCGCCAACGTGAAGAAACTG
ATGAAGGAATGGGAGAAGAAGATCAGCCAGAAGAAGAAGCAGAAGCGGGGCAAGAAACACCAGAAAAACATGAAGAACCGGAAGCCCAAGACCCCCCAGAG
CCGGCGGAGATCCAGAAAGACCACA*GGCGGAGGC***GAACAAAAGCTTATCTCCGAAGAAGACTTG**CAGGAACTGACAACTATATGCGAGCAAATCCCCTCAC
CAACTTTAGAATCGACGCCGTACTCTTTGTCAACGACTACTATTTTGGCCAACGGGAAGGCAATGCAAGGAGTTTTTGAATATTACAAATCAGTAACGTTT
GTCAGTAATTGCGGTTCTCACCCCTCAACAACTAGCAAAGGCAGCCCCATAAACACACAGTATGTTTTT**TAA**

pCHA-LS-mCXCL10$^{22-98}$-G_3-c-myc-Aga2

ATGAAGGTTTTGATTGTCTTGTTGGCTATCTTCGCTGCTTTGCCATTGGCCTTAGCTCAACCGGTTATTTCTACTACCGTCGGTTCCGCTGCAGAAGGCTC
TTTGGACAAGAGA**ATCCCACTGGCCAGAACCGTGCGGTGCAACTGCATCCACATCGACGATGGCCCCGTGCGGATGAGAGCCATCGGCAAGCTGGAAATCA**
TCCCCGCCAGCCTGAGCTGCCCCAGAGTGGAAATTATCGCCACCATGAAGAAGAACGACGAGCAGCGGTGCCTGAACCCCGAGAGCAAGACCATCAAGAAC
CTGATGAAGGCCTTTAGCCAGAAGCGGAGCAAGAGGGCCCCA*GGCGGAGGC***GAACAAAAGCTTATCTCCGAAGAAGACTTG**CAGGAACTGACAACTATATG
CGAGCAAATCCCCTCACCAACTTTAGAATCGACGCCGTACTCTTTGTCAACGACTACTATTTTGGCCAACGGGAAGGCAATGCAAGGAGTTTTTGAATATT
ACAAATCAGTAACGTTTGTCAGTAATTGCGGTTCTCACCCCTCAACAACTAGCAAAGGCAGCCCCATAAACACACAGTATGTTTTT**TAA**

pCHA-LS-mCXCL11$^{22-100}$-G_3-c-myc-Aga2

ATGAAGGTTTTGATTGTCTTGTTGGCTATCTTCGCTGCTTTGCCATTGGCCTTAGCTCAACCGGTTATTTCTACTACCGTCGGTTCCGCTGCAGAAGGCTC
TTTGGACAAGAGA**TTCCTGATGTTCAAGCAGGGCCGGTGCCTGTGCATCGGCCCTGGAATGAAGGCCGTGAAGATGGCCGAGATCGAGAAGGCCAGCGTGA**
TCTACCCCAGCAACGGCTGCGACAAGGTGGAAGTGATCGTGACCATGAAGGCCCACAAGCGGCAGAGATGCCTGGACCCCAGATCCAAGCAGGCCCGGCTG
ATCATGCAGGCTATCGAGAAGAAGAATTTCCTGCGGCGGCAGAACATG*GGCGGAGGC***GAACAAAAGCTTATCTCCGAAGAAGACTTG**CAGGAACTGACAAC
TATATGCGAGCAAATCCCCTCACCAACTTTAGAATCGACGCCGTACTCTTTGTCAACGACTACTATTTTGGCCAACGGGAAGGCAATGCAAGGAGTTTTTG
AATATTACAAATCAGTAACGTTTGTCAGTAATTGCGGTTCTCACCCCTCAACAACTAGCAAAGGCAGCCCCATAAACACACAGTATGTTTTT**TAA**

Amino-acid sequences of ELR^{+} and ELR^{-} CXC chemokines fused to the N-terminus of a-agglutinin Aga2 protein (Aga2p): ^{N}CXCL-Aga2^{C}

Amino acid sequences of translated polypeptides are shown. The sequences include the secretory leader peptide (LS, in grey underlined), an active form of CXCL (in blue bold), a tripeptide $(Gly)_3$ flexible linker (G_3, in black italic and underlined), the c-myc tag (c-myc, in red bold) and the a-agglutinin Aga2 protein (Aga2p, in grey).

LS-hCXCL1$^{38\text{-}107}$-G_3-c-myc-Aga2

MKVLIVLLAIFAALPLALAQPVISTTVGSAAEGSLDKR**ATELRCQCLQTLQGIHPKNIQSVNVKSPGPHCAQTEVIATLKNGRKACLNPASPIVKKIIEKM**
LNSDKSN*GGG***EQKLISEEDLQ**ELTTICEQIPSPTLESTPYSLSTTTILANGKAMQGVFEYYKSVTFVSNCGSHPSTTSKGSPINTQYVF-

LS-hCXCL2$^{38\text{-}107}$-G_3-c-myc-Aga2

MKVLIVLLAIFAALPLALAQPVISTTVGSAAEGSLDKR**ATELRCQCLQTLQGIHLKNIQSVKVKSPGPHCAQTEVIATLKNGQKACLNPASPMVKKIIEKM**
LKNGKSN*GGG***EQKLISEEDLQ**ELTTICEQIPSPTLESTPYSLSTTTILANGKAMQGVFEYYKSVTFVSNCGSHPSTTSKGSPINTQYVF-

LS-hCXCL3$^{38\text{-}107}$-G_3-c-myc-Aga2

MKVLIVLLAIFAALPLALAQPVISTTVGSAAEGSLDKR**VTELRCQCLQTLQGIHLKNIQSVNVRSPGPHCAQTEVIATLKNGKKACLNPASPMVQKIIEKI**
LNKGSTN*GGG***EQKLISEEDLQ**ELTTICEQIPSPTLESTPYSLSTTTILANGKAMQGVFEYYKSVTFVSNCGSHPSTTSKGSPINTQYVF-

LS-hCXCL4$^{32\text{-}101}$-G_3-c-myc-Aga2

MKVLIVLLAIFAALPLALAQPVISTTVGSAAEGSLDKR**EAEEDGDLQCLCVKTTSQVRPRHITSLEVIKAGPHCPTAQLIATLKNGRKICLDLQAPLYKKI**
IKKLLES*GGG***EQKLISEEDLQ**ELTTICEQIPSPTLESTPYSLSTTTILANGKAMQGVFEYYKSVTFVSNCGSHPSTTSKGSPINTQYVF-

LS-hCXCL5$^{44\text{-}114}$-G_3-c-myc-Aga2

MKVLIVLLAIFAALPLALAQPVISTTVGSAAEGSLDKR**LRELRCVCLQTTQGVHPKMISNLQVFAIGPQCSKVEVVASLKNGKEICLDPEAPFLKKVIQKI**
LDGGNKEN*GGG***EQKLISEEDLQ**ELTTICEQIPSPTLESTPYSLSTTTILANGKAMQGVFEYYKSVTFVSNCGSHPSTTSKGSPINTQYVF-

LS-hCXCL6$^{44-114}$-G$_3$-c-myc-Aga2

MKVLIVLLAIFAALPLALAQPVISTTVGSAAEGSLDKR**LTELRCTCLRVTLRVNPKTIGKLQVFPAGPQCSKVEVVASLKNGKQVCLDPEAPFLKKVIQKI**
LDSGNKKN*GGG***EQKLISEEDLQ**ELTTICEQIPSPTLESTPYSLSTTTILANGKAMQGVFEYYKSVTFVSNCGSHPSTTSKGSPINTQYVF-

LS-hCXCL7$^{59-121}$-G$_3$-c-myc-Aga2

MKVLIVLLAIFAALPLALAQPVISTTVGSAAEGSLDKR**AELRCMCIKTTSGIHPKNIQSLEVIGKGTHCNQVEVIATLKDGRKICLDPDAPRIKKIVQKKL**
*GGG***EQKLISEEDLQ**ELTTICEQIPSPTLESTPYSLSTTTILANGKAMQGVFEYYKSVTFVSNCGSHPSTTSKGSPINTQYVF-

LS-hCXCL8$^{29-99}$-G$_3$-c-myc-Aga2

MKVLIVLLAIFAALPLALAQPVISTTVGSAAEGSLDKR**AKELRCQCIKTYSKPFHPKFIKELRVIESGPHCANTEIIVKLSDGRELCLDPKENWVQRVVEK**
FLKRAENS*GGG***EQKLISEEDLQ**ELTTICEQIPSPTLESTPYSLSTTTILANGKAMQGVFEYYKSVTFVSNCGSHPSTTSKGSPINTQYVF-

LS-hCXCL9$^{23-115}$-G$_3$-c-myc-Aga2

MKVLIVLLAIFAALPLALAQPVISTTVGSAAEGSLDKR**TPVVRKGRCSCISTNQGTIHLQSLKDLKQFAPSPSCEKIEIIATLKNGVQTCLNPDSADVKEL**
IKKWEKQVSQKKKQKNGKKHQKKKVLKVRKSQRSRQKKTT*GGG***EQKLISEEDLQ**ELTTICEQIPSPTLESTPYSLSTTTILANGKAMQGVFEYYKSVTFVS
NCGSHPSTTSKGSPINTQYVF-

LS-hCXCL10$^{22-98}$-G$_3$-c-myc-Aga2

MKVLIVLLAIFAALPLALAQPVISTTVGSAAEGSLDKR**VPLSRTVRCTCISISNQPVNPRSLEKLEIIPASQFCPRVEIIATMKKKGEKRCLNPESKAIKN**
LLKAVSKERSKRSP*GGG***EQKLISEEDLQ**ELTTICEQIPSPTLESTPYSLSTTTILANGKAMQGVFEYYKSVTFVSNCGSHPSTTSKGSPINTQYVF-

LS-hCXCL11$^{22-94}$-G$_3$-c-myc-Aga2

MKVLIVLLAIFAALPLALAQPVISTTVGSAAEGSLDKR**FPMFKRGRCLCIGPGVKAVKVADIEKASIMYPSNNCDKIEVIITLKENKGQRCLNPKSKQARL**
IIKKVERKNF*GGG***EQKLISEEDLQ**ELTTICEQIPSPTLESTPYSLSTTTILANGKAMQGVFEYYKSVTFVSNCGSHPSTTSKGSPINTQYVF-

LS-mCXCL1$^{28-96}$-G$_3$-c-myc-Aga2

MKVLIVLLAIFAALPLALAQPVISTTVGSAAEGSLDKR**ANELRCQCLQTMAGIHLKNIQSLKVLPSGPHCTQTEVIATLKNGREACLDPEAPLVQKIVQKM**
LKGVPK*GGG***EQKLISEEDLQ**ELTTICEQIPSPTLESTPYSLSTTTILANGKAMQGVFEYYKSVTFVSNCGSHPSTTSKGSPINTQYVF-

LS-mCXCL2$^{31\text{-}100}$*-G*$_3$*-c-myc-Aga2*

MKVLIVLLAIFAALPLALAQPVISTTVGSAAEGSLDKR**ASELRCQCLKTLPRVDFKNIQSLSVTPPGPHCAQTEVIATLKGGQKVCLDPEAPLVQKIIQKI**
LNKGKAN_GGG_**EQKLISEEDLQ**ELTTICEQIPSPTLESTPYSLSTTTILANGKAMQGVFEYYKSVTFVSNCGSHPSTTSKGSPINTQYVF-

LS-mCXCL3$^{31\text{-}100}$*-G*$_3$*-c-myc-Aga2*

MKVLIVLLAIFAALPLALAQPVISTTVGSAAEGSLDKR**ASELRCQCLNTLPRVDFETIQSLTVTPPGPHCTQTEVIATLKDGQEVCLNPQGPRLQIIIKKI**
LKSGKSS_GGG_**EQKLISEEDLQ**ELTTICEQIPSPTLESTPYSLSTTTILANGKAMQGVFEYYKSVTFVSNCGSHPSTTSKGSPINTQYVF-

LS-mCXCL4$^{30\text{-}105}$*-G*$_3$*-c-myc-Aga2*

MKVLIVLLAIFAALPLALAQPVISTTVGSAAEGSLDKR**VTSAGPEESDGDLSCVCVKTISSGIHLKHITSLEVIKAGRHCAVPQLIATLKNGRKICLDRQA**
PLYKKVIKKILES_GGG_**EQKLISEEDLQ**ELTTICEQIPSPTLESTPYSLSTTTILANGKAMQGVFEYYKSVTFVSNCGSHPSTTSKGSPINTQYVF-

LS-mCXCL5$^{48\text{-}118}$*-G*$_3$*-c-myc-Aga2*

MKVLIVLLAIFAALPLALAQPVISTTVGSAAEGSLDKR**ATELRCVCLTVTPKINPKLIANLEVIPAGPQCPTVEVIAKLKNQKEVCLDPEAPVIKKIIQKI**
LGSDKKKA_GGG_**EQKLISEEDLQ**ELTTICEQIPSPTLESTPYSLSTTTILANGKAMQGVFEYYKSVTFVSNCGSHPSTTSKGSPINTQYVF-

LS-mCXCL7$^{48\text{-}113}$*-G*$_3$*-c-myc-Aga2*

MKVLIVLLAIFAALPLALAQPVISTTVGSAAEGSLDKR**IELRCRCTNTISGIPFNSISLVNVYRPGVHCADVEVIATLKNGQKTCLDPNAPGVKRIVMKIL**
EGY_GGG_**EQKLISEEDLQ**ELTTICEQIPSPTLESTPYSLSTTTILANGKAMQGVFEYYKSVTFVSNCGSHPSTTSKGSPINTQYVF-

LS-mCXCL9$^{22\text{-}126}$*-G*$_3$*-c-myc-Aga2*

MKVLIVLLAIFAALPLALAQPVISTTVGSAAEGSLDKR**TLVIRNARCSCISTSRGTIHYKSLKDLKQFAPSPNCNKTEIIATLKNGDQTCLDPDSANVKKL**
MKEWEKKISQKKKQKRGKKHQKNMKNRKPKTPQSRRRSRKTT_GGG_**EQKLISEEDLQ**ELTTICEQIPSPTLESTPYSLSTTTILANGKAMQGVFEYYKSVTF
VSNCGSHPSTTSKGSPINTQYVF-

LS-mCXCL10$^{22\text{-}98}$*-G*$_3$*-c-myc-Aga2*

MKVLIVLLAIFAALPLALAQPVISTTVGSAAEGSLDKR**IPLARTVRCNCIHIDDGPVRMRAIGKLEIIPASLSCPRVEIIATMKKNDEQRCLNPESKTIKN**
LMKAFSQKRSKRAP_GGG_**EQKLISEEDLQ**ELTTICEQIPSPTLESTPYSLSTTTILANGKAMQGVFEYYKSVTFVSNCGSHPSTTSKGSPINTQYVF-

LS-mCXCL11$^{22\text{-}100}$*-*G_3*-c-myc-Aga2*

MKVLIVLLAIFAALPLALAQPVISTTVGSAAEGSLDKR**FLMFKQGRCLCIGPGMKAVKMAEIEKASVIYPSNGCDKVEVIVTMKAHKRQRCLDPRSKQARL**
IMQAIEKKNFLRRQNM*GGG***EQKLISEEDLQ**ELTTICEQIPSPTLESTPYSLSTTTILANGKAMQGVFEYYKSVTFVSNCGSHPSTTSKGSPINTQYVF-
