# Supplementary material for: Directed evolution of broadly crossreactive chemokine-blocking antibodies efficacious in arthritis
Source: Nat Commun. 2018 Apr 13;9:1461. doi: 10.1038/s41467-018-03687-x (PMC5899157; doi:10.1038/s41467-018-03687-x)
Supplement: Supplementary file 9 — Supplementary Data 6 [file 41467_2018_3687_MOESM9_ESM.pdf]

Supplementary Data 6 - Epitope mapping by Alanine scanning of hCXCL1

DNA sequence of hCXCL1/GROα fused to the N-terminus of Aga2. Start and stop codons are shown in black bold. The sequence includes the secretory leader peptide (LS, in grey underlined), an active form of hCXCL1/GROα (in black), a tripeptide $(Gly)_3$ flexible linker (G_3, in black italic and underlined), a c-myc tag (c-myc, in red bold) and the a-agglutinin Aga2 protein (Aga2p, in green).

pCHA-LS-hCXCL1-G₃-c-myc-Aga2

ATGAAGGTTTTGATTGTCTTGTTGGCTATCTTCGCTGCTTTGCCATTGGCCTTAGCTCAACCGGTTATTTCTACTACCGTCGGT
TCCGCTGCAGAAGGCTCTTTGGACAAGAGAGCCACCGAGCTGAGATGCCAGTGCCTGCAGACCCTGCAGGGCATCCACCCCAAG
AACATCCAGAGCGTGAACGTGAAGTCCCCTGGCCCCCACTGCGCCCAGACCGAAGTGATCGCCACCCTGAAGAACGGCCGGAAG
GCCTGCCTGAACCCCGCCAGCCCCATCGTGAAGAAAATCATCGAGAAGATGCTGAACAGCGACAAGAGCAAC*GGCGGAGGC***GAA**
CAAAAGCTTATCTCCGAAGAAGACTTGCAGGAACTGACAACTATATGCGAGCAAATCCCCTCACCAACTTTAGAATCGACGCCG
TACTCTTTGTCAACGACTACTATTTTGGCCAACGGGAAGGCAATGCAAGGAGTTTTTGAATATTACAAATCAGTAACGTTTGTC
AGTAATTGCGGTTCTCACCCCTCAACAACTAGCAAAGGCAGCCCCATAAACACACAGTATGTTTTT**TAA**

Amino-acid sequence of translated ^{N}hCXCL1/GROα -Aga2^{C} polypeptide is shown. The sequence includes the secretory leader peptide (LS, in grey underlined), an active form of hCXCL1/GROα (in black), a tripeptide $(Gly)_3$ flexible linker (G_3, in black italic and underlined), the c-myc tag (c-myc, in red bold) and the a-agglutinin Aga2 protein (Aga2p, in green).

LS-hCXCL1-G₃-c-myc-Aga2

MKVLIVLLAIFAALPLALAQPVISTTVGSAAEGSLDKRATELRCQCLQTLQGIHPKNIQSVNVKSPGPHCAQTEVIATLKNGRK
ACLNPASPIVKKIIEKMLNSDKSN*GGG***EQKLISEEDLQ**ELTTICEQIPSPTLESTPYSLSTTTILANGKAMQGVFEYYKSVTFV
SNCGSHPSTTSKGSPINTQYVF-

Amino-acid sequence of translated wild-type ^{N}hCXCL1/GROα -Aga2^{C} polypeptide and relative alanine mutants are shown. For simplicity only the amino-acid sequence ^{N}hCXCL1/GROα^{C} is indicated. Mutated residues are presented in orange bold.

Wild-type Groα	^{N}ATELRCQCLQTLQGIHPKNIQSVNVKSPGPHCAQTEVIATLKNGRKACLNPASPIVKKIIEKMLNSDKSN^{C}
GROα – A4S	^{N}**S**TELRCQCLQTLQGIHPKNIQSVNVKSPGPHCAQTEVIATLKNGRKACLNPASPIVKKIIEKMLNSDKSN^{C}
GROα – T5S	^{N}A**A**ELRCQCLQTLQGIHPKNIQSVNVKSPGPHCAQTEVIATLKNGRKACLNPASPIVKKIIEKMLNSDKSN^{C}
GROα – E6A	^{N}AT**A**LRCQCLQTLQGIHPKNIQSVNVKSPGPHCAQTEVIATLKNGRKACLNPASPIVKKIIEKMLNSDKSN^{C}
GROα – L7A	^{N}ATE**A**RCQCLQTLQGIHPKNIQSVNVKSPGPHCAQTEVIATLKNGRKACLNPASPIVKKIIEKMLNSDKSN^{C}
GROα – R8A	^{N}ATEL**A**CQCLQTLQGIHPKNIQSVNVKSPGPHCAQTEVIATLKNGRKACLNPASPIVKKIIEKMLNSDKSN^{C}
GROα – Q10A	^{N}ATELRC**A**CLQTLQGIHPKNIQSVNVKSPGPHCAQTEVIATLKNGRKACLNPASPIVKKIIEKMLNSDKSN^{C}
GROα – L12A	^{N}ATELRCQC**A**QTLQGIHPKNIQSVNVKSPGPHCAQTEVIATLKNGRKACLNPASPIVKKIIEKMLNSDKSN^{C}
GROα – Q13A	^{N}ATELRCQCL**A**TLQGIHPKNIQSVNVKSPGPHCAQTEVIATLKNGRKACLNPASPIVKKIIEKMLNSDKSN^{C}
GROα – T14A	^{N}ATELRCQCLQ**A**LQGIHPKNIQSVNVKSPGPHCAQTEVIATLKNGRKACLNPASPIVKKIIEKMLNSDKSN^{C}
GROα – L15A	^{N}ATELRCQCLQT**A**QGIHPKNIQSVNVKSPGPHCAQTEVIATLKNGRKACLNPASPIVKKIIEKMLNSDKSN^{C}
GROα – Q16A	^{N}ATELRCQCLQTL**A**GIHPKNIQSVNVKSPGPHCAQTEVIATLKNGRKACLNPASPIVKKIIEKMLNSDKSN^{C}
GROα – G17A	^{N}ATELRCQCLQTLQ**A**IHPKNIQSVNVKSPGPHCAQTEVIATLKNGRKACLNPASPIVKKIIEKMLNSDKSN^{C}
GROα – I18A	^{N}ATELRCQCLQTLQG**A**HPKNIQSVNVKSPGPHCAQTEVIATLKNGRKACLNPASPIVKKIIEKMLNSDKSN^{C}
GROα – H19A	^{N}ATELRCQCLQTLQGI**A**PKNIQSVNVKSPGPHCAQTEVIATLKNGRKACLNPASPIVKKIIEKMLNSDKSN^{C}

GROα – K21A	^{N}ATELRCQCLQTLQGIHP**A**NIQSVNVKSPGPHCAQTEVIATLKNGRKACLNPASPIVKKIIEKMLNSDKSN^{C}
GROα – N22A	^{N}ATELRCQCLQTLQGIHPK**A**IQSVNVKSPGPHCAQTEVIATLKNGRKACLNPASPIVKKIIEKMLNSDKSN^{C}
GROα – Q24A	^{N}ATELRCQCLQTLQGIHPKNI**A**SVNVKSPGPHCAQTEVIATLKNGRKACLNPASPIVKKIIEKMLNSDKSN^{C}
GROα – S25A	^{N}ATELRCQCLQTLQGIHPKNIQ**A**VNVKSPGPHCAQTEVIATLKNGRKACLNPASPIVKKIIEKMLNSDKSN^{C}
GROα – V26A	^{N}ATELRCQCLQTLQGIHPKNIQS**A**NVKSPGPHCAQTEVIATLKNGRKACLNPASPIVKKIIEKMLNSDKSN^{C}
GROα – N27A	^{N}ATELRCQCLQTLQGIHPKNIQSV**A**VKSPGPHCAQTEVIATLKNGRKACLNPASPIVKKIIEKMLNSDKSN^{C}
GROα – V28A	^{N}ATELRCQCLQTLQGIHPKNIQSVN**A**KSPGPHCAQTEVIATLKNGRKACLNPASPIVKKIIEKMLNSDKSN^{C}
GROα – K29A	^{N}ATELRCQCLQTLQGIHPKNIQSVNV**A**SPGPHCAQTEVIATLKNGRKACLNPASPIVKKIIEKMLNSDKSN^{C}
GROα – S30A	^{N}ATELRCQCLQTLQGIHPKNIQSVNVK**A**PGPHCAQTEVIATLKNGRKACLNPASPIVKKIIEKMLNSDKSN^{C}
GROα – G32A	^{N}ATELRCQCLQTLQGIHPKNIQSVNVKSP**A**PHCAQTEVIATLKNGRKACLNPASPIVKKIIEKMLNSDKSN^{C}
GROα – H34A	^{N}ATELRCQCLQTLQGIHPKNIQSVNVKSPGP**A**CAQTEVIATLKNGRKACLNPASPIVKKIIEKMLNSDKSN^{C}
GROα – A36S	^{N}ATELRCQCLQTLQGIHPKNIQSVNVKSPGPHC**S**QTEVIATLKNGRKACLNPASPIVKKIIEKMLNSDKSN^{C}
GROα – Q37A	^{N}ATELRCQCLQTLQGIHPKNIQSVNVKSPGPHCA**A**TEVIATLKNGRKACLNPASPIVKKIIEKMLNSDKSN^{C}
GROα – T38A	^{N}ATELRCQCLQTLQGIHPKNIQSVNVKSPGPHCAQ**A**EVIATLKNGRKACLNPASPIVKKIIEKMLNSDKSN^{C}
GROα – E39A	^{N}ATELRCQCLQTLQGIHPKNIQSVNVKSPGPHCAQT**A**VIATLKNGRKACLNPASPIVKKIIEKMLNSDKSN^{C}
GROα – I41A	^{N}ATELRCQCLQTLQGIHPKNIQSVNVKSPGPHCAQTEV**A**ATLKNGRKACLNPASPIVKKIIEKMLNSDKSN^{C}
GROα – T43A	^{N}ATELRCQCLQTLQGIHPKNIQSVNVKSPGPHCAQTEVIA**A**LKNGRKACLNPASPIVKKIIEKMLNSDKSN^{C}
GROα – L44A	^{N}ATELRCQCLQTLQGIHPKNIQSVNVKSPGPHCAQTEVIAT**A**KNGRKACLNPASPIVKKIIEKMLNSDKSN^{C}

GROα – K45A	^{N}ATELRCQCLQTLQGIHPKNIQSVNVKSPGPHCAQTEVIATL**A**NGRKACLNPASPIVKKIIEKMLNSDKSN^{C}
GROα – N46A	^{N}ATELRCQCLQTLQGIHPKNIQSVNVKSPGPHCAQTEVIATLK**A**GRKACLNPASPIVKKIIEKMLNSDKSN^{C}
GROα – G47A	^{N}ATELRCQCLQTLQGIHPKNIQSVNVKSPGPHCAQTEVIATLKN**A**RKACLNPASPIVKKIIEKMLNSDKSN^{C}
GROα – R48A	^{N}ATELRCQCLQTLQGIHPKNIQSVNVKSPGPHCAQTEVIATLKNG**A**KACLNPASPIVKKIIEKMLNSDKSN^{C}
GROα – K49A	^{N}ATELRCQCLQTLQGIHPKNIQSVNVKSPGPHCAQTEVIATLKNGR**A**ACLNPASPIVKKIIEKMLNSDKSN^{C}
GROα – A50S	^{N}ATELRCQCLQTLQGIHPKNIQSVNVKSPGPHCAQTEVIATLKNGRK**S**CLNPASPIVKKIIEKMLNSDKSN^{C}
GROα – N53A	^{N}ATELRCQCLQTLQGIHPKNIQSVNVKSPGPHCAQTEVIATLKNGRKACL**A**PASPIVKKIIEKMLNSDKSN^{C}
GROα – A55S	^{N}ATELRCQCLQTLQGIHPKNIQSVNVKSPGPHCAQTEVIATLKNGRKACLNP**S**SPIVKKIIEKMLNSDKSN^{C}
GROα – S56A	^{N}ATELRCQCLQTLQGIHPKNIQSVNVKSPGPHCAQTEVIATLKNGRKACLNPA**A**PIVKKIIEKMLNSDKSN^{C}
GROα – I58A	^{N}ATELRCQCLQTLQGIHPKNIQSVNVKSPGPHCAQTEVIATLKNGRKACLNPASP**A**VKKIIEKMLNSDKSN^{C}
GROα – K60A	^{N}ATELRCQCLQTLQGIHPKNIQSVNVKSPGPHCAQTEVIATLKNGRKACLNPASPIV**A**KIIEKMLNSDKSN^{C}
GROα – K61A	^{N}ATELRCQCLQTLQGIHPKNIQSVNVKSPGPHCAQTEVIATLKNGRKACLNPASPIVK**A**IIEKMLNSDKSN^{C}
GROα – E64A	^{N}ATELRCQCLQTLQGIHPKNIQSVNVKSPGPHCAQTEVIATLKNGRKACLNPASPIVKKII**A**KMLNSDKSN^{C}
GROα – K65A	^{N}ATELRCQCLQTLQGIHPKNIQSVNVKSPGPHCAQTEVIATLKNGRKACLNPASPIVKKIIE**A**MLNSDKSN^{C}
GROα – M66A	^{N}ATELRCQCLQTLQGIHPKNIQSVNVKSPGPHCAQTEVIATLKNGRKACLNPASPIVKKIIEK**A**LNSDKSN^{C}
GROα – L67A	^{N}ATELRCQCLQTLQGIHPKNIQSVNVKSPGPHCAQTEVIATLKNGRKACLNPASPIVKKIIEKM**A**NSDKSN^{C}
GROα – N68A	^{N}ATELRCQCLQTLQGIHPKNIQSVNVKSPGPHCAQTEVIATLKNGRKACLNPASPIVKKIIEKML**A**SDKSN^{C}
GROα – S69A	^{N}ATELRCQCLQTLQGIHPKNIQSVNVKSPGPHCAQTEVIATLKNGRKACLNPASPIVKKIIEKMLN**A**DKSN^{C}

GROα – D70A	^{N}ATELRCQCLQTLQGIHPKNIQSVNVKSPGPHCAQTEVIATLKNGRKACLNPASPIVKKIIEKMLNS**A**KSN^{C}
GROα – K71A	^{N}ATELRCQCLQTLQGIHPKNIQSVNVKSPGPHCAQTEVIATLKNGRKACLNPASPIVKKIIEKMLNSD**A**SN^{C}
GROα – S72A	^{N}ATELRCQCLQTLQGIHPKNIQSVNVKSPGPHCAQTEVIATLKNGRKACLNPASPIVKKIIEKMLNSDK**A**N^{C}
GROα – N73A	^{N}ATELRCQCLQTLQGIHPKNIQSVNVKSPGPHCAQTEVIATLKNGRKACLNPASPIVKKIIEKMLNSDKS**A**^{C}

**Primer name**	**Primer forward sequence (5’ to 3’)**	**Primer reverse sequence (5’ to 3’)**
GROα – A4S	GCTCTTTGGACAAGAGATCCACCGAGCTGAGATGC	GCATCTCAGCTCGGTGGATCTCTTGTCCAAAGAGC
GROα – T5S	CTTTGGACAAGAGAGCCGCCGAGCTGAGATGCC	GGCATCTCAGCTCGGCGGCTCTCTTGTCCAAAG
GROα – E6A	GGACAAGAGAGCCACCGCCCTGAGATGCCAGTGCC	GGCACTGGCATCTCAGGGCGGTGGCTCTCTTGTCC
GROα – L7A	GACAAGAGAGCCACCGAGGCTAGATGCCAGTGCCTGC	GCAGGCACTGGCATCTAGCCTCGGTGGCTCTCTTGTC
GROα – R8A	GAGAGCCACCGAGCTGGCATGCCAGTGCCTGC	GCAGGCACTGGCATGCCAGCTCGGTGGCTCTC
GROα – Q10A	GAGCCACCGAGCTGAGATGCGCTTGCCTGCAGACCCTGCAGGG	CCCTGCAGGGTCTGCAGGCAAGCGCATCTCAGCTCGGTGGCTC
GROα – L12A	GCTGAGATGCCAGTGCGCTCAGACCCTGCAGGGC	GCCCTGCAGGGTCTGAGCGCACTGGCATCTCAGC
GROα – Q13A	GCTGAGATGCCAGTGCCTGGCTACCCTGCAGGGCATCCAC	GTGGATGCCCTGCAGGGTAGCCAGGCACTGGCATCTCAGC
GROα – T14A	GCCAGTGCCTGCAGGCCCTGCAGGGCATC	GATGCCCTGCAGGGCCTGCAGGCACTGGC
GROα – L15A	GCCAGTGCCTGCAGACCGCTCAGGGCATCCACCCCAAG	CTTGGTGGATGCCCTGAGCGGTCTGCAGGCACTGGC
GROα – Q16A	CAGTGCCTGCAGACCCTGGCTGGCATCCACCCCAAGAAC	GTTCTTGGGGTGGATGCCAGCCAGGGTCTGCAGGCACTG

GROα – G17A	GCAGACCCTGCAGGCCATCCACCCCAAG	CTTGGGGTGGATGGCCTGCAGGGTCTGC
GROα – I18A	GCAGACCCTGCAGGGCGCCCACCCCAAGAACATC	GATGTTCTTGGGGTGGGCGCCCTGCAGGGTCTGC
GROα – H19A	GACCCTGCAGGGCATCGCCCCCAAGAACATCCAG	CTGGATGTTCTTGGGGGCGATGCCCTGCAGGGTC
GROα – K21A	GCAGGGCATCCACCCCGCTAACATCCAGAGCGTGAACG	CGTTCACGCTCTGGATGTTAGCGGGGTGGATGCCCTGC
GROα – N22A	CAGGGCATCCACCCCAAGGCCATCCAGAGCGTGAACG	CGTTCACGCTCTGGATGGCCTTGGGGTGGATGCCCTG
GROα – Q24A	GCATCCACCCCAAGAACATCGCTAGCGTGAACGTGAAGTCC	GGACTTCACGTTCACGCTAGCGATGTTCTTGGGGTGGATGC
GROα – S25A	CACCCCAAGAACATCCAGGCCGTGAACGTGAAGTCCC	GGGACTTCACGTTCACGGCCTGGATGTTCTTGGGGTG
GROα – V26A	CACCCCAAGAACATCCAGAGCGCTAACGTGAAGTCCCCTGGCCCC	GGGGCCAGGGGACTTCACGTTAGCGCTCTGGATGTTCTTGGGGTG
GROα – N27A	GAACATCCAGAGCGTGGCCGTGAAGTCCCCTGG	CCAGGGGACTTCACGGCCACGCTCTGGATGTTC
GROα – V28A	GAACATCCAGAGCGTGAACGCTAAGTCCCCTGGCCCCCACTG	CAGTGGGGGCCAGGGGACTTAGCGTTCACGCTCTGGATGTTC
GROα – K29A	CCAGAGCGTGAACGTGGCTTCCCCTGGCCCCCACTG	CAGTGGGGGCCAGGGGAAGCCACGTTCACGCTCTGG
GROα – S30A	GCGTGAACGTGAAGGCCCCTGGCCCCCAC	GTGGGGGCCAGGGGCCTTCACGTTCACGC
GROα – G32A	GAACGTGAAGTCCCCTGCCCCCCACTGCGCCCAG	CTGGGCGCAGTGGGGGGCAGGGGACTTCACGTTC
GROα – H34A	GTGAAGTCCCCTGGCCCCGCCTGCGCCCAGACCGAAG	CTTCGGTCTGGGCGCAGGCGGGGCCAGGGGACTTCAC
GROα – A36S	CCTGGCCCCCACTGCTCCCAGACCGAAGTG	CACTTCGGTCTGGGAGCAGTGGGGGCCAGG
GROα – Q37A	CTGGCCCCCACTGCGCCGCTACCGAAGTGATCGCCAC	GTGGCGATCACTTCGGTAGCGGCGCAGTGGGGGCCAG
GROα – T38A	GCCCCCACTGCGCCCAGGCCGAAGTGATCGCCAC	GTGGCGATCACTTCGGCCTGGGCGCAGTGGGGGC
GROα – E39A	CCACTGCGCCCAGACCGCAGTGATCGCCACCCTG	CAGGGTGGCGATCACTGCGGTCTGGGCGCAGTGG

GROα – I41A	GCGCCCAGACCGAAGTGGCCGCCACCCTGAAGAACG	CGTTCTTCAGGGTGGCGGCCACTTCGGTCTGGGCGC
GROα – T43A	GACCGAAGTGATCGCCGCCCTGAAGAACGGCCG	CGGCCGTTCTTCAGGGCGGCGATCACTTCGGTC
GROα – L44A	CCGAAGTGATCGCCACCGCTAAGAACGGCCGGAAGGC	GCCTTCCGGCCGTTCTTAGCGGTGGCGATCACTTCGG
GROα – K45A	CGAAGTGATCGCCACCCTGGCTAACGGCCGGAAGGCCTGC	GCAGGCCTTCCGGCCGTTAGCCAGGGTGGCGATCACTTCG
GROα – N46A	GTGATCGCCACCCTGAAGGCCGGCCGGAAGGCCTGCC	GGCAGGCCTTCCGGCCGGCCTTCAGGGTGGCGATCAC
GROα – G47A	CGCCACCCTGAAGAACGCCCGGAAGGCCTGCCTG	CAGGCAGGCCTTCCGGGCGTTCTTCAGGGTGGCG
GROα – R48A	GCCACCCTGAAGAACGGCGCTAAGGCCTGCCTGAACC	GGTTCAGGCAGGCCTTAGCGCCGTTCTTCAGGGTGGC
GROα – K49A	CCTGAAGAACGGCCGGGCTGCCTGCCTGAACCCCGC	GCGGGGTTCAGGCAGGCAGCCCGGCCGTTCTTCAGG
GROα – A50S	GAAGAACGGCCGGAAGTCCTGCCTGAACCCCGC	GCGGGGTTCAGGCAGGACTTCCGGCCGTTCTTC
GROα – N53A	GCCGGAAGGCCTGCCTGGCCCCCGCCAGCCCCATCG	CGATGGGGCTGGCGGGGGCCAGGCAGGCCTTCCGGC
GROα – A55S	GCCTGCCTGAACCCCTCCAGCCCCATCGTG	CACGATGGGGCTGGAGGGGTTCAGGCAGGC
GROα – S56A	GCCTGCCTGAACCCCGCCGCCCCCATCGTGAAGAAAATC	GATTTTCTTCACGATGGGGGCGGCGGGGTTCAGGCAGGC
GROα – I58A	CTGAACCCCGCCAGCCCCGCCGTGAAGAAAATCATCG	CGATGATTTTCTTCACGGCGGGGCTGGCGGGGTTCAG
GROα – K60A	CCCGCCAGCCCCATCGTGGCTAAAATCATCGAGAAGATGC	GCATCTTCTCGATGATTTTAGCCACGATGGGGCTGGCGGG
GROα – K61A	GCCAGCCCCATCGTGAAGGCAATCATCGAGAAGATGC	GCATCTTCTCGATGATTGCCTTCACGATGGGGCTGGC
GROα – E64A	CATCGTGAAGAAAATCATCGCTAAGATGCTGAACAGCGACAAGAG	CTCTTGTCGCTGTTCAGCATCTTAGCGATGATTTTCTTCACGATG
GROα – K65A	CATCGTGAAGAAAATCATCGAGGCCATGCTGAACAGCGACAAGAG	CTCTTGTCGCTGTTCAGCATGGCCTCGATGATTTTCTTCACGATG
GROα – M66A	GTGAAGAAAATCATCGAGAAGGCCCTGAACAGCGACAAGAGCAAC	GTTGCTCTTGTCGCTGTTCAGGGCCTTCTCGATGATTTTCTTCAC

GROα – L67A	GAAAATCATCGAGAAGATGGCTAACAGCGACAAGAGCAACGGCGG	CCGCCGTTGCTCTTGTCGCTGTTAGCCATCTTCTCGATGATTTTC
GROα – N68A	GAAAATCATCGAGAAGATGCTGGCCAGCGACAAGAGCAACGGCGG	CCGCCGTTGCTCTTGTCGCTGGCCAGCATCTTCTCGATGATTTTC
GROα – S69A	AATCATCGAGAAGATGCTGAACGCCGACAAGAGCAACGGCGGAG	CTCCGCCGTTGCTCTTGTCGGCGTTCAGCATCTTCTCGATGATT
GROα – D70A	GAAGATGCTGAACAGCGCCAAGAGCAACGGCGG	CCGCCGTTGCTCTTGGCGCTGTTCAGCATCTTC
GROα – K71A	GAAGATGCTGAACAGCGACGCTAGCAACGGCGGAGGCGAAC	GTTCGCCTCCGCCGTTGCTAGCGTCGCTGTTCAGCATCTTC
GROα – S72A	GCTGAACAGCGACAAGGCCAACGGCGGAGGCG	CGCCTCCGCCGTTGGCCTTGTCGCTGTTCAGC
GROα – N73A	GCTGAACAGCGACAAGAGCGCCGGCGGAGGCGAACAAAAGC	GCTTTTGTTCGCCTCCGCCGGCGCTCTTGTCGCTGTTCAGC
